# Supplementary material for: Palladium-catalyzed difluorocarbene transfer enables access to enantioenriched chiral spirooxindoles
Source: Nat Commun. 2024 Oct 1;15:8510. doi: 10.1038/s41467-024-52392-5 (PMC11445564; doi:10.1038/s41467-024-52392-5)
Supplement: Supplementary file 1 — Supplementary Information [file 41467_2024_52392_MOESM1_ESM.pdf]

Supplementary Information

Palladium-catalyzed difluorocarbene transfer enables access to enantioenriched chiral spirooxindoles

Zhiwen Nie,‡ Keqin Wu,‡ Xiaohang Zhan,‡ Weiran Yang, Zhong Lian, Shaoquan Lin, Shou-Guo Wang and Qin Yin*

Table of contents

Supplementary Methods 2

1. General information 2

2. General procedures for the synthesis of substrates 3

3 Reaction optimization 20

4. General procedures for the synthesis of chiral spirooxindoles .26

5. Synthetic applications 43

Supplementary Discussion 49

6. Control experiments 49

Supplementary Notes 59

7. NMR spectra 59

8. HPLC spectra 138

9. Crystallographic information 170

Supplementary References 173

10. References 173

Supplementary Methods

1. General information

Unless otherwise mentioned, all reagents and solvents were purchased from commercial suppliers (Energy Chemical, Adamas-beta®, Bide Pharmatech, Leyan, J&K, TCI, Tansoole and Laajoo) and used without further purification. All ligands were purchased from Bide Pharmatech (a Chinese supplier). All reactions were assembled on a Schlenk vacuum line or in a glovebox using an oven-dried glass tube, stirred with Teflon-coated magnetic stirring bars, and heated by oil bath unless otherwise specified. Reactions were monitored using thin-layer chromatography (TLC), and visualization of the developed plates was performed under UV light (254 nm). Purification and isolation of products were performed via silica gel (300-400 mesh) chromatography. ^{1}H, ^{13}C, ^{19}F and ^{31}P NMR spectra were recorded on Bruker AscendTM 400 MHz (^{1}H: 400 MHz, ^{13}C: 100 MHz, ^{19}F: 376 MHz, ^{31}P: 162 MHz) or 600 MHz (^{1}H: 600 MHz, ^{13}C: 150 MHz). ^{1}H NMR and ^{13}C NMR spectra were internally referenced to the residual solvent signal ($CHCl_3$: δ = 7.26 ppm for ^{1}H NMR and $CDCl_3$: δ = 77.16 ppm for ^{13}C NMR; DMSO-d_6: δ = 2.50 ppm for ^{1}H NMR and DMSO-d_6: δ = 39.52 ppm for ^{13}C NMR; C_6D_6: δ = 7.16 ppm for ^{1}H NMR and C_6D_6: δ = 128.06 ppm for ^{13}C NMR). Data are reported as follows: chemical shift (δ ppm), multiplicity (s = singlet, d = doublet, t = triplet, q = quartet, dd = doublet of doublet, dt = doublet of triplet, ddd = doublet of doublet of doublet, m = multiplet), coupling constants (Hz) and integration. Enantiomeric excess values were determined with Shimadzu LC-20A or Shimadzu LC-40A series HPLC instrument. GC-MS spectra were measured with GC-MS QP-2030. Optical rotations were measured using a 1 mL cell with a 1 dm path length on a Rudolph Autopol I polarimeter at 589 nm. The HR-ESI-MS spectra were measured using SCIEX X500R and Waters Xevo G2-XS QTOF mass spectrometer. X-ray diffraction was measured on a Bruker APEX-II CCD diffractometer with Cu–Kα radiation.

2. General procedures for the synthesis of substrates

Procedure A[1]

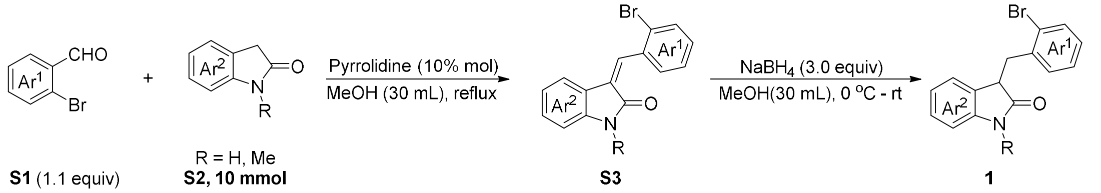

To a stirred solution of 2-oxindole **S2** (10.0 mmol, 1.0 equiv) in methanol (30 mL) was added 2-bromobenzaldehyde **S1** (11.0 mmol, 1.1 equiv) and 10 mol% of pyrrolidine (85 μL, 1.0 mmol, 0.1 equiv). The solution was heated to reflux for 3 h. After the mixture was cooled to room temperature, the precipitate was filtered, washed with cold methanol and the crude product was treated with sodium hydroborate (1.13 g, 30.0 mmol, 3.0 equiv) carefully over portionwise in methanol at 0 °C. The solution was quenched by satd. aq. NH_4Cl and extracted with ethyl acetate. The organic layer was dried over anhydrous sodium sulfate and evaporated to give a residue which was purified by column chromatography on silica gel (petroleum ether/ethyl acetate = 2/1) or recrystallization from ethanol to afford the desired product **1**.

Procedure B

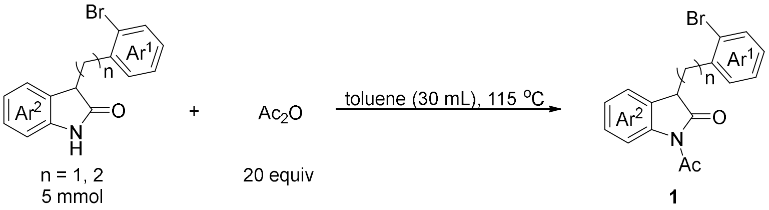

To a solution of NH oxindole-tethered aryl bromides (5.0 mmol, 1.0 equiv) in toluene (30 mL) was added acetic anhydride (10.21 g, 100.0 mmol, 20.0 equiv) at room temperature and stirred at 115 °C for 12 h. After completion of the reaction, the resulting solution was cooled to room temperature and quenched by satd. aq. $NaHCO_3$ carefully, then extracted with ethyl acetate. The organic layer was separated and dried over anhydrous sodium sulfate. The solvent was concentrated in vacuo and purified by flash chromatography on silica gel (eluent: petroleum ether/ethyl acetate = 20/1) or recrystallization from hexane/CH_2Cl_2 to afford the title compound.

Procedure C

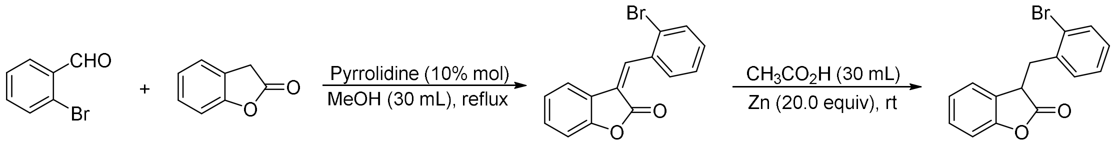

S1 (1.1 equiv) **S4**, 10 mmol **S5** **1x**

Step 1:

To a stirred solution of benzofuran-2(3*H*)-one **S4** (1.34 g, 10.0 mmol, 1.0 equiv) in methanol (30 mL) was added 2-bromobenzaldehyde **S1** (2.04 g, 11.0 mmol, 1.1 equiv) and 10 mol% of pyrrolidine (85 μL, 1.0 mmol, 0.1 equiv). The solution was heated to reflux for 3 h. After the mixture was cooled to room temperature, the precipitate was filtered, and washed with cold methanol and the crude product was used in the next step without further purification.

Step 2:

To a stirred solution of 3-(2-bromobenzylidene)benzofuran-2(3*H*)-one S5 (10.0 mmol, 1.0 equiv) in AcOH (30 mL) was added Zn (13.0 g, 200.0 mmol, 20.0 equiv) at room temperature. The reaction mixture was filtered and extracted with ethyl acetate. The residue was evaporated with vacuum distillation to remove the residual AcOH. The resulting residue was then purified by flash chromatography on silica gel (eluent: petroleum ether/ethyl acetate = 20/1) to obtain the corresponding product **1x** (2.78 g, 92% yield) as a white solid.

Procedure D[2]

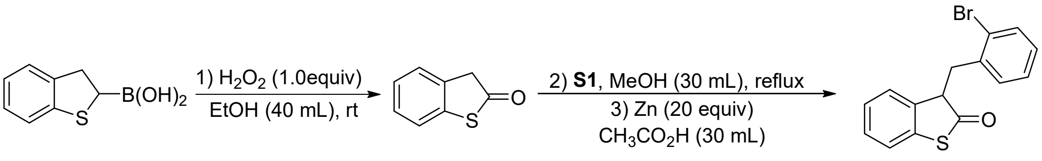

S6, 20mmol **S7** **1y**

Step 1:

To a stirred solution of benzo[*b*]thiophen-2-ylboronic acid **S6** (3.56 g, 20.0 mmol, 1.0 equiv) in ethanol (40 mL) was added H_2O_2 (30%, 20.0 mmol, 1.0 equiv) dropwise. Reaction was stirred overnight at room temperature. Then solvent was carefully evaporated with vacuum distillation. The residue was suspended in water and extracted with ethyl acetate. The collected organic phases were washed with brine and dried over anhydrous sodium sulfate. The mixture was filtered and the solvent was evaporated in

vacuo. The residue was purified by flash chromatography on silica gel (eluent: petroleum ether/ethyl acetate = 5/1) to afford the desired product **S7** (2.80 g, 93% yield) as a yellow solid.

Step 2 and **Step 3** refer to General **Procedure C**.

Procedure E

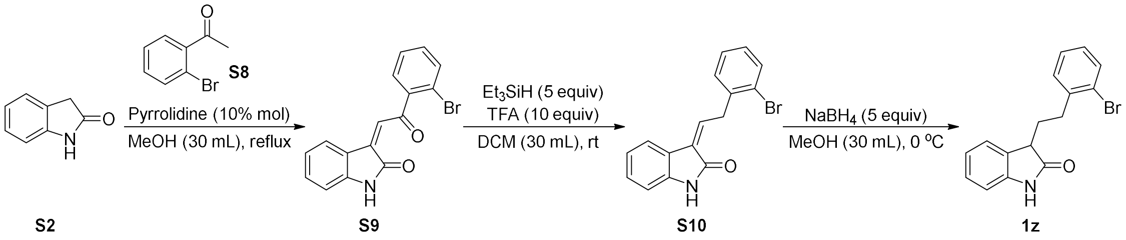

Step 1:

To a stirred solution of benzofuran-2(3*H*)-one **S2** (1.34 g, 10.0 mmol, 1.0 equiv) in methanol (30 mL) was added 2'-bromoacetophenone **S8** (2.04 g, 11.0 mmol, 1.1 equiv) and 10 mol% of pyrrolidine (85 μL, 1.0 mmol, 0.1 equiv). The solution was heated to reflux for 3 h. After the mixture was cooled to room temperature, the precipitate was filtered, washed with cold methanol and the crude product was used in the next step without further purification.

Step 2:

To a stirred solution of 3-(2-(2-bromophenyl)-2-oxoethylidene)indolin-2-one **S9** (10.0 mmol, 1.0 equiv) in CH_2Cl_2 (30 mL) was added Et_3SiH (5.81 g, 50 mmol, 5.0 equiv) and TFA (11.4 g, 100.0 mmol, 10.0 equiv) at room temperature. The reaction mixture was stirred overnight at room temperature. Upon completion, the solution was quenched by satd. aq. $NaHCO_3$ and extracted with ethyl acetate. The organic layer was dried over anhydrous sodium sulfate, and evaporated to give a residue, which was purified by column chromatography on silica gel (petroleum ether/ethyl acetate = 6/1) to afford the desired product **S10** (1.98 g, 63% yield) as a white solid.

Step 3:

To a stirred solution of 3-(2-(2-bromophenyl)ethylidene)indolin-2-on **S10** (6.3 mmol, 1.0 equiv) in MeOH (30 mL) was added $NaBH_4$ (1.19 g, 31.5 mmol, 5.0 equiv) at 0 °C. The reaction mixture was stirred for 4 h at 0 °C. Upon completion, the solution was quenched by satd. aq. NH_4Cl and extracted with ethyl acetate. The organic layer was

dried over anhydrous sodium sulfate and evaporated to give a residue, which was purified by column chromatography on silica gel (petroleum ether/ethyl acetate = 4/1) to afford the desired product **1z** (1.90 g, 95% yield) as a white solid.

Characterization data of 1a-1aa

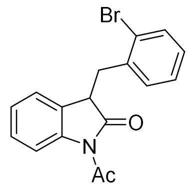

Chemical Formula: $C_{17}H_{14}BrNO_2$
Exact Mass: 343.0208

1-acetyl-3-(2-bromobenzyl)indolin-2-one (1a). Purification by column chromatography (eluent: petroleum ether/ethyl acetate = 20/1), 92% isolated yield (white solid, 1.59 g). **^{1}H NMR** (400 MHz, $CDCl_3$) δ 8.23 (d, *J* = 8.3 Hz, 1 H), 7.62 (dd, *J* = 7.9, 1.3 Hz, 1 H), 7.33 – 7.26 (m, 2 H), 7.23 – 7.15 (m, 2 H), 7.06 (td, *J* = 7.5, 1.1 Hz, 1 H), 6.76 (d, *J* = 7.5 Hz, 1 H), 4.08 (dd, *J* = 9.1, 6.2 Hz, 1 H), 3.54 (dd, *J* = 13.8, 6.2 Hz, 1 H), 3.08 (dd, *J* = 13.9, 9.1 Hz, 1 H), 2.69 (s, 3 H) ppm. **^{13}C {H} NMR** (100 MHz, $CDCl_3$) δ 177.6, 171.1, 140.4, 137.0, 133.3, 132.2, 129.0, 128.6, 127.52, 127.46, 125.0, 124.9, 124.4, 116.6, 45.4, 38.5, 26.9 ppm. HRMS (ESI) calculated for $[M+H]^+$ $C_{17}H_{15}BrNO_2^+$: 344.0281, found: 344.0285.

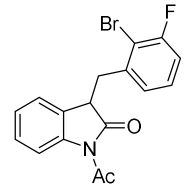

Chemical Formula: $C_{17}H_{13}BrFNO_2$
Exact Mass: 361.0114

1-acetyl-3-(2-bromo-3-fluorobenzyl)indolin-2-one (1b). Purification by column chromatography (eluent: petroleum ether/ethyl acetate = 20/1), 85% isolated yield (white solid, 1.54 g). **^{1}H NMR** (400 MHz, $CDCl_3$) δ 8.15 (d, *J* = 8.2 Hz, 1 H), 7.25 – 7.15 (m, 2 H), 7.00 (tdd, *J* = 7.6, 5.2, 1.3 Hz, 2 H), 6.94 (dt, *J* = 7.7, 1.2 Hz, 1 H), 6.72 (dd, *J* = 7.5, 1.4 Hz, 1 H), 3.97 (dd, *J* = 8.8, 6.3 Hz, 1 H), 3.47 (dd, *J* = 13.9, 6.3 Hz, 1 H), 3.06 (dd, *J* = 13.9, 8.8 Hz, 1 H), 2.60 (s, 3 H) ppm. **^{19}F NMR** (376 MHz, $CDCl_3$) δ -103.51 ppm. **^{13}C {H} NMR** (100 MHz, $CDCl_3$) δ 177.4, 171.0, 159.5 (d, *J* = 247.2 Hz),

140.4, 139.5, 128.7, 128.3 (d, J = 8.2 Hz), 127.3 (d, J = 3.3 Hz), 127.2, 125.0, 124.3, 116.7, 115.3 (d, J = 22.7 Hz), 112.0 (d, J = 20.4 Hz), 45.3, 38.1 (d, J = 2.7 Hz), 26.8 ppm. HRMS (ESI) calculated for $[M+H]^+$ $C_{17}H_{14}BrFNO_2^+$: 362.0186, found: 362.0188.

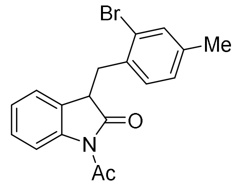

Chemical Formula: $C_{18}H_{16}BrNO_2$
Exact Mass: 357.0364

1-acetyl-3-(2-bromo-4-methylbenzyl)indolin-2-one (1c). Purification by column chromatography (eluent: petroleum ether/ethyl acetate = 20/1), 90% isolated yield (white solid, 1.61 g). **^{1}H NMR** (400 MHz, $CDCl_3$) δ 8.23 (d, J = 8.1 Hz, 1 H), 7.45 (s, 1 H), 7.29 (t, J = 7.9 Hz, 1 H), 7.11 – 7.05 (m, 3 H), 6.80 (d, J = 8.9 Hz, 1 H), 4.08 – 4.01 (m, 1 H), 3.48 (dd, J = 13.9, 6.3 Hz, 1 H), 3.05 (dd, J = 13.8, 9.0 Hz, 1 H), 2.69 (s, 3 H), 2.35 (s, 3 H) ppm. **^{13}C {H} NMR** (100 MHz, $CDCl_3$) δ 177.7, 171.1, 140.4, 139.2, 133.8, 133.7, 131.9, 128.5, 128.3, 127.6, 124.9, 124.7, 124.4, 116.6, 45.6, 38.1, 26.9, 20.8 ppm. HRMS (ESI) calculated for $[M+H]^+$ $C_{18}H_{17}BrNO_2^+$: 358.0437, found: 358.0436.

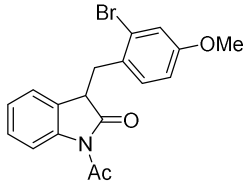

Chemical Formula: $C_{18}H_{16}BrNO_3$
Exact Mass: 373.0314

1-acetyl-3-(2-bromo-4-methoxybenzyl)indolin-2-one (1d). Recrystallization from hexane/CH_2Cl_2 to give the product, 72% isolated yield (white solid, 1.34 g). **^{1}H NMR** (400 MHz, $CDCl_3$) δ 8.22 (d, J = 8.4 Hz, 1 H), 7.29 (td, J = 8.4, 1.2 Hz, 1 H), 7.16 (d, J = 2.7 Hz, 1 H), 7.11 – 7.04 (m, 2 H), 6.82 (dd, J = 8.5, 2.6 Hz, 1 H), 6.78 (d, J = 7.6 Hz, 1 H), 4.01 (dd, J = 9.0, 6.2 Hz, 1 H), 3.81 (s, 3 H), 3.47 (dd, J = 14.0, 6.2 Hz, 1 H), 3.01 (dd, J = 14.0, 9.0 Hz, 1 H), 2.68 (s, 3 H) ppm. **^{13}C {H} NMR** (100 MHz, $CDCl_3$) δ 177.7, 171.1, 159.4, 140.4, 132.5, 128.8, 128.5, 127.6, 125.1, 124.8, 124.4, 118.4, 116.6, 113.6, 55.7, 45.7, 37.7, 26.8 ppm. HRMS (ESI) calculated for $[M+H]^+$

$C_{18}H_{17}BrNO_3^+$: 374.0386, found: 374.0382.

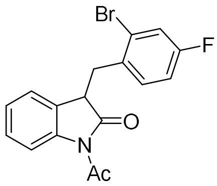

Chemical Formula: $C_{17}H_{13}BrFNO_2$
Exact Mass: 361.0114

1-acetyl-3-(2-bromo-4-fluorobenzyl)indolin-2-one (1e). Purification by column chromatography (eluent: petroleum ether/ethyl acetate = 20/1), 88% isolated yield (white solid, 1.60 g). **^{1}H NMR** (400 MHz, $CDCl_3$) δ 8.22 (dd, J = 8.2, 1.0 Hz,1 H), 7.37 (dd, J = 8.2, 2.7 Hz, 1 H), 7.30 (t, J = 8.0 Hz, 1 H), 7.18 (dd, J = 8.6, 6.0 Hz, 1 H), 7.09 (td, J = 7.6, 1.1 Hz, 1 H), 7.01 (td, J = 8.2, 2.6 Hz, 1 H), 6.80 (d, J = 7.6 Hz, 1 H), 4.01 (dd, J = 8.8, 6.3 Hz, 1 H), 3.48 (dd, J = 14.0, 6.5 Hz, 1 H), 3.09 (dd, J = 14.0, 8.7 Hz, 1 H), 2.68 (s, 3 H) ppm. **^{19}F NMR** (376 MHz, $CDCl_3$) δ -103.51 ppm. **^{13}C {H} NMR** (100 MHz, $CDCl_3$) δ 177.5, 171.0, 161.7 (d, J = 250.7 Hz), 140.4, 132.900 (d, J = 8.1 Hz), 132.895, 128.7, 127.3, 125.0, 124.8 (d, J = 9.5 Hz), 124.3, 120.5 (d, J = 24.3 Hz), 116.7, 114.7 (d, J = 20.8 Hz), 45.5, 37.7, 26.8 ppm. HRMS (ESI) calculated for $[M+H]^+$ $C_{17}H_{14}BrFNO_2^+$: 362.0186, found: 362.0185.

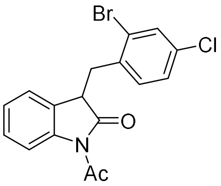

Chemical Formula: $C_{17}H_{13}BrClNO_2$
Exact Mass: 376.9818

1-acetyl-3-(2-bromo-4-chlorobenzyl)indolin-2-one (1f). Purification by column chromatography (eluent: petroleum ether/ethyl acetate = 20/1), 90% isolated yield (white solid, 1.70 g). **^{1}H NMR** (400 MHz, $CDCl_3$) δ 8.14 (d, J = 8.2 Hz, 1 H), 7.55 (d, J = 2.2 Hz, 1 H), 7.26 – 7.17 (m, 2 H), 7.06 (d, J = 8.2 Hz, 1 H), 7.01 (tt, J = 7.6, 0.8 Hz, 1 H), 6.74 (dt, J = 7.5, 1.1 Hz, 1 H), 3.93 (dd, J = 8.6, 6.5 Hz, 1 H), 3.38 (dd, J = 14.0, 6.5 Hz, 1 H), 3.00 (dd, J = 14.0, 8.7 Hz, 1 H), 2.60 (s, 3 H) ppm. **^{13}C {H} NMR** (100 MHz, $CDCl_3$) δ 177.4, 171.0, 140.4, 135.6, 133.9, 132.9, 132.8, 128.7, 127.8, 127.2, 125.2, 125.0, 124.2, 116.7, 45.3, 37.9, 26.8 ppm. HRMS (ESI) calculated for

[M+H]$^+$ $C_{17}H_{14}BrClNO_2^+$: 377.9891, found: 377.9886.

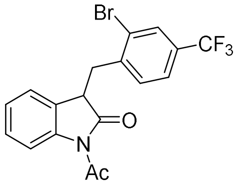

Chemical Formula: $C_{18}H_{13}BrF_3NO_2$
Exact Mass: 411.0082

1-acetyl-3-(2-bromo-4-(trifluoromethyl)benzyl)indolin-2-one (1g). Recrystallization from hexane/CH_2Cl_2 to give the product, 66% isolated yield (white solid, 1.36 g). **^{1}H NMR** (400 MHz, $CDCl_3$) δ 8.22 (d, *J* = 8.2 Hz, 1 H), 7.88 (s, 1 H), 7.55 (d, *J* = 8.1 Hz, 1 H), 7.36 (d, *J* = 8.0 Hz, 1 H), 7.31 (t, *J* = 7.9 Hz, 1 H), 7.10 (t, *J* = 7.6 Hz, 1 H), 6.85 (d, *J* = 7.5 Hz, 1 H), 4.04 (t, *J* = 7.5 Hz, 1 H), 3.51 (dd, *J* = 13.9, 6.7 Hz, 1 H), 3.19 (dd, *J* = 13.9, 8.4 Hz, 1 H), 2.66 (s, 3 H) ppm. **^{19}F NMR** (376 MHz, $CDCl_3$) δ -62.57 ppm. **^{13}C {H} NMR** (100 MHz, $CDCl_3$) δ 177.2, 170.9, 141.3, 140.4, 132.4, 131.2 (q, *J* = 33.2 Hz), 130.2 (q, *J* = 3.9 Hz), 128.8, 127.0, 125.05, 125.02, 124.3 (q, *J* = 3.7 Hz), 123.2 (q, *J* = 273.7 Hz), 124.0, 116.7, 45.0, 38.3, 26.7 ppm. HRMS (ESI) calculated for [M+H]$^+$ $C_{18}H_{13}BrF_3NO_2^+$: 412.0155, found: 412.0159.

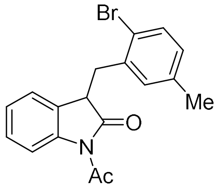

Chemical Formula: $C_{18}H_{16}BrNO_2$
Exact Mass: 357.0364

1-acetyl-3-(2-bromo-5-methylbenzyl)indolin-2-one (1h). Purification by column chromatography (eluent: petroleum ether/ethyl acetate = 20/1), 94% isolated yield (white solid, 1.67 g). **^{1}H NMR** (400 MHz, $CDCl_3$) δ 8.23 (d, *J* = 8.1 Hz, 1 H), 7.45 (s, 1 H), 7.29 (t, *J* = 7.9 Hz, 1 H), 7.11 – 7.05 (m, 3 H), 6.80 (d, *J* = 8.9 Hz, 1 H), 4.08 – 4.01 (m, 1 H), 3.48 (dd, *J* = 13.9, 6.3 Hz, 1 H), 3.05 (dd, *J* = 13.8, 9.0 Hz, 1 H), 2.69 (s, 3 H), 2.35 (s, 3 H) ppm. **^{13}C {H} NMR** (100 MHz, $CDCl_3$) δ 177.6, 171.1, 140.4, 137.4, 136.6, 133.0, 132.9, 129.8, 128.5, 127.5, 124.8, 124.5, 121.6, 116.6, 45.5, 38.4, 26.8, 21.0 ppm. HRMS (ESI) calculated for [M+H]$^+$ $C_{18}H_{17}BrNO_2^+$: 358.0437, found: 358.0435.

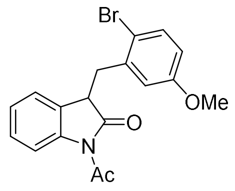

Chemical Formula: $C_{18}H_{16}BrNO_3$
Exact Mass: 373.0314

1-acetyl-3-(2-bromo-5-methoxybenzyl)indolin-2-one (1i). Recrystallization from hexane/CH_2Cl_2 to give the product, 70% isolated yield (white solid, 1.32 g). **^{1}H NMR** (400 MHz, $CDCl_3$) δ 8.22 (d, *J* = 8.2 Hz, 1 H), 7.48 (dd, 1 H), 7.29 (tdd, *J* = 8.3, 1.5, 0.7 Hz, 1 H), 7.07 (td, *J* = 7.5, 1.0 Hz, 1 H), 6.81 (d, *J* = 7.5 Hz, 1 H), 6.76 – 6.71 (m, 2 H), 4.05 (dd, *J* = 9.1, 6.0 Hz, 1 H), 3.75 (s, 3 H), 3.50 (dd, *J* = 13.8, 6.1 Hz, 1 H), 3.03 (dd, *J* = 13.8, 9.1 Hz, 1 H), 2.69 (s, 3 H) ppm. **^{13}C {H} NMR** (100 MHz, $CDCl_3$) δ 177.6, 171.0, 158.9, 140.4, 137.9, 133.8, 128.6, 127.4, 124.9, 124.5, 117.7, 116.6, 115.3, 114.7, 55.6, 45.5, 38.5, 26.8 ppm. HRMS (ESI) calculated for $[M+H]^+$ $C_{18}H_{17}BrNO_3^+$: 374.0386, found: 374.0390.

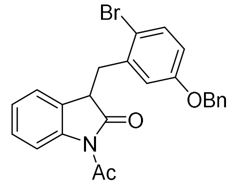

Chemical Formula: $C_{24}H_{20}BrNO_3$
Exact Mass: 449.0627

1-acetyl-3-(5-(benzyloxy)-2-bromobenzyl)indolin-2-one (1j). Recrystallization from hexane/CH_2Cl_2 to give the product, 78% isolated yield (white solid, 1.76 g). **^{1}H NMR** (400 MHz, $CDCl_3$) δ 8.26 (d, *J* = 8.2 Hz, 1 H), 7.52 (d, *J* = 8.5 Hz, 1 H), 7.45 – 7.27 (m, 6 H), 7.09 (td, *J* = 7.6, 1.1 Hz, 1 H), 6.90 – 6.79 (m, 3 H), 5.03 (s, 2 H), 4.08 (dd, *J* = 9.1, 6.0 Hz, 1 H), 3.53 (dd, *J* = 13.8, 6.0 Hz, 1 H), 3.06 (dd, *J* = 13.8, 9.1 Hz, 1 H), 2.72 (s, 3 H) ppm. **^{13}C {H} NMR** (100 MHz, $CDCl_3$) δ 177.6, 171.1, 158.0, 140.4, 138.0, 136.6, 133.9, 128.8, 128.6, 128.3, 127.5, 127.4, 124.9, 124.5, 118.6, 116.6, 115.8, 115.6, 70.4, 45.5, 38.6, 26.8 ppm. HRMS (ESI) calculated for $[M+H]^+$ $C_{24}H_{21}BrNO_3^+$: 450.0699, found: 450.0692.

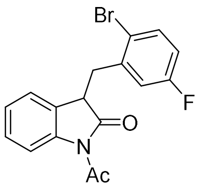

Chemical Formula: $C_{17}H_{13}BrFNO_2$
Exact Mass: 361.0114

1-acetyl-3-(2-bromo-5-fluorobenzyl)indolin-2-one (1k). Purification by column chromatography (eluent: petroleum ether/ethyl acetate = 20/1), 84% isolated yield (white solid, 1.52 g). **^{1}H NMR** (400 MHz, $CDCl_3$) δ 8.15 (d, *J* = 8.2 Hz, 1 H), 7.49 (dd, *J* = 8.7, 5.3 Hz, 1 H), 7.22 (t, 1 H), 7.02 (td, *J* = 7.6, 1.1 Hz, 1 H), 6.89 (dd, *J* = 9.1, 3.0 Hz, 1 H), 6.84 (td, *J* = 8.3, 3.0 Hz, 1 H), 6.77 (d, *J* = 7.5 Hz, 1 H), 4.09 – 3.81 (m, 1 H), 3.40 (dd, *J* = 13.9, 6.4 Hz, 1 H), 3.01 (dd, *J* = 13.9, 8.7 Hz, 1 H), 2.61 (s, 3 H) ppm. **^{19}F NMR** (376 MHz, $CDCl_3$) δ -114.30 ppm. **^{13}C {H} NMR** (100 MHz, $CDCl_3$) δ 177.4, 171.0, 161.8 (d, *J* = 247.3 Hz), 140.4, 139.1 (d, *J* = 7.4 Hz), 134.4 (d, *J* = 8.1 Hz), 128.8, 127.1, 125.1, 124.2, 119.058 (d, *J* = 22.8 Hz), 119.057 (d, *J* = 3.6 Hz), 116.7, 116.2 (d, *J* = 22.6 Hz), 45.3, 38.4, 26.8 ppm. HRMS (ESI) calculated for $[M+H]^+$ $C_{17}H_{14}BrFNO_2^+$: 362.0186, found: 362.0186.

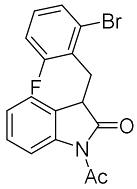

Chemical Formula: $C_{17}H_{13}BrFNO_2$
Exact Mass: 361.0114

1-acetyl-3-(2-bromo-6-fluorobenzyl)indolin-2-one (1l). Purification by column chromatography (eluent: petroleum ether/ethyl acetate = 20/1), 78% isolated yield (white solid, 1.41 g). **^{1}H NMR** (400 MHz, $CDCl_3$) δ 8.24 (d, *J* = 8.2 Hz, 1 H), 7.43 (d, *J* = 8.1 Hz, 1 H), 7.31 (t, *J* = 7.9 Hz, 1 H), 7.19 (td, *J* = 8.2, 5.9 Hz, 1 H), 7.14 – 7.04 (m, 2 H), 6.87 (d, *J* = 7.6 Hz, 1 H), 4.01 (t, *J* = 8.2 Hz, 1 H), 3.42 (dd, *J* = 13.8, 7.9 Hz, 1 H), 3.26 (dd, *J* = 13.8, 8.4 Hz, 1 H), 2.67 (s, 3 H) ppm. **^{19}F NMR** (376 MHz, $CDCl_3$) δ -110.55 ppm. **^{13}C {H} NMR** (100 MHz, $CDCl_3$) δ 177.0, 171.1, 161.7 (d, *J* = 249.8 Hz), 140.4, 129.6 (d, *J* = 9.5 Hz), 128.8 (d, *J* = 3.7 Hz), 128.7, 127.4, 125.9 (d, *J* = 4.8 Hz), 125.3 (d, *J* = 18.0 Hz), 125.1, 123.8, 116.7, 114.8 (d, *J* = 23.1 Hz), 44.4, 31.6 (d,

J = 2.2 Hz), 26.8 ppm. HRMS (ESI) calculated for $[M+H]^+$ $C_{17}H_{14}BrFNO_2^+$: 362.0186, found: 362.0187.

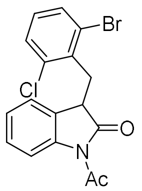

Chemical Formula: $C_{17}H_{13}BrClNO_2$
Exact Mass: 376.9818

1-acetyl-3-(2-bromo-6-chlorobenzyl)indolin-2-one (1m). Purification by column chromatography (eluent: petroleum ether/ethyl acetate = 20/1), 83% isolated yield (white solid, 1.57 g). **^{1}H NMR** (400 MHz, $CDCl_3$) δ 8.25 (d, J = 7.8 Hz, 1 H), 7.54 (dd, J = 8.0, 1.2 Hz, 1 H), 7.40 (dd, J = 8.0, 1.2 Hz, 1 H), 7.31 (t, J = 7.8 Hz, 1 H), 7.18 – 7.06 (m, 2 H), 6.84 (d, J = 7.6 Hz, 1 H), 4.13 (t, J = 8.5 Hz, 1 H), 3.56 (dd, J = 13.7, 8.4 Hz, 1 H), 3.45 (dd, J = 13.8, 8.8 Hz, 1 H), 2.67 (s, 3 H) ppm. **^{13}C {H} NMR** (100 MHz, $CDCl_3$) δ 176.9, 171.1, 140.4, 136.2, 134.9, 131.9, 129.4, 129.2, 128.6, 127.3, 126.7, 125.0, 123.9, 116.7, 43.9, 36.4, 26.8 ppm. HRMS (ESI) calculated for $[M+H]^+$ $C_{17}H_{14}BrClNO_2^+$: 377.9891, found: 377.9895.

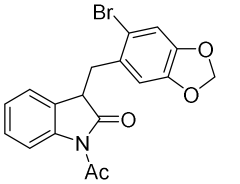

Chemical Formula: $C_{18}H_{14}BrNO_4$
Exact Mass: 387.0106

1-acetyl-3-((6-bromobenzo[d][1,3]dioxol-5-yl)methyl)indolin-2-one (1n). Recrystallization from hexane/CH_2Cl_2 to give the product, 62% isolated yield (white solid, 1.20 g). **^{1}H NMR** (400 MHz, $CDCl_3$) δ 8.21 (d, J = 8.2 Hz, 1 H), 7.30 (tt, J = 8.3, 1.1 Hz, 1 H), 7.10 (td, J = 7.6, 1.1 Hz, 1 H), 7.04 (s, 1 H), 6.90 (dd, J = 7.6, 1.4 Hz, 1 H), 6.69 (s, 1 H), 5.99 (d, J = 5.1 Hz, 2 H), 3.98 (dd, J = 8.7, 6.4 Hz, 1 H), 3.39 (dd, J = 14.0, 6.4 Hz, 1 H), 3.02 (dd, J = 14.0, 8.7 Hz, 1 H), 2.68 (s, 3 H) ppm. **^{13}C {H} NMR** (100 MHz, $CDCl_3$) δ 177.6, 171.0, 147.7, 147.4, 140.4, 129.9, 128.6, 127.4, 124.9, 124.4, 116.6, 115.3, 113.0, 111.4, 102.0, 45.8, 38.3, 26.8 ppm. HRMS (ESI) calculated for $[M+H]^+$ $C_{18}H_{14}BrNO_4^+$: 388.0179, found: 388.0180.

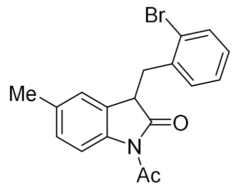

Chemical Formula: $C_{18}H_{16}BrNO_2$
Exact Mass: 357.0364

1-acetyl-3-(2-bromobenzyl)-5-methylindolin-2-one (1o). Purification by column chromatography (eluent: petroleum ether/ethyl acetate = 20/1), 91% isolated yield (white solid, 1.63 g). **^{1}H NMR** (400 MHz, $CDCl_3$) δ 8.11 (d, *J* = 8.3 Hz, 1 H), 7.64 (d, *J* = 7.8 Hz, 1 H), 7.33 – 7.28 (m, 1 H), 7.25 – 7.18 (m, 2 H), 7.11 (d, *J* = 8.3 Hz, 1 H), 6.62 (s, 1 H), 4.04 (t, *J* = 7.6 Hz, 1 H), 3.51 (dd, *J* = 13.9, 6.5 Hz, 1 H), 3.11 (dd, *J* = 13.8, 8.7 Hz, 1 H), 2.69 (s, 3 H), 2.27 (s, 3 H) ppm. **^{13}C {H} NMR** (100 MHz, $CDCl_3$) δ 177.7, 170.9, 138.0, 137.1, 134.5, 133.2, 132.3, 129.0, 128.9, 127.4, 127.4, 125.0, 125.0, 116.3, 45.4, 38.5, 26.8, 21.2 ppm. HRMS (ESI) calculated for $[M+H]^+$ $C_{18}H_{17}BrNO_2^+$: 358.0437, found: 358.0441.

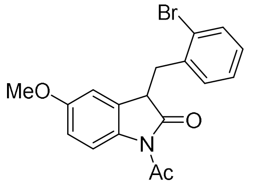

Chemical Formula: $C_{18}H_{16}BrNO_3$
Exact Mass: 373.0314

1-acetyl-3-(2-bromobenzyl)-5-methoxyindolin-2-one (1p). Recrystallization from hexane/CH_2Cl_2 to give the product, 72% isolated yield (white solid, 1.34 g). **^{1}H NMR** (400 MHz, $CDCl_3$) δ 8.14 (d, *J* = 9.0 Hz, 1 H), 7.62 (d, *J* = 7.9 Hz, 1 H), 7.29 (td, *J* = 7.3, 1.3 Hz, 1 H), 7.23 – 7.14 (m, 2 H), 6.80 (dd, *J* = 9.0, 2.8 Hz, 1 H), 6.27 (d, *J* = 2.7 Hz, 1 H), 4.04 (dd, *J* = 9.2, 6.1 Hz, 1 H), 3.66 (s, 3 H), 3.56 (dd, *J* = 13.8, 6.2 Hz, 1 H), 3.05 (dd, *J* = 13.8, 9.2 Hz, 1 H), 2.67 (s, 3 H) ppm. **^{13}C {H} NMR** (100 MHz, $CDCl_3$) δ 177.5, 170.7, 156.9, 137.0, 133.8, 133.3, 132.3, 129.1, 128.7, 127.5, 125.0, 117.5, 113.4, 110.5, 55.5, 45.6, 38.4, 26.7 ppm. HRMS (ESI) calculated for $[M+H]^+$ $C_{18}H_{17}BrNO_3^+$: 374.0386, found: 374.0388.

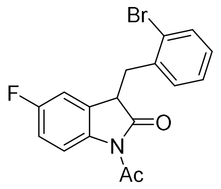

Chemical Formula: $C_{17}H_{13}BrFNO_2$
Exact Mass: 361.0114

1-acetyl-3-(2-bromobenzyl)-5-fluoroindolin-2-one (1q). Recrystallization from hexane/CH_2Cl_2 to give the product, 80% isolated yield (white solid, 1.44 g). **^{1}H NMR** (400 MHz, $CDCl_3$) δ 8.13 (dd, *J* = 9.0, 4.8 Hz, 1 H), 7.55 (dd, *J* = 8.1, 1.4 Hz, 1 H), 7.22 (td, *J* = 7.3, 1.3 Hz, 1 H), 7.19 – 7.09 (m, 2 H), 6.90 (td, *J* = 9.1, 2.8 Hz, 1 H), 6.36 (dd, *J* = 8.2, 2.5 Hz, 1 H), 3.99 (dd, *J* = 9.2, 6.1 Hz, 1 H), 3.48 (dd, *J* = 13.8, 6.1 Hz, 1 H), 2.97 (dd, *J* = 13.8, 9.2 Hz, 1 H), 2.60 (s, 3 H) ppm. **^{19}F NMR** (376 MHz, $CDCl_3$) δ -116.54 ppm. **^{13}C {H} NMR** (100 MHz, $CDCl_3$) δ 177.1, 170.8, 159.9 (d, *J* = 244.2 Hz), 136.5, 136.4, 133.4, 132.2, 129.34, 129.27, 127.7, 124.9, 117.9 (d, *J* = 7.7 Hz), 115.0 (d, *J* = 22.6 Hz), 112.0 (d, *J* = 24.7 Hz), 45.5, 38.3, 26.7 ppm. HRMS (ESI) calculated for $[M+H]^+$ $C_{17}H_{14}BrFNO_2^+$: 362.0186, found: 362.0189.

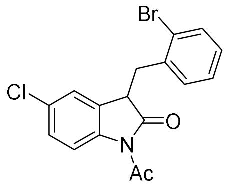

Chemical Formula: $C_{17}H_{13}BrClNO_2$
Exact Mass: 376.9818

1-acetyl-3-(2-bromobenzyl)-5-chloroindolin-2-one (1r). Recrystallization from hexane/CH_2Cl_2 to give the product, 75% isolated yield (white solid, 1.42 g). **^{1}H NMR** (400 MHz, $CDCl_3$) δ 8.18 (d, *J* = 8.8 Hz, 1 H), 7.65 (d, *J* = 8.6 Hz, 1 H), 7.36 – 7.19 (m, 4 H), 6.73 (s, 1 H), 4.07 (t, *J* = 7.7 Hz, 1 H), 3.55 (dd, *J* = 13.9, 6.4 Hz, 1 H), 3.08 (dd, *J* = 13.8, 8.9 Hz, 1 H), 2.69 (s, 3 H) ppm. **^{13}C {H} NMR** (100 MHz, $CDCl_3$) δ 176.8, 170.8, 138.8, 136.4, 133.4, 132.2, 130.3, 129.3, 129.2, 128.5, 127.6, 124.9, 124.7, 117.8, 45.2, 38.3, 26.7 ppm. HRMS (ESI) calculated for $[M+H]^+$ $C_{17}H_{14}BrClNO_2^+$: 377.9891, found: 377.9895.

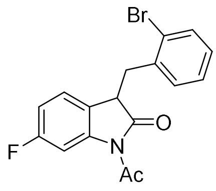

Chemical Formula: $C_{17}H_{13}BrFNO_2$
Exact Mass: 361.0114

1-acetyl-3-(2-bromobenzyl)-6-fluoroindolin-2-one (1s). Purification by column chromatography (eluent: petroleum ether/ethyl acetate = 20/1), 87% isolated yield (white solid, 1.58 g). **^{1}H NMR** (400 MHz, $CDCl_3$) δ 8.00 (dd, J = 10.3, 2.5 Hz, 1 H), 7.61 (dd, J = 8.3, 1.3 Hz, 1 H), 7.28 (td, J = 7.5, 1.3 Hz, 1 H), 7.22 – 7.14 (m, 2 H), 6.75 (td, J = 8.6, 2.5 Hz, 1 H), 6.64 (ddd, J = 8.4, 5.6, 1.1 Hz, 1 H), 4.12 – 3.95 (m, 1 H), 3.54 (dd, J = 13.8, 6.1 Hz, 1 H), 3.03 (dd, J = 13.8, 9.2 Hz, 1 H), 2.68 (s, 3 H) ppm. **^{19}F NMR** (376 MHz, $CDCl_3$) δ -110.87 ppm. **^{13}C {H} NMR** (100 MHz, $CDCl_3$) δ 177.5, 170.9, 162.6 (d, J = 244.5 Hz), 141.2 (d, J = 12.8 Hz), 136.7, 133.4, 132.2, 129.1, 127.6, 125.3 (d, J = 9.2 Hz), 124.9, 122.8 (d, J = 3.4 Hz), 111.5 (d, J = 22.7 Hz), 105.3 (d, J = 29.9 Hz), 45.0, 38.5, 26.7 ppm. HRMS (ESI) calculated for $[M+H]^+$ $C_{17}H_{14}BrFNO_2^+$: 362.0186, found: 362.0190.

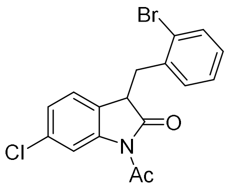

Chemical Formula: $C_{17}H_{13}BrClNO_2$
Exact Mass: 376.9818

1-acetyl-3-(2-bromobenzyl)-6-chloroindolin-2-one (1t). Purification by column chromatography (eluent: petroleum ether/ethyl acetate = 20/1), 88% isolated yield (white solid, 1.67 g). **^{1}H NMR** (400 MHz, $CDCl_3$) δ 8.29 (d, J = 2.0 Hz, 1 H), 7.63 (d, J = 8.3 Hz, 1 H), 7.33 – 7.28 (m, 1 H), 7.24 – 7.18 (m, 2 H), 7.05 (dd, J = 8.1, 2.0 Hz, 1 H), 6.64 (dd, J = 8.2, 1.2 Hz, 1 H), 4.06 (dd, J = 9.3, 6.1 Hz, 1 H), 3.56 (dd, J = 13.8, 6.1 Hz, 1 H), 3.05 (dd, J = 13.8, 9.3 Hz, 1 H), 2.70 (s, 3 H) ppm. **^{13}C {H} NMR** (100 MHz, $CDCl_3$) δ 177.1, 170.8, 141.1, 136.6, 134.3, 133.4, 132.2, 129.2, 127.6, 125.7, 125.2, 124.9, 124.9, 117.1, 45.1, 38.4, 26.7 ppm. HRMS (ESI) calculated for $[M+H]^+$ $C_{17}H_{14}BrClNO_2^+$: 377.9891, found: 377.9892.

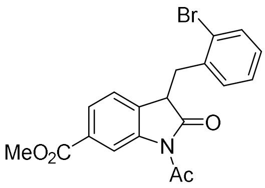

Chemical Formula: $C_{19}H_{16}BrNO_4$
Exact Mass: 401.0263

Methyl 1-acetyl-3-(2-bromobenzyl)-2-oxoindoline-6-carboxylate (1u). Recrystallization from hexane/CH_2Cl_2 to give the product, 70% isolated yield (white solid, 1.40 g). **^{1}H NMR** (400 MHz, $CDCl_3$) δ 8.84 (s, 1 H), 7.79 (dd, J = 7.9, 1.6 Hz, 1 H), 7.63 (d, J = 8.1 Hz, 1 H), 7.29 (td, J = 7.2, 1.3 Hz, 1 H), 7.23 – 7.17 (m, 2 H), 6.80 (d, J = 8.6 Hz, 1 H), 4.12 (dd, J = 9.2, 6.2 Hz, 1 H), 3.92 (s, 3 H), 3.58 (dd, J = 13.8, 6.2 Hz, 1 H), 3.09 (dd, J = 13.8, 9.2 Hz, 1 H), 2.71 (s, 3 H) ppm. **^{13}C {H} NMR** (100 MHz, $CDCl_3$) δ 176.9, 170.8, 166.6, 140.5, 136.5, 133.4, 132.3, 132.1, 130.6, 129.2, 127.6, 126.5, 124.9, 124.3, 117.3, 52.4, 45.5, 38.2, 26.8 ppm. HRMS (ESI) calculated for $[M+H]^+$ $C_{19}H_{17}BrNO_4^+$: 402.0335, found: 402.0339.

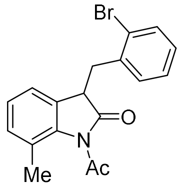

Chemical Formula: $C_{18}H_{16}BrNO_2$
Exact Mass: 357.0364

1-acetyl-3-(2-bromobenzyl)-7-methylindolin-2-one (1v). Recrystallization from hexane/CH_2Cl_2 to give the product, 75% isolated yield (white solid, 1.35 g). **^{1}H NMR** (400 MHz, $CDCl_3$) δ 7.53 (dd, J = 7.9, 1.3 Hz, 1 H), 7.20 – 7.17 (m, 1 H), 7.14 – 7.07 (m, 2 H), 7.04 (d, J = 8.2 Hz, 1 H), 6.92 (t, J = 7.6 Hz, 1 H), 6.54 (d, J = 7.4 Hz, 1 H), 3.98 (dd, J = 8.9, 6.3 Hz, 1 H), 3.43 (dd, J = 13.8, 6.2 Hz, 1 H), 3.00 (dd, J = 13.8, 8.9 Hz, 1 H), 2.63 (s, 3 H), 2.09 (s, 3 H) ppm. **^{13}C {H} NMR** (100 MHz, $CDCl_3$) δ 178.3, 170.6, 138.7, 137.1, 133.3, 132.2, 131.6, 129.0, 128.9, 127.5, 126.5, 125.1, 125.0, 121.8, 46.2, 38.6, 26.7, 21.6 ppm. HRMS (ESI) calculated for $[M+H]^+$ $C_{18}H_{17}BrNO_2^+$: 358.0437, found: 358.0440.

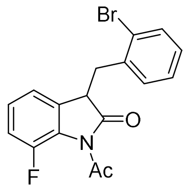

Chemical Formula: $C_{17}H_{13}BrFNO_2$
Exact Mass: 361.0114

1-acetyl-3-(2-bromobenzyl)-7-fluoroindolin-2-one (1w). Recrystallization from hexane/CH_2Cl_2 to give the product, 86% isolated yield (white solid, 1.56 g). **^{1}H NMR** (400 MHz, $CDCl_3$) δ 7.57 (d, *J* = 7.3 Hz, 1 H), 7.23 (d, *J* = 6.7 Hz, 1 H), 7.18 – 7.11 (m, 2 H), 7.06 – 6.98 (m, 2 H), 6.55 – 6.47 (m, 1 H), 4.07 (dd, *J* = 9.1, 6.2 Hz, 1 H), 3.50 (dd, *J* = 13.9, 6.2 Hz, 1 H), 3.05 (dd, *J* = 13.9, 9.0 Hz, 1 H), 2.66 (s, 3 H) ppm. **^{19}F NMR** (376 MHz, $CDCl_3$) δ -110.58 ppm. **^{13}C {H} NMR** (100 MHz, $CDCl_3$) δ 176.5, 168.8, 149.7 (d, *J* = 254.2 Hz), 136.6, 133.3, 132.2, 130.8 (d, *J* = 2.5 Hz), 129.1, 127.6, 126.4 (d, *J* = 10.0 Hz), 126.2 (d, *J* = 7.1 Hz), 124.9, 120.3 (d, *J* = 3.6 Hz), 117.2 (d, *J* = 21.4 Hz), 46.3, 38.4, 26.2 (d, *J* = 1.8 Hz) ppm. HRMS (ESI) calculated for $[M+H]^+$ $C_{17}H_{14}BrFNO_2^+$: 362.0186, found: 362.0185.

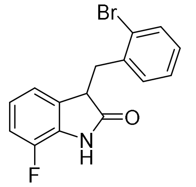

Chemical Formula: $C_{15}H_{11}BrFNO$
Exact Mass: 319.0008

3-(2-bromobenzyl)-7-fluoroindolin-2-one (1w'). Purification by column chromatography (eluent: petroleum ether/ethyl acetate = 2/1), 92% isolated yield (white solid, 2.93 g). **^{1}H NMR** (400 MHz, $CDCl_3$) δ 9.07 (s, 1 H), 7.59 (d, *J* = 7.9 Hz, 1 H), 7.25 (p, *J* = 6.4, 5.5 Hz, 2 H), 7.18 – 7.12 (m, 1 H), 6.96 (t, *J* = 9.1 Hz, 1 H), 6.81 (td, *J* = 8.0, 4.9 Hz, 1 H), 6.41 (d, *J* = 7.5 Hz, 1 H), 3.98 (dd, *J* = 9.9, 5.7 Hz, 1 H), 3.62 (dd, *J* = 13.9, 5.8 Hz, 1 H), 3.01 (dd, *J* = 13.9, 9.9 Hz, 1 H) ppm. **^{19}F NMR** (376 MHz, $CDCl_3$) δ -133.57 ppm. **^{13}C {H} NMR** (100 MHz, $CDCl_3$) δ 178.9, 147.1 (d, *J* = 243.7 Hz), 137.4, 133.2, 132.1, 131.5 (d, *J* = 3.1 Hz), 128.74 (d, *J* = 12.3 Hz), 128.72, 127.5, 125.0, 122.7 (d, *J* = 5.8 Hz), 120.8 (d, *J* = 3.5 Hz), 115.2 (d, *J* = 17.1 Hz), 45.7 (d, *J* = 2.3 Hz), 37.3 ppm. HRMS (ESI) calculated for $[M+H]^+$ $C_{15}H_{12}BrFNO^+$: 320.0081,

found: 320.0082.

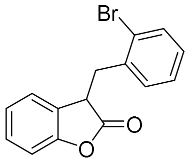

Chemical Formula: $C_{15}H_{11}BrO_2$
Exact Mass: 301.9942

3-(2-bromobenzyl)benzofuran-2(3H)-one (1x). Purification by column chromatography (eluent: petroleum ether/ethyl acetate = 5/1), 93% isolated yield in two steps (yellow solid, 2.80 g). **^{1}H NMR** (400 MHz, $CDCl_3$) δ 7.62 (d, *J* = 8.1 Hz, 1 H), 7.32 – 7.25 (m, 2 H), 7.24 – 7.16 (m, 2 H), 7.10 (d, *J* = 7.9 Hz, 1 H), 7.01 (t, *J* = 7.5 Hz, 1 H), 6.71 (d, *J* = 7.5 Hz, 1 H), 4.18 (dd, *J* = 9.5, 5.9 Hz, 1 H), 3.58 (dd, *J* = 13.8, 6.2 Hz, 1 H), 3.09 (dd, *J* = 13.8, 9.5 Hz, 1 H) ppm. **^{13}C {H} NMR** (100 MHz, $CDCl_3$) δ 176.3, 153.8, 136.5, 133.4, 132.2, 129.2, 129.1, 127.6, 126.7, 125.1, 124.9, 124.0, 110.8, 42.7, 38.1 ppm. HRMS (ESI) calculated for $[M+H]^+$ $C_{15}H_{12}BrO_2^+$: 303.0015, found: 303.0019.

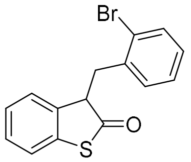

Chemical Formula: $C_{15}H_{11}BrOS$
Exact Mass: 317.9714

3-(2-bromobenzyl)benzo[b]thiophen-2(3H)-one.*[2] *(1y). Purification by column chromatography (eluent: petroleum ether/ethyl acetate = 20/1), 92% isolated yield in two steps (yellow solid, 2.93 g). **^{1}H NMR** (400 MHz, $CDCl_3$) δ 7.50 (dd, *J* = 8.0, 1.3 Hz, 1 H), 7.25 (d, *J* = 7.8 Hz, 1 H), 7.18 (dt, *J* = 7.8, 1.2 Hz, 1 H), 7.14 (dd, *J* = 7.5, 1.4 Hz, 1 H), 7.07 (dd, *J* = 7.7, 1.9 Hz, 1 H), 7.03 (dd, *J* = 7.5, 1.9 Hz, 1 H), 6.99 (td, *J* = 7.6, 1.3 Hz, 1 H), 6.67 (d, *J* = 7.6 Hz, 1 H), 4.18 – 4.11 (m, 1 H), 3.37 (dd, *J* = 13.9, 6.6 Hz, 1 H), 3.06 (dd, *J* = 13.9, 8.3 Hz, 1 H) ppm. **^{13}C {H} NMR** (100 MHz, $CDCl_3$) δ 205.0, 136.7, 136.1, 135.7, 133.2, 132.4, 128.9, 128.6, 127.4, 126.0, 125.6, 125.0, 123.1, 56.1, 39.8 ppm.

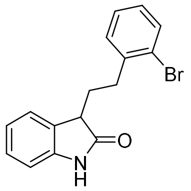

Chemical Formula: $C_{16}H_{14}BrNO$
Exact Mass: 315.0259

3-(2-bromophenethyl)indolin-2-one (1z). Purification by column chromatography (eluent: petroleum ether/ethyl acetate = 4/1), 60% isolated yield in three steps (white solid, 1.90 g). **^{1}H NMR** (400 MHz, $CDCl_3$) δ 8.91 (s, 1 H), 7.50 (d, *J* = 7.9 Hz, 1 H), 7.32 (d, *J* = 7.4 Hz, 1 H), 7.22 (dt, *J* = 15.9, 7.8 Hz, 3 H), 7.11 – 7.00 (m, 2 H), 6.94 (d, *J* = 7.7 Hz, 1 H), 3.56 (t, *J* = 5.8 Hz, 1 H), 2.99 – 2.67 (m, 2 H), 2.29 (dd, *J* = 14.2, 8.2 Hz, 2 H) ppm. **^{13}C {H} NMR** (100 MHz, $CDCl_3$) δ 180.3, 141.8, 140.8, 132.9, 130.7, 129.3, 128.2, 128.0, 127.6, 124.5, 124.4, 122.5, 110.0, 45.7, 32.5, 30.5 ppm. HRMS (ESI) calculated for $[M+H]^+$ $C_{16}H_{15}BrNO^+$: 316.0332, found: 316.0336.

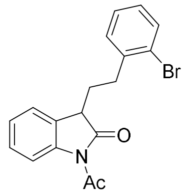

Chemical Formula: $C_{18}H_{16}BrNO_2$
Exact Mass: 357.0364

1-acetyl-3-(2-bromophenethyl)indolin-2-one (1aa). Recrystallization from hexane/CH_2Cl_2 to give the product, 89% isolated yield (white solid, 1.59 g). **^{1}H NMR** (400 MHz, $CDCl_3$) δ 8.24 (d, *J* = 8.4 Hz, 1 H), 7.51 (d, *J* = 7.9 Hz, 1 H), 7.33 (t, *J* = 6.1 Hz, 2 H), 7.25 – 7.15 (m, 2 H), 7.12 (dd, *J* = 7.5, 1.5 Hz, 1 H), 7.05 (td, *J* = 7.8, 1.7 Hz, 1 H), 3.68 (t, *J* = 5.7 Hz, 1 H), 2.89 – 2.70 (m, 2 H), 2.65 (s, 3 H), 2.55 – 2.35 (m, 1 H), 2.33 – 2.21 (m, 1 H) ppm. **^{13}C {H} NMR** (100 MHz, $CDCl_3$) δ 178.1, 171.0, 140.7, 140.1, 133.0, 130.7, 128.5, 128.2, 127.6, 127.6, 125.2, 124.5, 123.6, 116.7, 45.6, 32.4, 30.8, 26.8 ppm. HRMS (ESI) calculated for $[M+H]^+$ $C_{18}H_{17}BrNO_2^+$: 358.0437, found: 358.0439.

3 Reaction optimization

Supplementary Table 1 Evaluation of different Pd precursors for preparation of **2a**[a]

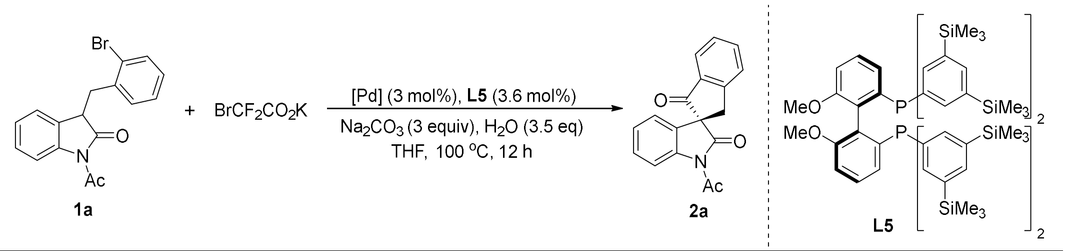

Entry	[Pd]	Yield (%)[b]	ee (%)[c]
1	$Pd(OAc)_2$	97	96
2	$Pd(CF_3CO_2)_2$	86	87
3	$PdCl_2$	95	91
4	$Pd_2(dba)_3$	90	85
5	$Pd(PPh_3)_4$	94	88
6	-	0	-

[a]Reaction condition: **1a** (0.1 mmol), $BrCF_2CO_2K$ (2.0 equiv, 0.2 mmol), [Pd] (3.0 mol%), **L5** (3.6 mol%), Na_2CO_3 (3.0 equiv, 0.3 mmol), THF (2.5 mL), H_2O (3.5 equiv, 0.35 mmol), 100 °C, 12 h. [b]Isolated yield. [c]ee was determined by HPLC analysis.

Supplementary Table 2 Evaluation of different chiral ligands[a]

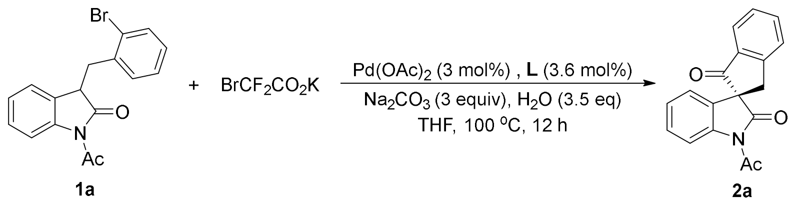

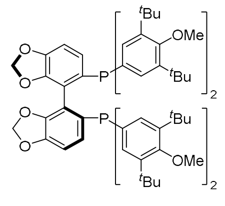

L9
94% yield, 73% ee

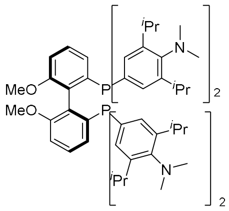

L10
92% yield, 65% ee

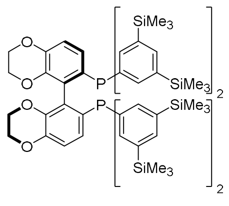

L11
89% yield, 45% ee

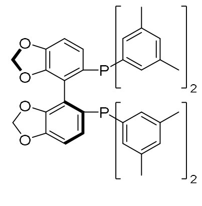

L12
93% yield, 56% ee

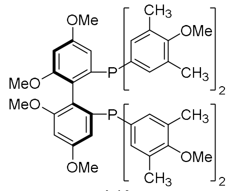

L13
87% yield, 21% ee

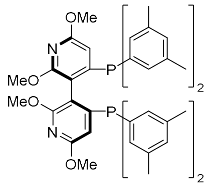

L14
78% yield, 81% ee

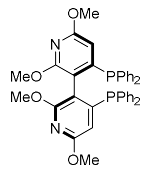

L15
90% yield, 77% ee

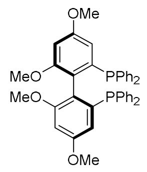

L16
92% yield, 58% ee

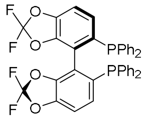

L17
76% yield, 10% ee

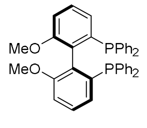

L18
92% yield, 48% ee

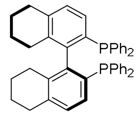

L19
88% yield, 2% ee

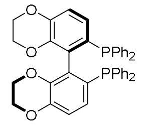

L20
86% yield, 5% ee

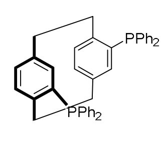

L21
94% yield, 15% ee

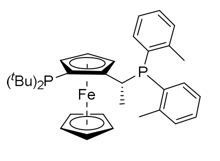

L22
81% yield, 37% ee

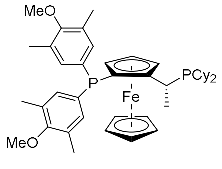

L23
78% yield, 41% ee

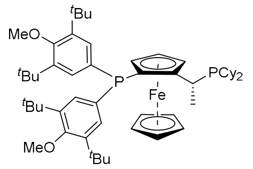

L24
83% yield, 35% ee

[a]Reaction condition: **1a** (0.1 mmol), $BrCF_2CO_2K$ (2.0 equiv, 0.2 mmol), $Pd(OAc)_2$ (3.0 mol%), **L** (3.6 mol%), Na_2CO_3 (3.0 equiv, 0.3 mmol), THF (2.5 mL), H_2O (3.5 equiv, 0.35 mmol), 100 °C, 12 h. Isolated yield. ee was determined by HPLC analysis.

Supplementary Table 3 Evaluation of different bases[a]

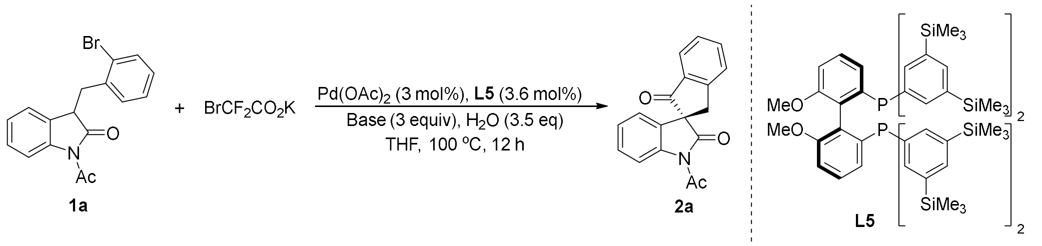

Entry	Base	Yield (%)[b]	ee (%)[c]
1	Na_2CO_3	97	96
2	K_2CO_3	95	91
3	Cs_2CO_3	95	88
4	NaOH	trace	-
5	$NaHCO_3$	trace	-
6	-	0	-

[a]Reaction condition: **1a** (0.1 mmol), $BrCF_2CO_2K$ (2.0 equiv, 0.2 mmol), $Pd(OAc)_2$ (3.0 mol%), **L5** (3.6 mol%), Base (3.0 equiv, 0.3 mmol), THF (2.5 mL), H_2O (3.5 equiv, 0.35 mmol), 100 °C,

12 h. [b]Isolated yield. [c]ee was determined by HPLC analysis.

Supplementary Table 4 Evaluation of different Na_2CO_3 supplier[a]

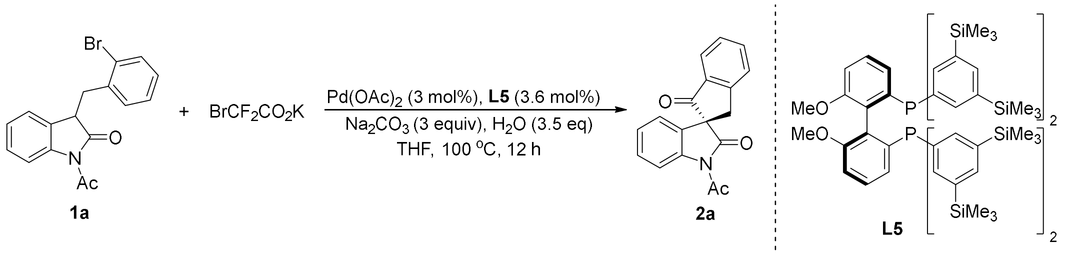

Entry	Na_2CO_3 supplier	Specification	Content (%)	Yield (%)	ee (%)
1	Adamas	2.5 kg	≥99.5	83	96
2	Bide	500 g	≥99	97	96
3	Leyan	100 g	99.95	85	92
4	J&K	5 g	≥99	95	94
5	TCI	5 g	≥99.5	90	95

[a]Reaction condition: **1a** (0.1 mmol), $BrCF_2CO_2K$ (2.0 equiv, 0.2 mmol), $Pd(OAc)_2$ (3.0 mol%), **L5** (3.6 mol%), Na_2CO_3 (3.0 equiv, 0.3 mmol), THF (2.5 mL), H_2O (3.5 equiv, 0.35 mmol), 100 °C, 12 h. [b]Isolated yield. [c]ee was determined by HPLC analysis.

Supplementary Table 5 Evaluation of solvent effect[a]

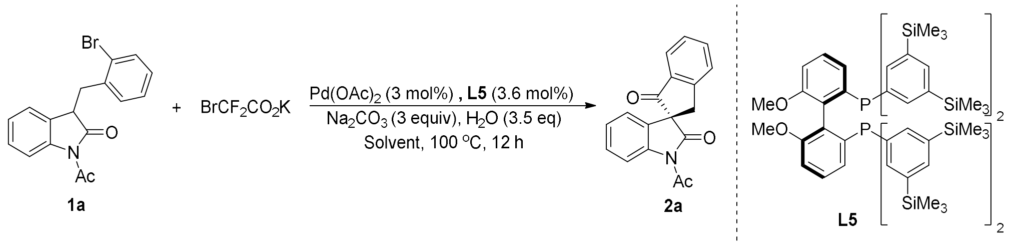

Entry	Solvent	Yield (%)[b]	ee (%)[c]
1	THF	97	96
2	toluene	75	34
3	dioxane	82	62
4	MeCN	45	0
5	benzene	57	28
6	2-MeTHF	92	72
7	glyme	83	75
8	diglyme	85	70

[a]Reaction condition: **1a** (0.1 mmol), $BrCF_2CO_2K$ (2.0 equiv, 0.2 mmol), $Pd(OAc)_2$ (3.0 mol%), **L5** (3.6 mol%), Na_2CO_3 (3.0 equiv, 0.3 mmol), solvent (2.5 mL), H_2O (3.5 equiv, 0.35 mmol), 100 °C, 12 h. [b]Isolated yield. [c]ee was determined by HPLC analysis.

Supplementary Table 6 Evaluation of different difluorocarbene precursors[a]

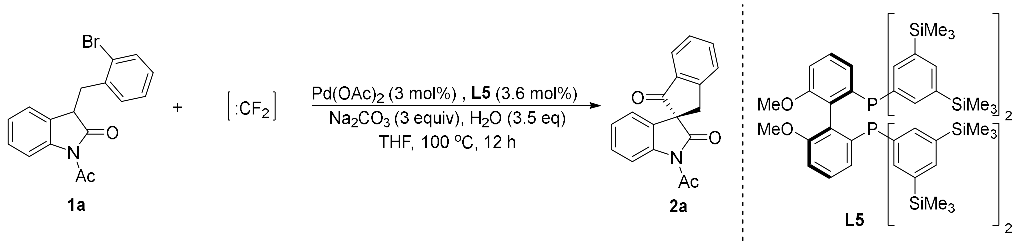

Entry	[:CF2]	Yield (%)[b]	ee (%)[c]
1	$BrCF_2CO_2K$	97	96
2	$ClCF_2CO_2Na$	95	95
3	$BrCF_2CO_2Et$	91	96
4	$ClCF_2CO_2Et$	90	94
5	$TMSCF_2Br$	96	96

Reaction condition: **1a** (0.1 mmol), [:CF_2] (2.0 equiv, 0.2 mmol), $Pd(OAc)_2$ (3.0 mol%), **L5** (3.6 mol%), Na_2CO_3 (3.0 equiv, 0.3 mmol), THF (2.5 mL), H_2O (3.5 equiv, 0.35 mmol), 100 °C, 12 h. [b] Isolated yield. [c] ee was determined by HPLC analysis.

Supplementary Table 7 Evaluation of the effect on the amount of water[a]

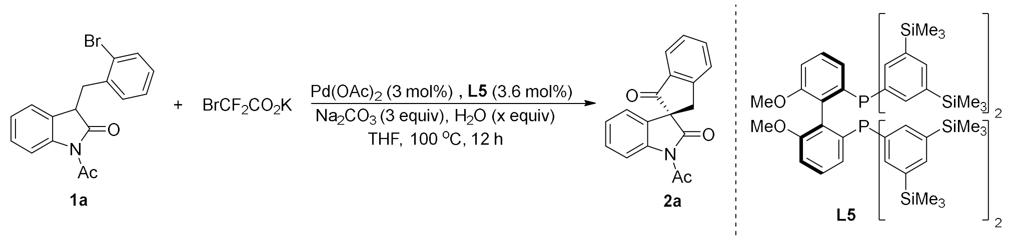

Entry	Amount of water	Yield (%)[b]	ee (%)[c]
1	0	51	96
2	0.5 equiv	78	96
3	1.0 equiv	82	96
4	2.0 equiv	90	96
5	3.0 equiv	95	96
6	3.5 equiv	97	96
7	4.0 equiv	97	93

[a]Reaction condition: **1a** (0.1 mmol), $BrCF_2CO_2K$ (2.0 equiv, 0.2 mmol), $Pd(OAc)_2$ (3.0 mol%), **L5** (3.6 mol%), Na_2CO_3 (3.0 equiv, 0.3 mmol), solvent (2.5 mL), H_2O (x equiv, x mmol), 100 °C, 12 h. [b]Isolated yield. [c]ee was determined by HPLC analysis.

Supplementary Table 8 Evaluation of temperature effect[a]

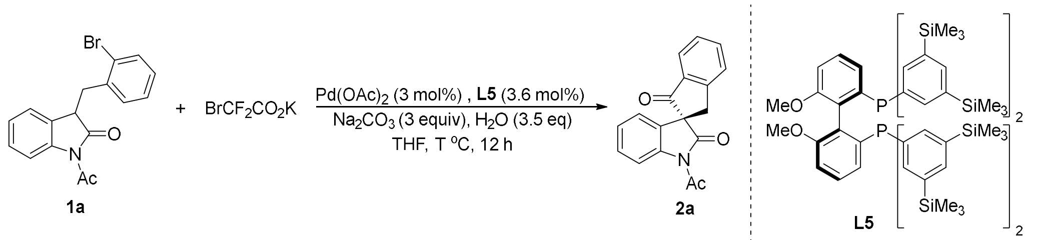

Entry	Temp (°C)	Yield (%)[b]	ee (%)[c]
1	60	trace	-
2	70	10	96
3	80	25	96
4	90	49	96
5	100	97	96
6	110	97	85

[a]Reaction condition: **1a** (0.1 mmol), $BrCF_2CO_2K$ (2.0 equiv, 0.2 mmol), $Pd(OAc)_2$ (3.0 mol%), **L5** (3.6 mol%), Na_2CO_3 (3.0 equiv, 0.3 mmol), solvent (2.5 mL), H_2O (3.5 equiv, 0.35 mmol), T °C, 12 h. [b]Isolated yield. [c]ee was determined by HPLC analysis.

Supplementary Table 9 Evaluation of reaction time[a]

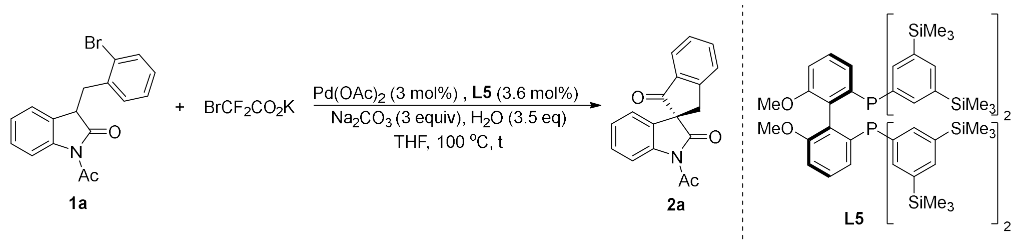

Entry	Time (h)	Yield (%)[b]	ee (%)[c]
1	8	77	96
2	10	87	96
3	12	97	96
4	24	97	96

[a]Reaction condition: **1a** (0.1 mmol), $BrCF_2CO_2K$ (2.0 equiv, 0.2 mmol), $Pd(OAc)_2$ (3.0 mol%), **L5** (3.6 mol%), Na_2CO_3 (3.0 equiv, 0.3 mmol), solvent (2.5 mL), H_2O (3.5 equiv, 0.35 mmol), 100 °C, t. [b]Isolated yield. [c]ee was determined by HPLC analysis.

Supplementary Table 10 Evaluation of *N*-substituent effect[a]

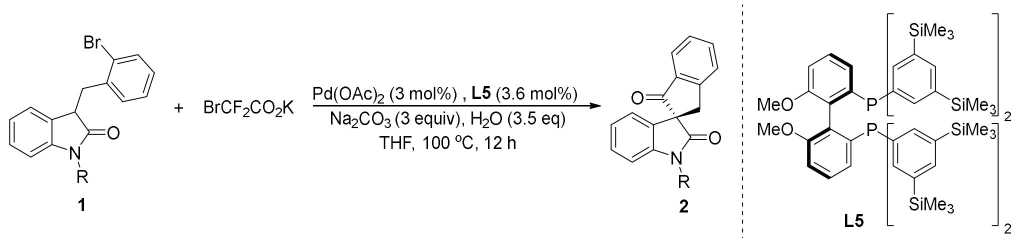

Entry	R	Yield (%)[b]	ee (%)[c]
1	H	82	48
2	Me	61	51
3	Ph	91	10
4	Bn	85	37
5	Boc	44	40
6	Ac	97	96

[a]Reaction condition: **1** (0.1 mmol), $BrCF_2CO_2K$ (2.0 equiv, 0.2 mmol), $Pd(OAc)_2$ (3.0 mol%), **L5** (3.6 mol%), Na_2CO_3 (3.0 equiv, 0.3 mmol), solvent (2.5 mL), H_2O (3.5 equiv, 0.35 mmol), 100 °C, 12 h. [b]Isolated yield. [c]ee was determined by HPLC analysis.

4. General procedures for the synthesis of chiral spirooxindoles

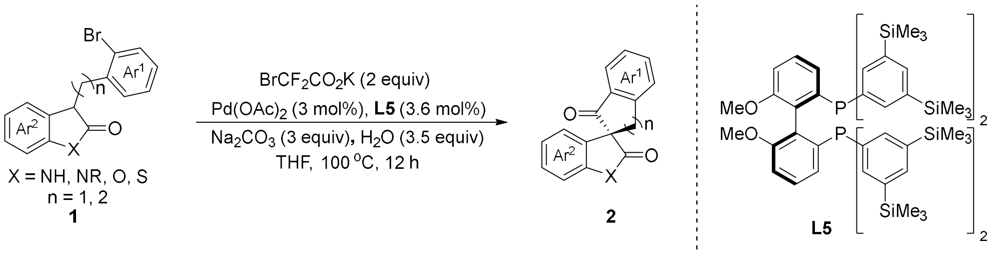

A reaction tube was charged with $Pd(OAc)_2$ (0.7 mg, 0.003 mmol, 0.03 equiv), **L5** (4.2 mg, 0.0036 mmol, 1.2 equiv to [Pd]), **1** (0.1 mmol, 1.0 equiv), $BrCF_2CO_2K$ (43 mg, 0.2 mmol, 2.0 equiv), Na_2CO_3 (31.8 mg, 0.3 mmol, 3.0 equiv), degassed H_2O (0.35 mmol, 3.5 equiv) and THF (2.5 mL) under N_2 atmosphere. The reaction vessel was sealed using a PTFE septum, and the mixture was stirred at 100 °C for 12 h. After completion of the reaction, the resulting solution was cooled to room temperature; then it was diluted with DCM (6 mL), washed with water (6 mL), extracted with DCM (6×3 mL), and dried over anhydrous Na_2SO_4 and concentrated in vacuo. The residue was purified by flash chromatography on silica gel to afford the desired product **2**.

Characterization data of 2a-2aa

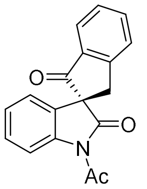

Chemical Formula: $C_{18}H_{13}NO_3$
Exact Mass: 291.0895

(R)-1'-acetylspiro[indene-2,3'-indoline]-1,2'(3H)-dione (2a). Purification by column chromatography (0.1 mmol scale, eluent: ethyl acetate/hexane = 1/6), 97% isolated yield (white solid, 28.3 mg), 96% ee, $[\alpha]^{25}D$ = +18.8 (*c* 1.0, $CHCl_3$). **^{1}H NMR** (400 MHz, $CDCl_3$) δ 8.31 (d, *J* = 6.2 Hz, 1 H), 7.82 (d, *J* = 5.8 Hz, 1 H), 7.73 (td, *J* = 5.6, 0.9 Hz, 1 H), 7.63 (d, *J* = 5.8 Hz, 1 H), 7.49 (t, *J* = 5.6 Hz, 1 H), 7.36 (td, *J* = 5.9, 1.0 Hz, 1 H), 7.14 (td, *J* = 5.7, 0.8 Hz, 1 H), 6.92 (dd, *J* = 5.6, 1.0 Hz, 1 H), 3.88 (ab, *J* = 12.9 Hz, 1 H), 3.50 (ab, *J* = 13.0 Hz, 1 H), 2.67 (s, 3 H) ppm. **^{13}C {H} NMR** (100 MHz, $CDCl_3$) δ 198.8, 176.0, 170.7, 153.6, 141.3, 136.2, 134.3, 129.4, 129.1, 128.7, 126.7, 126.0, 125.7, 121.8, 117.2, 63.8, 38.7, 26.7 ppm. HRMS (ESI) calculated for $[M+H]^+$

$C_{18}H_{14}NO_3^+$: 292.0968, found: 292.0971. **HPLC analysis:** The enantiomeric excess was determined by HPLC on Daicel Chiralpak MD (2) Column (hexane/isopropanol = 90/10, flow rate = 1.0 mL/min, T = 25 °C, UV detection at λ = 254 nm); retention time: 6.170 min (major), 7.946 min (minor).

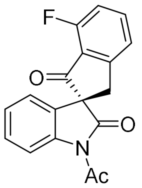

Chemical Formula: $C_{18}H_{12}FNO_3$
Exact Mass: 309.0801

(R)-1'-acetyl-7-fluorospiro[indene-2,3'-indoline]-1,2'(3H)-dione (2b). Purification by column chromatography (0.1 mmol scale, eluent: ethyl acetate/hexane = 1/10), 94% isolated yield (white solid, 28.9 mg), 92% ee, $[\alpha]^{25}D$ = +23.4 (*c* 1.0, $CHCl_3$). **^{1}H NMR** (400 MHz, $CDCl_3$) δ 8.30 (d, *J* = 8.3 Hz, 1 H), 7.71 (td, *J* = 7.9, 5.0 Hz, 1 H), 7.43 – 7.35 (m, 2 H), 7.17 (t, *J* = 7.6 Hz, 1 H), 7.11 (t, *J* = 8.6 Hz, 1 H), 6.96 (d, *J* = 7.5 Hz, 1 H), 3.88 (ab, *J* = 17.5 Hz, 1 H), 3.49 (ab, *J* = 18.4 Hz, 1 H), 2.67 (s, 3 H) ppm. **^{19}F NMR** (376 MHz, $CDCl_3$) δ -111.63 ppm. **^{13}C {H} NMR** (100 MHz, $CDCl_3$) δ 194.7, 175.6, 170.6, 160.2 (d, *J* = 266.9 Hz), 155.2 (d, *J* = 2.0 Hz), 141.4, 138.2 (d, *J* = 8.5 Hz), 129.6, 128.6, 125.8, 122.5, 122.4 (d, *J* = 4.4 Hz), 121.9, 117.3, 115.6 (d, *J* = 18.7 Hz), 64.2, 38.4 (d, *J* = 1.5 Hz), 26.7 ppm. HRMS (ESI) calculated for $[M+H]^+$ $C_{18}H_{13}FNO_3^+$: 310.0874, found: 310.0877. **HPLC analysis:** The enantiomeric excess was determined by HPLC on Daicel Chiralpak MD (2) Column (hexane/isopropanol = 90/10, flow rate = 1.0 mL/min, T = 25 °C, UV detection at λ = 254 nm); retention time: 9.006 min (major), 10.692 min (minor).

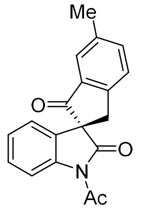

Chemical Formula: $C_{19}H_{15}NO_3$
Exact Mass: 305.1052

(R)-1'-acetyl-6-methylspiro[indene-2,3'-indoline]-1,2'(3H)-dione (2c). Purification by column chromatography (0.1 mmol scale, eluent: ethyl acetate/hexane = 1/10), 96%

isolated yield (white solid, 29.2 mg), 92% ee, $[\alpha]^{25}D$ = +24.9 (*c* 1.0, $CHCl_3$). **^{1}H NMR** (400 MHz, $CDCl_3$) δ 8.30 (d, *J* = 8.2 Hz, 1 H), 7.61 (s, 1 H), 7.57 – 7.49 (m, 2 H), 7.35 (td, *J* = 7.9, 1.4 Hz, 1 H), 7.14 (td, *J* = 7.6, 1.0 Hz, 1 H), 6.91 (d, *J* = 7.6 Hz, 1 H), 3.83 (ab, *J* = 17.1 Hz, 1 H), 3.44 (ab, *J* = 17.2 Hz, 1 H), 2.67 (s, 3 H), 2.45 (s, 3 H) ppm. **^{13}C {H} NMR** (100 MHz, $CDCl_3$) δ 198.8, 176.1, 170.7, 151.0, 141.3, 138.9, 137.5, 134.6, 129.3, 126.30, 126.29, 125.8, 125.7, 121.8, 117.2, 64.2, 38.4, 26.7, 21.2 ppm. HRMS (ESI) calculated for $[M+H]^+$ $C_{19}H_{16}NO_3^+$: 306.1125, found: 306.1126. **HPLC analysis:** The enantiomeric excess was determined by HPLC on Daicel Chiralpak MD (2) Column (hexane/isopropanol = 90/10, flow rate = 1.0 mL/min, T = 25 °C, UV detection at λ = 254 nm); retention time: 7.014 min (major), 9.538 min (minor).

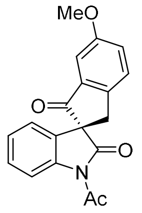

Chemical Formula: $C_{19}H_{15}NO_4$
Exact Mass: 321.1001

(R)-1'-acetyl-6-methoxyspiro[indene-2,3'-indoline]-1,2'(3H)-dione (2d). Purification by column chromatography (0.1 mmol scale, eluent: ethyl acetate/hexane = 1/8), 94% isolated yield (white solid, 30.1 mg), 93% ee, $[\alpha]^{25}D$ = +22.0 (*c* 1.0, $CHCl_3$). **^{1}H NMR** (400 MHz, $CDCl_3$) δ 8.31 (d, *J* = 7.4 Hz, 1 H), 7.51 (d, *J* = 8.4 Hz, 1 H), 7.41 – 7.30 (m, 2 H), 7.22 (d, *J* = 2.6 Hz, 1 H), 7.15 (tt, *J* = 7.6, 0.9 Hz, 1 H), 6.93 (d, *J* = 8.1 Hz, 1 H), 3.86 (s, 3 H), 3.79 (ab, *J* = 16.9 Hz, 1 H), 3.41 (ab, *J* = 16.9 Hz, 1 H), 2.68 (s, 3 H) ppm. **^{13}C {H} NMR** (100 MHz, $CDCl_3$) δ 198.7, 175.9, 170.7, 160.4, 146.6, 141.3, 135.6, 129.4, 129.2, 127.3, 125.8, 125.7, 121.8, 117.2, 106.7, 64.6, 55.9, 38.2, 26.7 ppm. HRMS (ESI) calculated for $[M+H]^+$ $C_{19}H_{16}NO_4^+$: 322.1074, found: 322.1074. **HPLC analysis:** The enantiomeric excess was determined by HPLC on Daicel Chiralpak MD (2) Column (hexane/isopropanol = 90/10, flow rate = 0.8 mL/min, T = 25 °C, UV detection at λ = 254 nm); retention time: 9.337 min (major), 10.580 min (minor).

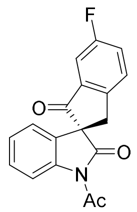

Chemical Formula: $C_{18}H_{12}FNO_3$
Exact Mass: 309.0801

(R)-1'-acetyl-6-fluorospiro[indene-2,3'-indoline]-1,2'(3H)-dione (2e). Purification by column chromatography (0.1 mmol scale, eluent: ethyl acetate/hexane = 1/10), 95% isolated yield (white solid, 29.4 mg), 94% ee, $[\alpha]^{25}D = +23.4$ (*c* 1.0, $CHCl_3$). **^{1}H NMR** (400 MHz, $CDCl_3$) δ 8.30 (d, *J* = 8.2 Hz, 1 H), 7.60 (dd, *J* = 9.2, 4.5 Hz, 1 H), 7.50 – 7.42 (m, 2 H), 7.37 (td, *J* = 7.8, 1.3 Hz, 1 H), 7.16 (t, *J* = 7.6 Hz, 1 H), 6.92 (d, *J* = 5.9 Hz, 1 H), 3.84 (ab, *J* = 17.1 Hz, 1 H), 3.46 (ab, *J* = 17.1 Hz, 1 H), 2.67 (s, 3 H) ppm. **^{19}F NMR** (376 MHz, $CDCl_3$) δ -112.59 ppm. **^{13}C {H} NMR** (100 MHz, $CDCl_3$) δ 197.8 (d, *J* = 2.9 Hz), 175.5, 170.6, 163.0 (d, *J* = 250.2 Hz), 149.0 (d, *J* = 2.2 Hz), 141.3, 136.2 (d, *J* = 7.7 Hz), 129.6, 128.7, 128.0 (d, *J* = 8.1 Hz), 125.8, 124.0 (d, *J* = 23.8 Hz), 121.8, 117.3, 111.6 (d, *J* = 22.4 Hz), 64.6, 38.1, 26.7 ppm. HRMS (ESI) calculated for $[M+H]^+$ $C_{18}H_{13}FNO_3^+$: 310.0874, found: 310.0881. **HPLC analysis:** The enantiomeric excess was determined by HPLC on Daicel Chiralpak IBN Column (hexane/isopropanol = 90/10, flow rate = 1.0 mL/min, T = 25 °C, UV detection at λ = 254 nm); retention time: 6.303 min (major), 6.934 min (minor).

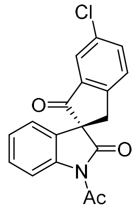

Chemical Formula: $C_{18}H_{12}ClNO_3$
Exact Mass: 325.0506

(R)-1'-acetyl-6-chlorospiro[indene-2,3'-indoline]-1,2'(3H)-dione (2f). Purification by column chromatography (0.1 mmol scale, eluent: ethyl acetate/hexane = 1/8), 94% isolated yield (white solid, 30.4 mg), 94% ee, $[\alpha]^{25}D = +26.8$ (*c* 1.0, $CHCl_3$). **^{1}H NMR** (400 MHz, $CDCl_3$) δ 8.30 (d, *J* = 8.2 Hz, 1 H), 7.78 (d, *J* = 2.0 Hz, 1 H), 7.69 (dd, *J* = 8.2, 2.1 Hz, 1 H), 7.58 (d, *J* = 9.1 Hz, 1 H), 7.37 (td, *J* = 7.9, 1.4 Hz, 1 H), 7.16 (td, *J* = 7.6, 1.1 Hz, 1 H), 6.91 (d, *J* = 7.4 Hz, 1 H), 3.84 (ab, *J* = 17.4 Hz, 1 H), 3.46 (ab, *J* =

17.4 Hz, 1 H), 2.67 (s, 3 H) ppm. **^{13}C {H} NMR** (100 MHz, $CDCl_3$) δ 197.5, 175.5, 170.6, 151.6, 141.3, 136.2, 135.9, 135.2, 129.6, 128.6, 127.8, 125.8, 125.5, 121.8, 117.3, 64.3, 38.2, 26.7 ppm. HRMS (ESI) calculated for $[M+H]^+$ $C_{18}H_{13}ClNO_3^+$: 326.0578, found: 326.0589. **HPLC analysis:** The enantiomeric excess was determined by HPLC on Daicel Chiralpak AD-H Column (hexane/isopropanol = 90/10, flow rate = 1.0 mL/min, T = 25 °C, UV detection at λ = 254 nm); retention time: 16.288 min (major), 19.680 min (minor).

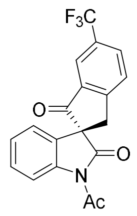

Chemical Formula: $C_{19}H_{12}F_3NO_3$
Exact Mass: 359.0769

(R)-1'-acetyl-6-(trifluoromethyl)spiro[indene-2,3'-indoline]-1,2'(3H)-dione (2g). Purification by column chromatography (0.1 mmol scale, eluent: ethyl acetate/hexane = 1/10), 93% isolated yield (white solid, 33.5 mg), 92% ee, $[\alpha]^{25}D$ = +13.2 (*c* 1.0, $CHCl_3$). **^{1}H NMR** (400 MHz, $CDCl_3$) δ 8.31 (d, *J* = 8.2 Hz, 1 H), 8.09 (s, 1 H), 7.98 (dd, *J* = 8.5, 1.8 Hz, 1 H), 7.78 (d, *J* = 8.1 Hz, 1 H), 7.39 (ddd, *J* = 8.2, 7.6, 1.4 Hz, 1 H), 7.17 (td, *J* = 7.6, 1.1 Hz, 1 H), 6.92 (d, *J* = 7.6 Hz, 1 H), 3.94 (ab, *J* = 18.9 Hz, 1 H), 3.56 (ab, *J* = 18.2 Hz, 1 H), 2.67 (s, 3 H) ppm. **^{19}F NMR** (376 MHz, $CDCl_3$) δ -62.59 ppm. **^{13}C {H} NMR** (100 MHz, $CDCl_3$) δ 197.6, 175.3, 170.5, 156.6, 141.3, 134.9, 132.6 (q, *J* = 4.0 Hz), 131.7 (q, *J* = 33.4 Hz), 129.7, 128.4, 127.5, 125.9, 123.6 (q, *J* = 271.3 Hz), 123.1 (q, *J* = 4.0 Hz), 121.8, 117.4, 64.0, 38.6, 26.7 ppm. HRMS (ESI) calculated for $[M+H]^+$ $C_{19}H_{13}F3NO_3^+$: 360.0842, found: 310.0847. **HPLC analysis:** The enantiomeric excess was determined by HPLC on Daicel Chiralpak MD (2) Column (hexane/isopropanol = 95/5, flow rate = 0.5 mL/min, T = 25 °C, UV detection at λ = 254 nm); retention time: 15.268 min (major), 18.060 min (minor).

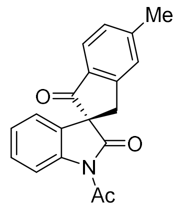

Chemical Formula: $C_{19}H_{15}NO_3$
Exact Mass: 305.1052

(R)-1'-acetyl-5-methylspiro[indene-2,3'-indoline]-1,2'(3H)-dione (2h). Purification by column chromatography (0.1 mmol scale, eluent: ethyl acetate/hexane = 1/10), 95% isolated yield (white solid, 29.1 mg), 92% ee, $[\alpha]^{25}D$ = +14.7 (*c* 1.0, $CHCl_3$). **^{1}H NMR** (400 MHz, $CDCl_3$) δ 8.30 (d, *J* = 8.2 Hz, 1 H), 7.71 (d, *J* = 7.9 Hz, 1 H), 7.42 (s, 1 H), 7.35 (td, *J* = 8.3, 1.3 Hz, 1 H), 7.30 (d, *J* = 7.9 Hz, 1 H), 7.14 (td, *J* = 7.6, 1.1 Hz, 1 H), 6.92 (dd, *J* = 7.5, 1.5 Hz, 1 H), 3.82 (ab, *J* = 17.2 Hz, 1 H), 3.44 (ab, *J* = 17.2 Hz, 1 H), 2.67 (s, 3 H), 2.52 (s, 3 H) ppm. **^{13}C {H} NMR** (100 MHz, $CDCl_3$) δ 198.1, 176.1, 170.7, 154.1, 147.8, 141.3, 132.1, 130.0, 129.32, 129.27, 127.0, 125.75, 125.68, 121.7, 117.2, 64.0, 38.5, 26.7, 22.4 ppm. HRMS (ESI) calculated for $[M+H]^+$ $C_{19}H_{16}NO_3^+$: 306.1125, found: 306.1126. **HPLC analysis:** The enantiomeric excess was determined by HPLC on Daicel Chiralpak MD (2) Column (hexane/isopropanol = 99/1, flow rate = 0.5 mL/min, T = 25 °C, UV detection at λ = 254 nm); retention time: 25.053 min (major), 29.135 min (minor).

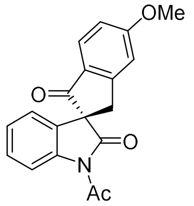

Chemical Formula: $C_{19}H_{15}NO_4$
Exact Mass: 321.1001

(R)-1'-acetyl-5-methoxyspiro[indene-2,3'-indoline]-1,2'(3H)-dione (2i). Purification by column chromatography (0.1 mmol scale, eluent: ethyl acetate/hexane = 1/8), 94% isolated yield (white solid, 30.1 mg), 86% ee, $[\alpha]^{25}D$ = +15.3 (*c* 1.0, $CHCl_3$). **^{1}H NMR** (400 MHz, $CDCl_3$) δ 8.30 (d, *J* = 8.2 Hz, 1 H), 7.74 (d, *J* = 8.4 Hz, 1 H), 7.35 (t, *J* = 7.9 Hz, 1 H), 7.15 (t, *J* = 7.6 Hz, 1 H), 7.06 – 7.00 (m, 2 H), 6.94 (d, *J* = 7.6 Hz, 1 H), 3.95 (s, 3 H), 3.81 (ab, *J* = 17.5 Hz, 1 H), 3.43 (ab, *J* = 17.2 Hz, 1 H), 2.67 (s, 3 H) ppm. **^{13}C {H} NMR** (100 MHz, $CDCl_3$) δ 196.7, 176.3, 170.7, 166.5, 156.7, 141.3, 129.4, 129.3,

127.6, 127.4, 125.7, 121.8, 117.2, 116.9, 109.8, 64.1, 56.0, 38.6, 26.7 ppm. HRMS (ESI) calculated for $[M+H]^+$ $C_{19}H_{16}NO_4^+$: 322.1074, found: 322.1076. **HPLC analysis:** The enantiomeric excess was determined by HPLC on Daicel Chiralpak MD (2) Column (hexane/isopropanol = 90/10, flow rate = 1.0 mL/min, T = 25 °C, UV detection at λ = 254 nm); retention time: 12.104 min (major), 14.116 min (minor).

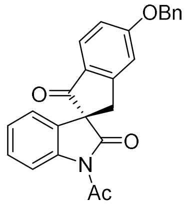

Chemical Formula: $C_{25}H_{19}NO_4$
Exact Mass: 397.1314

(R)-1'-acetyl-5-(benzyloxy)spiro[indene-2,3'-indoline]-1,2'(3H)-dione (2j). Purification by column chromatography (0.1 mmol scale, eluent: ethyl acetate/hexane = 1/8), 92% isolated yield (white solid, 36.7 mg), 93% ee, $[\alpha]^{25}D$ = +17.1 (*c* 1.0, $CHCl_3$). **^{1}H NMR** (400 MHz, $CDCl_3$) δ 8.30 (d, *J* = 8.2 Hz, 1 H), 7.75 (d, *J* = 8.6 Hz, 1 H), 7.49 – 7.33 (m, 6 H), 7.19 – 7.06 (m, 3 H), 6.94 (d, *J* = 6.7 Hz, 1 H), 5.21 (s, 2 H), 3.81 (ab, *J* = 17.4 Hz, 1 H), 3.42 (ab, *J* = 17.4 Hz, 1 H), 2.67 (s, 3 H) ppm. **^{13}C {H} NMR** (100 MHz, $CDCl_3$) δ 196.6, 176.2, 170.7, 165.7, 156.6, 141.3, 135.8, 129.4, 129.3, 129.0, 128.6, 127.7, 127.6, 127.5, 125.7, 121.8, 117.4, 117.2, 110.8, 70.8, 64.1, 38.6, 26.7 ppm. HRMS (ESI) calculated for $[M+H]^+$ $C_{25}H_{20}NO_4^+$: 398.1387, found: 398.1393. **HPLC analysis:** The enantiomeric excess was determined by HPLC on Daicel Chiralpak MD (2) Column (hexane/isopropanol = 70/30, flow rate = 1.0 mL/min, T = 25 °C, UV detection at λ = 254 nm); retention time: 10.509 min (major), 13.857 min (minor).

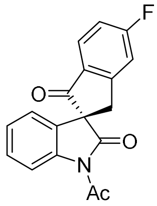

Chemical Formula: $C_{18}H_{12}FNO_3$
Exact Mass: 309.0801

(R)-1'-acetyl-5-fluorospiro[indene-2,3'-indoline]-1,2'(3H)-dione (2k). Purification by column chromatography (0.1 mmol scale, eluent: ethyl acetate/hexane = 1/10), 93% isolated yield (white solid, 28.6 mg), 92% ee, $[\alpha]^{25}D$ = +18.0 (*c* 1.0, $CHCl_3$). **^{1}H NMR**

(400 MHz, $CDCl_3$) δ 8.30 (d, *J* = 8.3 Hz, 1 H), 7.83 (dd, *J* = 8.6, 5.3 Hz, 1 H), 7.37 (t, *J* = 7.9 Hz, 1 H), 7.30 (d, *J* = 8.3 Hz, 1 H), 7.24 – 7.13 (m, 2 H), 6.94 (d, *J* = 1.3 Hz, 1 H), 3.86 (ab, *J* = 17.5 Hz, 1 H), 3.48 (ab, *J* = 17.6 Hz, 1 H), 2.67 (s, 3 H) ppm. **^{19}F NMR** (376 MHz, $CDCl_3$) δ -99.87 ppm. **^{13}C {H} NMR** (100 MHz, $CDCl_3$) δ 196.8, 175.7, 170.6, 168.0 (d, *J* = 259.3 Hz), 156.5 (d, *J* = 10.6 Hz), 141.3, 130.7, 129.5, 128.8, 128.3 (d, *J* = 11.0 Hz), 125.8, 121.8, 117.3, 117.1, 113.5 (d, *J* = 22.7 Hz), 64.0, 38.4 (d, *J* = 2.2 Hz), 26.7 ppm. HRMS (ESI) calculated for $[M+H]^+$ $C_{18}H_{13}FNO_3^+$: 310.0874, found: 310.0877. **HPLC analysis:** The enantiomeric excess was determined by HPLC on Daicel Chiralpak AD-H Column (hexane/isopropanol = 90/10, flow rate = 1.0 mL/min, T = 25 °C, UV detection at λ = 254 nm); retention time: 20.996 min (minor), 25.594 min (major).

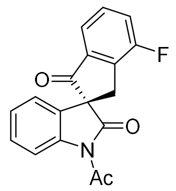

Chemical Formula: $C_{18}H_{12}FNO_3$
Exact Mass: 309.0801

(R)-1'-acetyl-4-fluorospiro[indene-2,3'-indoline]-1,2'(3H)-dione (2l). Purification by column chromatography (0.1 mmol scale, eluent: ethyl acetate/hexane = 1/10), 90% isolated yield (white solid, 27.9 mg), 91% ee, $[\alpha]^{25}D$ = +19.3 (*c* 1.0, $CHCl_3$). **^{1}H NMR** (400 MHz, $CDCl_3$) δ 8.31 (d, *J* = 8.3 Hz, 1 H), 7.64 (d, *J* = 7.6 Hz, 1 H), 7.54 – 7.47 (m, 1 H), 7.46 – 7.35 (m, 2 H), 7.17 (t, *J* = 7.6 Hz, 1 H), 6.94 (d, *J* = 7.5 Hz, 1 H), 3.88 (ab, *J* = 17.6 Hz, 1 H), 3.51 (ab, *J* = 17.6 Hz, 1 H), 2.68 (s, 3 H) ppm. **^{19}F NMR** (376 MHz, $CDCl_3$) δ -117.87 ppm. **^{13}C {H} NMR** (100 MHz, $CDCl_3$) δ 197.6, 175.5, 170.6, 159.9 (d, *J* = 251.6 Hz), 141.3, 139.4 (d, *J* = 19.8 Hz), 137.1 (d, *J* = 4.8 Hz), 130.8 (d, *J* = 6.6 Hz), 129.7, 128.6, 125.9, 122.3 (d, *J* = 19.4 Hz), 121.8, 121.7 (d, *J* = 4.0 Hz), 117.4, 63.6, 34.5, 26.7 ppm. HRMS (ESI) calculated for $[M+H]^+$ $C_{18}H_{13}FNO_3^+$: 310.0874, found: 310.0878. **HPLC analysis:** The enantiomeric excess was determined by HPLC on Daicel Chiralpak MD (2) Column (hexane/isopropanol = 90/10, flow rate = 1.0 mL/min, T = 25 °C, UV detection at λ = 254 nm); retention time: 7.236 min (major), 8.132 min (minor).

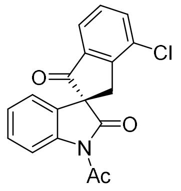

Chemical Formula: $C_{18}H_{12}ClNO_3$
Exact Mass: 325.0506

(R)-1'-acetyl-4-chlorospiro[indene-2,3'-indoline]-1,2'(3H)-dione (2m). Purification by column chromatography (0.1 mmol scale, eluent: ethyl acetate/hexane = 1/8), 93% isolated yield (white solid, 30.1 mg), 92% ee, $[\alpha]^{25}D$ = +12.8 (*c* 1.0, $CHCl_3$). **^{1}H NMR** (400 MHz, $CDCl_3$) δ 8.31 (d, *J* = 8.2 Hz, 1 H), 7.73 (d, *J* = 7.8 Hz, 2 H), 7.47 (t, *J* = 7.7 Hz, 1 H), 7.38 (td, *J* = 7.8, 1.3 Hz, 1 H), 7.17 (td, *J* = 7.6, 1.1 Hz, 1 H), 6.94 (d, *J* = 7.5 Hz, 1 H), 3.86 (ab, *J* = 18.0 Hz, 1 H), 3.49 (ab, *J* = 17.9 Hz, 1 H), 2.68 (s, 3 H) ppm. **^{13}C {H} NMR** (100 MHz, $CDCl_3$) δ 197.9, 175.5, 170.6, 151.2, 141.3, 136.2, 135.8, 133.0, 130.3, 129.6, 128.6, 125.8, 124.1, 121.9, 117.3, 63.7, 37.7, 26.7 ppm. HRMS (ESI) calculated for $[M+H]^+$ $C_{18}H_{13}ClNO_3^+$: 326.0578, found: 326.0586. **HPLC analysis:** The enantiomeric excess was determined by HPLC on Daicel Chiralpak MD (2) Column (hexane/isopropanol = 95/5, flow rate = 1.0 mL/min, T = 25 °C, UV detection at λ = 254 nm); retention time: 15.655 min (major), 17.564 min (minor).

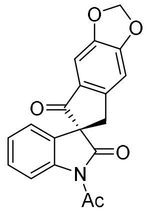

Chemical Formula: $C_{19}H_{13}NO_5$
Exact Mass: 335.0794

(R)-1'-acetylspiro[indeno[5,6-d][1,3]dioxole-6,3'-indoline]-2',5(7H)-dione (2n). Purification by column chromatography (0.1 mmol scale, eluent: ethyl acetate/hexane = 1/2), 86% isolated yield (white solid, 28.8 mg), 93% ee, $[\alpha]^{25}D$ = +15.6 (*c* 1.0, $CHCl_3$). **^{1}H NMR** (400 MHz, DMSO-d_6) δ 8.15 (d, *J* = 8.2 Hz, 1 H), 7.40 (td, *J* = 7.8, 1.5 Hz, 1 H), 7.29 (s, 1 H), 7.21 (td, *J* = 7.5, 1.1 Hz, 1 H), 7.15 – 7.12 (m, 2 H), 6.26 (s, 2 H),3.66 (d, *J* = 18.1 Hz, 1 H), 3.43 (d, *J* = 17.7 Hz, 1 H), 2.56 (s, 3 H) ppm. **^{13}C {H} NMR** (100 MHz, DMSO-d_6) δ 196.7, 175.4, 170.4, 155.5, 152.8, 149.0, 140.9, 129.3, 128.8, 127.8, 125.4, 122.4, 115.9, 106.0, 103.1, 102.5, 63.9, 38.0, 26.2 ppm. HRMS (ESI) calculated

for $[M+H]^+$ $C_{19}H_{14}NO_5^+$: 336.0866, found: 336.0870. **HPLC analysis:** The enantiomeric excess was determined by HPLC on Daicel Chiralpak MD (2) Column (hexane/isopropanol = 90/10, flow rate = 1.0 mL/min, T = 25 °C, UV detection at λ = 254 nm); retention time: 10.906 min (major), 13.290 min (minor).

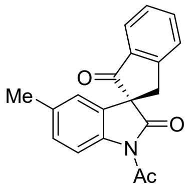

Chemical Formula: $C_{19}H_{15}NO_3$
Exact Mass: 305.1052

(R)-1'-acetyl-5'-methylspiro[indene-2,3'-indoline]-1,2'(3H)-dione (2o). Purification by column chromatography (0.1 mmol scale, eluent: ethyl acetate/hexane = 1/10), 93% isolated yield (white solid, 28.3 mg), 91% ee, $[\alpha]^{25}D$ = +28.1 (*c* 1.0, $CHCl_3$). **^{1}H NMR** (400 MHz, $CDCl_3$) δ 8.18 (d, *J* = 8.3 Hz, 1 H), 7.82 (d, *J* = 7.8 Hz, 1 H), 7.74 (t, *J* = 7.5 Hz, 1 H), 7.63 (d, *J* = 7.7 Hz, 1 H), 7.50 (t, *J* = 7.5 Hz, 1 H), 7.15 (dd, *J* = 8.4, 1.0 Hz, 1 H), 6.71 (s, 1 H), 3.87 (ab, *J* = 17.2 Hz, 1 H), 3.48 (ab, *J* = 17.2 Hz, 1 H), 2.66 (s, 3 H), 2.26 (s, 3 H) ppm. **^{13}C {H} NMR** (100 MHz, $CDCl_3$) δ 198.9, 176.1, 170.6, 153.6, 138.9, 136.1, 135.6, 134.4, 129.8, 129.1, 128.6, 126.6, 125.9, 122.3, 117.0, 63.8, 38.7, 26.6, 21.2 ppm. HRMS (ESI) calculated for $[M+H]^+$ $C_{19}H_{16}NO_3^+$: 306.1125, found: 306.1127. **HPLC analysis:** The enantiomeric excess was determined by HPLC on Daicel Chiralpak MD (2) Column (hexane/isopropanol = 90/10, flow rate = 1.0 mL/min, T = 25 °C, UV detection at λ = 254 nm); retention time: 5.173 min (major), 7.221 min (minor).

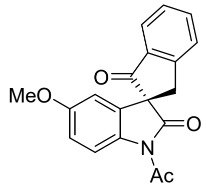

Chemical Formula: $C_{19}H_{15}NO_4$
Exact Mass: 321.1001

(R)-1'-acetyl-5'-methoxyspiro[indene-2,3'-indoline]-1,2'(3H)-dione (2p). Purification by column chromatography (0.1 mmol scale, eluent: ethyl acetate/hexane = 1/8), 95% isolated yield (white solid, 30.6 mg), 92% ee, $[\alpha]^{25}D$ = +19.9 (*c* 1.0, $CHCl_3$).

^{1}H NMR (400 MHz, $CDCl_3$) δ 8.23 (d, *J* = 9.0 Hz, 1 H), 7.83 (d, *J* = 7.8 Hz, 1 H), 7.74 (td, *J* = 7.4, 1.3 Hz, 1 H), 7.63 (d, *J* = 7.8 Hz, 1 H), 7.50 (t, *J* = 7.5 Hz, 1 H), 6.87 (dd, *J* = 9.0, 2.7 Hz, 1 H), 6.45 (d, *J* = 2.7 Hz, 1 H), 3.88 (ab, *J* = 17.4 Hz, 1 H), 3.72 (s, 3 H), 3.48 (ab, *J* = 17.2 Hz, 1 H), 2.66 (s, 3 H) ppm. **^{13}C {H} NMR** (100 MHz, $CDCl_3$) δ 198.6, 175.9, 170.4, 157.7, 153.5, 136.2, 134.7, 134.4, 130.4, 128.7, 126.7, 126.0, 118.2, 113.6, 108.3, 64.1, 55.8, 38.7, 26.6 ppm. HRMS (ESI) calculated for $[M+H]^+$ $C_{19}H_{16}NO_4^+$: 322.1074, found: 322.1073. **HPLC analysis:** The enantiomeric excess was determined by HPLC on Daicel Chiralpak MD (2) Column (hexane/isopropanol = 90/10, flow rate = 1.0 mL/min, T = 25 °C, UV detection at λ = 254 nm); retention time: 8.054 min (major), 10.414 min (minor).

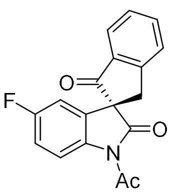

Chemical Formula: $C_{18}H_{12}FNO_3$
Exact Mass: 309.0801

(R)-1'-acetyl-5'-fluorospiro[indene-2,3'-indoline]-1,2'(3H)-dione (2q). Purification by column chromatography (0.1 mmol scale, eluent: ethyl acetate/hexane = 1/10), 93% isolated yield (white solid, 28.7 mg), 95% ee, $[\alpha]^{25}$D = +18.3 (*c* 1.0, $CHCl_3$). **^{1}H NMR** (400 MHz, $CDCl_3$) δ 8.30 (dd, *J* = 9.0, 4.7 Hz, 1H), 7.82 (d, *J* = 7.7 Hz, 1H), 7.78 – 7.69 (m, 1H), 7.63 (d, *J* = 7.7 Hz, 1H), 7.50 (t, *J* = 7.4 Hz, 1H), 7.05 (td, *J* = 9.0, 2.7 Hz, 1H), 6.64 (dd, *J* = 7.5, 2.7 Hz, 1H), 3.88 (d, *J* = 17.3 Hz, 1H), 3.47 (d, *J* = 17.3 Hz, 1H), 2.66 (s, 3H) ppm. **^{19}F NMR** (376 MHz, $CDCl_3$) δ -115.57 ppm. **^{13}C {H} NMR** (100 MHz, $CDCl_3$) δ197.8, 175.3, 170.3, 161.5, 159.1, 153.1, 137.2 (d, *J* = 2.5 Hz), 136.2, 133.9, 130.6 (d, *J* = 8.4 Hz), 128.7, 126.2 (d, *J* = 63.5 Hz), 118.5 (d, *J* = 7.9 Hz), 115.6 (d, *J* = 22.5 Hz), 109.3 (d, *J* = 24.8 Hz), 63.7 (d, *J* = 1.9 Hz), 38.3, 26.4 ppm. HRMS (ESI) calculated for $[M+H]^+$ $C_{18}H_{13}FNO_3^+$: 310.0874, found: 310.0883. **HPLC analysis:** The enantiomeric excess was determined by HPLC on Daicel Chiralpak MD (2) Column (hexane/isopropanol = 90/10, flow rate = 1.0 mL/min, T = 25 °C, UV detection at λ = 254 nm); retention time: 7.132 min (major), 8.443 min (minor).

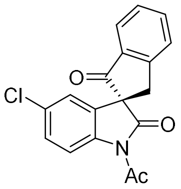

Chemical Formula: $C_{18}H_{12}ClNO_3$
Exact Mass: 325.0506

(R)-1'-acetyl-5'-chlorospiro[indene-2,3'-indoline]-1,2'(3H)-dione (2r). Purification by column chromatography (0.1 mmol scale, eluent: ethyl acetate/hexane = 1/8), 94% isolated yield (white solid, 30.5 mg), 92% ee, $[\alpha]^{25}D$ = +17.3 (*c* 1.0, $CHCl_3$). **^{1}H NMR** (400 MHz, $CDCl_3$) δ 8.26 (d, *J* = 8.7 Hz, 1H), 7.83 (d, *J* = 7.6 Hz, 1H), 7.75 (t, *J* = 7.4 Hz, 1H), 7.63 (d, *J* = 7.6 Hz, 1H), 7.51 (t, *J* = 7.4 Hz, 1H), 7.33 (dd, *J* = 8.6, 1.4 Hz, 1H), 6.89 (d, *J* = 1.3 Hz, 1H), 3.88 (d, *J* = 17.3 Hz, 1H), 3.48 (d, *J* = 17.3 Hz, 1H), 2.66 (s, 3H) ppm. **^{13}C {H} NMR** (100 MHz, $CDCl_3$) δ 197.8, 175.1, 170.3, 153.2, 139.6, 136.3, 133.9, 130.9, 130.6, 129.2, 128.7, 126.5, 125.9, 121.9, 118.3, 63.5, 38.3, 26.4 ppm. HRMS (ESI) calculated for $[M+H]^+$ $C_{18}H_{13}ClNO_3^+$: 326.0578, found: 326.0587. **HPLC analysis:** The enantiomeric excess was determined by HPLC on Daicel Chiralpak MD (2) Column (hexane/isopropanol = 90/10, flow rate = 1.0 mL/min, T = 25 °C, UV detection at λ = 254 nm); retention time: 7.070 min (major), 8.843 min (minor).

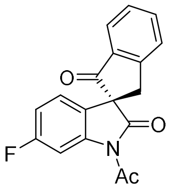

Chemical Formula: $C_{18}H_{12}FNO_3$
Exact Mass: 309.0801

(R)-1'-acetyl-6'-fluorospiro[indene-2,3'-indoline]-1,2'(3H)-dione (2s). Purification by column chromatography (0.1 mmol scale, eluent: ethyl acetate/hexane = 1/10), 93% isolated yield (white solid, 28.8 mg), 93% ee, $[\alpha]^{25}D$ = +19.1 (*c* 1.0, $CHCl_3$). **^{1}H NMR** (400 MHz, $CDCl_3$) δ 8.09 (d, *J* = 10.1 Hz, 1 H), 7.81 (d, *J* = 7.8 Hz, 1 H), 7.74 (t, *J* = 7.5 Hz, 1 H), 7.63 (d, *J* = 7.8 Hz, 1 H), 7.49 (t, *J* = 7.5 Hz, 1 H), 6.93 – 6.79 (m, 2 H), 3.87 (ab, *J* = 17.4 Hz, 1 H), 3.47 (ab, *J* = 17.2 Hz, 1 H), 2.66 (s, 3 H) ppm. **^{19}F NMR** (376 MHz, $CDCl_3$) δ -109.46 ppm. **^{13}C {H} NMR** (100 MHz, $CDCl_3$) δ 198.5, 175.7,

170.5, 162.9 (d, *J* = 245.8 Hz), 153.4, 142.2 (d, *J* = 12.5 Hz), 136.3, 134.2, 128.8, 126.7, 126.0, 124.5, 122.8 (d, *J* = 9.9 Hz), 112.4 (d, *J* = 22.7 Hz), 106.0 (d, *J* = 30.1 Hz), 63.4, 38.6, 26.5 ppm. HRMS (ESI) calculated for $[M+H]^+$ $C_{18}H_{13}FNO_3^+$: 310.0874, found: 310.0883. **HPLC analysis:** The enantiomeric excess was determined by HPLC on Daicel Chiralpak MD (2) Column (hexane/isopropanol = 90/10, flow rate = 1.0 mL/min, T = 25 °C, UV detection at λ = 254 nm); retention time: 6.993 min (major), 8.676 min (minor).

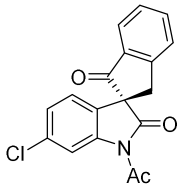

Chemical Formula: $C_{18}H_{12}ClNO_3$
Exact Mass: 325.0506

(R)-1'-acetyl-6'-chlorospiro[indene-2,3'-indoline]-1,2'(3H)-dione (2t). Purification by column chromatography (0.1 mmol scale, eluent: ethyl acetate/hexane = 1/8), 91% isolated yield (white solid, 29.5 mg), 88% ee, $[\alpha]^{25}D$ = +25.6 (*c* 1.0, $CHCl_3$). **^{1}H NMR** (400 MHz, $CDCl_3$) δ 8.37 (d, *J* = 1.8 Hz, 1 H), 7.82 (d, *J* = 7.7 Hz, 1 H), 7.75 (td, *J* = 7.5, 1.3 Hz, 1 H), 7.63 (d, *J* = 7.7 Hz, 1 H), 7.50 (t, *J* = 7.0 Hz, 1 H), 7.14 (dd, *J* = 8.1, 2.0 Hz, 1 H), 6.85 (d, *J* = 8.1 Hz, 1 H), 3.88 (ab, *J* = 17.2 Hz, 1 H), 3.47 (ab, *J* = 17.4 Hz, 1 H), 2.67 (s, 3 H) ppm. **^{13}C {H} NMR** (100 MHz, $CDCl_3$) δ 198.2, 175.5, 170.5, 153.4, 142.1, 136.4, 135.1, 134.2, 128.8, 127.5, 126.7, 126.1, 125.8, 122.7, 117.9, 63.5, 38.5, 26.6 ppm. HRMS (ESI) calculated for $[M+H]^+$ $C_{18}H_{13}ClNO_3^+$: 326.0578, found: 326.0590. **HPLC analysis:** The enantiomeric excess was determined by HPLC on Daicel Chiralpak MD (2) Column (hexane/isopropanol = 90/10, flow rate = 1.0 mL/min, T = 25 °C, UV detection at λ = 254 nm); retention time: 8.423 min (major), 10.670 min (minor).

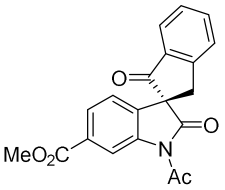

Chemical Formula: $C_{20}H_{15}NO_5$
Exact Mass: 349.0950

Methyl-(R)-1'-acetyl-1,2'-dioxo-1,3-dihydrospiro[indene-2,3'-indoline]-6'-carboxylate (2u). Purification by column chromatography (0.1 mmol scale, eluent: ethyl acetate/hexane = 1/8), 88% isolated yield (white solid, 30.8 mg), 93% ee, $[\alpha]^{25}D$ = +19.4 (*c* 1.0, $CHCl_3$). **^{1}H NMR** (400 MHz, $CDCl_3$) δ 8.93 (s, 1 H), 7.88 (dd, *J* = 7.9, 1.5 Hz, 1 H), 7.83 (d, *J* = 7.7 Hz, 1 H), 7.75 (td, *J* = 7.4, 1.3 Hz, 1 H), 7.64 (d, *J* = 7.7 Hz, 1 H), 7.51 (t, *J* = 7.5 Hz, 1 H), 7.00 (d, *J* = 7.9 Hz, 1 H), 3.93 (s, 3 H), 3.91 (ab, *J* = 17.4 Hz, 1 H), 3.52 (ab, *J* = 17.4 Hz, 1 H), 2.69 (s, 3 H) ppm. **^{13}C {H} NMR** (100 MHz, $CDCl_3$) δ 197.8, 175.4, 170.5, 166.5, 153.4, 141.4, 136.4, 134.1, 133.9, 131.4, 128.9, 127.5, 126.7, 126.1, 121.7, 118.1, 64.0, 52.5, 38.4, 26.6 ppm. HRMS (ESI) calculated for $[M+H]^+$ $C_{20}H_{16}NO_5^+$: 350.1023, found: 350.1030. **HPLC analysis:** The enantiomeric excess was determined by HPLC on Daicel Chiralpak MD (2) Column (hexane/isopropanol = 90/10, flow rate = 1.0 mL/min, T = 25 °C, UV detection at λ = 254 nm); retention time: 7.954 min (major), 9.890 min (minor).

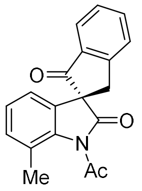

Chemical Formula: $C_{19}H_{15}NO_3$
Exact Mass: 305.1052

(R)-1'-acetyl-7'-methylspiro[indene-2,3'-indoline]-1,2'(3H)-dione (2v). Purification by column chromatography (0.1 mmol scale, eluent: ethyl acetate/hexane = 1/10), 95% isolated yield (white solid, 29.0 mg), 90% ee, $[\alpha]^{25}D$ = +23.2 (*c* 1.0, $CHCl_3$). **^{1}H NMR** (400 MHz, $CDCl_3$) δ 7.80 (d, *J* = 7.7 Hz, 1 H), 7.72 (td, *J* = 7.5, 1.3 Hz, 1 H), 7.61 (d, *J* = 7.7 Hz, 1 H), 7.47 (t, *J* = 7.0 Hz, 1 H), 7.18 (d, *J* = 7.7 Hz, 1 H), 7.07 (t, *J* = 7.6 Hz, 1 H), 6.74 (d, *J* = 6.7 Hz, 1 H), 3.86 (ab, *J* = 17.4 Hz, 1 H), 3.47 (ab, *J* = 17.4 Hz, 1 H), 2.69 (s, 3 H), 2.25 (s, 3 H) ppm. **^{13}C {H} NMR** (100 MHz, $CDCl_3$) δ 198.7, 176.6, 170.0, 153.5, 139.6, 136.1, 134.3, 132.4, 130.5, 128.6, 127.1, 126.6, 125.9, 125.8, 119.2, 64.5, 38.5, 26.5, 21.7 ppm. HRMS (ESI) calculated for $[M+H]^+$ $C_{19}H_{16}NO_3^+$: 306.1125, found: 306.1126. **HPLC analysis:** The enantiomeric excess was determined by HPLC on Daicel Chiralpak MD (2) Column (hexane/isopropanol = 90/10, flow rate = 1.0 mL/min, T = 25 °C, UV detection at λ = 254 nm); retention time: 5.748 min (major),

7.903 min (minor).

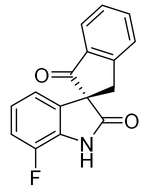

Chemical Formula: $C_{16}H_{10}FNO_2$
Exact Mass: 267.0696

(R)-7'-fluorospiro[indene-2,3'-indoline]-1,2'(3H)-dione [2w]. Purification by column chromatography (0.1 mmol scale, eluent: ethyl acetate/hexane = 1/5), 94% isolated yield (white solid, 25 mg), >99% ee, $[\alpha]^{25}$D = +20.4 (*c* 1.0, $CHCl_3$). **^{1}H NMR** (400 MHz, DMSO-d_6) δ 11.27 (s, 1 H), 7.82 (t, *J* = 7.5 Hz, 1 H), 7.77 – 7.72 (m, 2 H), 7.54 (t, *J* = 7.4 Hz, 1 H), 7.18 (ddd, *J* = 10.5, 8.4, 1.2 Hz, 1 H), 6.97 (td, *J* = 7.9, 4.7 Hz, 1 H), 6.91 (d, *J* = 7.5 Hz, 1 H), 3.66 (ab, *J* = 17.6 Hz, 1 H), 3.53 (ab, *J* = 17.6 Hz, 1 H) ppm. **^{19}F NMR** (376 MHz, DMSO-d_6) δ -132.23 ppm. **^{13}C {H} NMR** (100 MHz, DMSO-d_6) δ 199.6, 175.6, 154.4, 146.3 (d, *J* = 242.8 Hz), 136.1, 134.3, 133.2 (d, *J* = 4.4 Hz), 130.3 (d, *J* = 12.1 Hz), 128.3, 126.9, 124.7, 123.0 (d, *J* = 5.9 Hz), 119.1 (d, *J* = 3.3 Hz), 115.9 (d, *J* = 17.2 Hz), 63.5 (d, *J* = 2.2 Hz), 36.9 ppm. HRMS (ESI) calculated for $[M+H]^+$ $C_{16}H_{11}FNO_2^+$: 268.0768, found: 268.0765. **HPLC analysis:** The enantiomeric excess was determined by HPLC on Daicel Chiralpak MD (2) Column (hexane/isopropanol = 80/20, flow rate = 1.0 mL/min, T = 25 °C, UV detection at λ = 254 nm); retention time: 5.774 min (major).

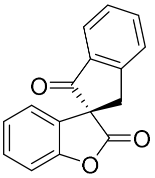

Chemical Formula: $C_{16}H_{10}O_3$
Exact Mass: 250.0630

(R)-2H-spiro[benzofuran-3,2'-indene]-1',2(3'H)-dione [2x]. Purification by column chromatography (0.1 mmol scale, eluent: ethyl acetate/hexane = 1/10), 90% isolated yield (white solid, 22.4 mg), 90% ee, $[\alpha]^{25}$D = +16.7 (*c* 1.0, $CHCl_3$). **^{1}H NMR** (400 MHz, $CDCl_3$) δ 7.82 (d, *J* = 7.7 Hz, 1H), 7.76 – 7.66 (m, 1H), 7.61 (d, *J* = 7.7 Hz, 1H), 7.49 (t, *J* = 7.5 Hz, 1H), 7.35 (td, *J* = 8.1, 1.2 Hz, 1H), 7.20 (d, *J* = 8.0 Hz, 1H), 7.11 (t,

J = 7.6 Hz, 1H), 6.95 (dd, J = 7.5, 0.7 Hz, 1H), 3.89 (d, J = 17.3 Hz, 1H), 3.52 (d, J = 17.3 Hz, 1H) ppm. **^{13}C {H} NMR** (100 MHz, $CDCl_3$) δ 197.5, 174.1, 154.2, 152.9, 136.2, 133.9, 129.8, 128.6, 128.4, 126.5, 125.7, 124.7, 122.4, 111.1, 61.2, 38.4 ppm. HRMS (ESI) calculated for $[M+H]^+$ $C_{16}H_{11}O_3^+$: 251.0703, found: 251.0707. **HPLC analysis:** The enantiomeric excess was determined by HPLC on Daicel Chiralpak MD (2) Column (hexane/isopropanol = 95/5, flow rate = 0.5 mL/min, T = 25 °C, UV detection at λ = 254 nm); retention time: 17.412 min (major), 19.394 min (minor).

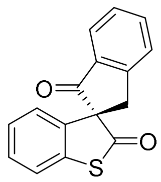

Chemical Formula: $C_{16}H_{10}O_2S$
Exact Mass: 266.0402

(R)-2H-spiro[benzo[b]thiophene-3,2'-indene]-1',2(3'H)-dione [2y]. Purification by column chromatography (0.1 mmol scale, eluent: ethyl acetate/hexane = 1/10), 94% isolated yield (white solid, 25.1 mg), 82% ee, $[\alpha]^{25}D$ = +27.0 (c 1.0, $CHCl_3$). **^{1}H NMR** (400 MHz, $CDCl_3$) δ 7.81 (d, J = 7.7 Hz, 1 H), 7.72 (td, J = 7.5, 1.3 Hz, 1 H), 7.61 (d, J = 7.7 Hz, 1 H), 7.51 – 7.43 (m, 2 H), 7.34 (td, J = 7.6, 1.3 Hz, 1 H), 7.18 (td, J = 7.6, 1.3 Hz, 1 H), 6.90 (d, J = 7.7 Hz, 1 H), 3.92 (ab, J = 17.5 Hz, 1 H), 3.48 (ab, J = 17.5 Hz, 1 H) ppm. **^{13}C {H} NMR** (100 MHz, $CDCl_3$) δ 203.3, 197.8, 153.4, 137.7, 136.8, 136.1, 133.9, 129.4, 128.7, 127.0, 126.5, 126.1, 123.5, 123.2, 73.7, 39.8 ppm. HRMS (ESI) calculated for $[M+H]^+$ $C_{16}H_{11}O_2S^+$: 267.0474, found: 267.0475. **HPLC analysis:** The enantiomeric excess was determined by HPLC on Daicel Chiralpak MD (2) Column (hexane/isopropanol = 92/8, flow rate = 1.0 mL/min, T = 25 °C, UV detection at λ = 254 nm); retention time: 8.791 min (minor), 9.702 min (major).

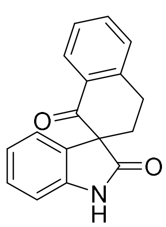

Chemical Formula: $C_{17}H_{13}NO_2$
Exact Mass: 263.0946

(R)-3',4'-dihydro-1'H-spiro[indoline-3,2'-naphthalene]-1',2-dione [2z]. Purification

by column chromatography (0.1 mmol scale, eluent: ethyl acetate/hexane = 1/10), 92% isolated yield (white solid, 24.2 mg), 38% ee, $[\alpha]^{25}D = 27.3$ (*c* 1.0, $CHCl_3$). **^{1}H NMR** (400 MHz, $CDCl_3$) δ 9.26 (s, 1H), 8.07 (dd, *J* = 8.4, 1.1 Hz, 1H), 7.58 (td, *J* = 7.7, 1.4 Hz, 1H), 7.37 (t, *J* = 7.4 Hz, 2H), 7.21 (dt, *J* = 7.7, 3.9 Hz, 1H), 7.08 – 6.85 (m, 3H), 3.36 (dd, *J* = 7.5, 5.3 Hz, 2H), 2.81 (dt, *J* = 13.7, 7.8 Hz, 1H), 2.35 (dt, *J* = 13.6, 5.0 Hz, 1H) ppm. **^{13}C {H} NMR** (100 MHz, $CDCl_3$) δ 193.2, 178.2, 143.7, 141.5, 134.4, 131.4, 129.8, 129.2, 129.0, 128.7, 127.3, 124.0, 122.5, 110.9, 60.7, 31.0, 24.8 ppm. HRMS (ESI) calculated for $[M+H]^+$ $C_{17}H_{14}NO_2^+$: 264.1019, found: 264.1024. **HPLC analysis:** The enantiomeric excess was determined by HPLC on Daicel Chiralpak MD (2) Column (hexane/isopropanol = 90/10, flow rate = 1.0 mL/min, T = 25 °C, UV detection at λ = 254 nm); retention time: 12.680 min (major), 17.674 min (minor).

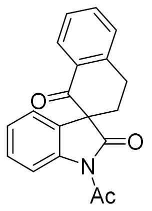

Chemical Formula: $C_{19}H_{15}NO_3$
Exact Mass: 305.1052

(R)-1-acetyl-3',4'-dihydro-1'H-spiro[indoline-3,2'-naphthalene]-1',2-dione (2aa). Purification by column chromatography (0.1 mmol scale, eluent: ethyl acetate/hexane = 1/10), 90% isolated yield (white solid, 27.3 mg), 0% ee. **^{1}H NMR** (400 MHz, $CDCl_3$) δ 8.32 (d, *J* = 8.3 Hz, 1H), 8.12 – 7.99 (m, 1H), 7.60 (td, *J* = 7.6, 1.3 Hz, 1H), 7.46 – 7.32 (m, 3H), 7.20 – 7.09 (m, 1H), 7.05 (dd, *J* = 7.5, 1.1 Hz, 1H), 3.46 – 3.27 (m, 2H), 2.81 (dt, *J* = 13.8, 7.6 Hz, 1H), 2.72 (s, 3H), 2.43 - 2.37 (m, 1H) ppm. **^{13}C {H} NMR** (100 MHz, $CDCl_3$) δ 192.4, 176.2, 170.9, 143.5, 140.4, 134.7, 131.0, 129.4, 129.3, 128.8, 128.5, 127.5, 125.4, 123.4, 117.3, 60.5, 31.7, 26.9, 24.6 ppm. HRMS (ESI) calculated for $[M+H]^+$ $C_{19}H_{16}NO_3^+$: 306.1125, found: 306.1116.

5. Synthetic applications

5.1 Gram-scale synthesis of 2a

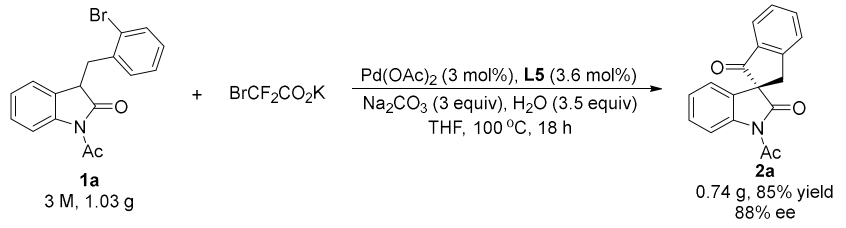

A reaction tube was charged with $Pd(OAc)_2$ (20 mg, 0.09 mmol, 0.03 equiv), **L5** (125 mg, 0.108 mmol, 1.2 equiv to [Pd]), **1a** (1.03 g, 3 mmol, 1.0 equiv), $BrCF_2CO_2K$ (1.28 g, 6 mmol, 2.0 equiv), Na_2CO_3 (954 mg, 9 mmol, 3.0 equiv), degassed H_2O (10.5 mmol, 3.5 equiv) and THF (40.0 mL) under N_2 atmosphere. The reaction vessel was sealed using a PTFE septum, and the mixture was stirred at 100 °C for 18 h. After completion of the reaction, the resulting solution was cooled to room temperature; then it was diluted with CH_2Cl_2 (20 mL), washed with water (20 mL), extracted with CH_2Cl_2 (3×20 mL), and dried over anhydrous Na_2SO_4 and concentrated in vacuo. The crude product was purified by flash column chromatography on silica gel to give the desired product final product **2a** in 85% yield (0.74 g) with 88% ee.

5.2 Synthesis of 3a

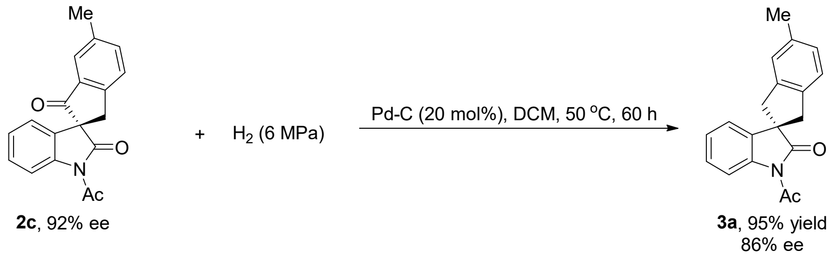

To an autoclave was added **2c** (30.5 mg, 0.1 mmol), Pd/C (10%) (wetted with ca. 55% Water) (47 mg, 20 mol%) and 2 mL CH_2Cl_2. The autoclave was then charged with H_2 (60 bar), and the reaction mixture was allowed to stir at 50 °C for 60 h. The Pd/C was filtered off and the filtrate was evaporated under reduced pressure. The residue was purified by flash chromatography on silica gel to afford the desired **3a** product in 95% yield (27.6 mg) with 86% ee.

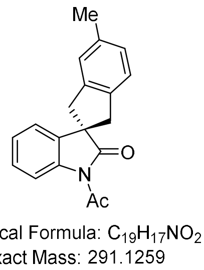

(S)-1′-acetyl-5-methyl-1,3-dihydrospiro[indene-2,3′-indolin]-2′-one (3a). Purification by column chromatography (eluent: ethyl acetate/hexane = 1/10), 95% isolated yield (white solid, 27.6 mg), 86% ee, $[\alpha]^{25}D$ = +12.4 (*c* 1.0, $CHCl_3$). **^{1}H NMR** (400 MHz, $CDCl_3$) δ 8.25 (d, *J* = 8.2 Hz, 1 H), 7.29 (td, *J* = 8.2, 1.4 Hz, 1 H), 7.15 (d, *J* = 7.6 Hz, 1 H), 7.11 – 7.03 (m, 3 H), 6.92 (dd, *J* = 7.5, 0.9 Hz, 1 H), 3.69 – 3.41 (m, 2 H), 3.14 (d, *J* = 15.6 Hz, 2 H), 2.72 (s, 3 H), 2.38 (s, 3 H) ppm. **^{13}C {H} NMR** (100 MHz, $CDCl_3$) δ 181.4, 171.2, 140.8, 138.8, 137.6, 137.2, 135.1, 128.5, 128.2, 125.7, 125.4, 124.4, 121.4, 116.6, 54.8, 45.5, 45.3, 26.9, 21.5 ppm. HRMS (ESI) calculated for $[M+H]^+$ $C_{19}H_{18}NO_2^+$: 292.1332, found: 292.1338. **HPLC analysis:** The enantiomeric excess was determined by HPLC on Daicel Chiralpak MD (2) Column (hexane/isopropanol = 90/10, flow rate = 1.0 mL/min, T = 25 °C, UV detection at λ = 254 nm); retention time: 3.020 min (major), 3.482 min (minor).

5.3 Synthesis of 3b

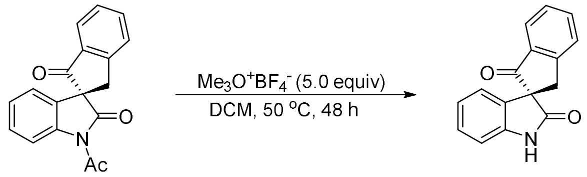

2a, 96% ee

3b, 85% yield
96% ee

To a Schlenk tube, **2a** (29.1 mg, 0.1 mmol, 96% ee), $Me_3O^+BF_4^-$ (74 mg, 0.5 mmol) and 2 mL CH_2Cl_2 were added. The mixture was stirred at 50 °C for 48 h under Air atmosphere. After completion of the reaction, the resulting solution was cooled to room temperature; then it was diluted with CH_2Cl_2 (6 mL), washed with water (6 mL), extracted with CH_2Cl_2 (6×3 mL), and dried over anhydrous Na_2SO_4 and concentrated in vacuo. The residue was purified by flash chromatography on silica gel to afford the desired **3b** product in 85% yield (21.1 mg) with 96% ee.

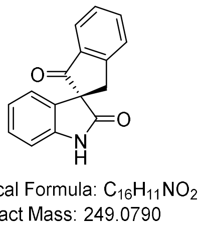

(R)-spiro[indene-2,3'-indoline]-1,2'(3H)-dione (3b).[3] Purification by column chromatography (eluent: ethyl acetate/hexane = 1/10), 85% isolated yield (white solid, 21.1 mg), 96% ee, $[\alpha]^{25}$D = +17.6 (*c* 1.0, $CHCl_3$). **^{1}H NMR** (400 MHz, $CDCl_3$) δ 8.91 (s, 1 H), 7.83 (d, *J* = 7.6 Hz, 1 H), 7.71 (t, *J* = 7.4 Hz, 1 H), 7.62 (d, *J* = 7.6 Hz, 1 H), 7.47 (t, *J* = 7.4 Hz, 1 H), 7.22 (t, *J* = 7.6 Hz, 1 H), 6.99 – 6.91 (m, 2 H), 6.88 (d, *J* = 7.3 Hz, 1 H), 3.87 (ab, *J* = 17.2 Hz, 1 H), 3.47 (ab, *J* = 17.3 Hz, 1 H) ppm. **^{13}C {H} NMR** (100 MHz, $CDCl_3$) δ 199.9, 177.4, 153.9, 142.2, 135.9, 135.2, 130.8, 129.0, 128.3, 126.7, 125.7, 123.0, 122.5, 110.6, 63.7, 37.7 ppm. **HPLC analysis:** The enantiomeric excess was determined by HPLC on Daicel Chiralpak MD (2) Column (hexane/isopropanol = 90/10, flow rate = 1.0 mL/min, T = 25 °C, UV detection at λ = 254 nm); retention time: 12.404 min (major), 18.059 min (minor).

5.4 Synthesis of 3c

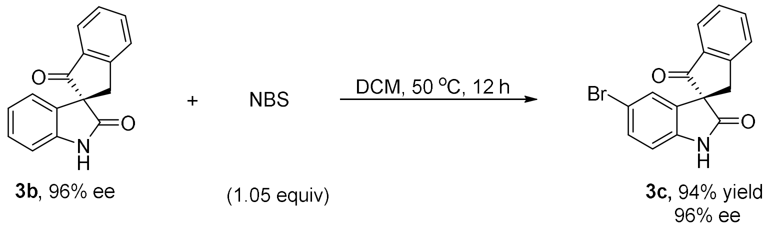

To a Schlenk tube, **3b** (24.9 mg, 0.1 mmol, 96% ee), NBS (18.7 mg, 0.105 mmol) and 2 mL CH_2Cl_2 were added. The mixture was stirred at 50 °C for 12 h under Air atmosphere. After completion of the reaction, the resulting solution was cooled to room temperature; the resulting mixture was added to saturated aqueous NH_4Cl (10 mL). The mixture was extracted with CH_2Cl_2 (3×10 mL) and the combined organic layers were dried over Na_2SO_4, filtered, and concentrated. The residue was purified by flash chromatography on silica gel to afford the desired **3c** product in 94% yield (30.9 mg) with 96% ee.

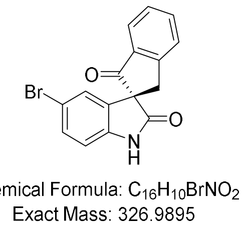

(R)-5'-bromospiro[indene-2,3'-indoline]-1,2'(3H)-dione (3c). Purification by column chromatography (eluent: ethyl acetate/hexane = 1/6), 94% isolated yield (white solid, 30.9 mg), 96% ee, $[\alpha]^{25}D$ = +15.7 (*c* 1.0, $CHCl_3$). **^{1}H NMR** (400 MHz, $CDCl_3$) δ 8.41 (s, 1 H), 7.83 (d, *J* = 7.7 Hz, 1 H), 7.78 – 7.69 (m, 1 H), 7.62 (d, *J* = 7.7 Hz, 1 H), 7.49 (t, *J* = 7.5 Hz, 1 H), 7.36 (dd, *J* = 8.3, 1.9 Hz, 1 H), 7.01 (d, *J* = 1.9 Hz, 1 H), 6.83 (d, *J* = 8.3 Hz, 1 H), 3.85 (d, *J* = 17.3 Hz, 1 H), 3.46 (d, *J* = 17.3 Hz, 1 H) ppm. **^{13}C {H} NMR** (100 MHz, $CDCl_3$) δ 198.9, 176.4, 153.7, 141.0, 136.2, 134.8, 132.7, 131.9, 128.6, 126.8, 125.9, 125.9, 115.6, 111.9, 63.5, 37.5 ppm. HRMS (ESI) calculated for $[M+H]^+$ $C_{16}H_{11}BrNO_2^+$: 327.9968, found: 327.9961. **HPLC analysis:** The enantiomeric excess was determined by HPLC on Daicel Chiralpak OD-H Column (hexane/isopropanol = 90/10, flow rate = 0.8 mL/min, T = 25 °C, UV detection at λ = 254 nm); retention time: 25.816 min (major), 30.043 min (minor).

5.5 Synthesis of 3d[4]

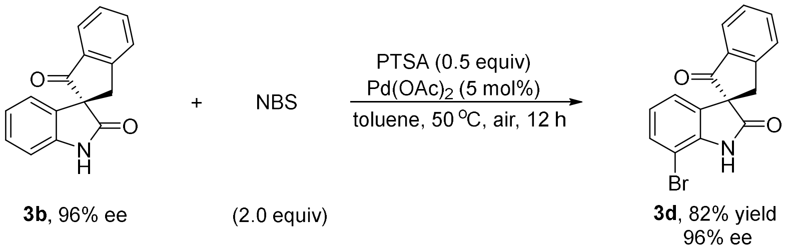

To a Schlenk tube, **3b** (24.9 mg, 0.1 mmol, 96% ee), NBS (35.6 mg, 0.2 mmol), PTSA (8.6 mg, 0.05 mmol), $Pd(OAc)_2$ (1.2 mg, 0.005 mmol) and 2 mL toluene were added. The mixture was stirred at 50 °C for 12 hours under Air atmosphere. After completion of the reaction, the resulting solution was cooled to room temperature; the resulting mixture was added to saturated aqueous NH_4Cl (10 mL). The mixture was extracted with CH_2Cl_2 (3×10 mL) and the combined organic layers were dried over Na_2SO_4, filtered, and concentrated. The residue was purified by flash chromatography on silica

gel to afford the desired **3d** product in 82% yield (26.8 mg) with 96% ee.

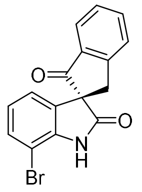

Chemical Formula: $C_{16}H_{10}BrNO_2$
Exact Mass: 326.9895

(R)-7'-bromospiro[indene-2,3'-indoline]-1,2'(3H)-dione (3d). Purification by column chromatography (eluent: ethyl acetate/hexane = 1/6), 82% isolated yield (white solid, 26.8 mg), 96% ee, $[\alpha]^{25}D$ = +20.1 (*c* 1.0, CH_3Cl). **^{1}H NMR** (400 MHz, $CDCl_3$) δ 9.00 (s, 1H), 7.83 (d, *J* = 7.7 Hz, 1H), 7.77 – 7.68 (m, 1H), 7.62 (d, *J* = 7.7 Hz, 1H), 7.49 (t, *J* = 7.4 Hz, 1H), 7.34 (dd, *J* = 8.3, 1.9 Hz, 1H), 7.00 (d, *J* = 1.8 Hz, 1H), 6.80 (d, *J* = 8.3 Hz, 1H), 3.85 (d, *J* = 17.4 Hz, 1H), 3.45 (d, *J* = 17.4 Hz, 1H) ppm. **^{13}C {H} NMR** (100 MHz, $CDCl_3$) δ 199.1, 176.9, 153.7, 141.2, 136.2, 134.8, 132.7, 131.9, 128.6, 126.8, 125.9, 125.7, 115.5, 112.1, 63.6, 37.5 ppm. HRMS (ESI) calculated for $[M+H]^+$ $C_{16}H_{11}BrNO_2^+$: 327.9968, found: 327.9957. **HPLC analysis:** The enantiomeric excess was determined by HPLC on Daicel Chiralpak AD-H Column (hexane/isopropanol = 70/30, flow rate = 1.0 mL/min, T = 25 °C, UV detection at λ = 254 nm); retention time: 7.869 min (minor), 17.357 min (major).

5.6 Synthesis of 3e

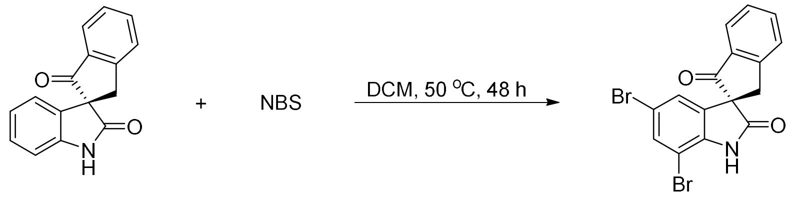

3b, 96% ee (2.0 equiv) **3e**, 92% yield 96% ee

To a Schlenk tube, **3b** (24.9 mg, 0.1 mmol, 96% ee), NBS (35.6 mg, 0.2 mmol) and 2 mL CH_2Cl_2 were added. The mixture was stirred at 50 °C for 48 hours under Air atmosphere. After completion of the reaction, the resulting solution was cooled to room temperature; the resulting mixture was added to saturated aqueous NH_4Cl (10 mL). The mixture was extracted with CH_2Cl_2 (3×10 mL) and the combined organic layers were

dried over Na_2SO_4, filtered, and concentrated. The residue was purified by flash chromatography on silica gel to afford the desired **3e** product in 92% yield (37.1 mg) with 96% ee.

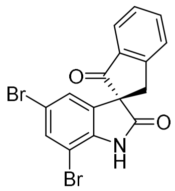

(R)-5',7'-dibromospiro[indene-2,3'-indoline]-1,2'(3H)-dione (3e). Purification by column chromatography (eluent: ethyl acetate/hexane = 1/6), 92% isolated yield (white solid, 37.1 mg), 96% ee, $[\alpha]^{25}D$ = +19.1 (*c* 1.0, $CHCl_3$). **^{1}H NMR** (400 MHz, DMSO) δ 11.22 (s, 1H), 7.82 (dd, *J* = 11.2, 4.5 Hz, 1H), 7.73 (dd, *J* = 10.7, 4.9 Hz, 3H), 7.54 (t, *J* = 7.3 Hz, 1H), 7.43 (d, *J* = 1.7 Hz, 1H), 3.86 – 3.44 (m, 2H) ppm. **^{13}C {H} NMR** (100 MHz, DMSO) δ 199.1, 175.2, 154.6, 142.4, 136.2, 134.1, 133.4, 133.3, 128.3, 127.0, 125.6, 124.8, 114.1, 102.6, 64.5, 36.8. HRMS (ESI) calculated for $[M+H]^+$ $C_{16}H_{10}Br_2NO_2^+$: 405.9073, found: 405.9066. **HPLC analysis:** The enantiomeric excess was determined by HPLC on Daicel Chiralpak OD-H Column (hexane/isopropanol = 90/10, flow rate = 0.8 mL/min, T = 25 °C, UV detection at λ = 254 nm); retention time: 23.605 min (minor), 25.675 min (major).

Supplementary Discussion

6. Control experiments

6.1 The ^{18}O-labelling experiment

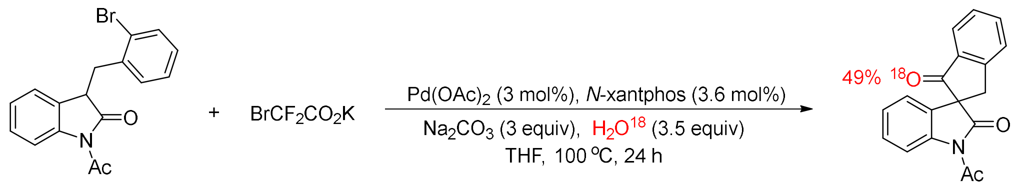

A reaction tube was charged with $PdCl_2$ (1.1 mg, 0.006 mmol, 0.03 equiv), *N*-xantphos (4.0 mg, 0.0072 mmol, 1.2 equiv to [Pd]), **1a** (0.2 mmol, 1.0 equiv), $BrCF_2CO_2K$ (85.2 mg, 0.4 mmol, 2.0 equiv), Na_2CO_3 (63.6 mg, 0.6 mmol, 3.0 equiv), $H_2{}^{18}O$ (0.7 mmol, 3.5 equiv) and THF (4.0 mL) under N_2 atmosphere. The reaction vessel was sealed using a PTFE septum, and the mixture was stirred at 100 °C for 24 h. After complete conversion, the resulting mixture was cooled to rt, and using GC-MS to detect the desired product rac-**2a**-^{18}O (Supplementary Figure 1). Around 49% of the O atom in the carbonyl group was labeled by mass analysis, which indicated the O atom of the newly formed carbonyl group was probably originated from the external water.

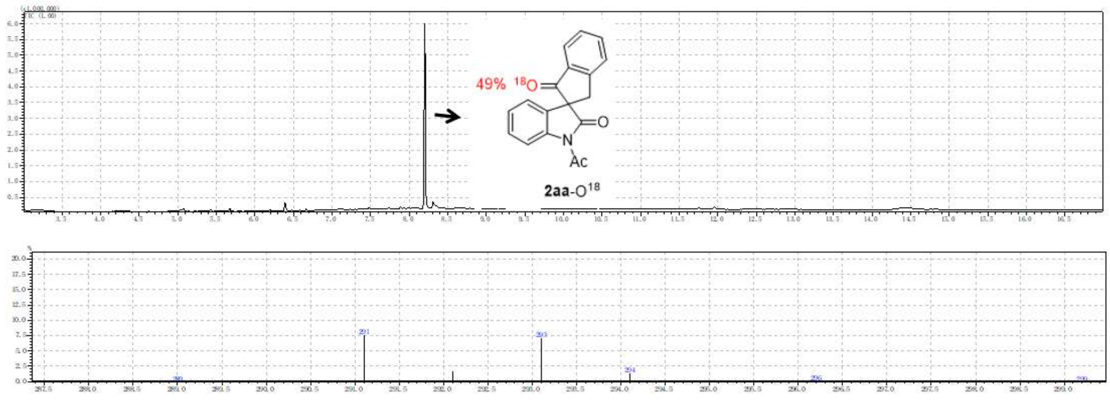

Supplementary Figure 1. The GCMS spectra of rac-**2a-**^{18}O.

Supplementary Table 11. MS spectrum peak list of rac-**2a-**^{18}O

M/Z	Abundance	M/Z	Abundance	M/Z	Abundance
35.00	30	38.00	58	41.15	573
36.00	36	39.00	44	42.25	20129
37.00	47	40.00	46	43.15	31185
44.15	29227	84.15	17819	213.50	1478
45.25	29161	85.15	17705	214.55	1431

46.15	28608	86.25	17681	215.45	1402
47.15	27813	87.15	17575	216.45	1363
48.25	27289	88.15	17508	217.45	1307
49.15	27021	89.15	17420	218.50	1263
50.15	26160	90.25	17393	219.45	1233
51.15	24942	91.15	17309	220.55	1201
52.15	24193	92.15	17246	221.45	1200
53.15	23585	93.15	17151	222.45	1147
54.15	23593	94.15	17066	223.45	1123
55.15	23028	95.15	17058	224.45	1088
56.25	22684	96.15	16965	225.45	1066
57.15	22689	97.15	16230	226.45	1046
58.25	22475	98.15	1838	227.55	1035
59.25	22013	187.95	5902	228.55	1003
60.25	22409	188.95	11879	229.45	980
61.25	22809	189.95	16996	230.55	966
62.25	24008	190.95	6949	231.55	943
63.15	25498	191.95	704	232.45	934
64.15	19916	193.55	761	233.20	930
65.15	19725	194.55	2897	234.15	893
66.15	19361	195.50	3924	235.15	861
67.15	19229	196.45	3397	236.20	894
68.15	19164	197.45	2605	237.25	873
69.25	19065	198.45	2230	238.20	823
70.15	18958	199.55	2243	239.15	814
71.15	18843	200.55	2327	240.15	818
72.15	18762	201.45	2323	241.05	792
73.15	18683	202.45	2180	242.15	807
74.15	18610	203.45	2082	243.20	810
75.15	18521	204.45	1968	244.25	792
76.15	18452	205.45	1908	245.25	760
77.20	18364	206.45	1845	246.25	738
78.15	18297	207.50	1818	247.25	740
79.15	18193	208.45	1755	248.25	733
80.15	18123	209.45	1674	249.15	722
81.20	18049	210.45	1650	250.25	721
82.15	17958	211.45	1563	251.15	693
83.15	17904	212.45	1541	252.15	681
253.25	679	269.15	600	285.25	523
254.15	711	270.15	594	286.15	540
255.15	686	271.25	583	287.25	511
256.20	663	272.15	569	288.25	528
257.25	666	273.20	566	289.25	507

258.20	641	274.20	580	290.15	496
259.15	661	275.20	567	**291.15 (M)**	528
260.20	634	276.25	555	292.25	485
261.20	614	277.15	545	**293.25 (M+2)**	504
262.25	627	278.25	566	294.20	524
263.15	626	279.25	547	295.20	489
264.25	628	280.25	541	296.25	499
265.15	617	281.25	549	297.25	496
266.20	599	282.25	539	298.20	514
267.15	574	283.20	586	299.25	504
268.25	594	284.20	537	300.20	476

I(M) : I(M+2) = 528 : 504 = 1 : 0.955; ^{18}O enrichment = 0.955/1.955 = 49%

6.2 Evaluation of organic bases without oxygen atoms

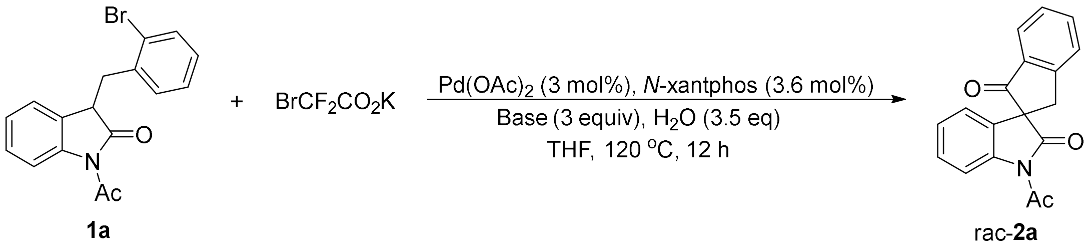

Supplementary Table 12. Evaluation of different organic bases

Entry	Base	Yield (%)
1	Et_2NH	41
2	Et_3N	0
3	$^iPr_2NH$	37
4	DBU	46
5	$^iPr_2NEt$	53
6	Piperidine	0

A reaction tube was charged with $Pd(OAc)_2$ (0.003 mmol, 0.03 equiv), *N*-xantphos (0.0036 mmol, 1.2 equiv to [Pd]), **1a** (0.1 mmol, 1.0 equiv), $BrCF_2CO_2K$ (0.2 mmol, 2.0 equiv), Base (0.3 mmol, 3.0 equiv), degassed H_2O (0.35 mmol, 3.5 equiv) and THF (2.0 mL) under N_2 atmosphere. The reaction vessel was sealed using a PTFE septum, and the mixture was stirred at 120 °C for 12 h. After complete conversion, the resulting mixture was cooled and evaperated under reduced pressure, then the residue was purified by flash chromatography to give the compound rac-**2a**.

Conclusion: The target product **2a** can be obtained smoothly using organic bases

without oxygen atoms, which indicates that the oxygen atom in the newly formed carbonyl group is likely not from Na_2CO_3.

6.3 Trapping experiment of the difluorocarbene intermediate

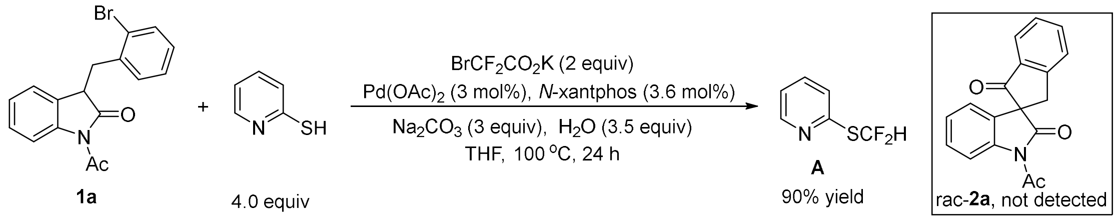

A reaction tube was charged with $Pd(OAc)_2$ (1.4 mg, 0.006 mmol, 0.03 equiv), N-xantphos (4.0 mg, 0.0072 mmol, 1.2 equiv to [Pd]), **1a** (68.8 mg, 0.2 mmol, 1.0 equiv), $BrCF_2CO_2K$ (85.2 mg, 0.4 mmol, 2.0 equiv), Na_2CO_3 (63.6 mg, 0.6 mmol, 3.0 equiv), pyridine-2-thiol (88.8 mg, 0.8 mmol, 4.0 equiv), degassed H_2O (0.7 mmol, 3.5 equiv) and THF (4.0 mL) under N_2 atmosphere. The reaction vessel was sealed using a PTFE septum, and the mixture was stirred at 100 °C for 24 h. After complete conversion, the resulting mixture was cooled and evaperated under reduced pressure, then the residue was purified by flash chromatography to give the compound **A**.

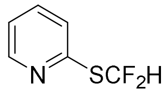

Chemical Formula: $C_6H_5F_2NS$
Exact Mass: 161.0111

2-((difluoromethyl)thio)pyridine (A).[5] Known compound.[7] Purification by column chromatography (eluent: ethyl acetate/hexane = 1/100), 90% isolated yield (colorless oil, 58.0 mg). **^{1}H NMR** (400 MHz, $CDCl_3$) δ 8.48 (dd, J = 4.8, 0.8 Hz, 1 H), 7.67 (t, J = 56.3 Hz, 1 H), 7.59 (td, J = 7.8, 1.9 Hz, 1 H), 7.31 – 7.21 (m, 1 H), 7.13 (ddd, J = 7.5, 4.9, 0.9 Hz, 1 H). ppm. **^{19}F NMR** (376 MHz, $CDCl_3$) δ -96.24 (d, J = 56.4 Hz) ppm.

6.4 Preparation of palladium complex B

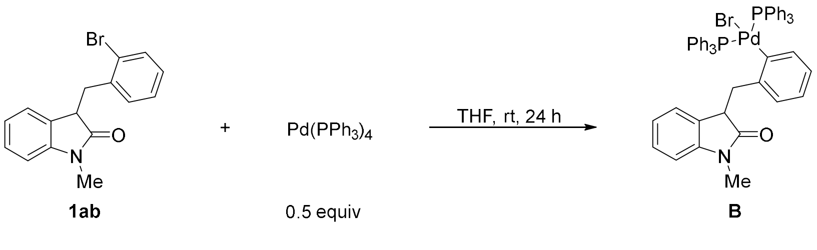

A reaction tube was charged with **1ab** (158 mg, 0.5 mmol), $Pd(PPh_3)_4$ (288.9 mg, 0.25 mmol, 0.5 equiv to **1ab**) and dry THF (4.0 mL) under N_2 atmosphere. The reaction vessel was sealed using a PTFE septum, and the mixture was stirred at rt for 24 h. After complete conversion, the resulting mixture was purified by recrystallization from hexane/THF to obtain the compound **B** (217 mg, 92% yield), and its structure was determined by HRMS, NMR and X-ray analysis.

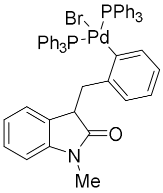

Chemical Formula: $C_{52}H_{44}BrNOP_2Pd$
Exact Mass: 945.1116

Palladium complex B: Purification by Recrystallization from hexane/THF, 92% isolated yield (yellow solid, 217.1 mg). **^{1}H NMR** (400 MHz, C_6D_6) δ 7.85 - 7.80 (m, 10H), 7.27 (d, J = 6.5 Hz, 1H), 7.16 (d, J = 1.4 Hz, 1H), 7.01 - 6.97 (m, 20H), 6.82 (d, J = 7.1 Hz, 1H), 6.77 (t, J = 7.3 Hz, 1H), 6.60 (t, J = 7.1 Hz, 1H), 6.52 (d, J = 7.1 Hz, 1H), 6.32 (d, J = 7.1 Hz, 1H), 6.25 (d, J = 7.7 Hz, 1H), 3.87 (dd, J = 13.9, 3.9 Hz, 1H), 3.58 (dd, J = 10.0, 3.7 Hz, 1H), 2.73 (s, 3H), 2.43 (dd, J = 13.9, 10.3 Hz, 1H) ppm. **^{31}P{H} NMR** (162 MHz, C_6D_6) δ 23.93 (d, J = 42.4 Hz) ppm. HRMS (ESI) calculated for $C_{52}H_{44}NOP_2Pd$ $[M-Br]^+$: 866.1951, found: 866.1956.

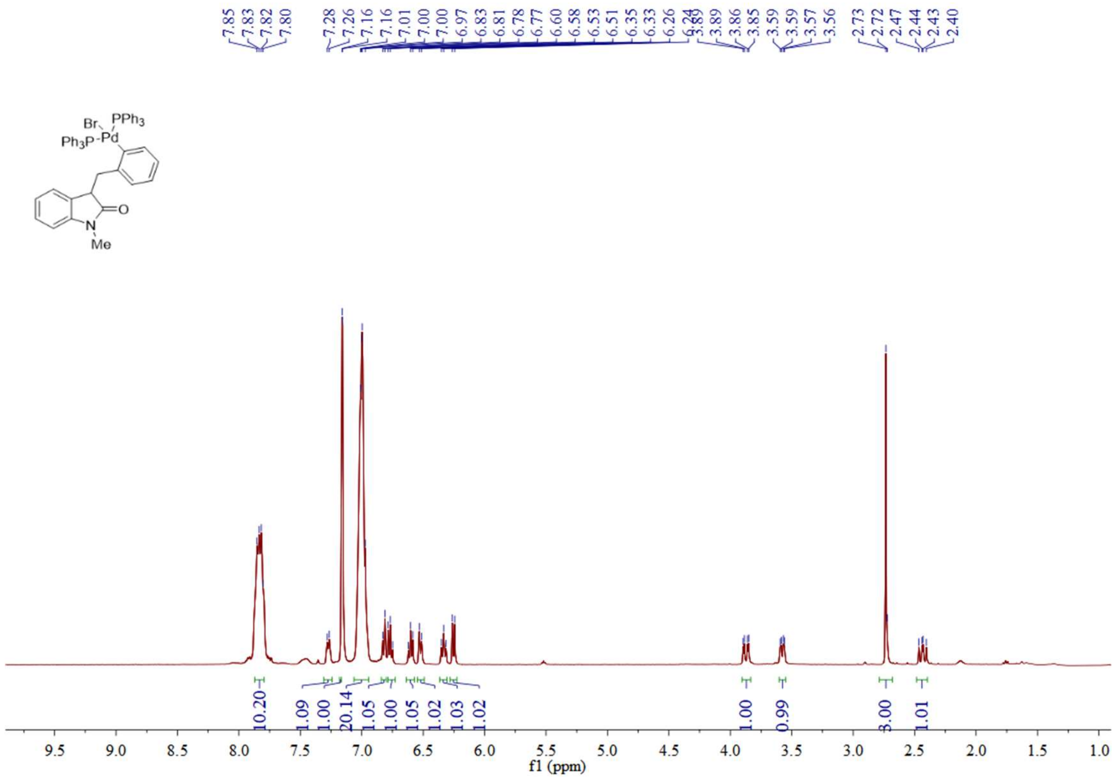

Supplementary Figure 2. The ^{1}**H NMR** spectra (400 MHz, $CDCl_3$) of **B**.

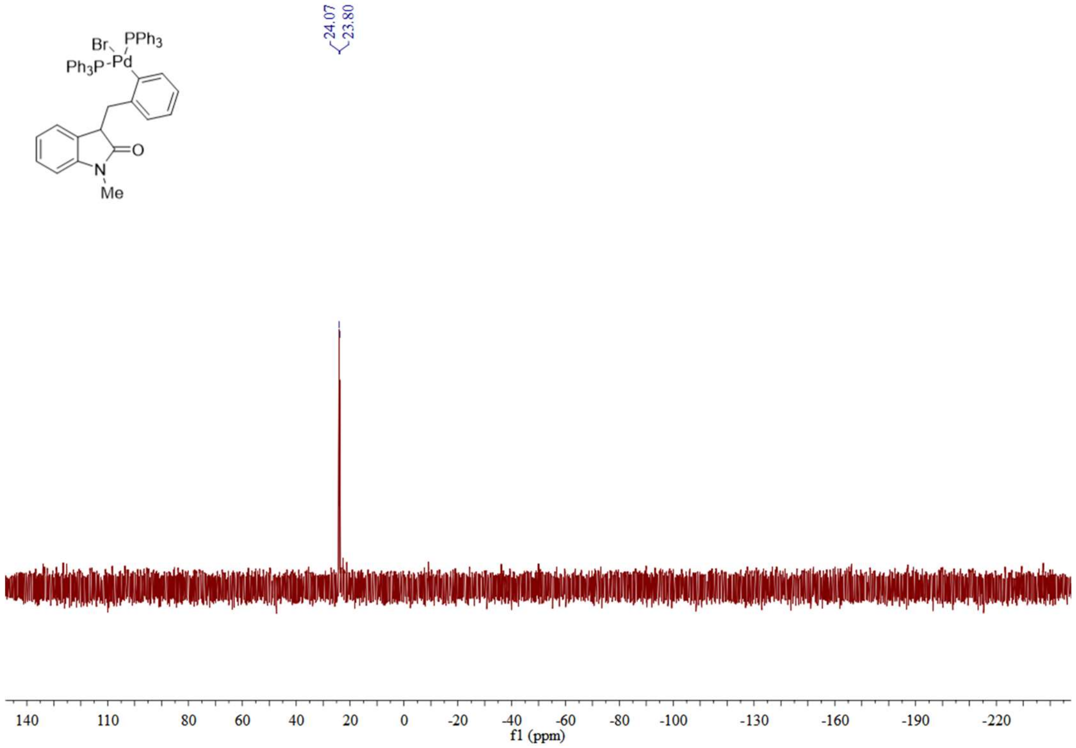

Supplementary Figure 3. The ^{31}**P{H} NMR** spectra (162 MHz, $CDCl_3$) of **B**.

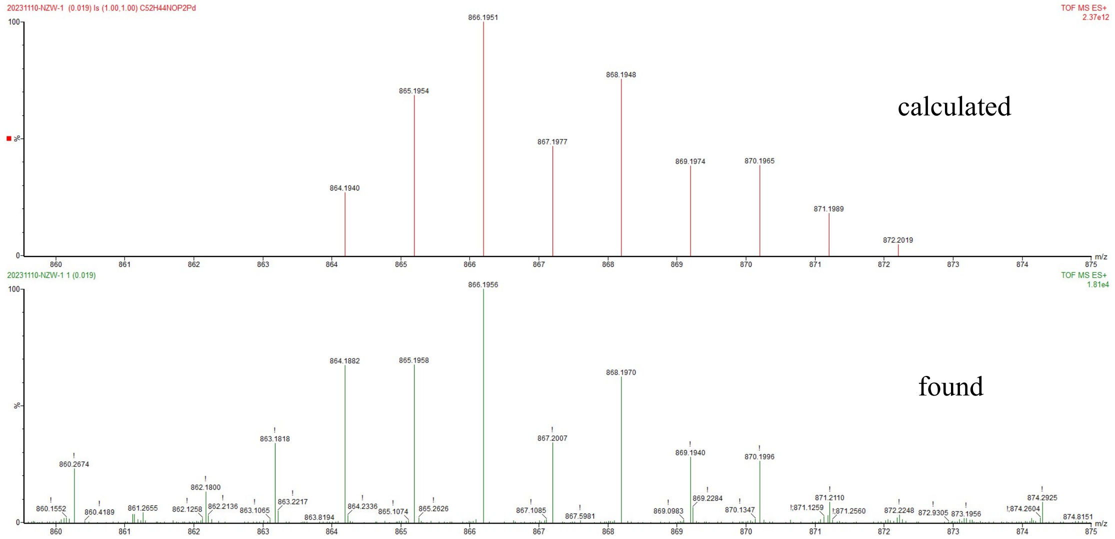

Supplementary Figure 4. HRMS (ESI) spectra of **B**.

6.5 Preparation, characterization, and transformations of key intermediate C

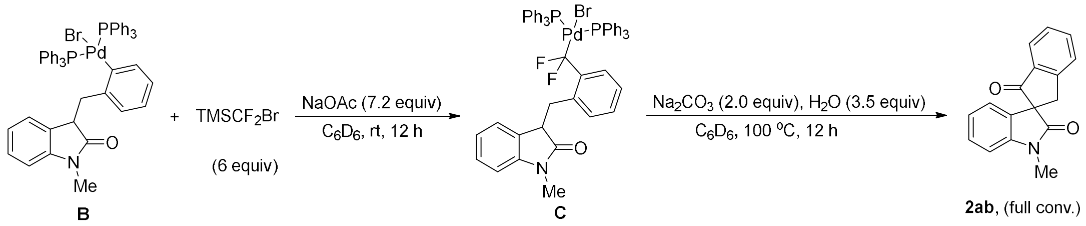

Step 1:

In an argon-filled glove box, the palladium complex **B** (47 mg, 0.05 mmol), $TMSCF_2Br$ (61 mg, 0.3 mmol), NaOAc (30 mg, 0.36 mmol), and anhydrous C_6D_6 (1.0 mL) were added into a Schlenk tube. The Schlenk tube was sealed using a PTFE septum, and the mixture was stirred at rt for 12 h. After that, the reaction mixture was filtered and the filtrate was analyzed by HRMS, ^{19}F NMR and ^{31}P NMR. According to ^{19}F NMR analysis, the major peak at -67.92 ppm (d, J = 60.0 Hz) was similar to that of reported analogues of $ArCF_2$-$Pd(PPh_3)_2$-Br.[6-8] However, several other minor F-containing species were also observed (Supplementary Figure 5). According to $^{31}P\{H\}$ NMR analysis, a major peak at 18.93 ppm (br) was observed, while a minor peak closed to that of complex **B** was also observed (Supplementary Figure 6). The Attempts to purify and obtain the X-ray structure of **C** were so far unsuccessful.

To gain more information about the reaction outcome, we submitted the reaction mixture to HRMS analysis, which indicated the possible presence of the intermediate

C.

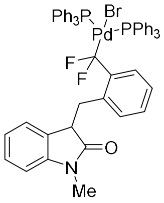

Tentative characterization of Intermediate C: ^{19}F{H} NMR (376 MHz, C_6D_6) -67.92 (d, *J* = 60.0 Hz, 2 F) ppm. **^{31}P{H} NMR** (162 MHz, C_6D_6) δ 18.93 (br) ppm. **HRMS** (ESI) calculated for $[M+H]^+$ $C_{53}H_{45}BrF_2NOP_2Pd^+$: 998.1168, found: 998.1155.

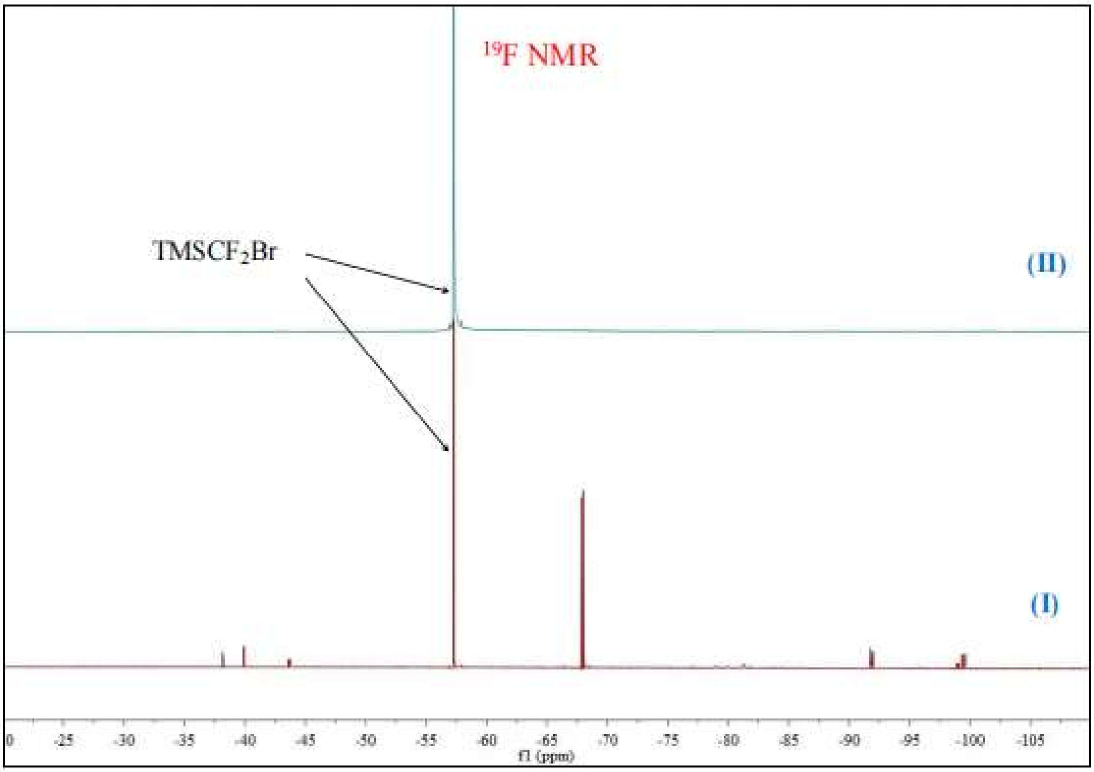

Supplementary Figure 5. The **^{19}F{H} NMR** (376 MHz, C_6D_6) spectra associated with reaction of complex **B** with $TMSCF_2Br$ in C_6D_6. (I) $TMSCF_2Br$; (II) The reaction was stirred at rt for 12 h.

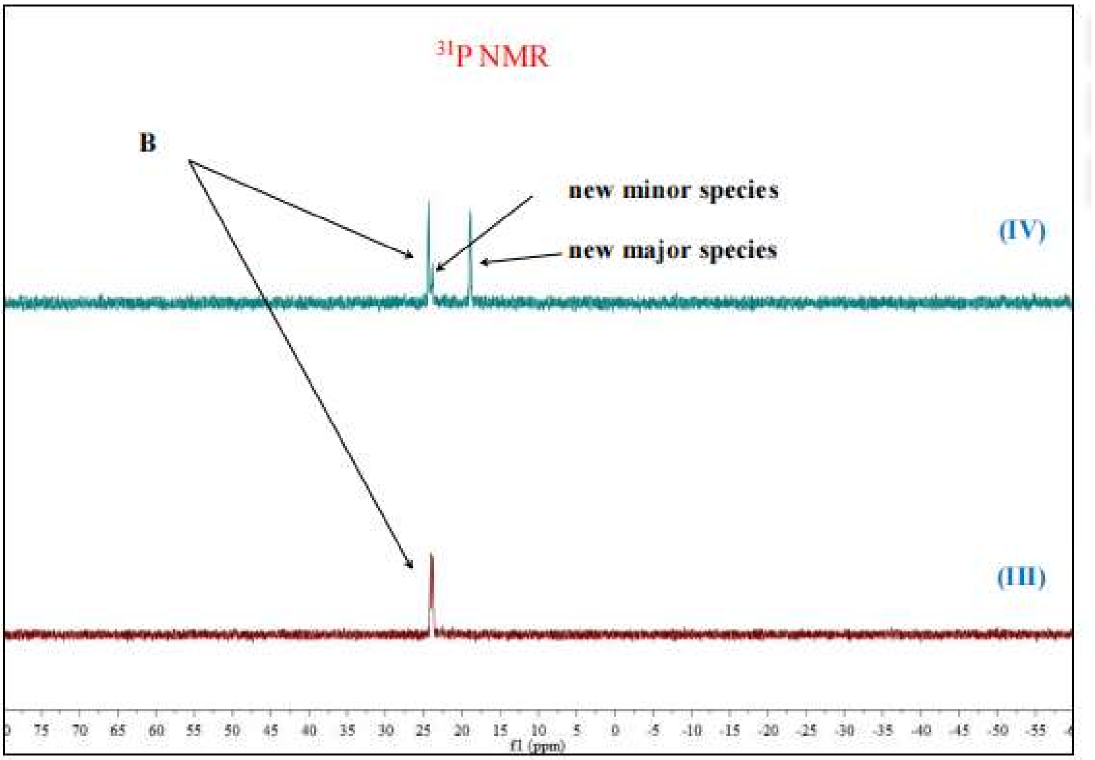

Supplementary Figure 6. The **^{31}P{H} NMR** (162 MHz, C_6D_6) spectra associated with reaction of complex **B** with $TMSCF_2Br$ in C_6D_6. (III) Complex **B**; (IV) Reaction mixture after stirred at rt for 12 h.

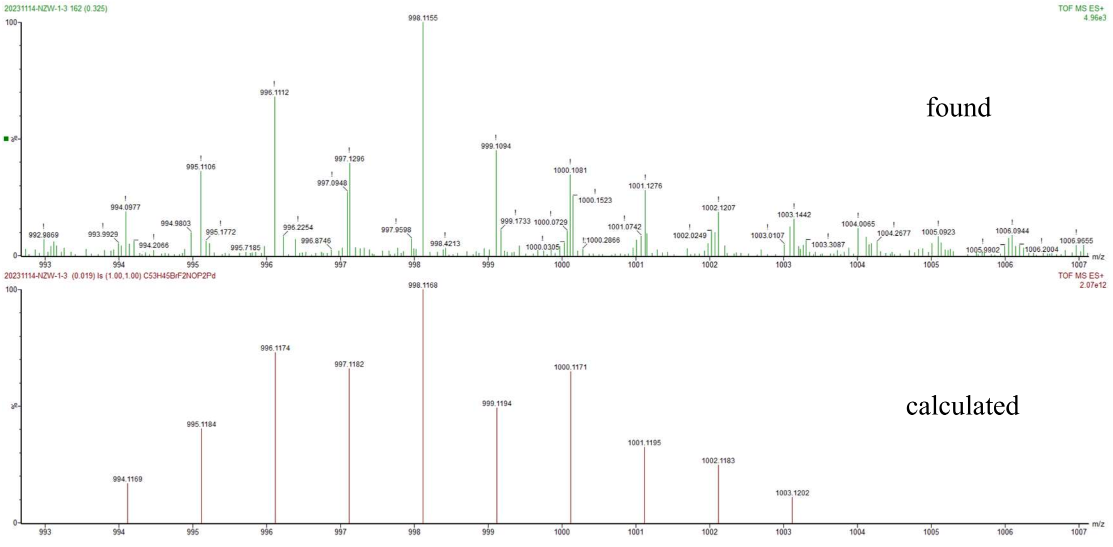

Supplementary Figure 7. The HRMS (ESI) spectra of **C**.

Step 2:

In an argon-filled glove box, the filtrate obtained at the last step was transferred to a Schlenk tube. Subsequently, Na_2CO_3 (10.6 mg, 0.1 mmol) and water (3.2 mg, 0.175 mmol) were added into the Schlenk tube. The Schlenk tube was sealed using a PTFE

septum, and the mixture was stirred at 100 °C for 12 h. After that, the resulting mixture was cooled to rt, and subjected to GC-MS analysis. The results showed that the intermediate **C** was completely converted into compound **2ab**.

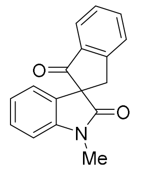

Chemical Formula: $C_{17}H_{13}NO_2$
Exact Mass: 263.0946

1'-methylspiro[indene-2,3'-indoline]-1,2'(3H)-dione (2ab). Purification by column chromatography (0.2 mmol scale, eluent: ethyl acetate/hexane = 1/8), 98% isolated yield (white solid, 51.8 mg). **^{1}H NMR** (400 MHz, $CDCl_3$) δ 7.80 (d, *J* = 7.7 Hz, 1 H), 7.69 (td, *J* = 7.5, 1.2 Hz, 1 H), 7.60 (d, *J* = 7.7 Hz, 1 H), 7.45 (t, *J* = 7.4 Hz, 1 H), 7.31 (td, *J* = 7.7, 1.3 Hz, 1 H), 6.99 (t, *J* = 7.5 Hz, 1 H), 6.91 (t, *J* = 8.6 Hz, 2 H), 3.82 (ab, *J* = 17.2 Hz, 1 H), 3.44 (ab, *J* = 17.2 Hz, 1 H), 3.28 (s, 3 H) ppm. **^{13}C {H} NMR** (100 MHz, $CDCl_3$) δ 200.0, 175.0, 153.9, 145.0, 135.8, 135.2, 130.3, 129.0, 128.3, 126.7, 125.6, 123.1, 122.2, 108.8, 63.1, 37.7, 26.9 ppm. HRMS (ESI) calculated for $[M+H]^+$ $C_{17}H_{14}NO_2^+$: 264.1019, found: 264.1018.

Supplementary Notes

7. NMR spectra

^{1}H NMR of **1a**, 400 MHz, $CDCl_3$

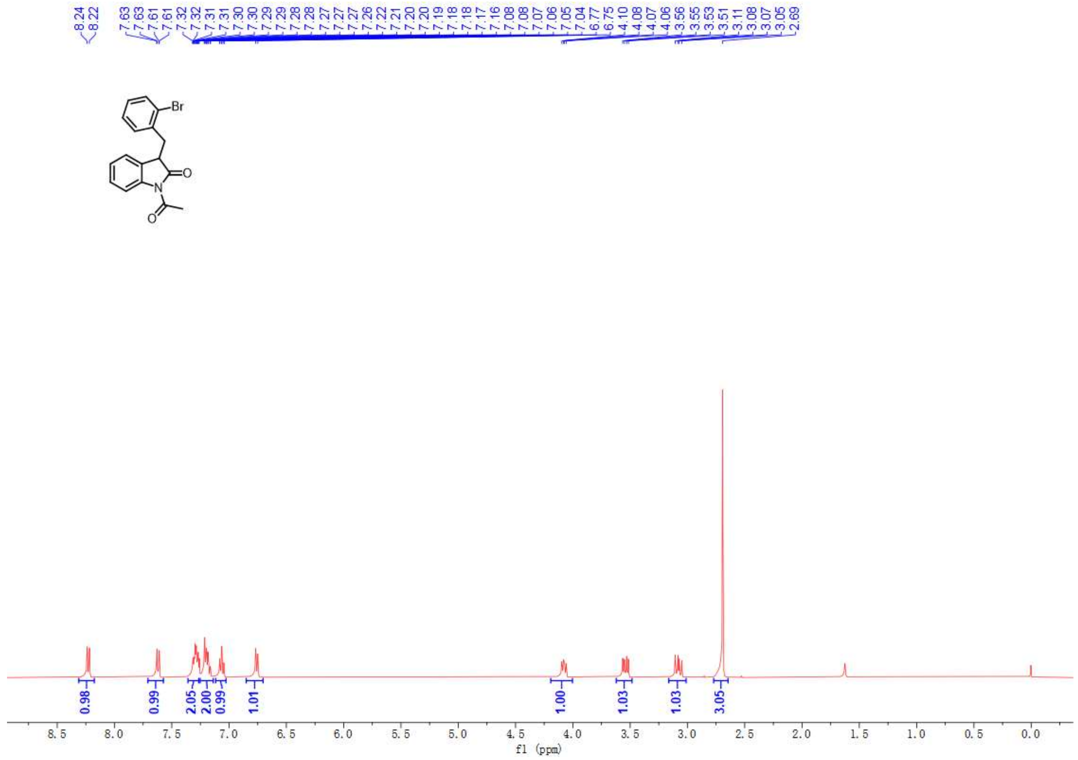

^{13}C {^{1}H} NMR of **1a**, 100 MHz, $CDCl_3$

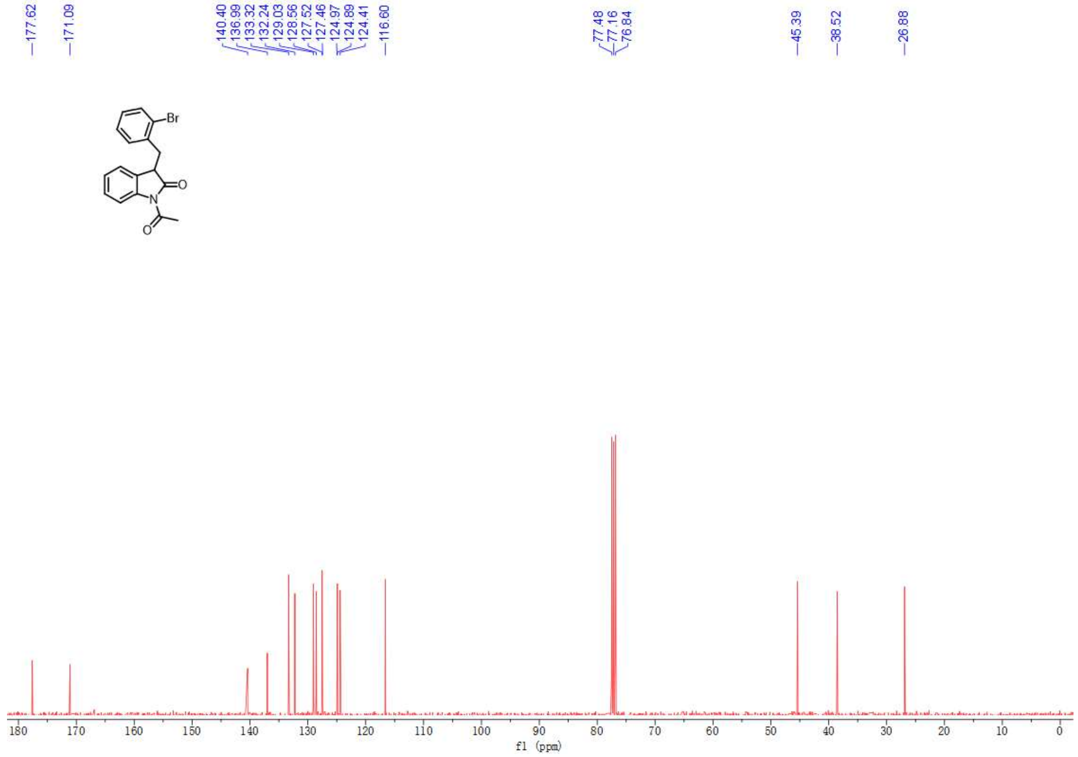

^{1}H NMR of **1b**, 400 MHz, $CDCl_3$

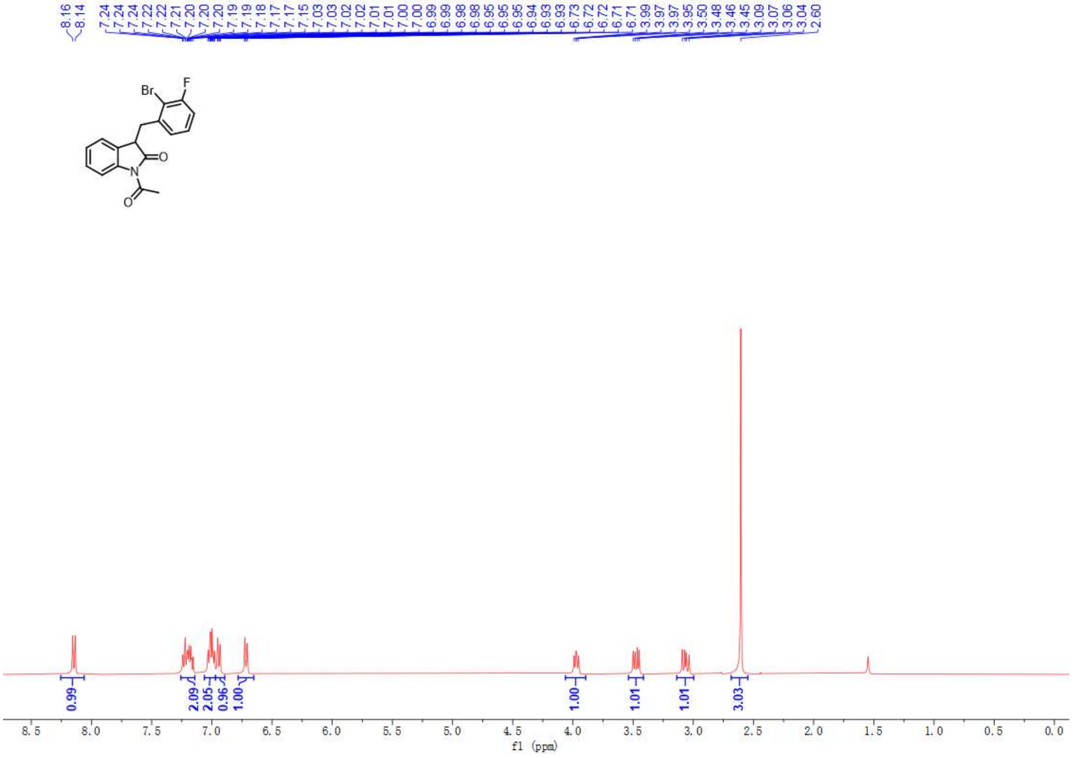

^{13}C {^{1}H} NMR of **1b**, 100 MHz, $CDCl_3$

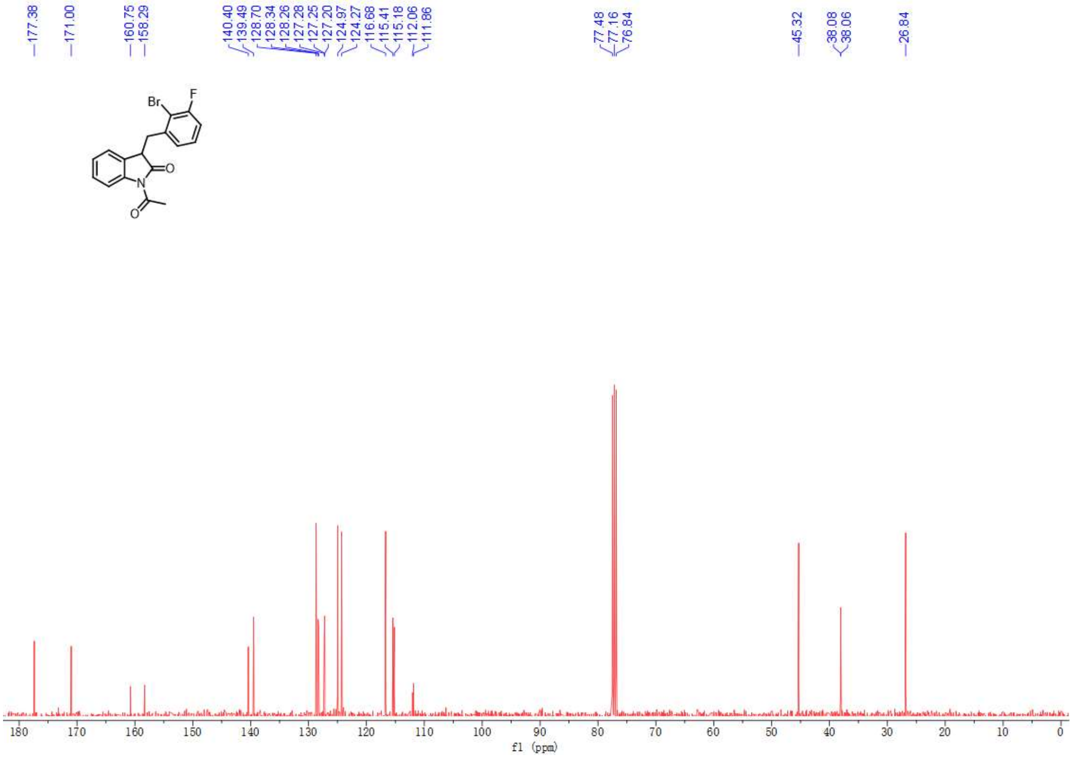

^{19}F NMR of **1b**, 376 MHz, $CDCl_3$

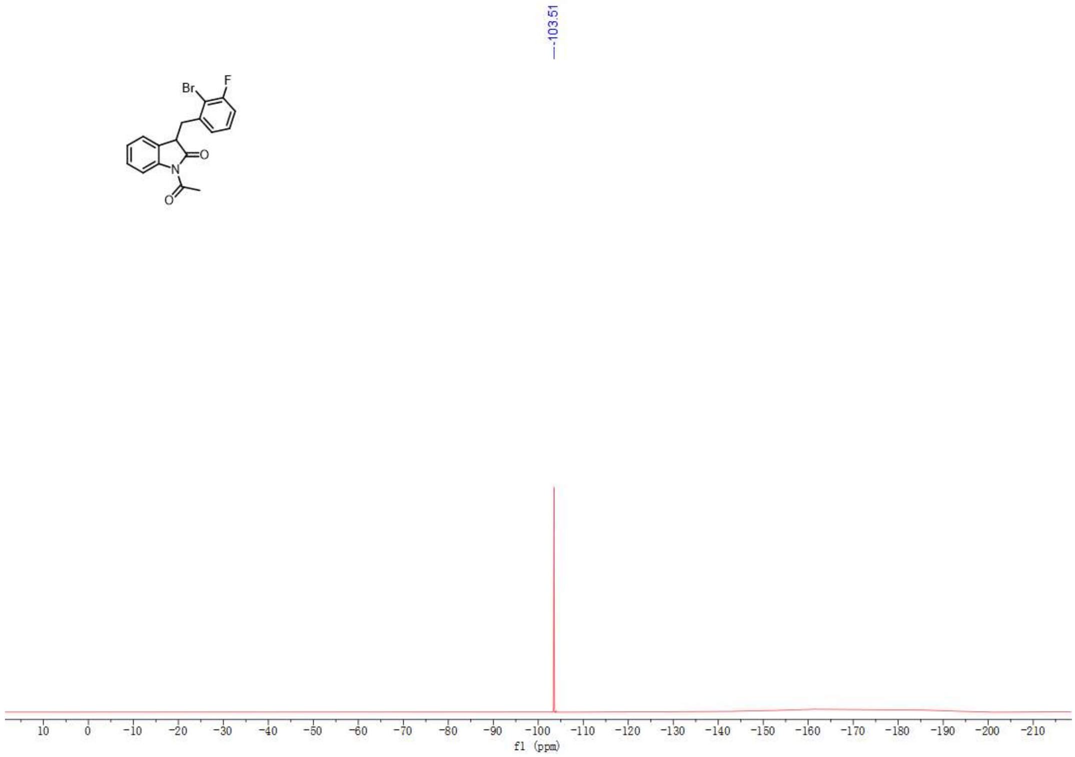

^{1}H NMR of **1c**, 400 MHz, $CDCl_3$

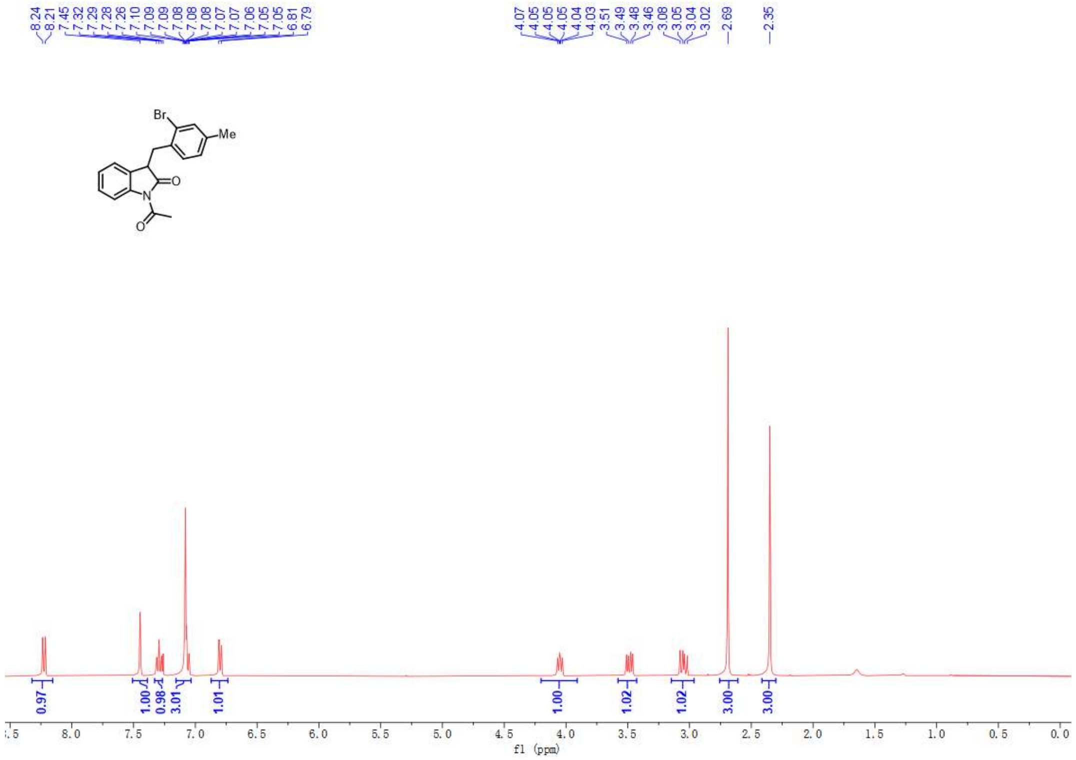

^{13}C {^{1}H} NMR of **1c**, 100 MHz, $CDCl_3$

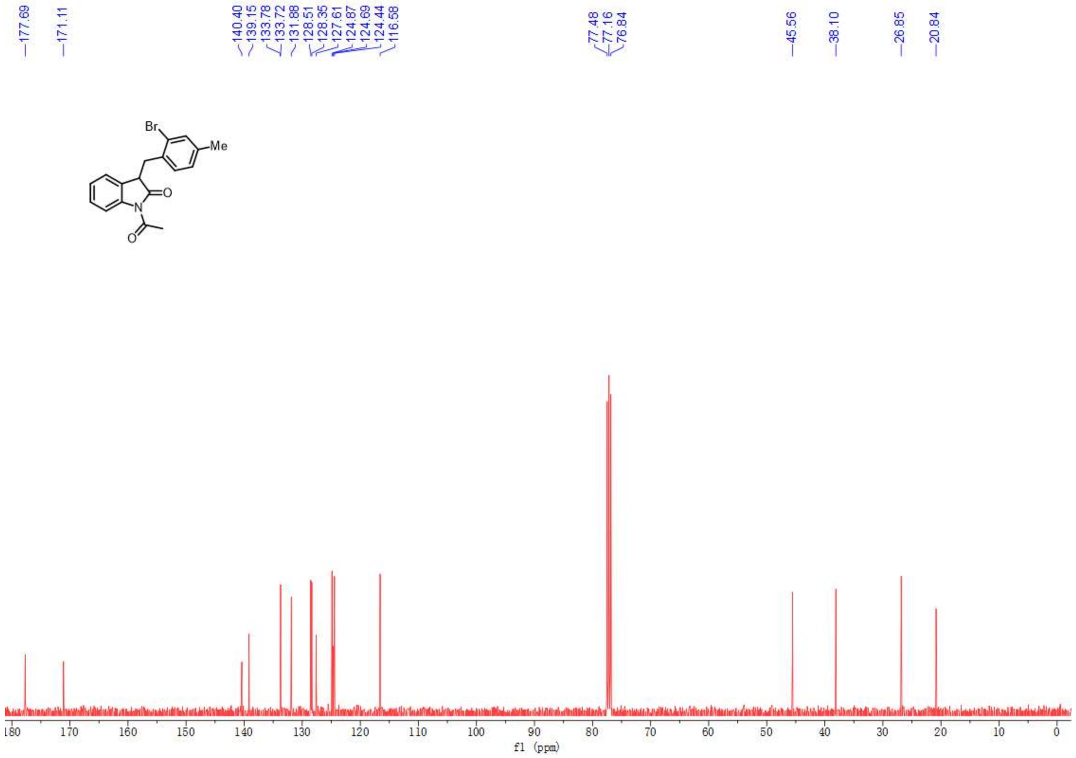

^{1}H NMR of **1d**, 400 MHz, $CDCl_3$

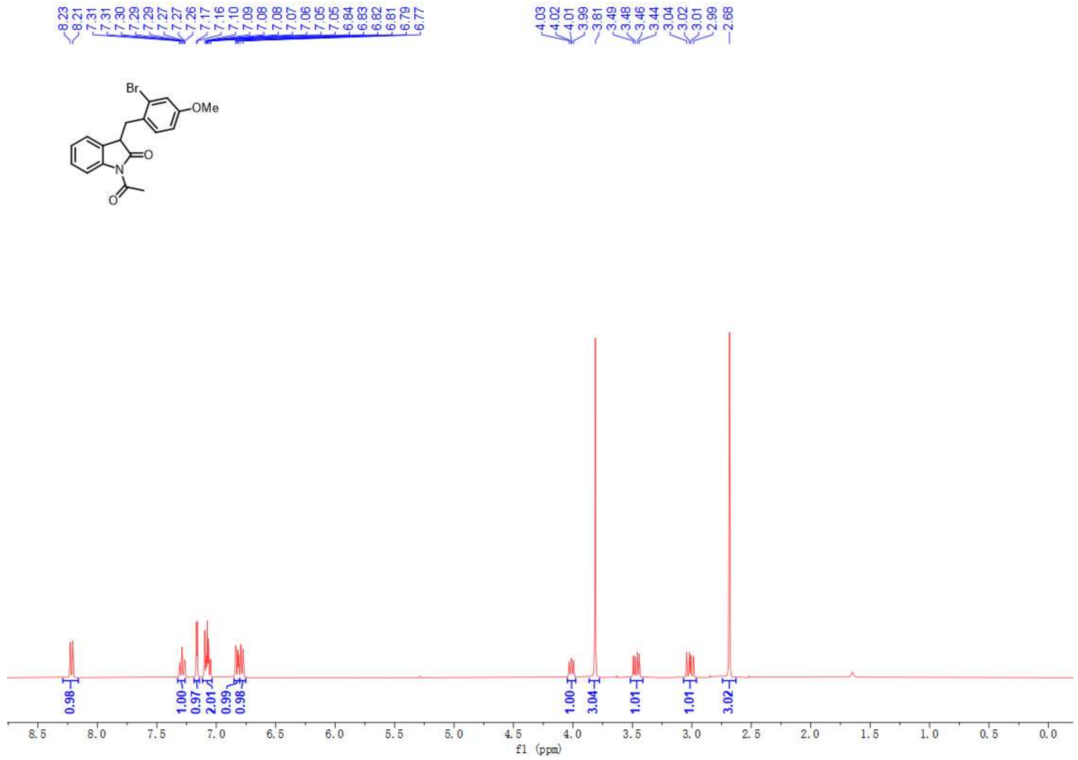

^{13}C {^{1}H} NMR of **1d**, 100 MHz, $CDCl_3$

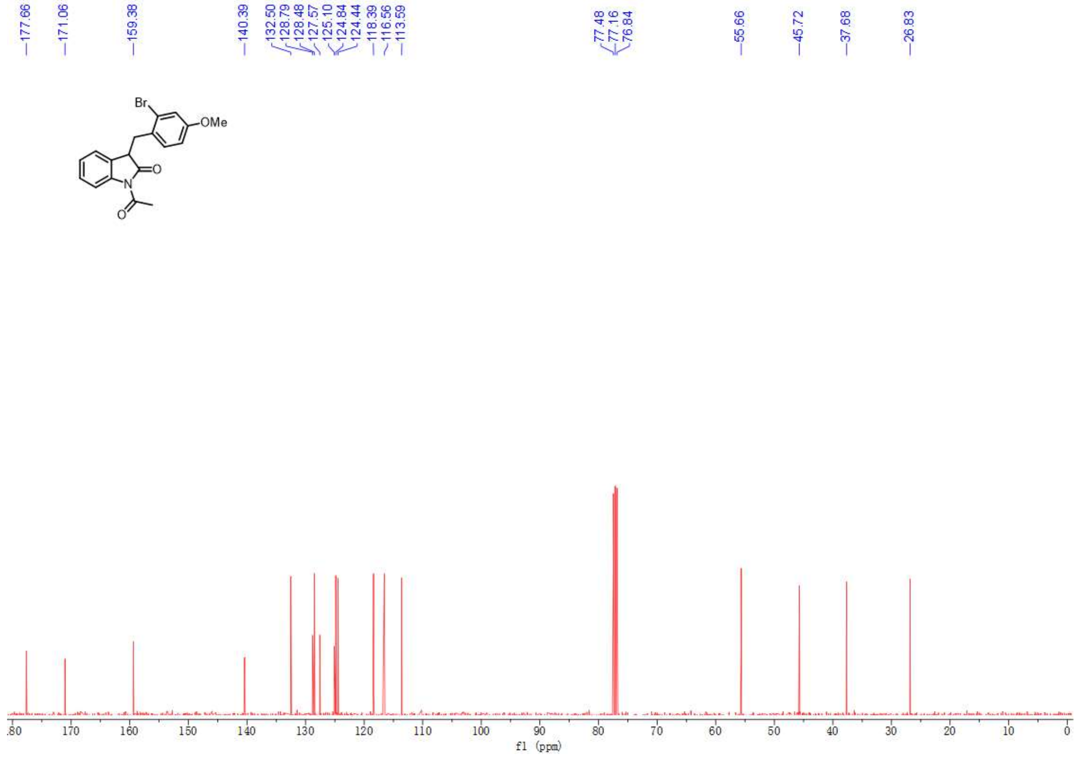

^{1}H NMR of **1e**, 400 MHz, $CDCl_3$

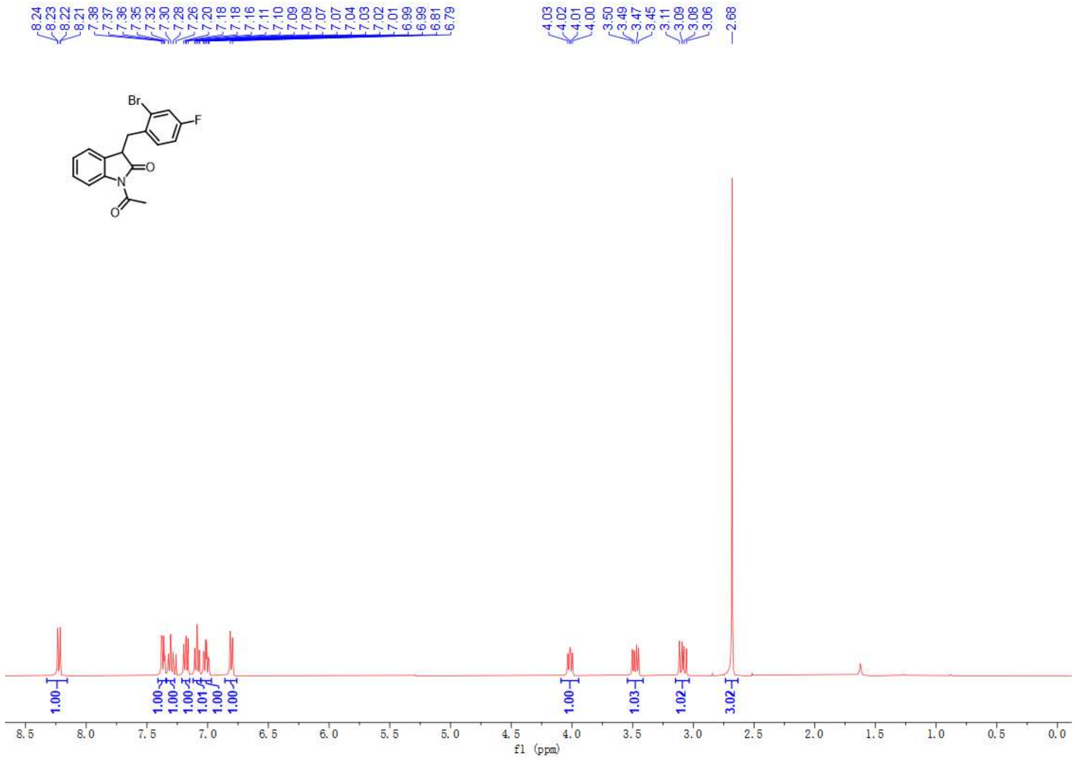

^{13}C {^{1}H} NMR of **1e**, 100 MHz, $CDCl_3$

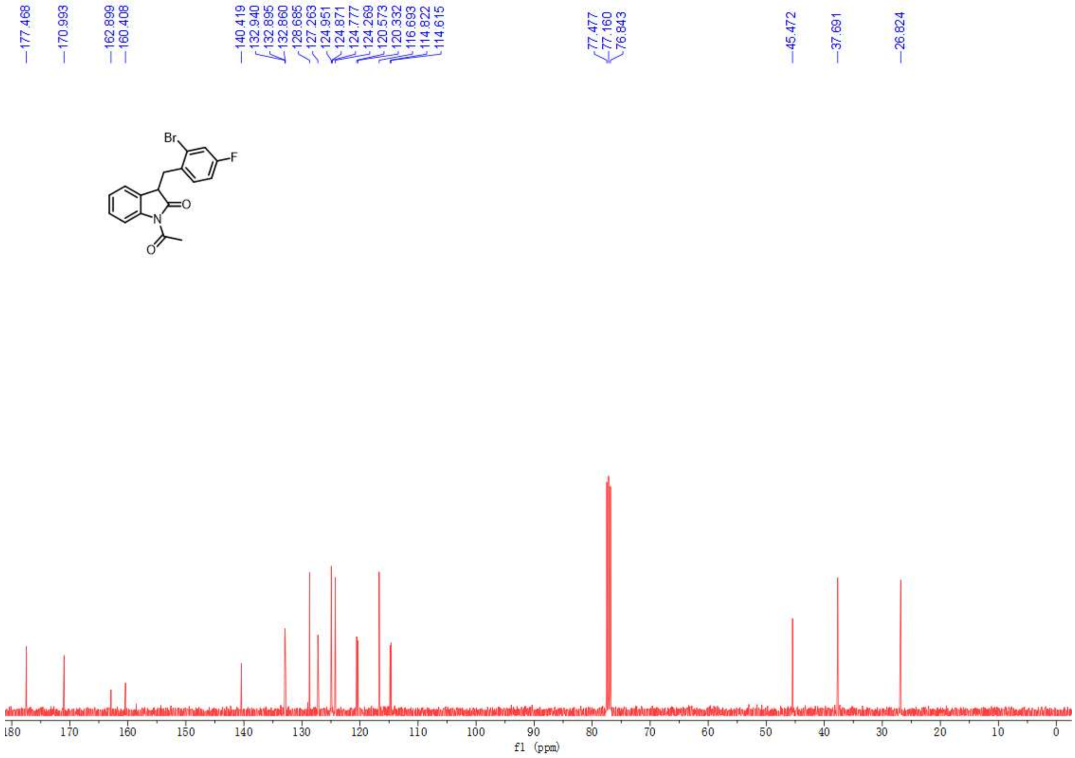

^{19}**F NMR** of **1e**, 376 MHz, $CDCl_3$

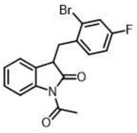

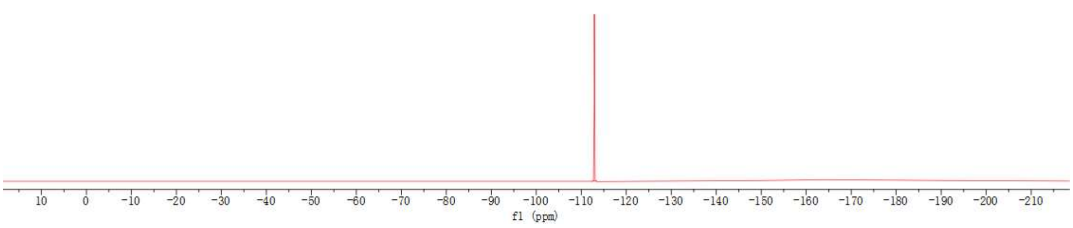

^{1}H NMR of **1f**, 400 MHz, $CDCl_3$

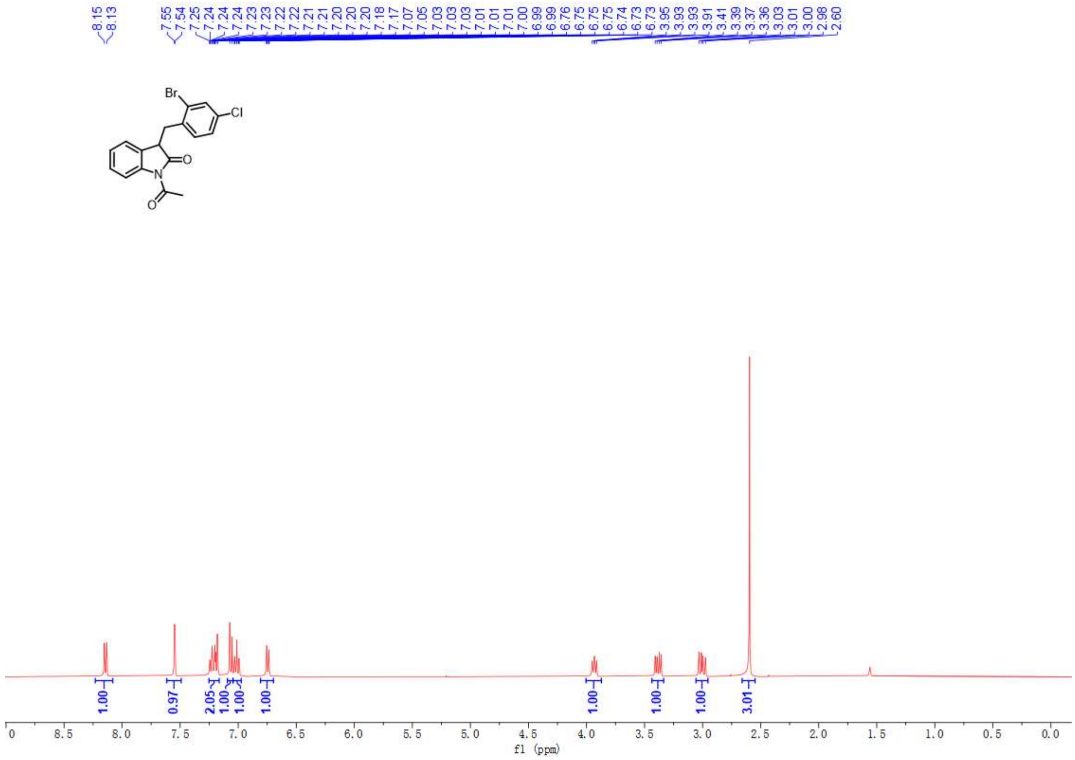

^{13}C {^{1}H} NMR of **1f**, 100 MHz, $CDCl_3$

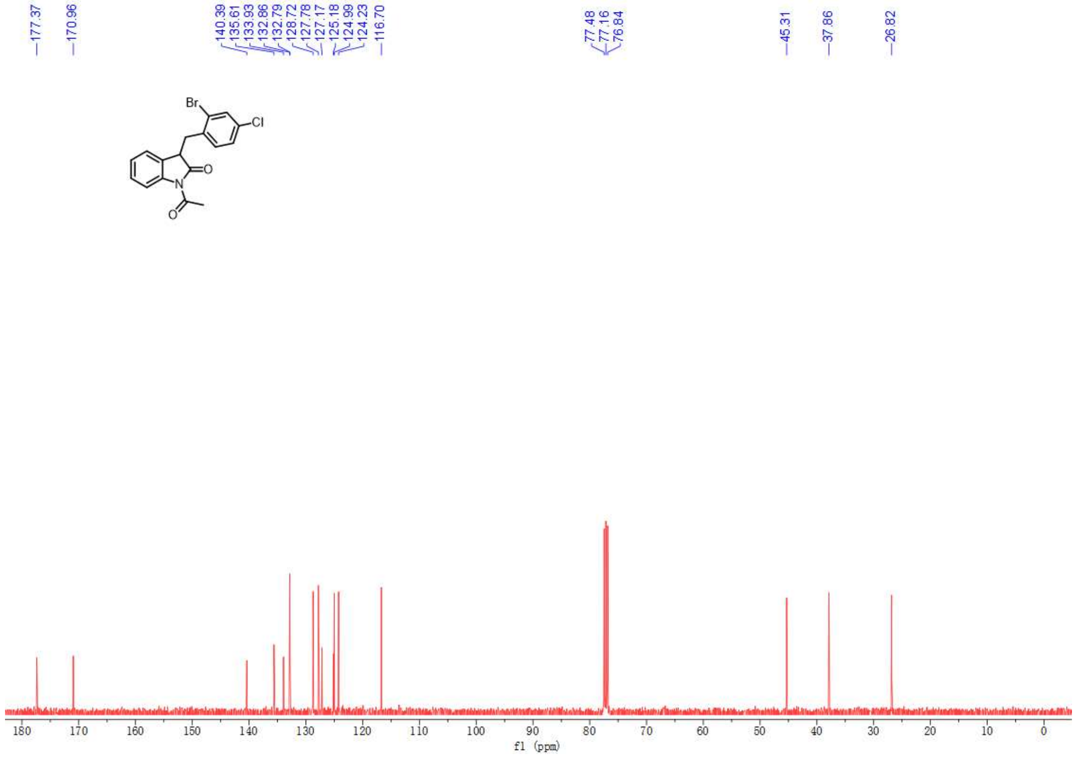

^{1}H NMR of **1g**, 400 MHz, $CDCl_3$

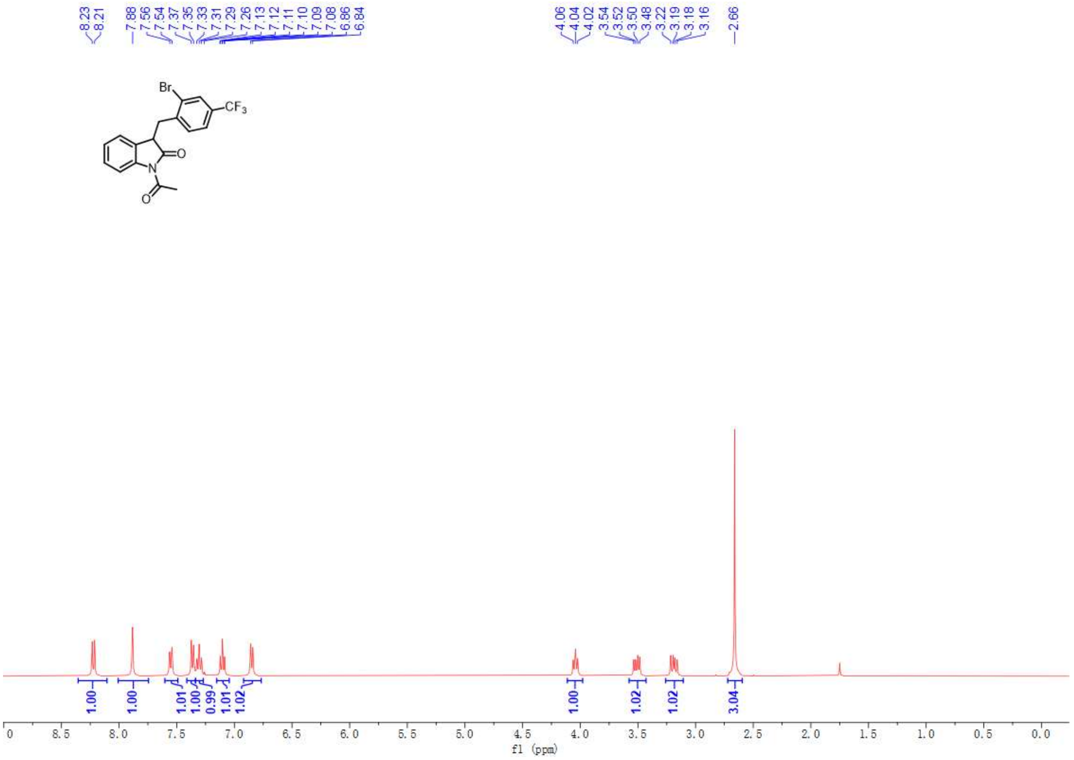

^{13}C {^{1}H} NMR of **1g**, 100 MHz, $CDCl_3$

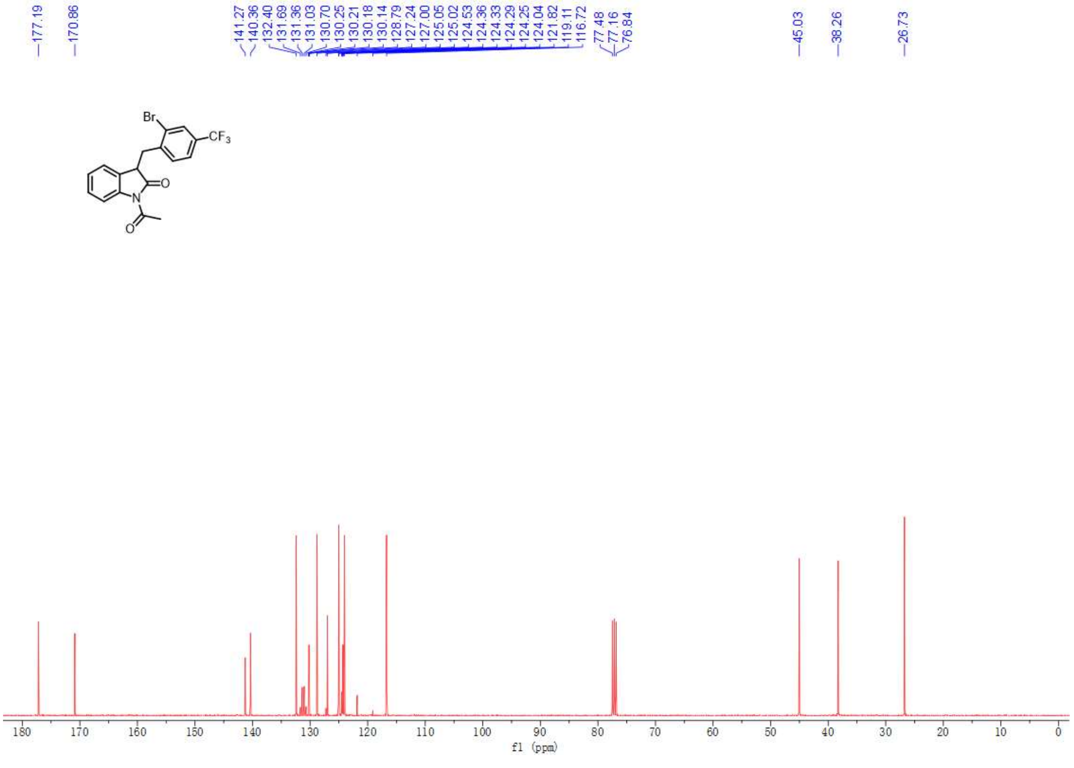

^{19}F NMR of **1g**, 376 MHz, $CDCl_3$

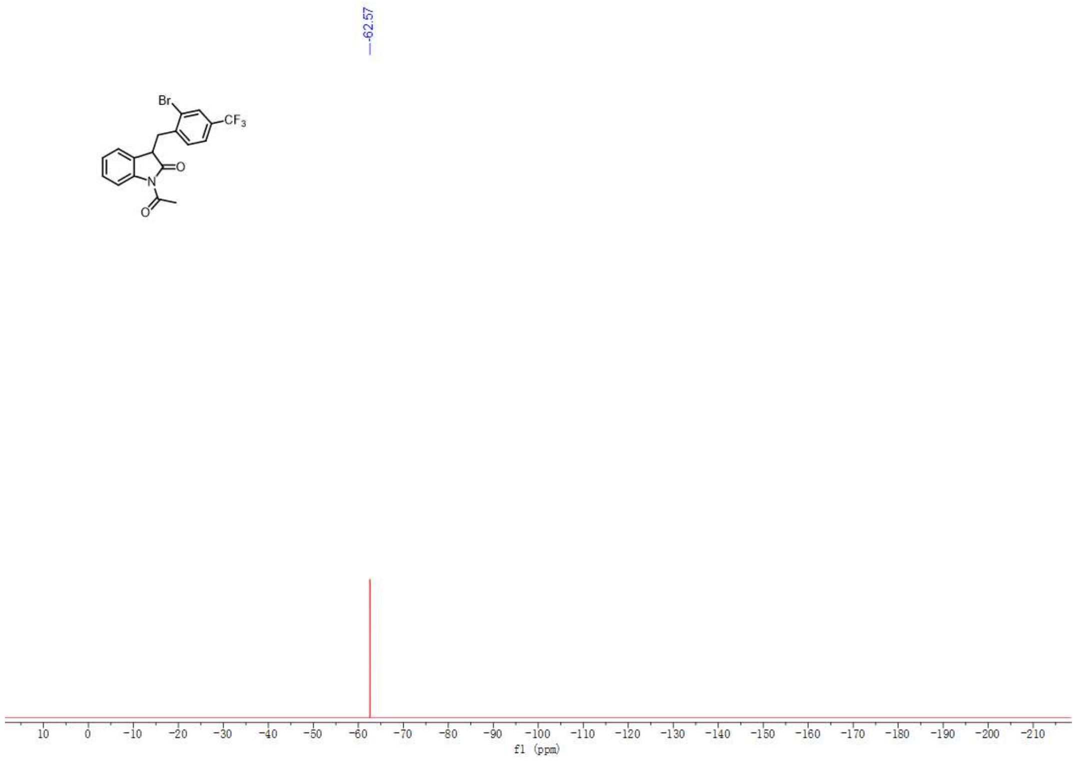

^{1}H NMR of **1h**, 400 MHz, $CDCl_3$

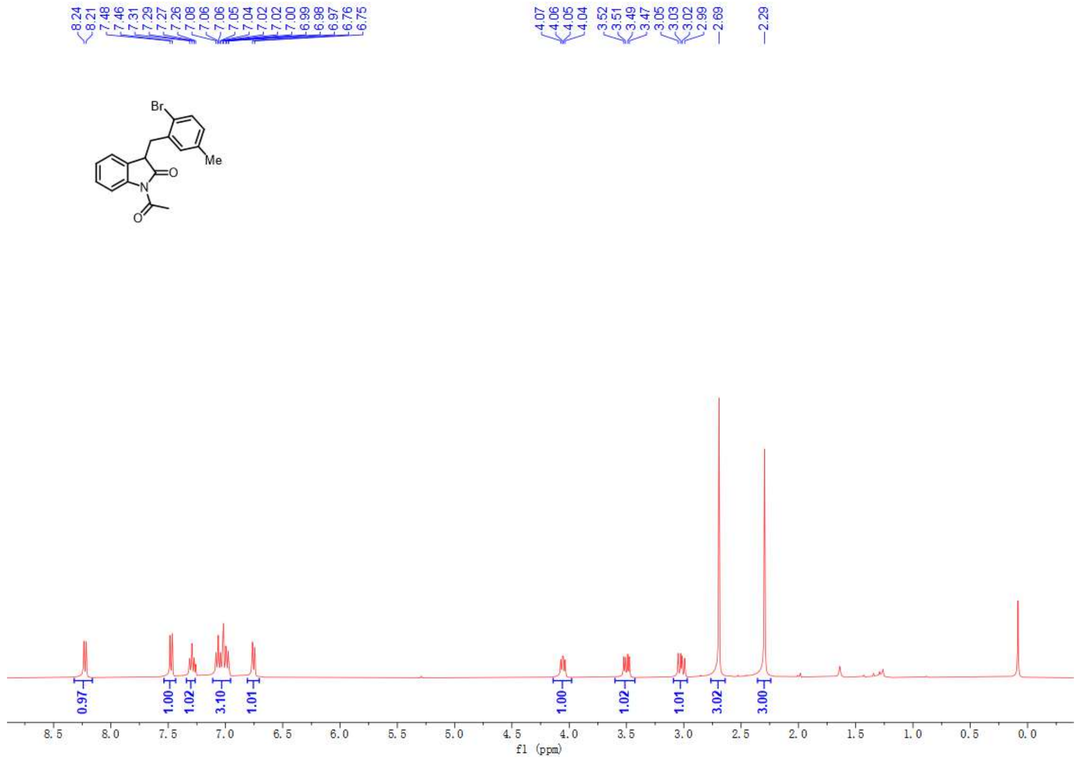

^{13}C {^{1}H} NMR of **1h**, 100 MHz, $CDCl_3$

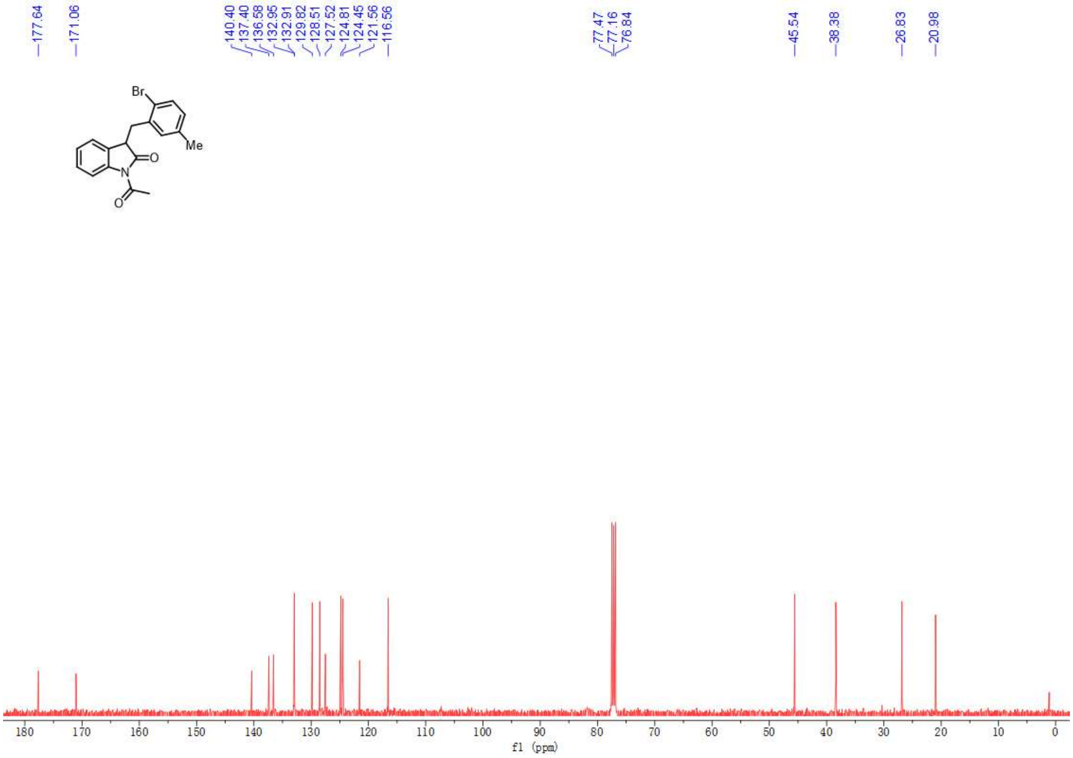

^{1}H NMR of **1i**, 400 MHz, $CDCl_3$

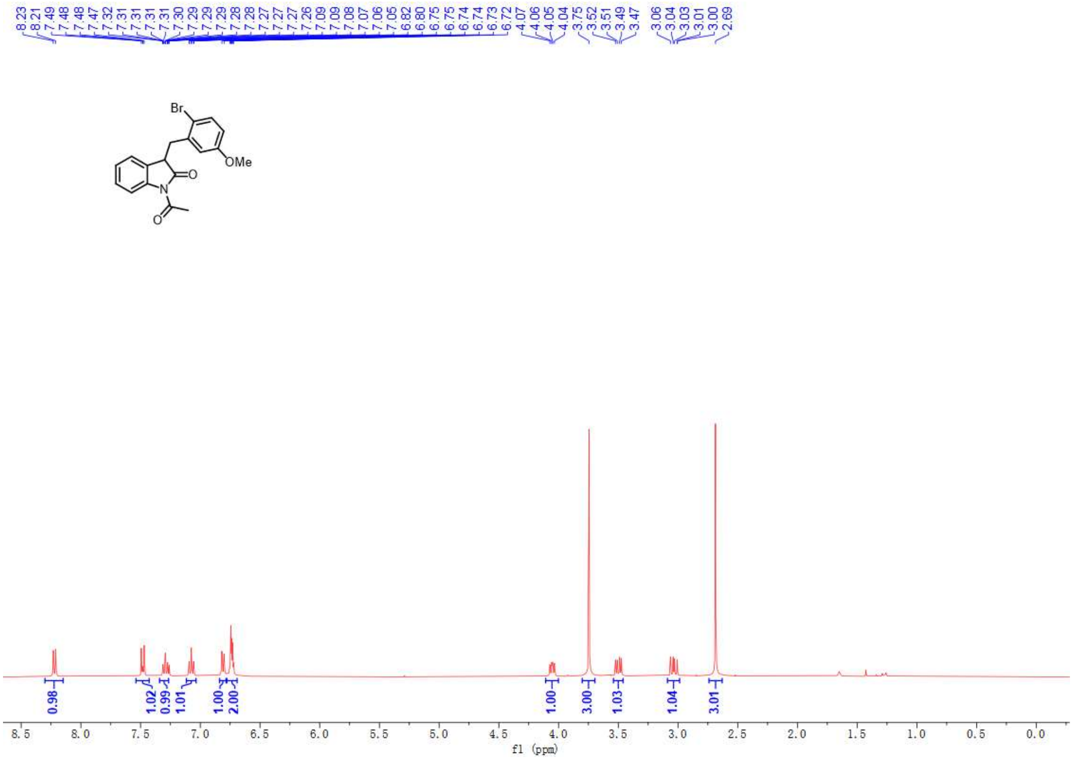

^{13}C {^{1}H} NMR of **1i**, 100 MHz, $CDCl_3$

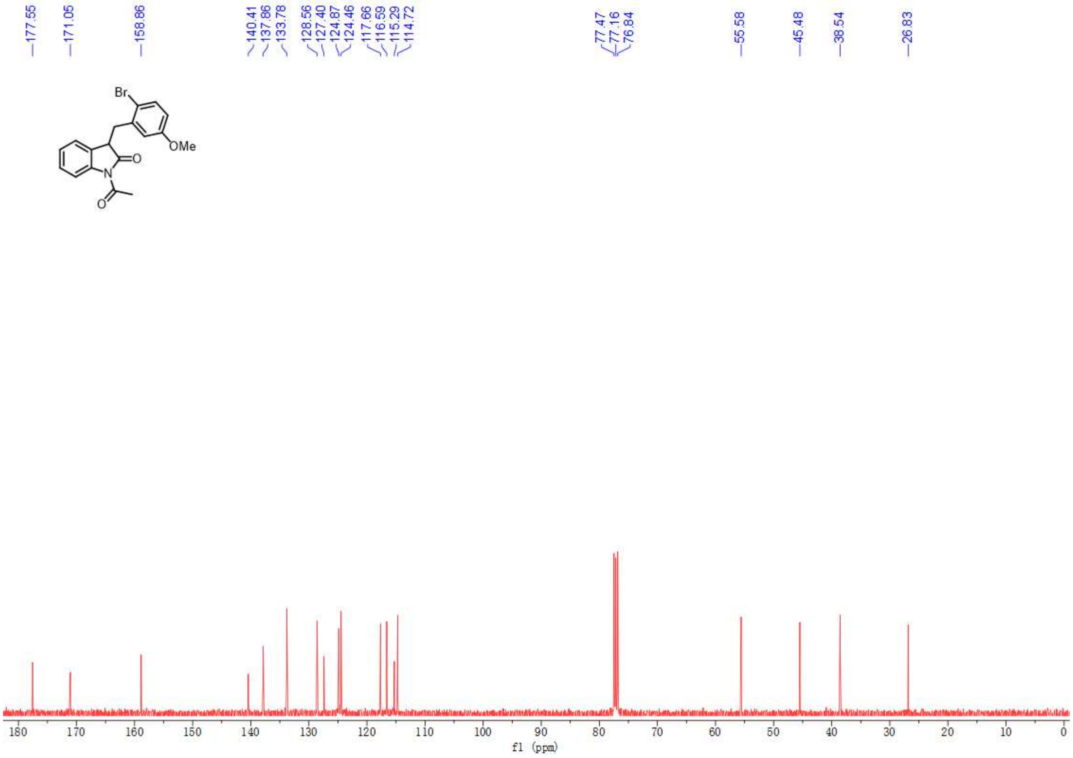

^{1}H NMR of **1j**, 400 MHz, $CDCl_3$

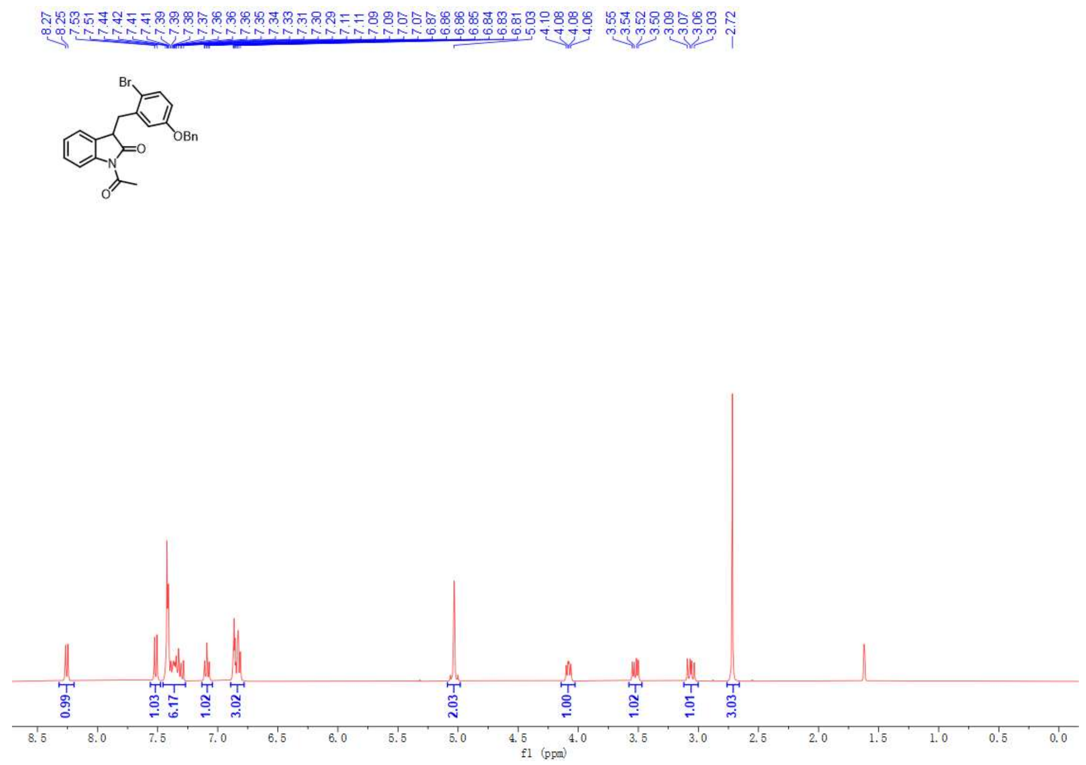

^{13}C {^{1}H} NMR of **1j**, 100 MHz, $CDCl_3$

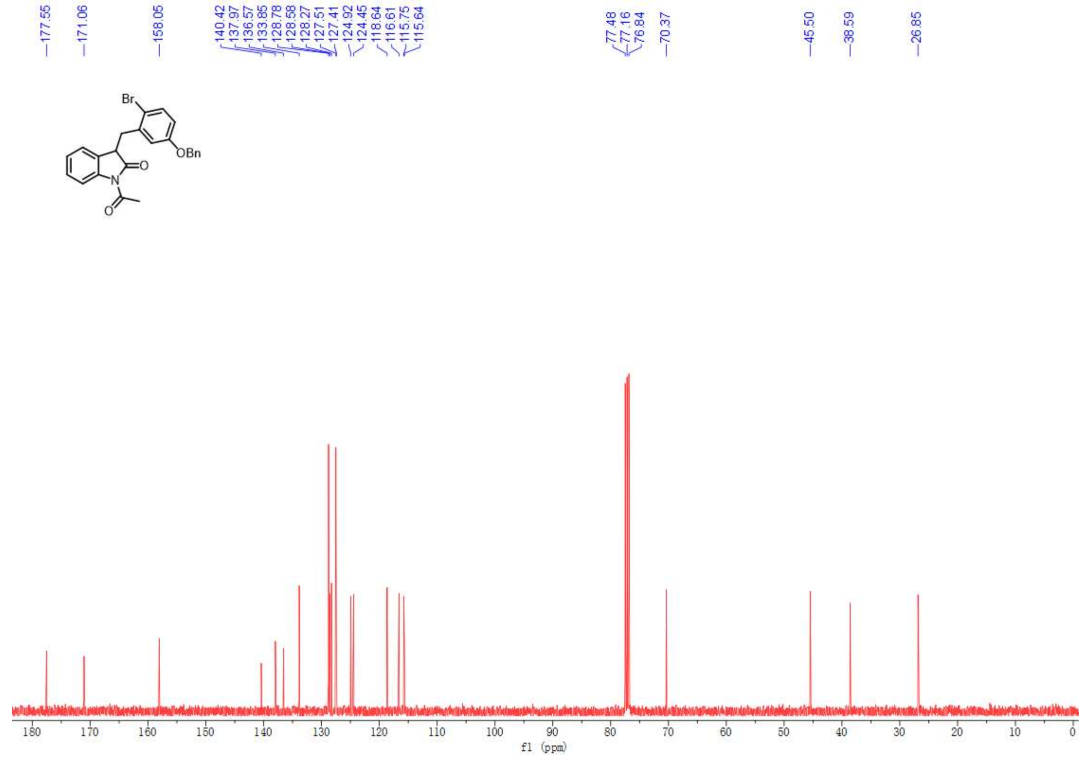

^{1}H NMR of **1k**, 400 MHz, $CDCl_3$

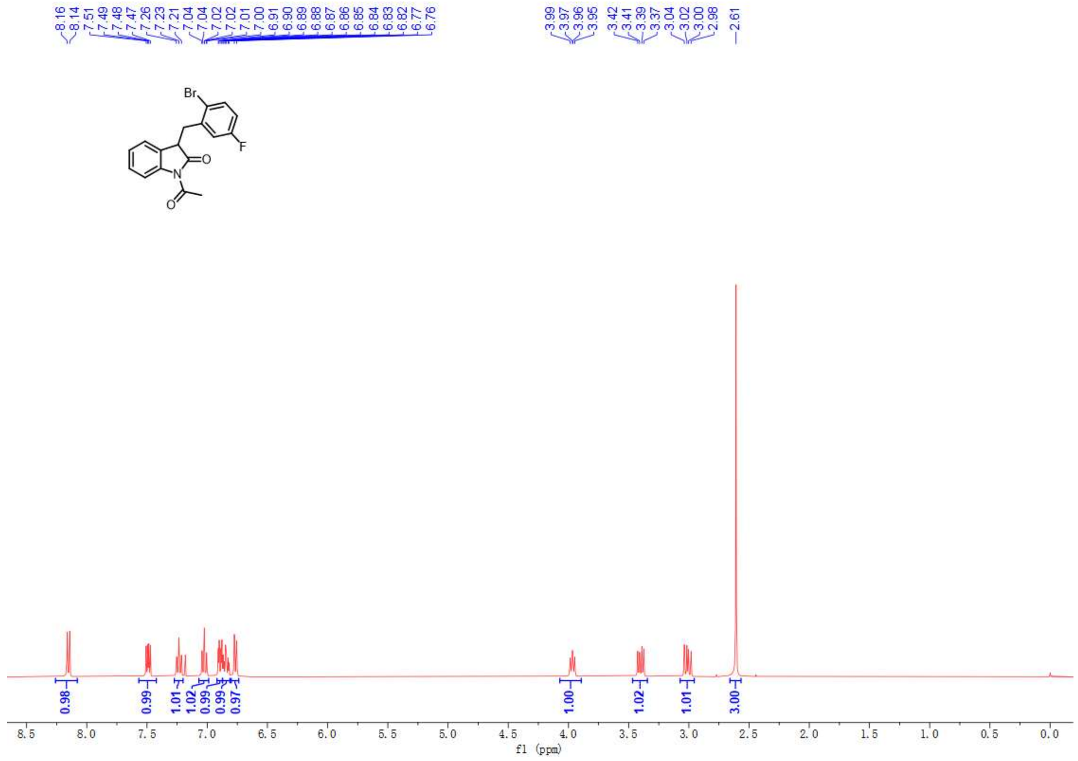

^{13}C {^{1}H} NMR of **1k**, 100 MHz, $CDCl_3$

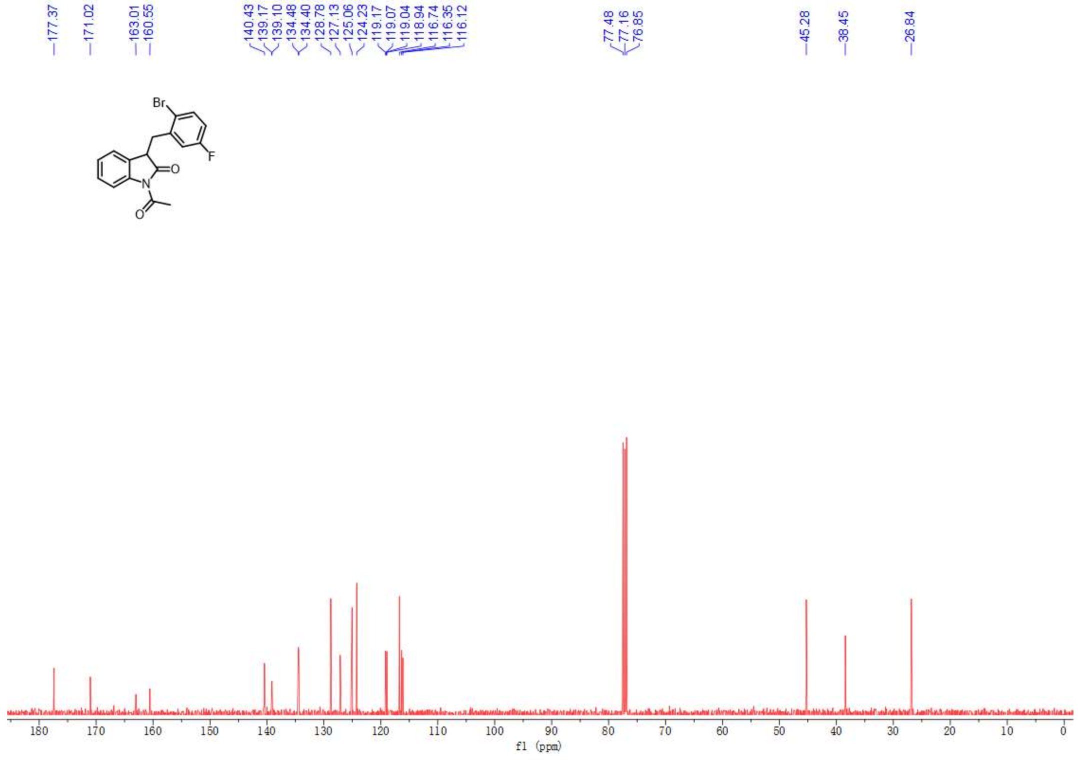

^{19}F NMR of **1k**, 376 MHz, $CDCl_3$

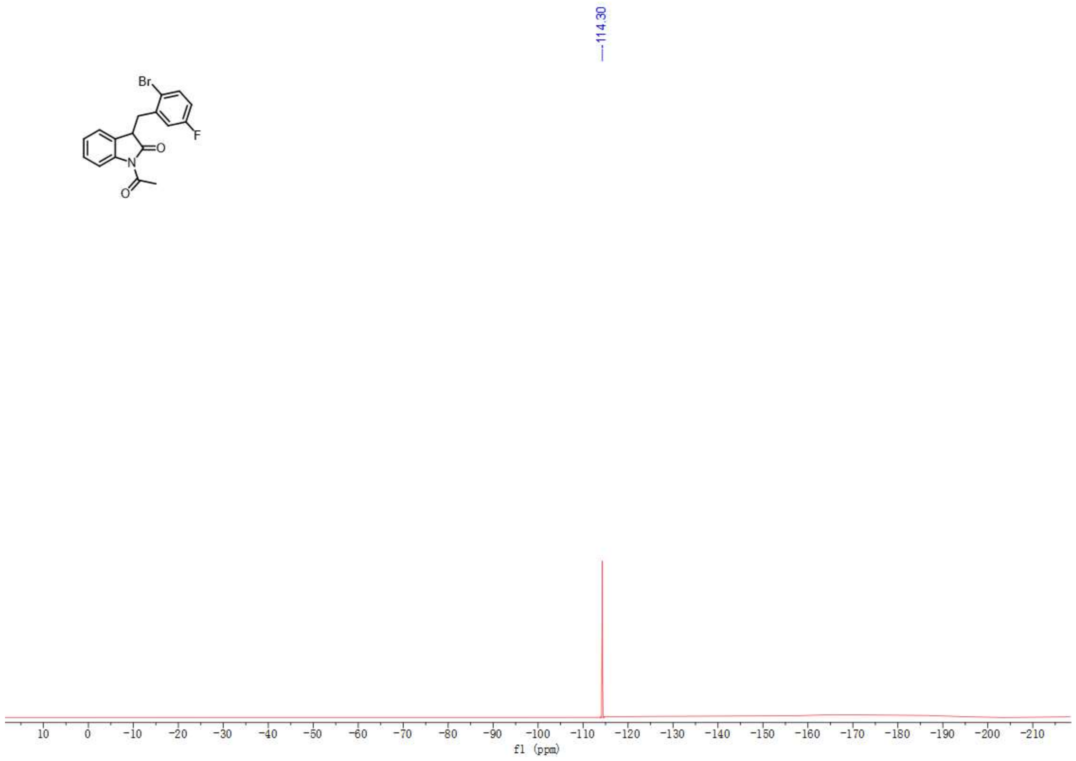

^{1}H NMR of **1l**, 400 MHz, $CDCl_3$

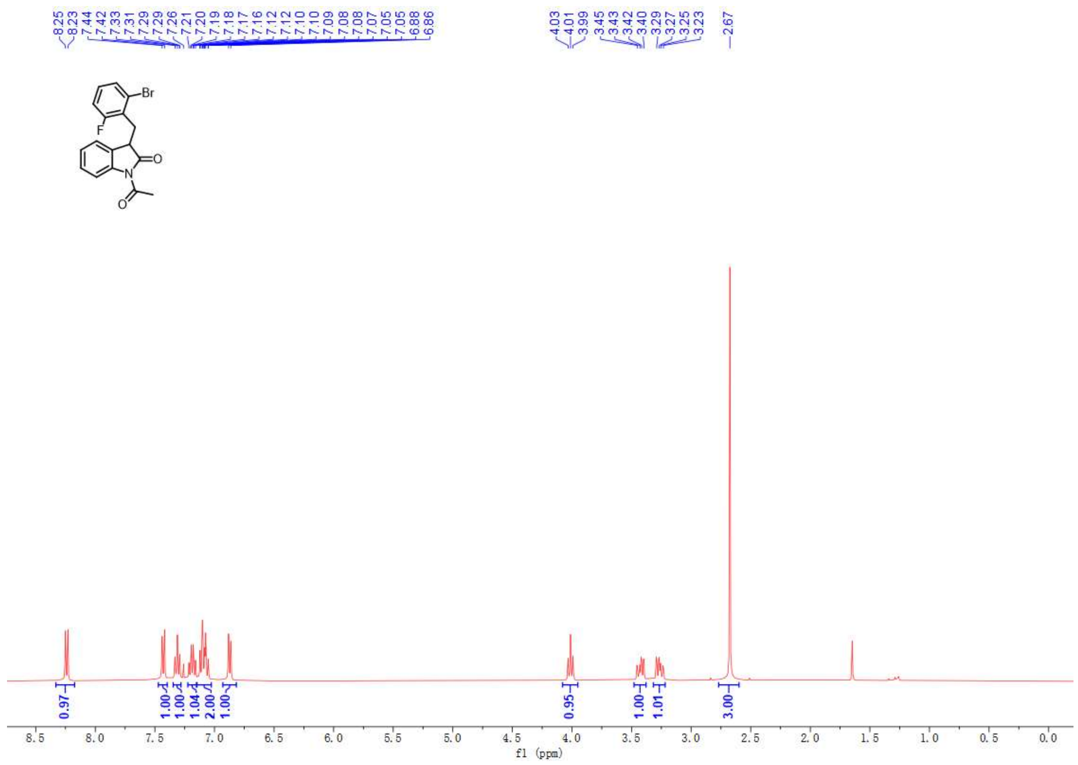

^{13}C {^{1}H} NMR of **1l**, 100 MHz, $CDCl_3$

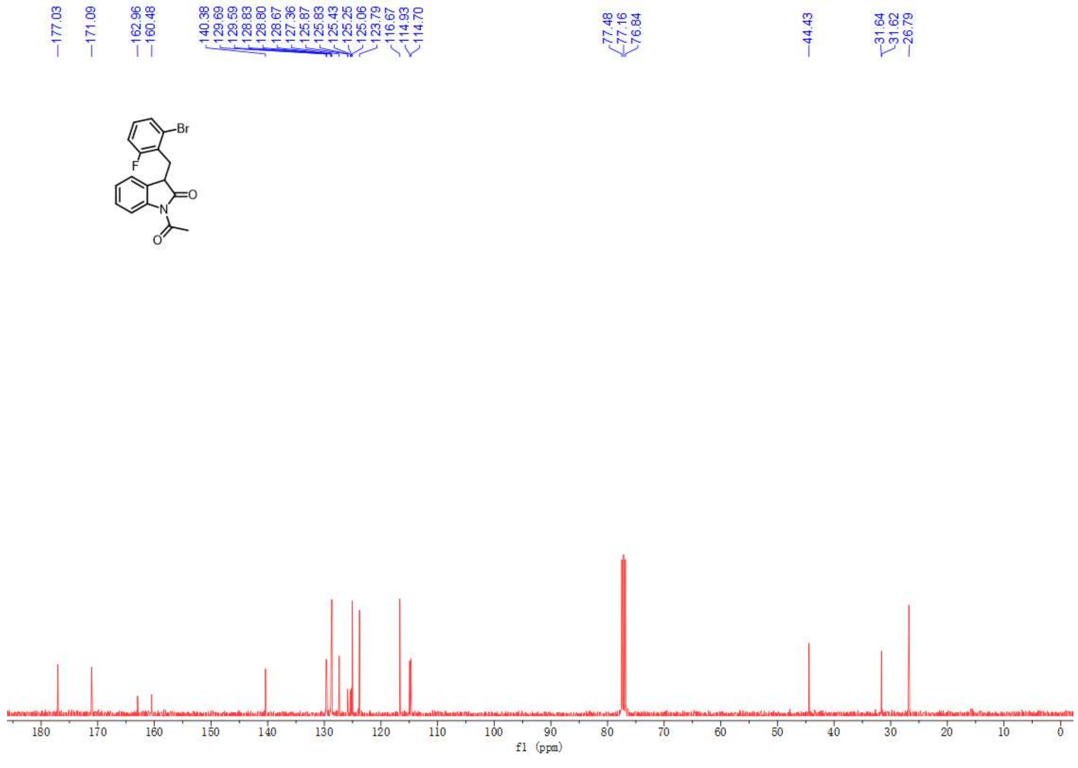

^{19}F NMR of **1l**, 376 MHz, $CDCl_3$

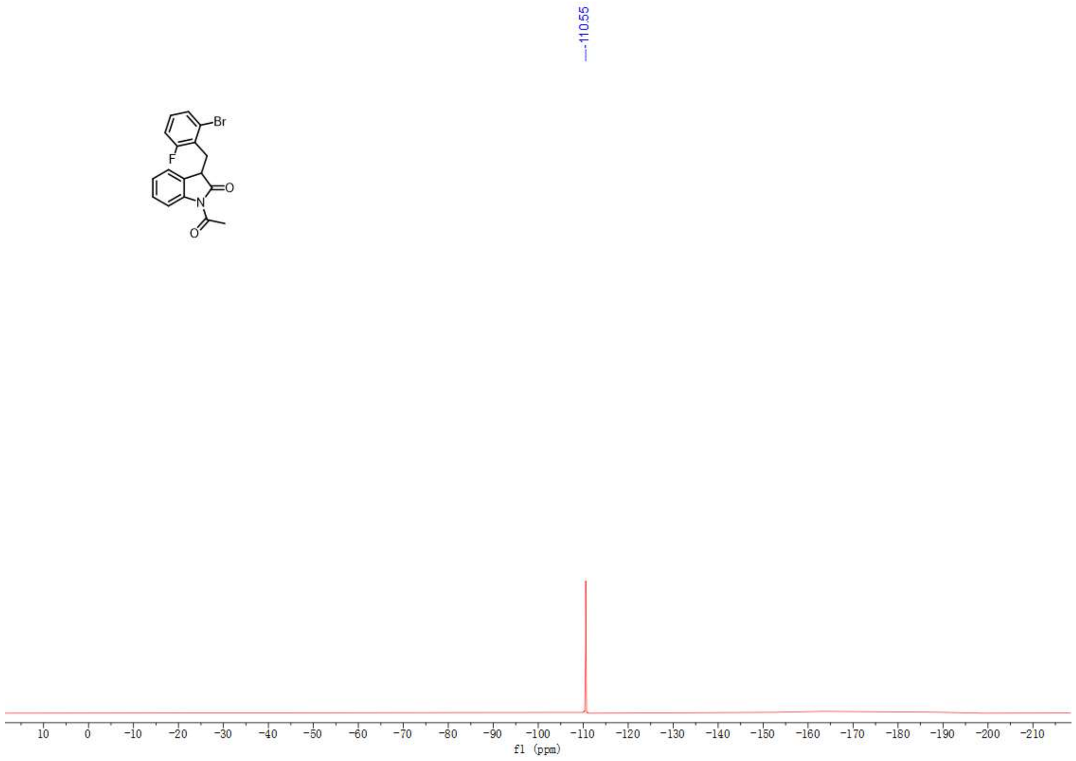

^{1}H **NMR** of **1m**, 400 MHz, $CDCl_3$

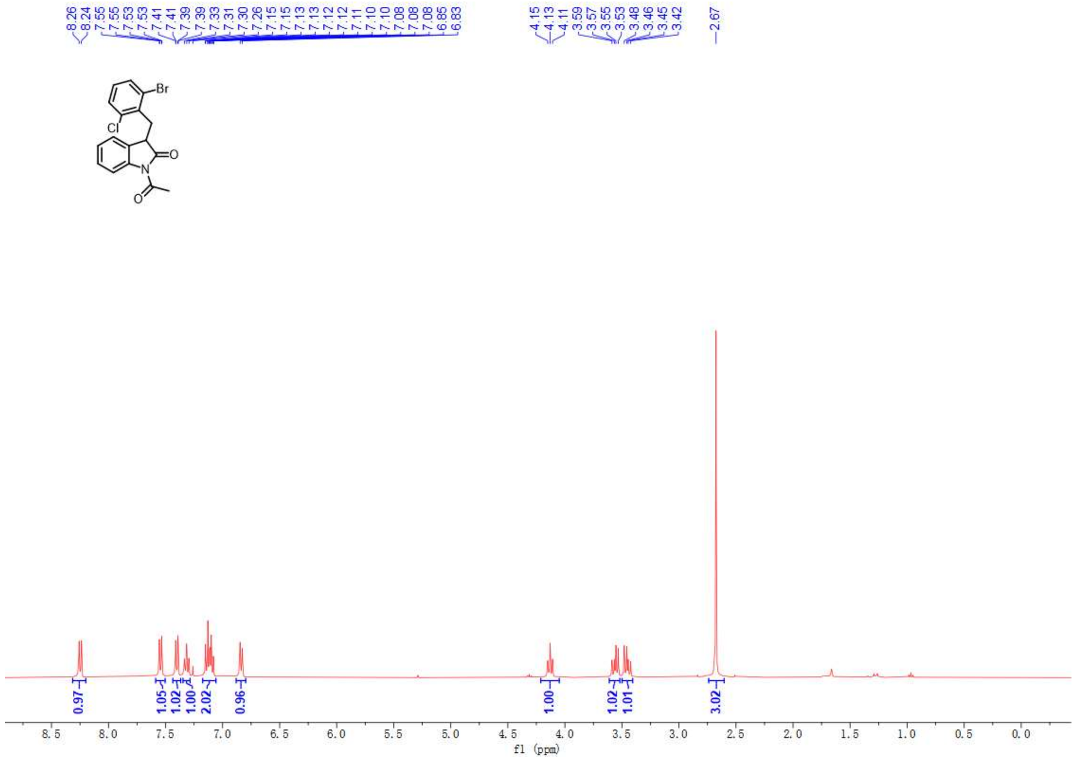

^{13}C {^{1}H} **NMR** of **1m**, 100 MHz, $CDCl_3$

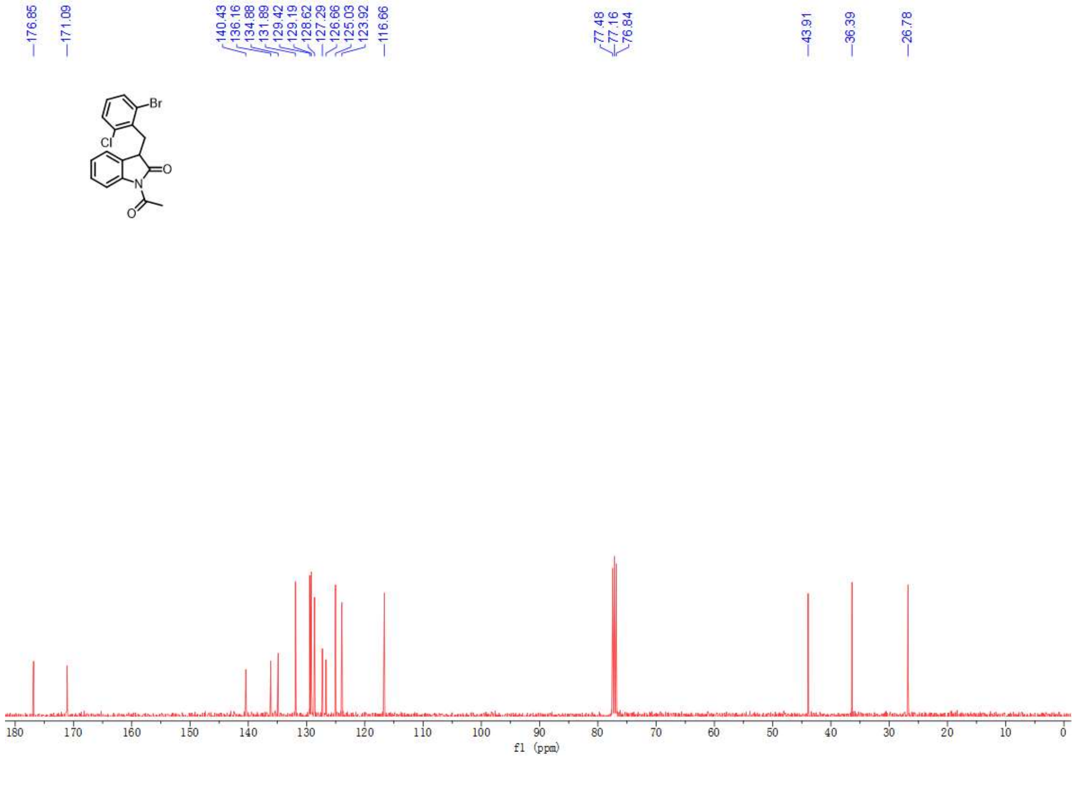

^{1}H NMR of **1n**, 400 MHz, $CDCl_3$

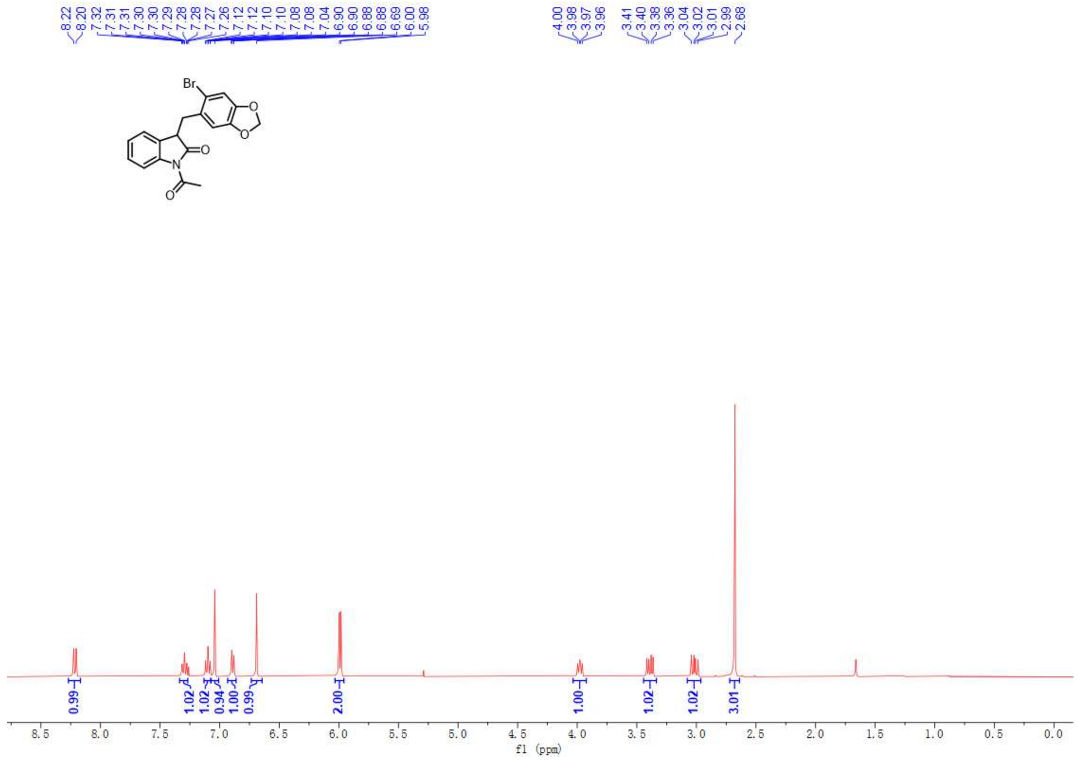

^{13}C {^{1}H} NMR of **1n**, 100 MHz, $CDCl_3$

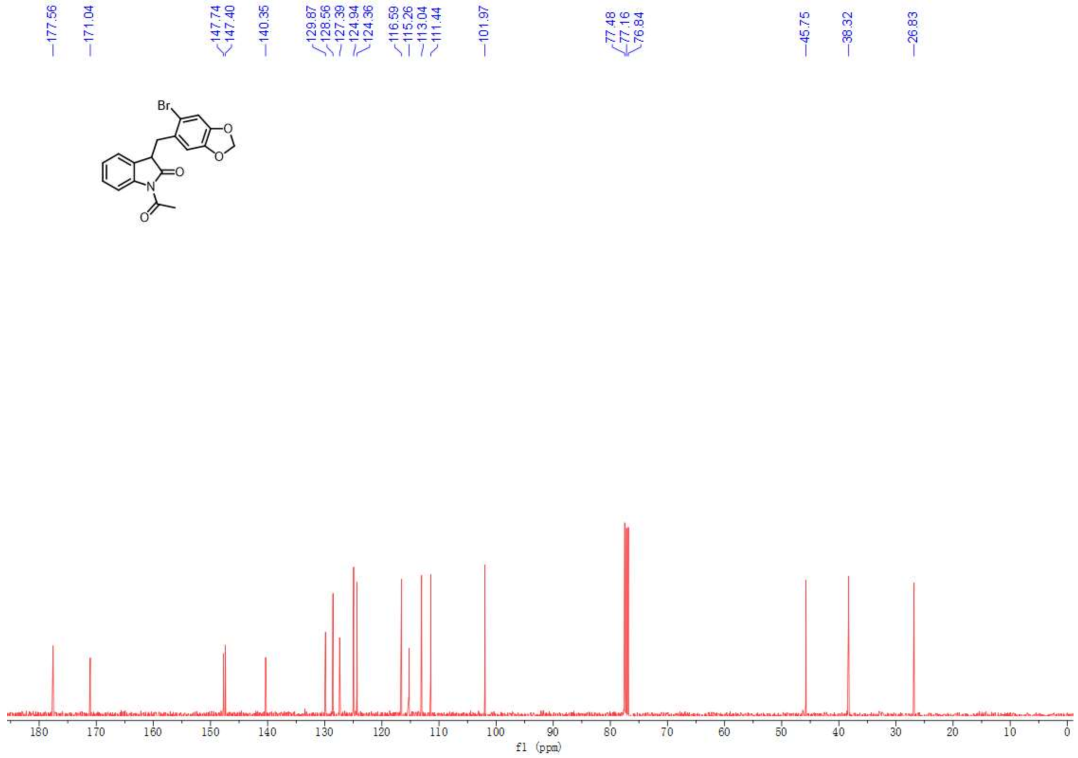

^{1}**H NMR** of **1o**, 400 MHz, $CDCl_3$

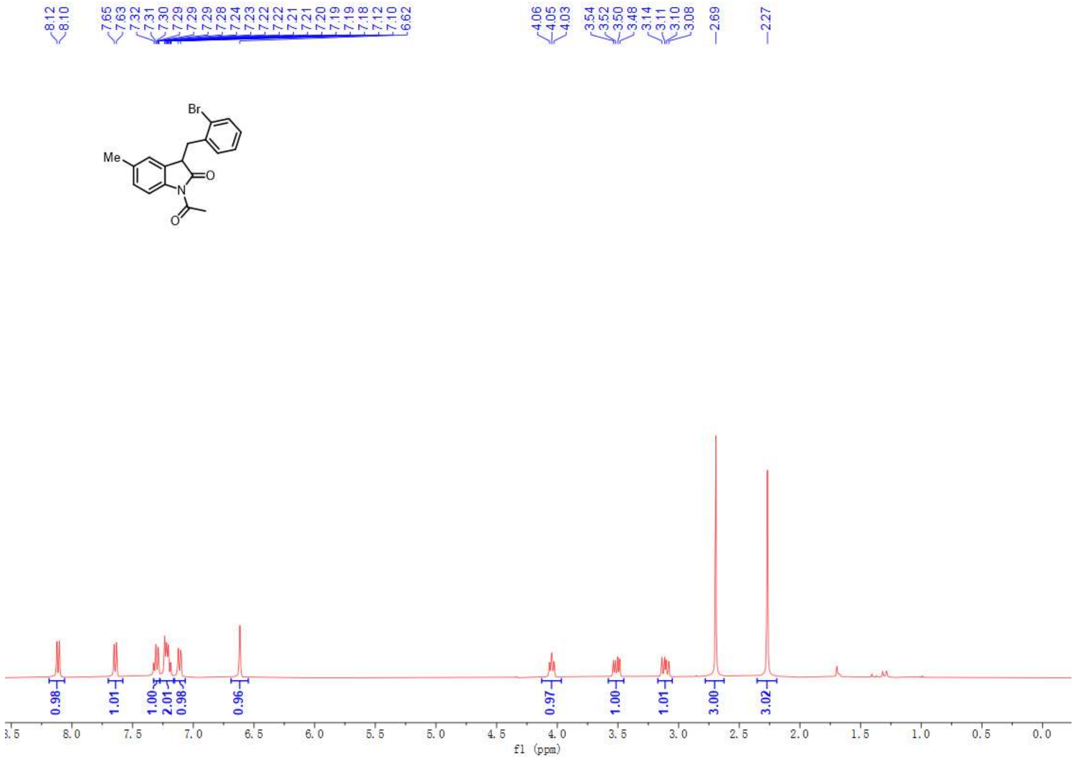

^{13}**C** {^{1}**H**} **NMR** of **1o**, 100 MHz, $CDCl_3$

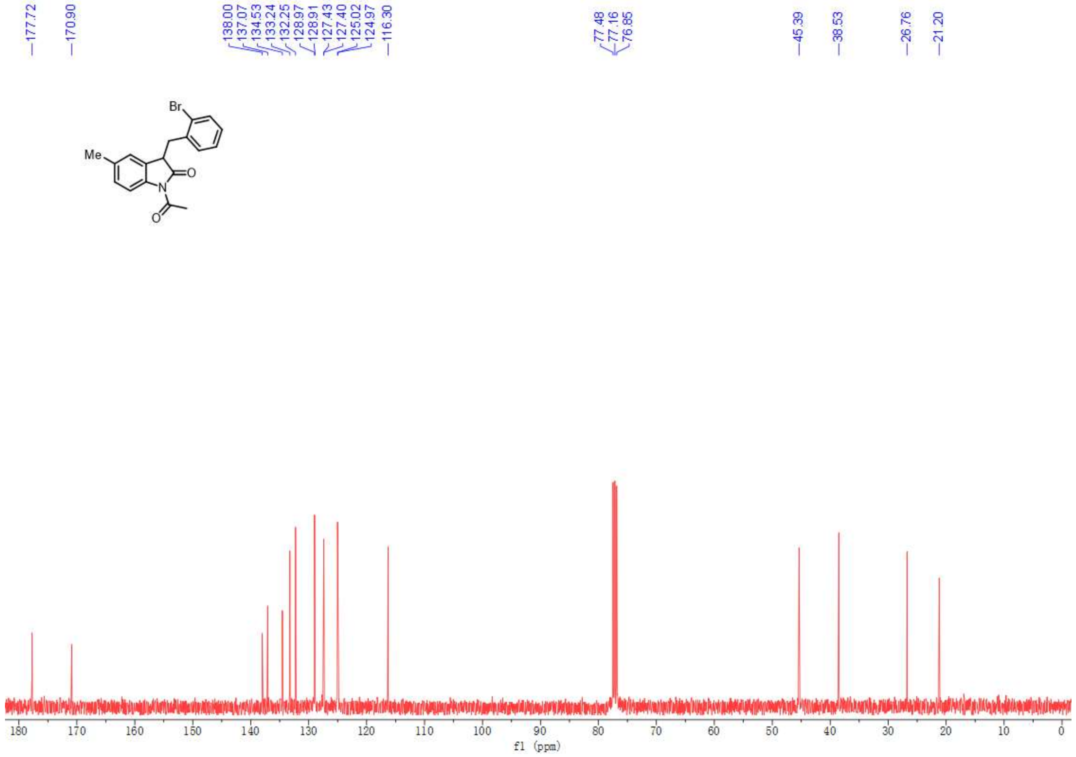

^{1}H NMR of **1p**, 400 MHz, $CDCl_3$

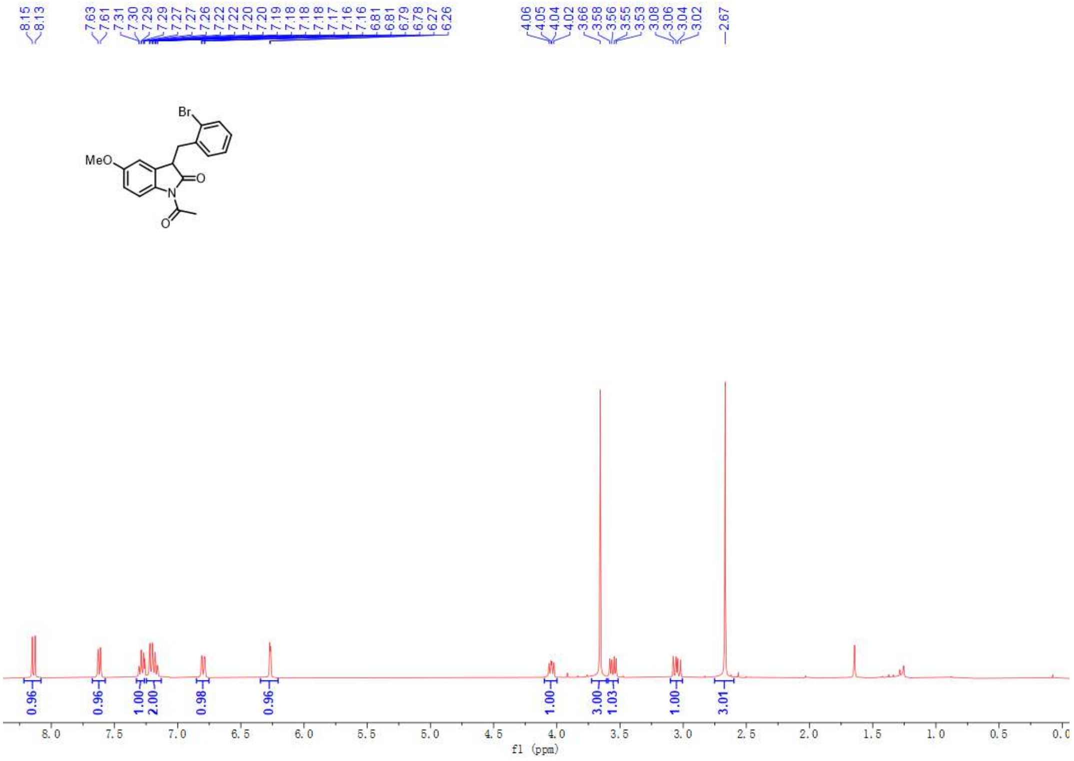

^{13}C {^{1}H} NMR of **1p**, 100 MHz, $CDCl_3$

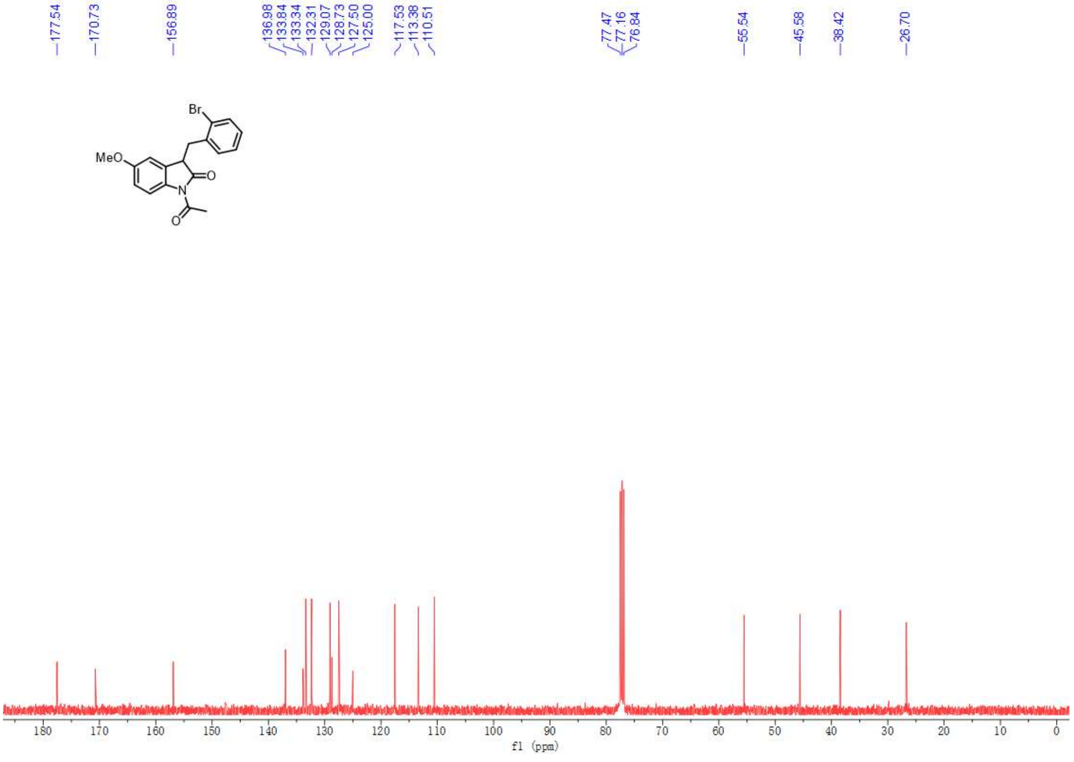

^{1}H NMR of **1q**, 400 MHz, $CDCl_3$

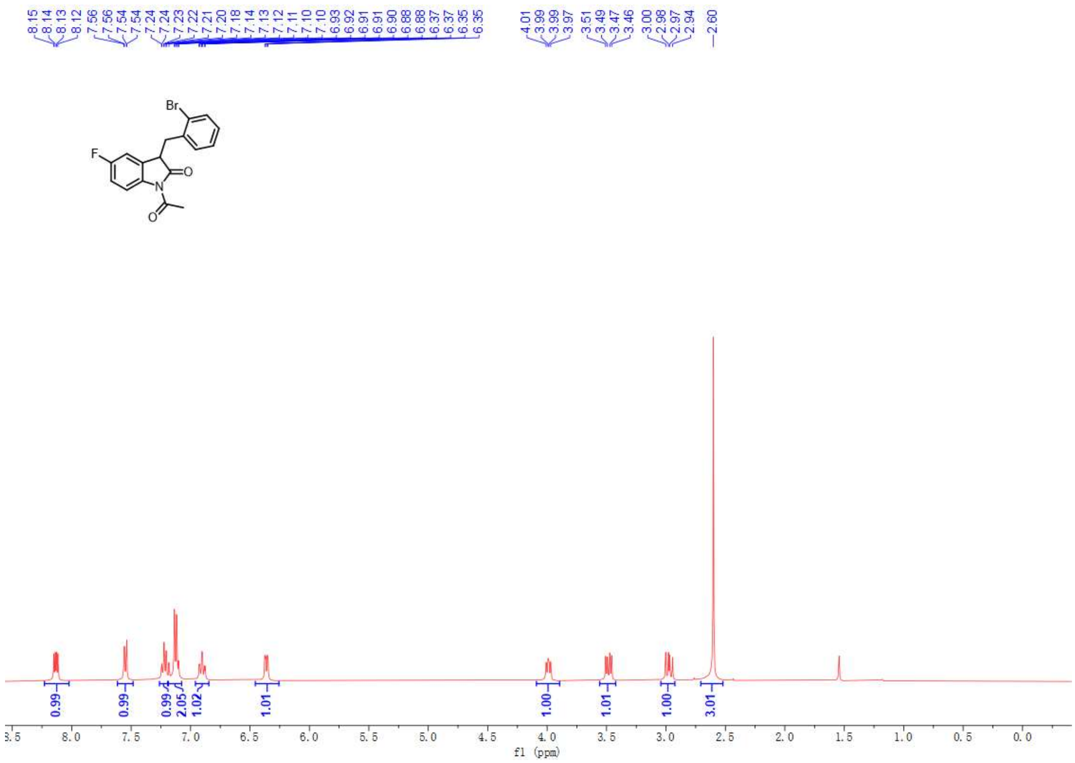

^{13}C {^{1}H} NMR of **1q**, 100 MHz, $CDCl_3$

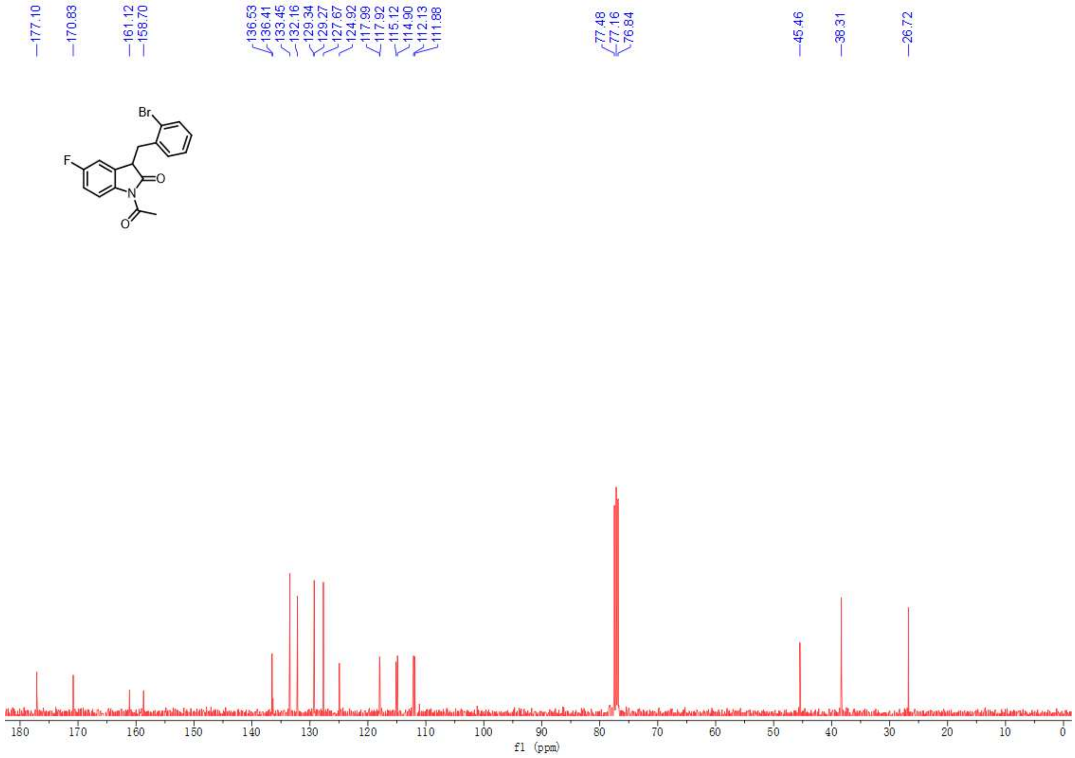

^{19}F NMR of **1q**, 376 MHz, $CDCl_3$

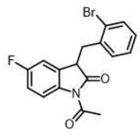

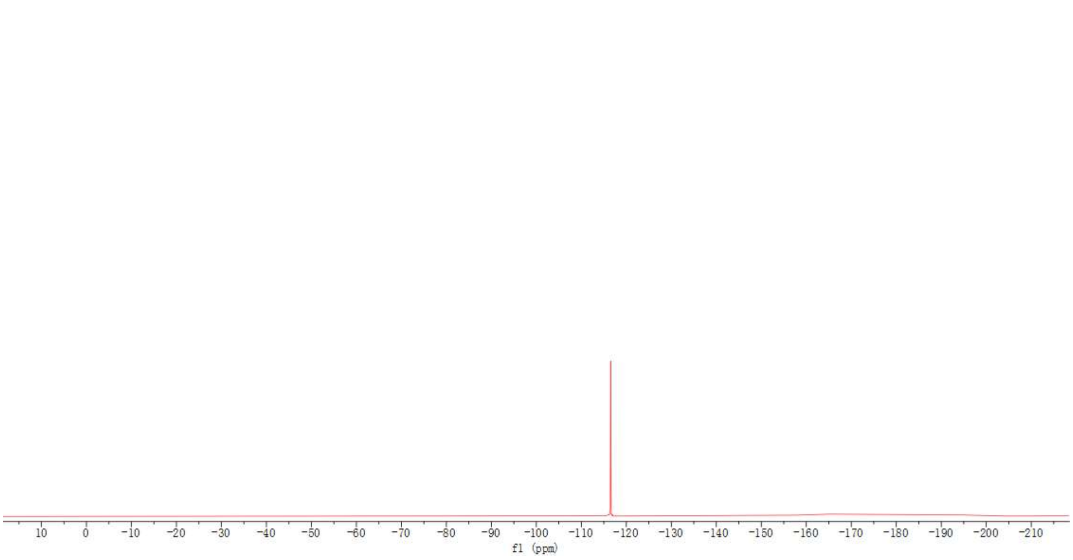

^{1}H NMR of **1r**, 400 MHz, $CDCl_3$

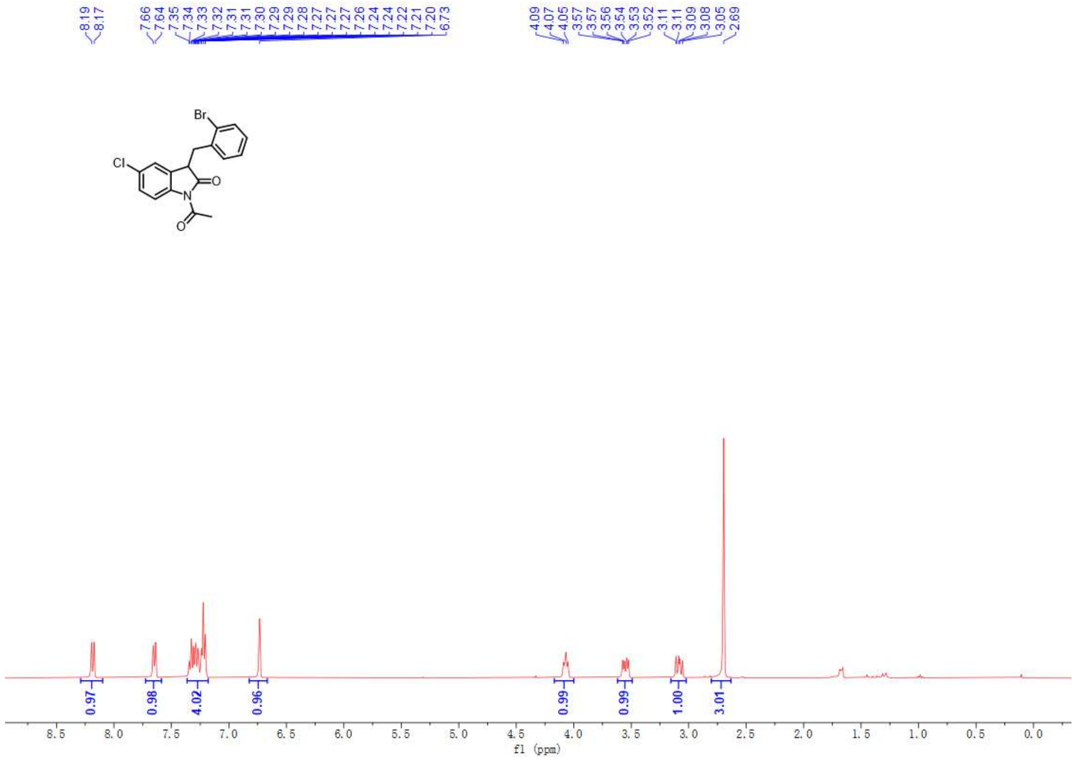

^{13}C {^{1}H} NMR of **1r**, 100 MHz, $CDCl_3$

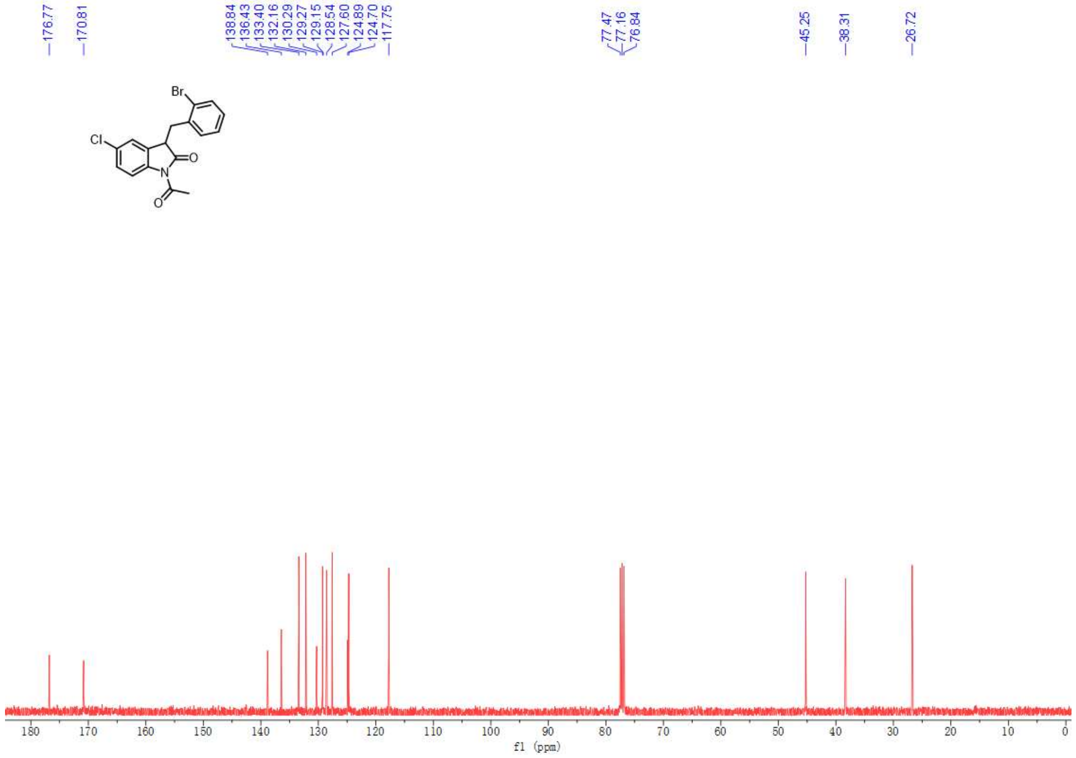

^{1}H **NMR** of **1s**, 400 MHz, $CDCl_3$

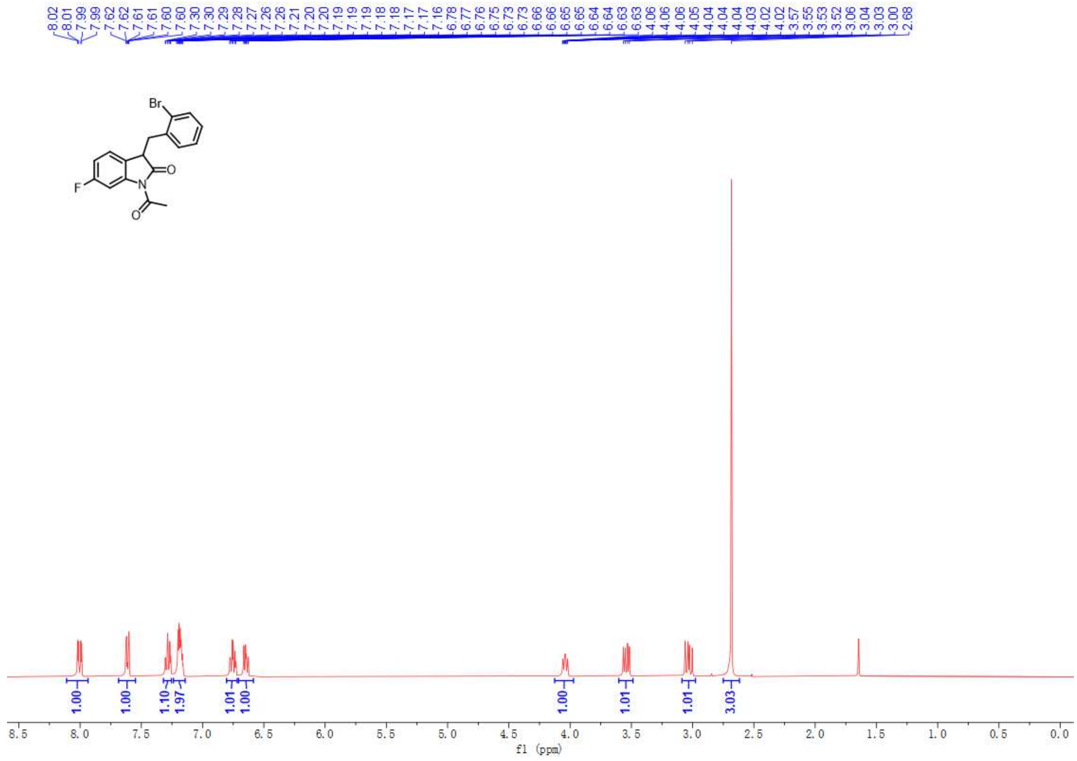

^{13}C {^{1}H} **NMR** of **1s**, 100 MHz, $CDCl_3$

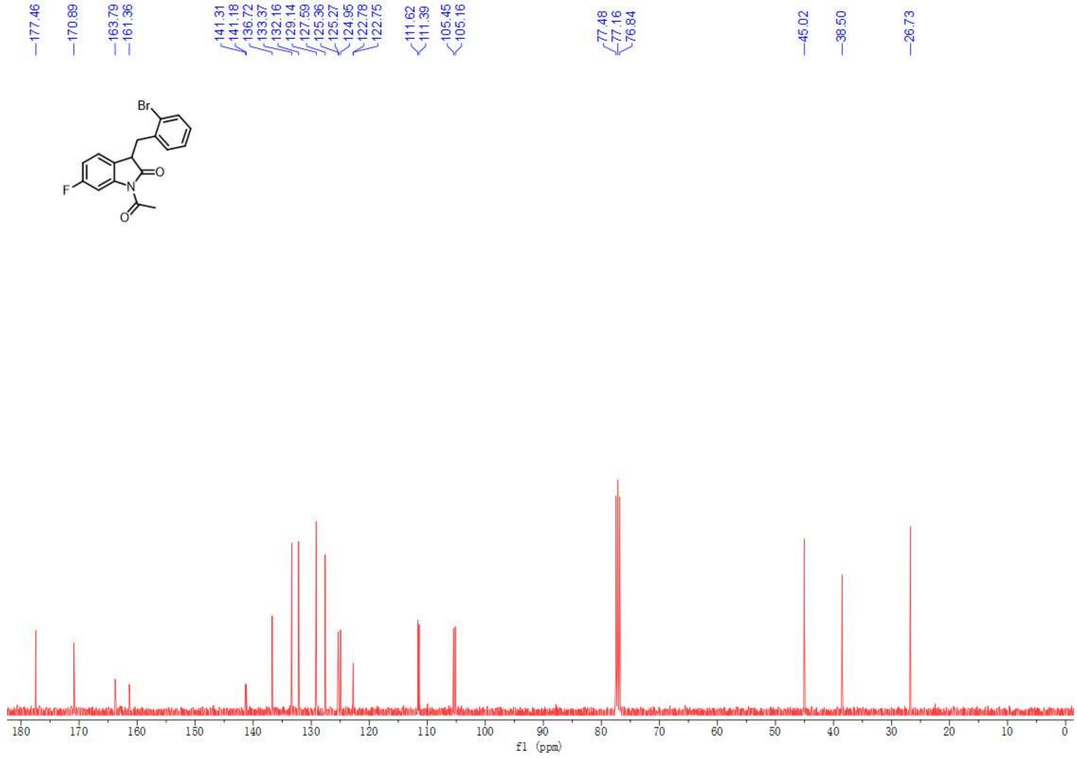

^{19}**F NMR** of **1s**, 376 MHz, $CDCl_3$

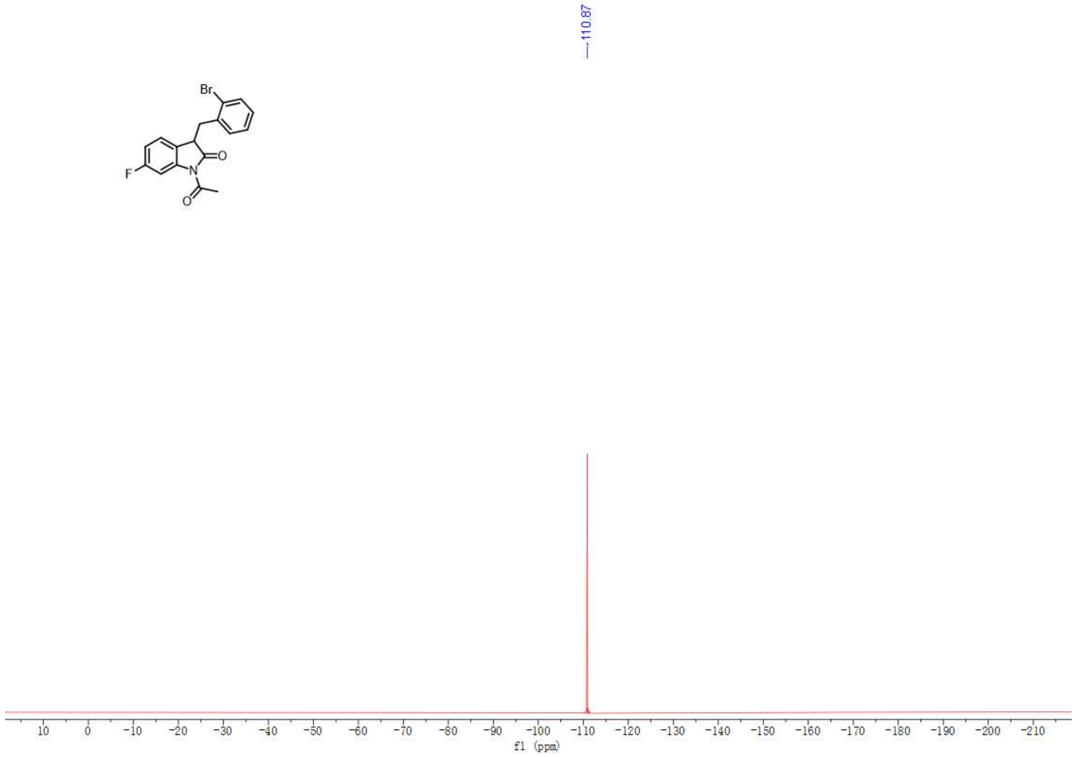

^{1}H NMR of **1t**, 400 MHz, $CDCl_3$

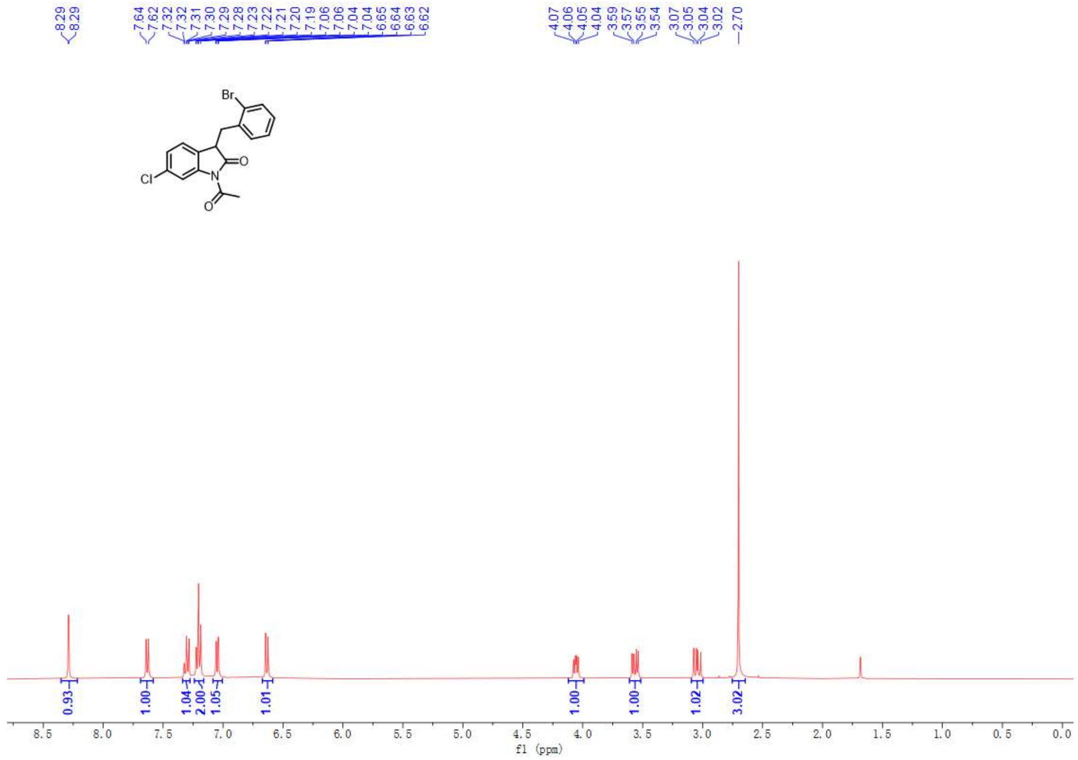

^{13}C {^{1}H} NMR of **1t**, 100 MHz, $CDCl_3$

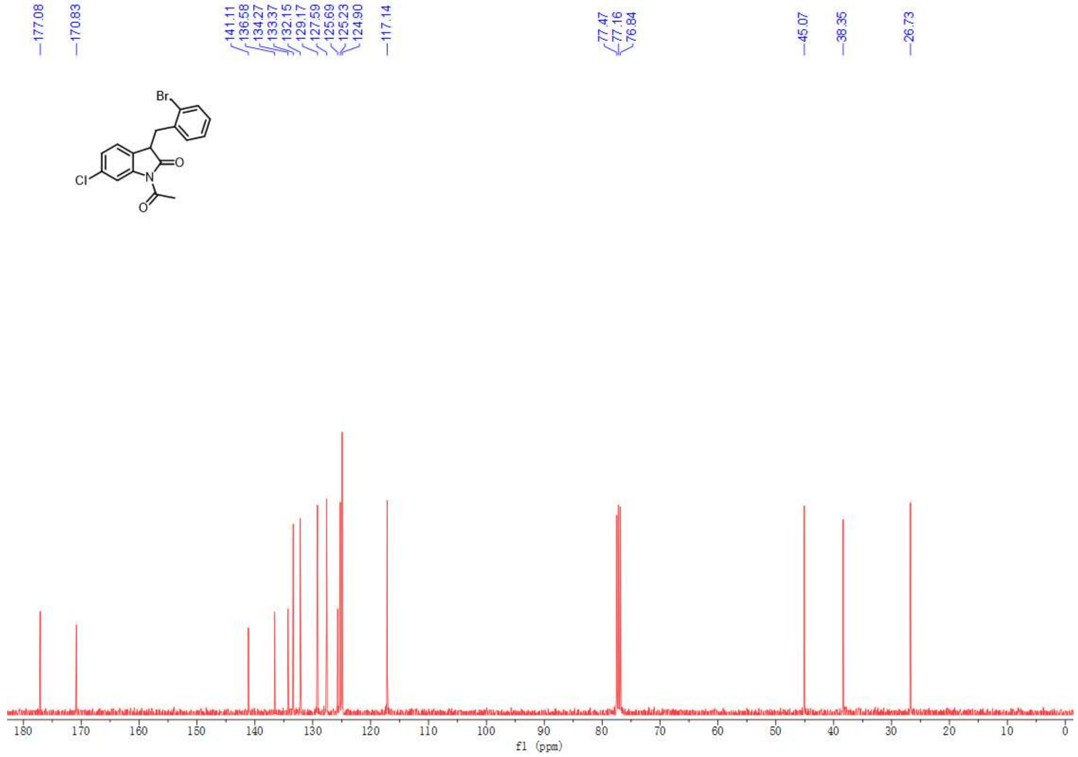

^{1}H NMR of **1u**, 400 MHz, $CDCl_3$

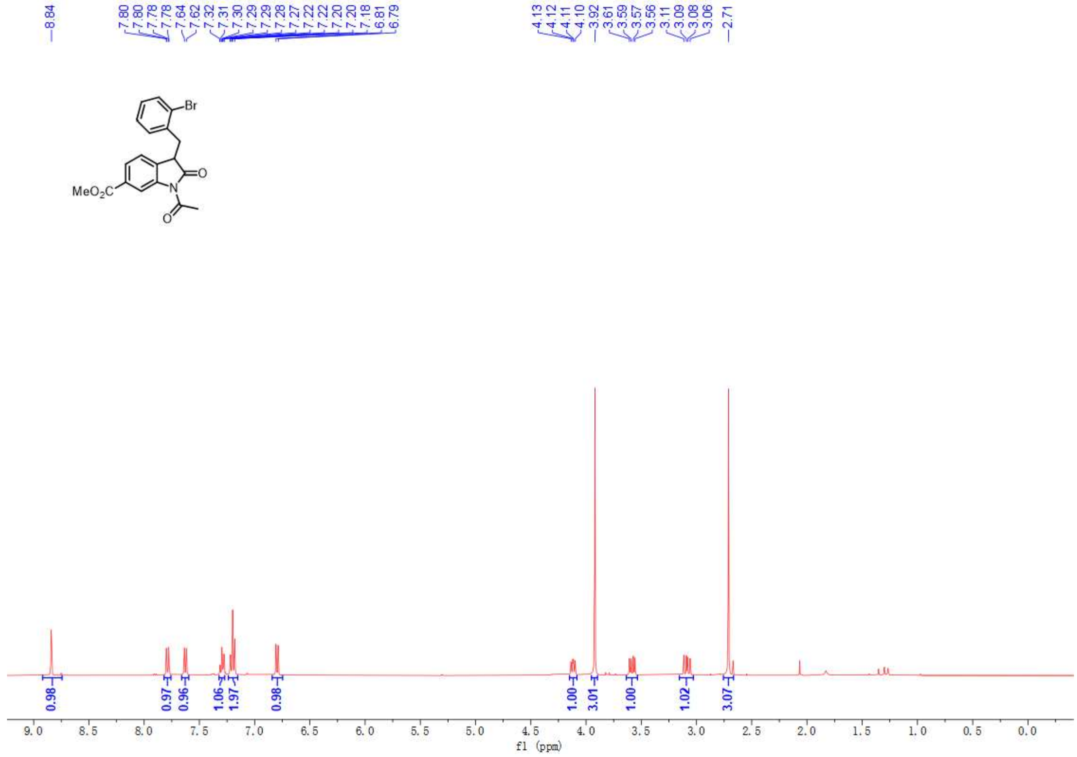

^{13}C {^{1}H} NMR of **1u**, 100 MHz, $CDCl_3$

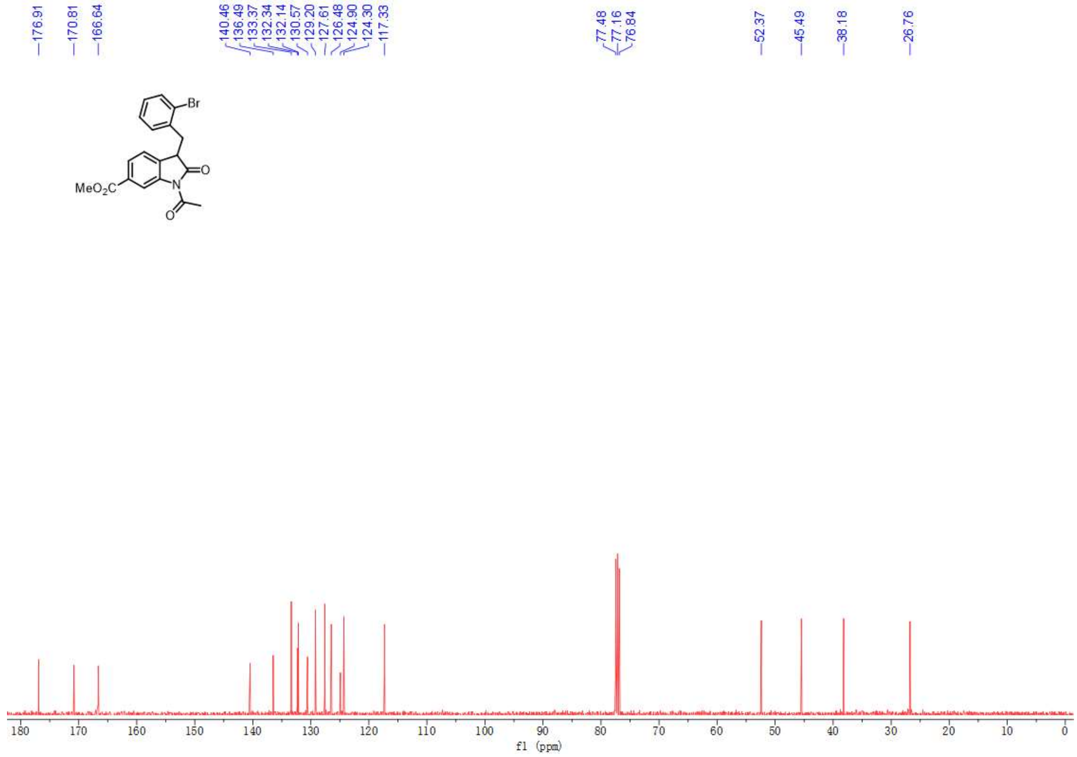

^{1}H NMR of **1v**, 400 MHz, $CDCl_3$

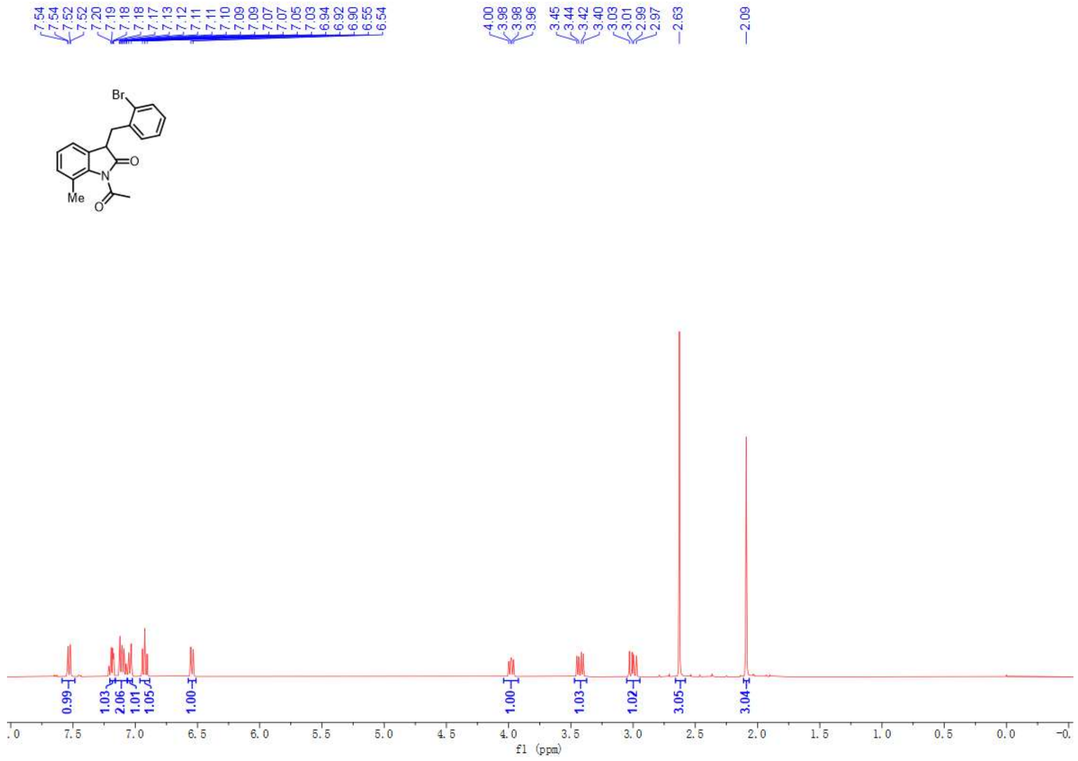

^{13}C {^{1}H} NMR of **1v**, 100 MHz, $CDCl_3$

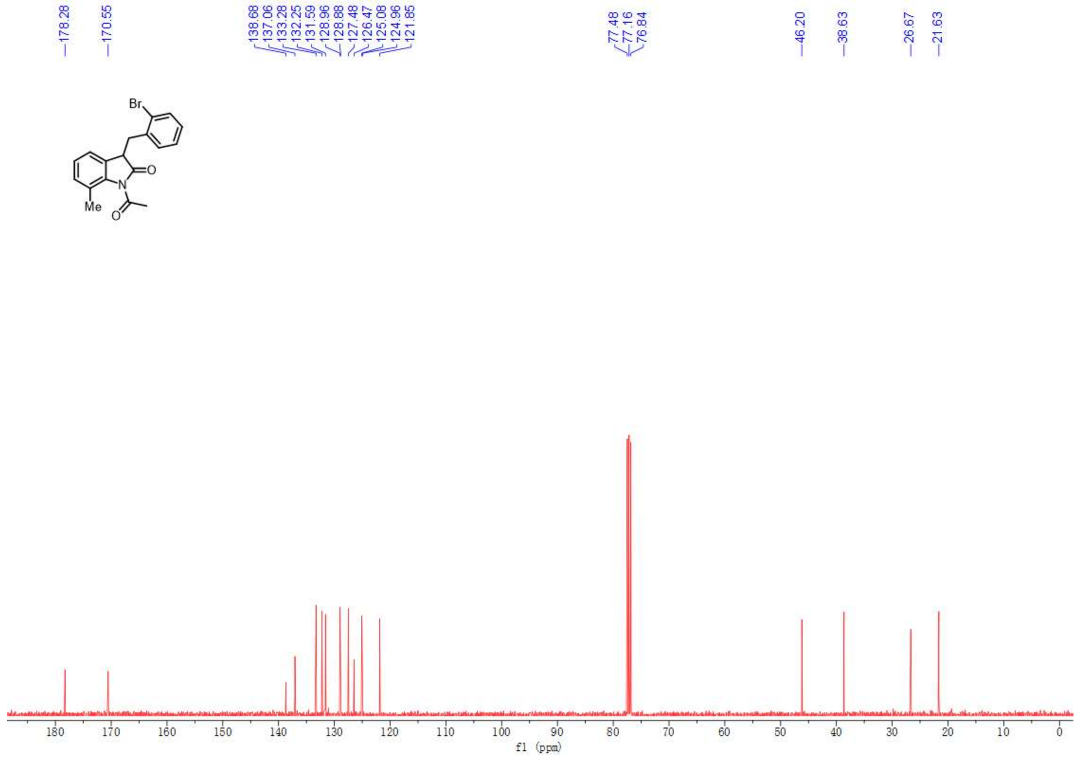

^{1}H NMR of **1w**, 400 MHz, $CDCl_3$

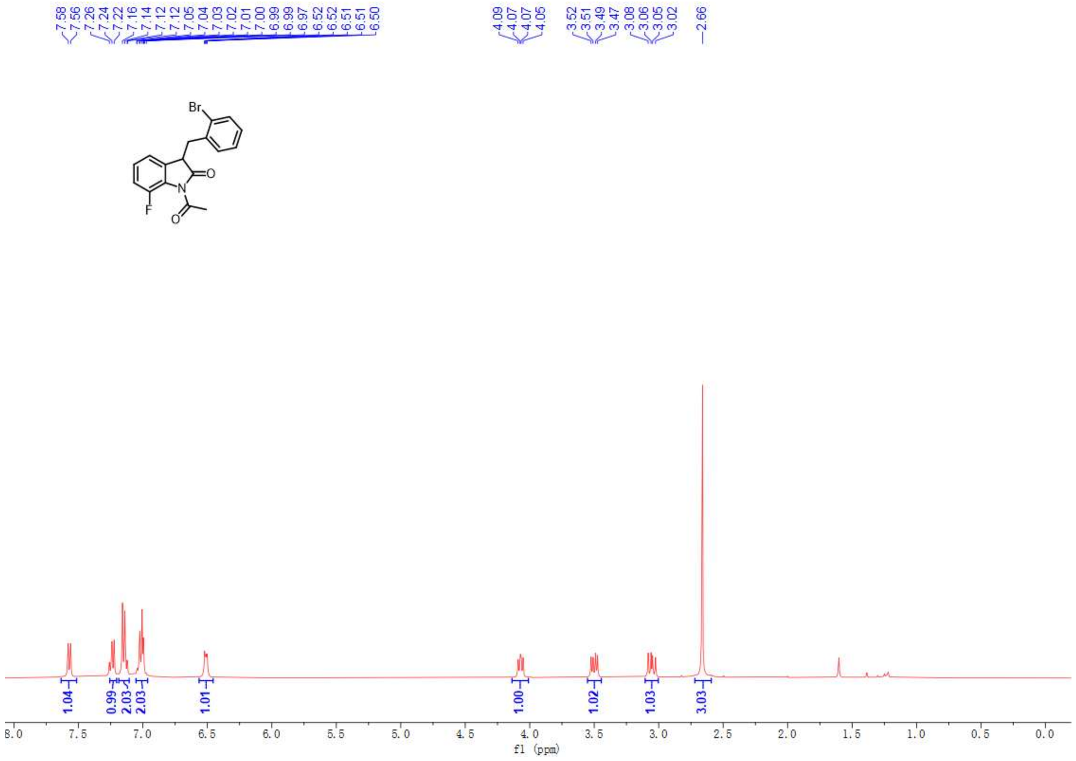

^{13}C {^{1}H} NMR of **1w**, 100 MHz, $CDCl_3$

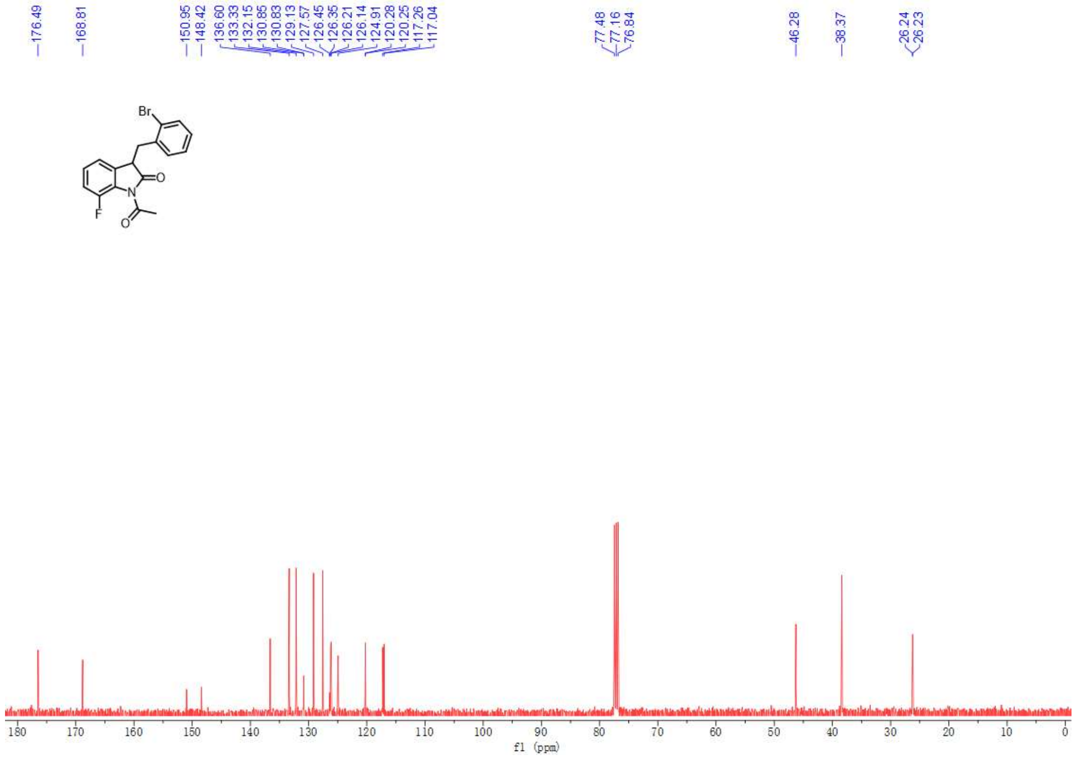

^{19}F NMR of **1w**, 376 MHz, $CDCl_3$

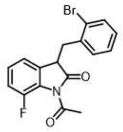

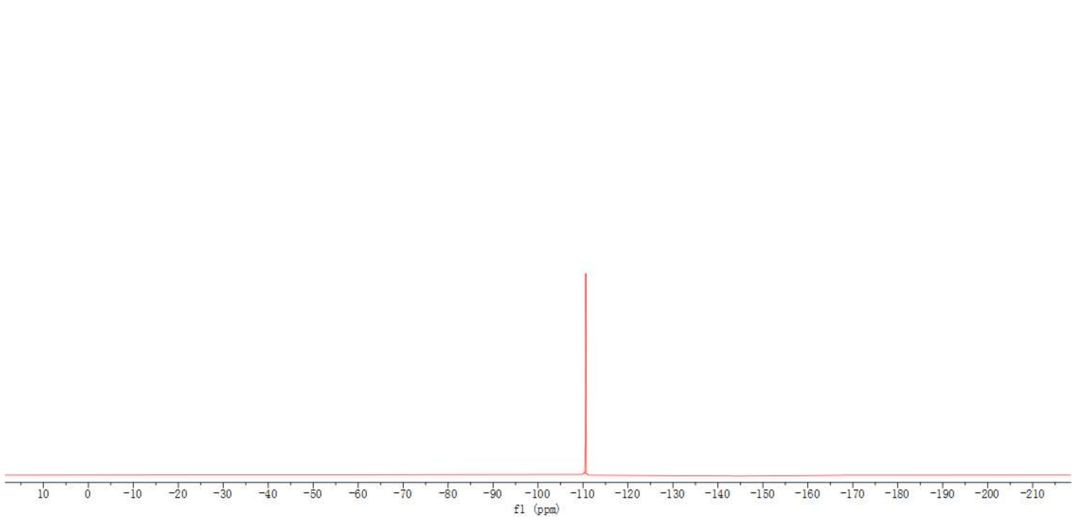

^{1}H NMR of **1w’**, 400 MHz, CDCl$_3$

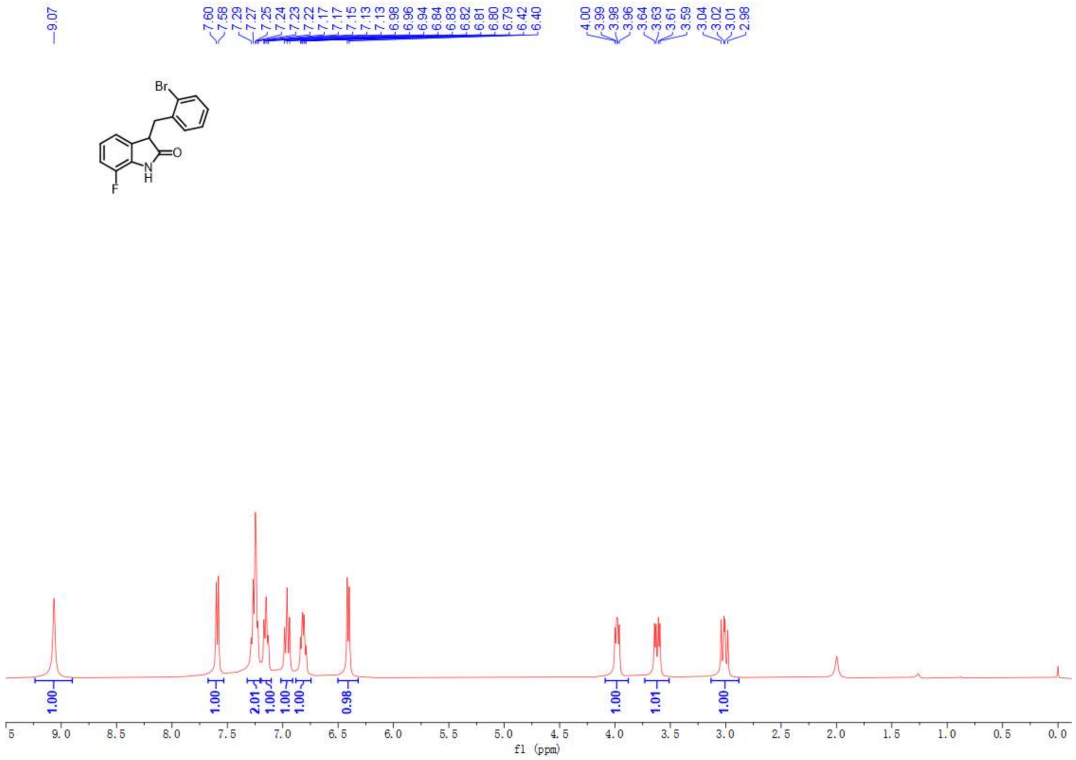

^{13}C {^{1}H} NMR of **1w’**, 100 MHz, CDCl$_3$

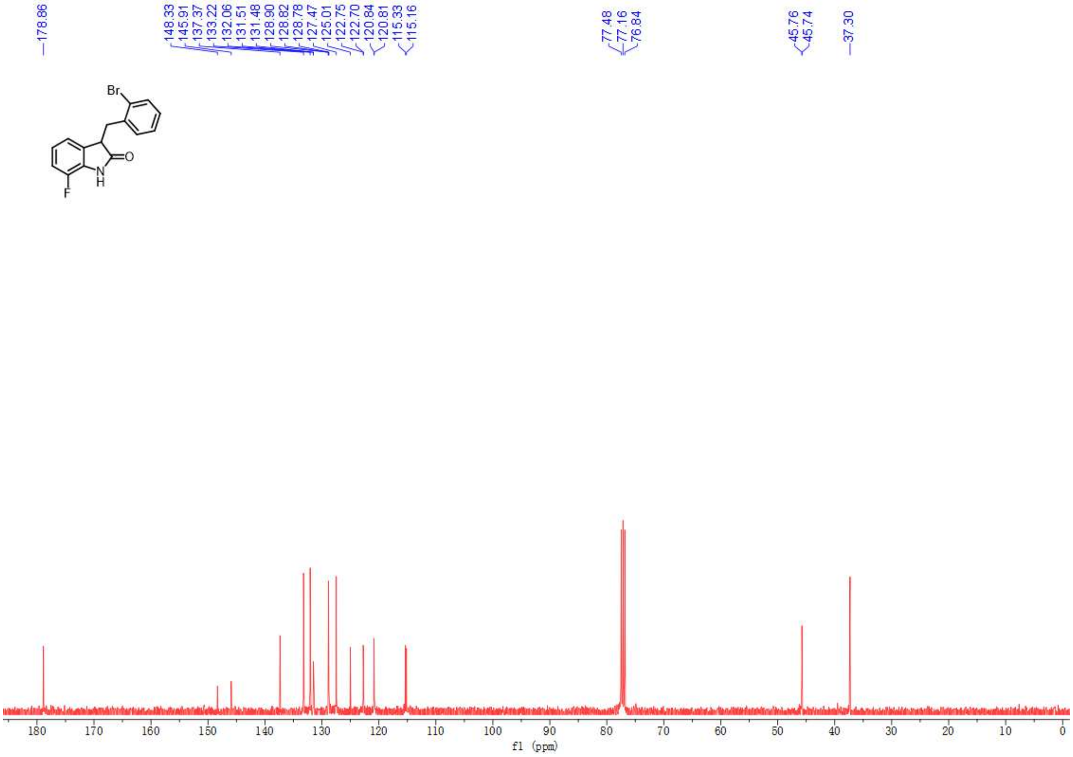

^{19}F NMR of **1w'**, 376 MHz, $CDCl_3$

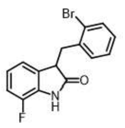

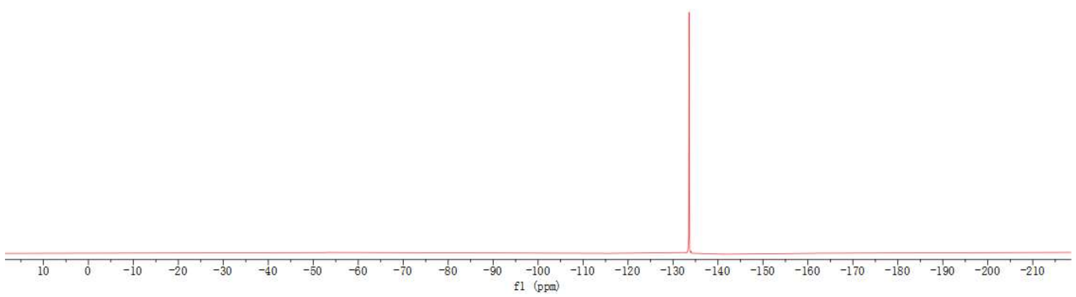

^{1}H NMR of **1x**, 400 MHz, $CDCl_3$

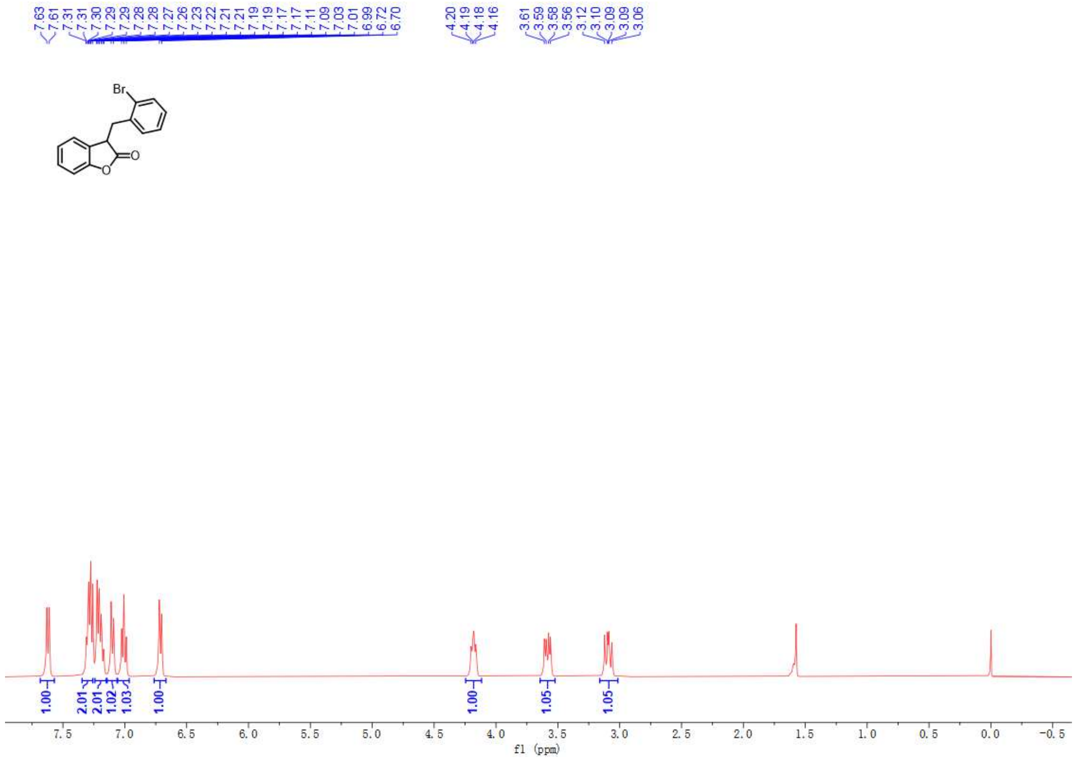

^{13}C {^{1}H} NMR of **1x**, 100 MHz, $CDCl_3$

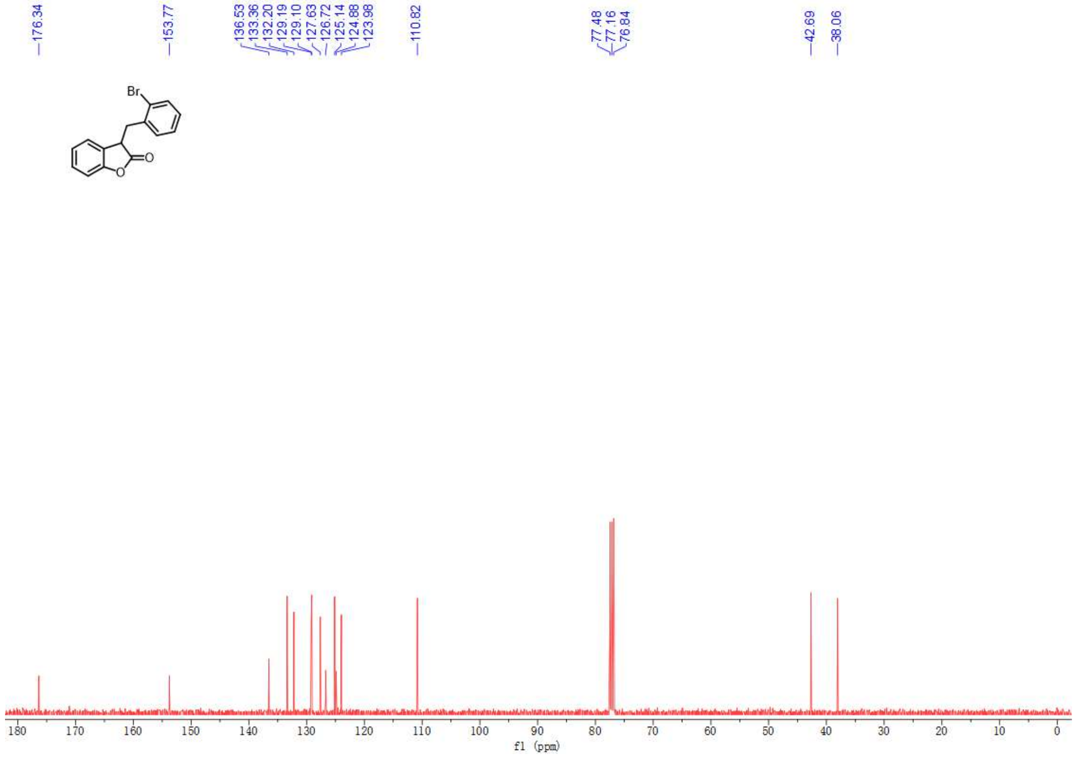

^{1}H NMR of **1y**, 400 MHz, $CDCl_3$

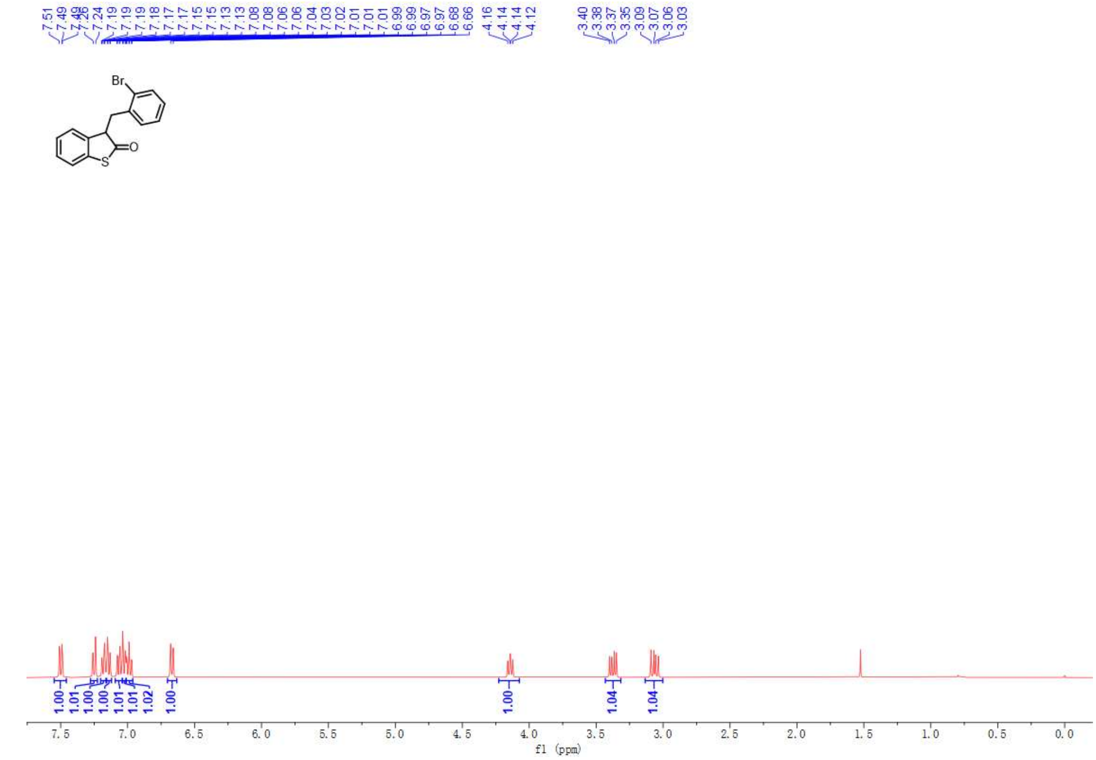

^{13}C {^{1}H} NMR of **1y**, 100 MHz, $CDCl_3$

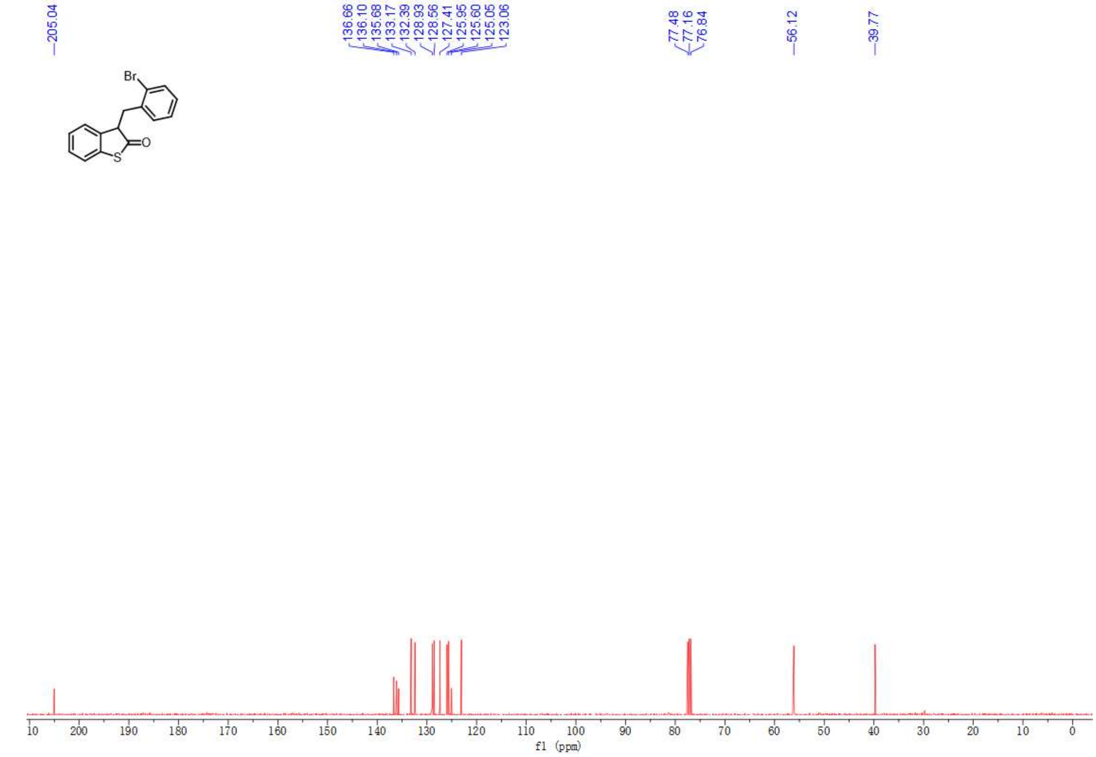

^{1}H NMR of **1z**, 400 MHz, $CDCl_3$

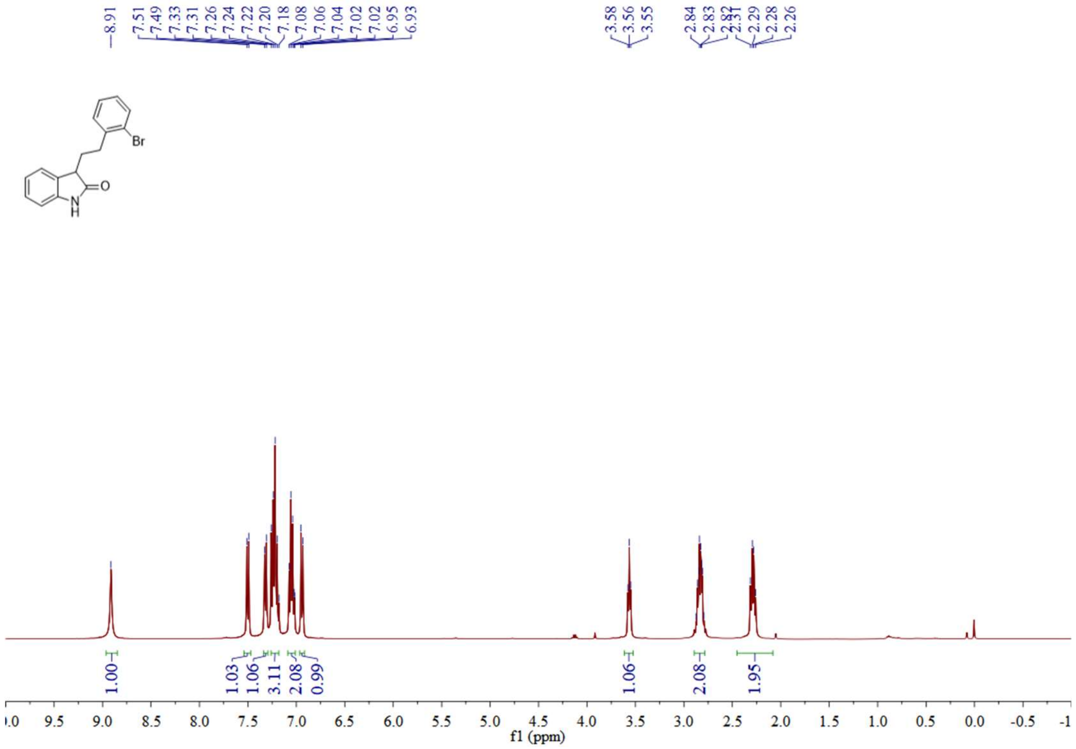

^{13}C {^{1}H} NMR of **1z**, 100 MHz, $CDCl_3$

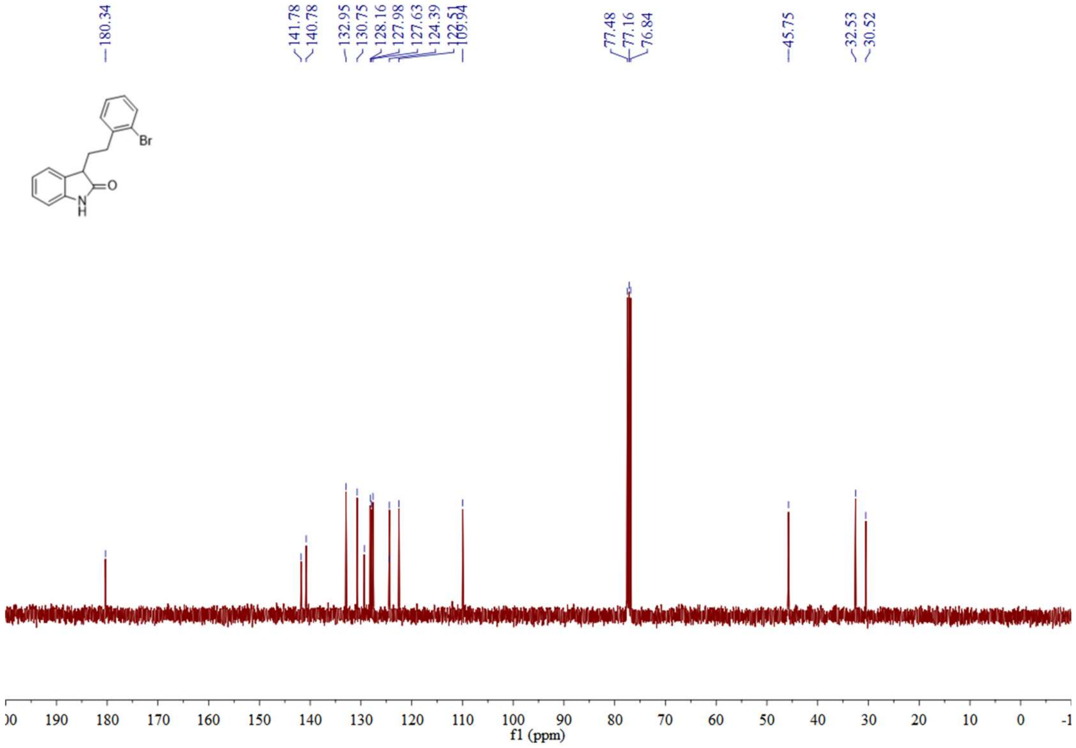

^{1}H NMR of **1aa**, 400 MHz, $CDCl_3$

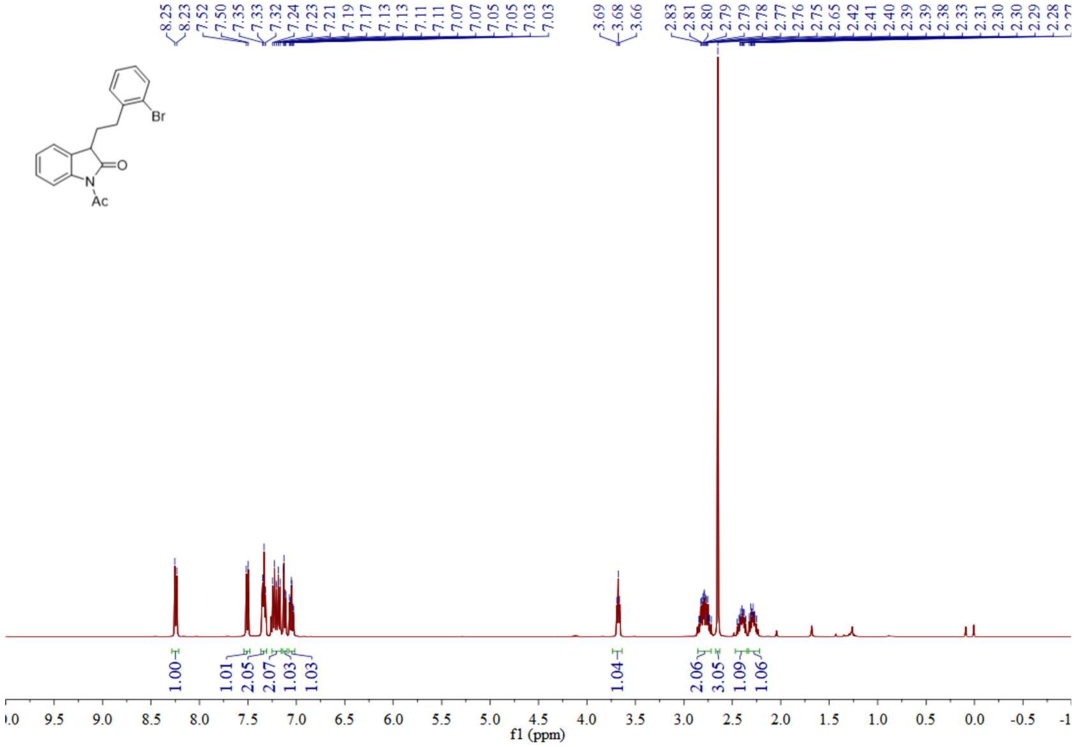

^{13}C {^{1}H} NMR of **1aa**, 100 MHz, $CDCl_3$

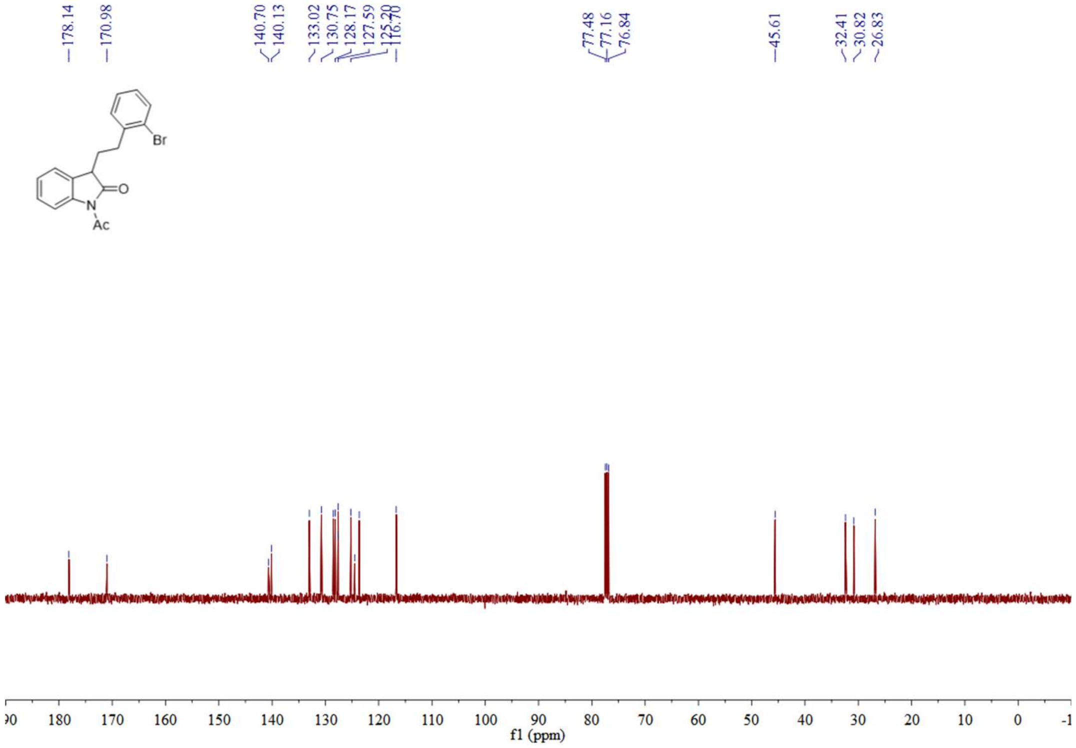

^{1}H NMR of **1ab**, 400 MHz, $CDCl_3$

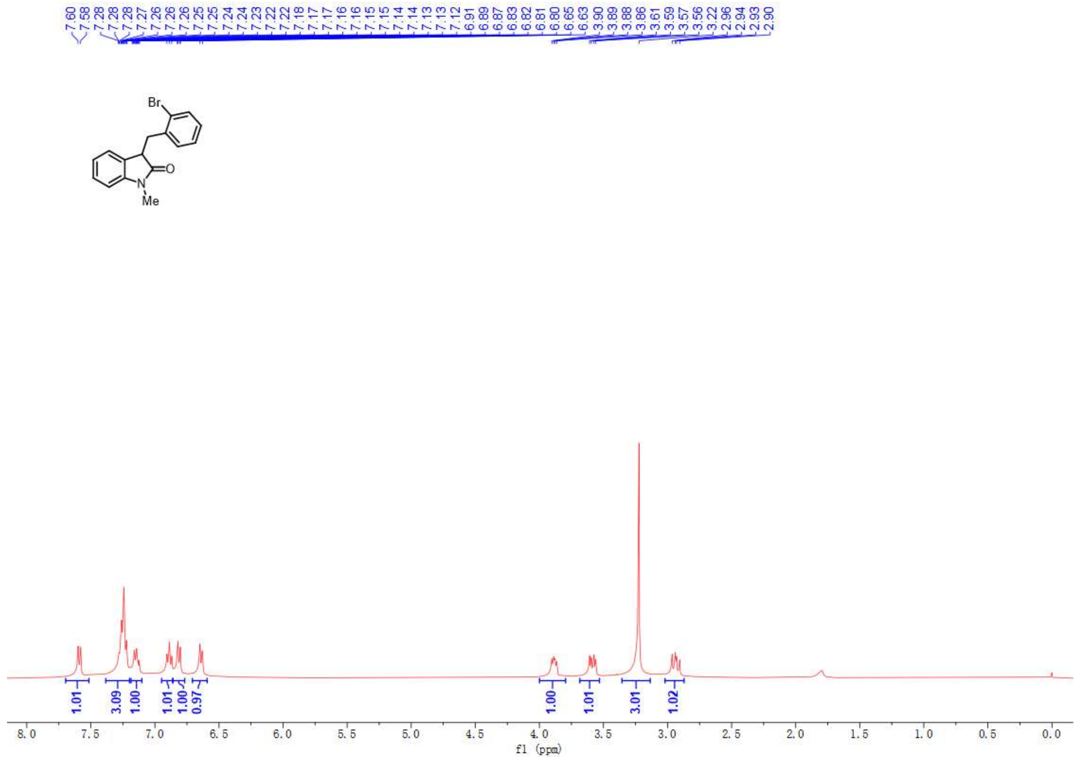

^{13}C {^{1}H} NMR of **1ab**, 100 MHz, $CDCl_3$

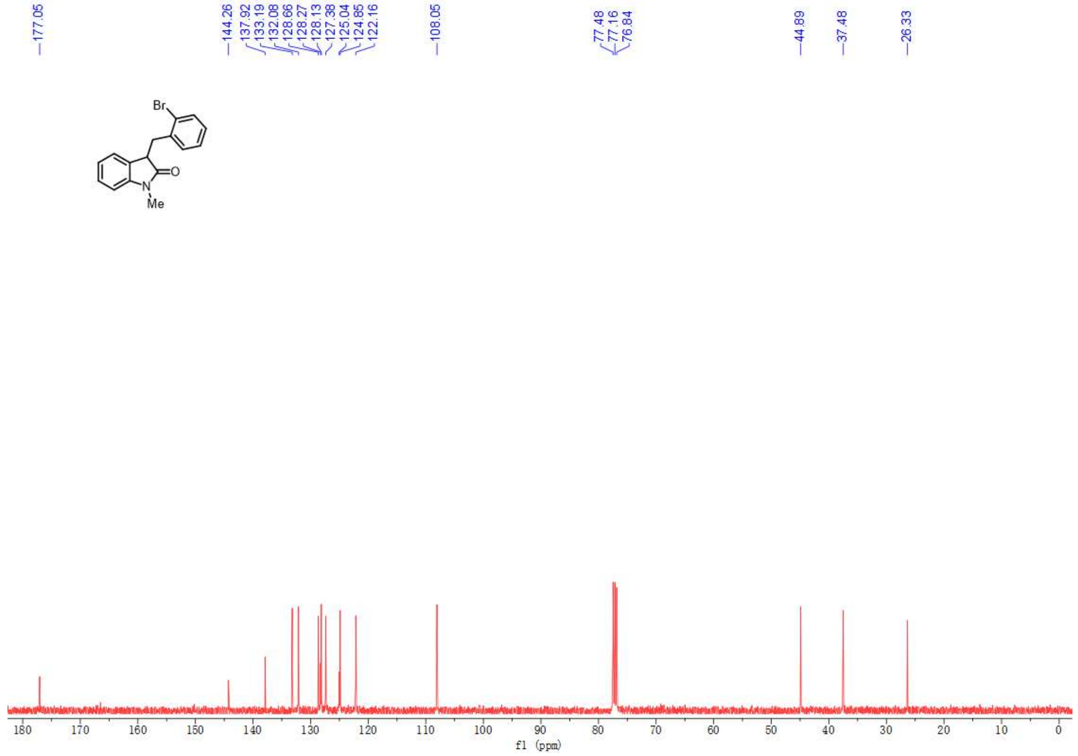

^{1}H NMR of **2a**, 400 MHz, $CDCl_3$

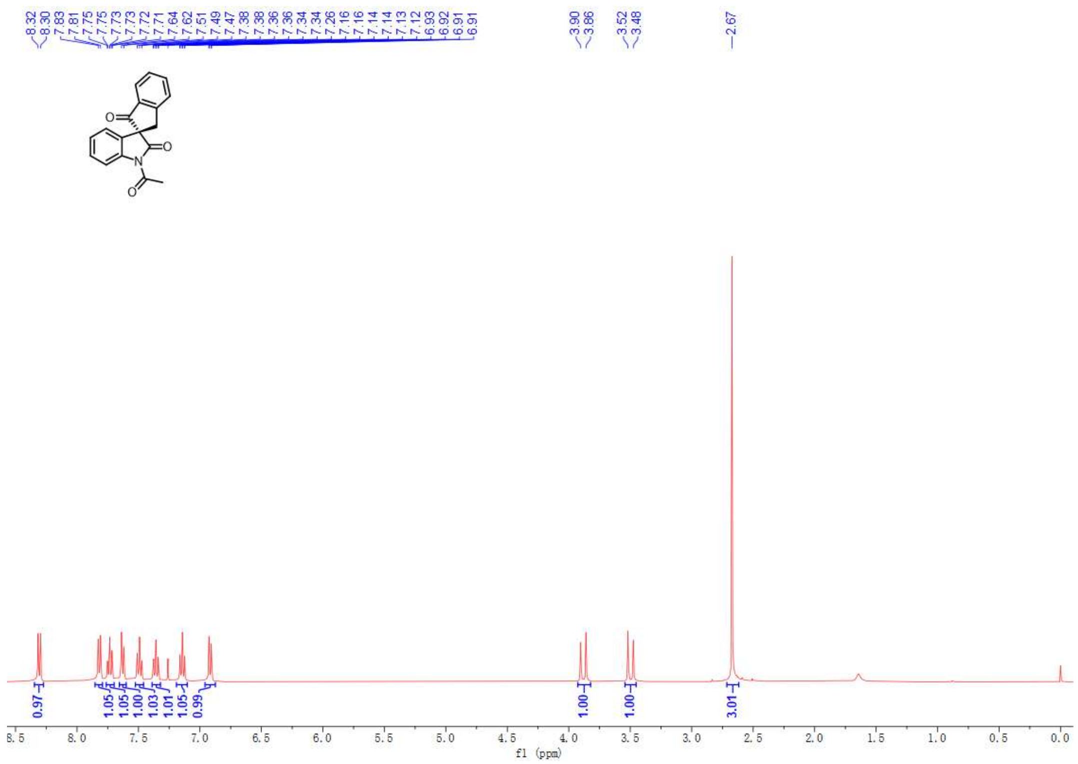

^{13}C {^{1}H} NMR of **2a**, 100 MHz, $CDCl_3$

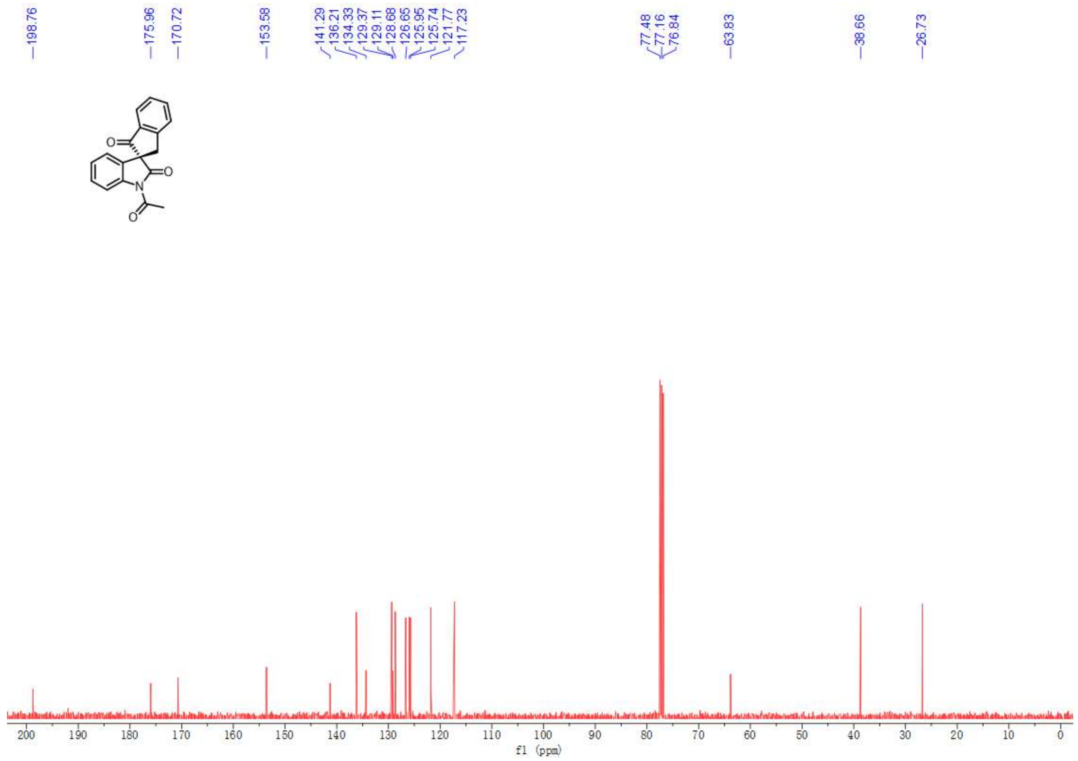

^{1}**H NMR** of **2b**, 400 MHz, $CDCl_3$

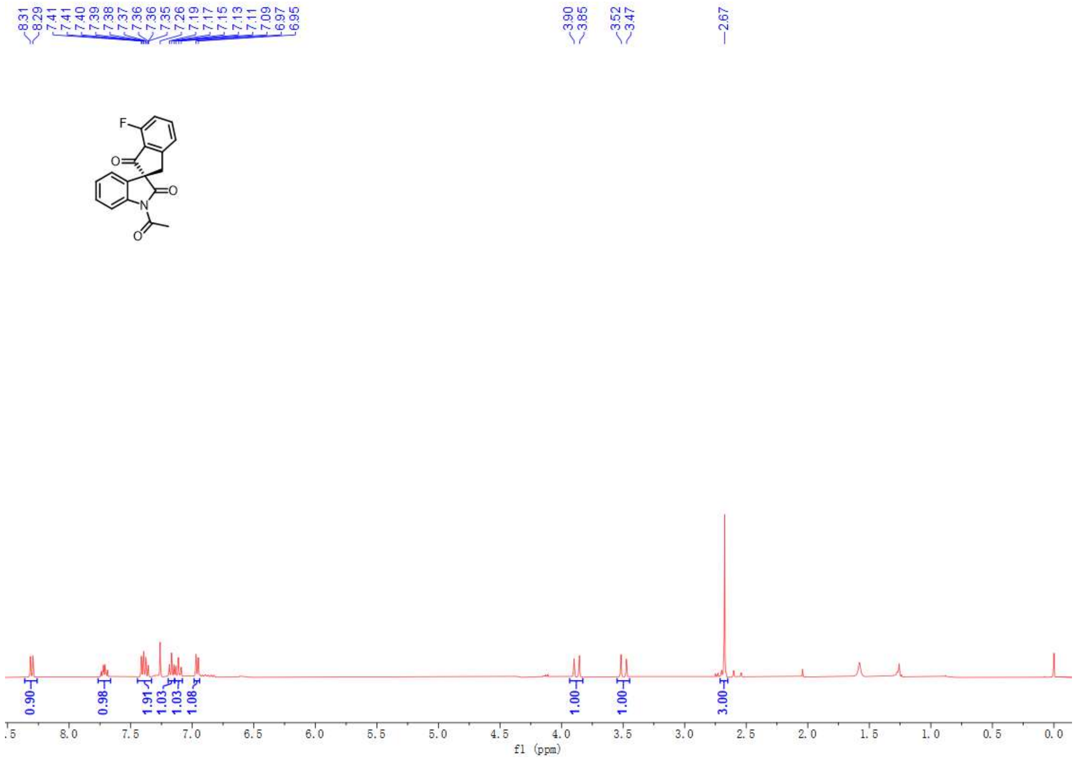

^{13}**C** {^{1}**H**} **NMR** of **2b**, 100 MHz, $CDCl_3$

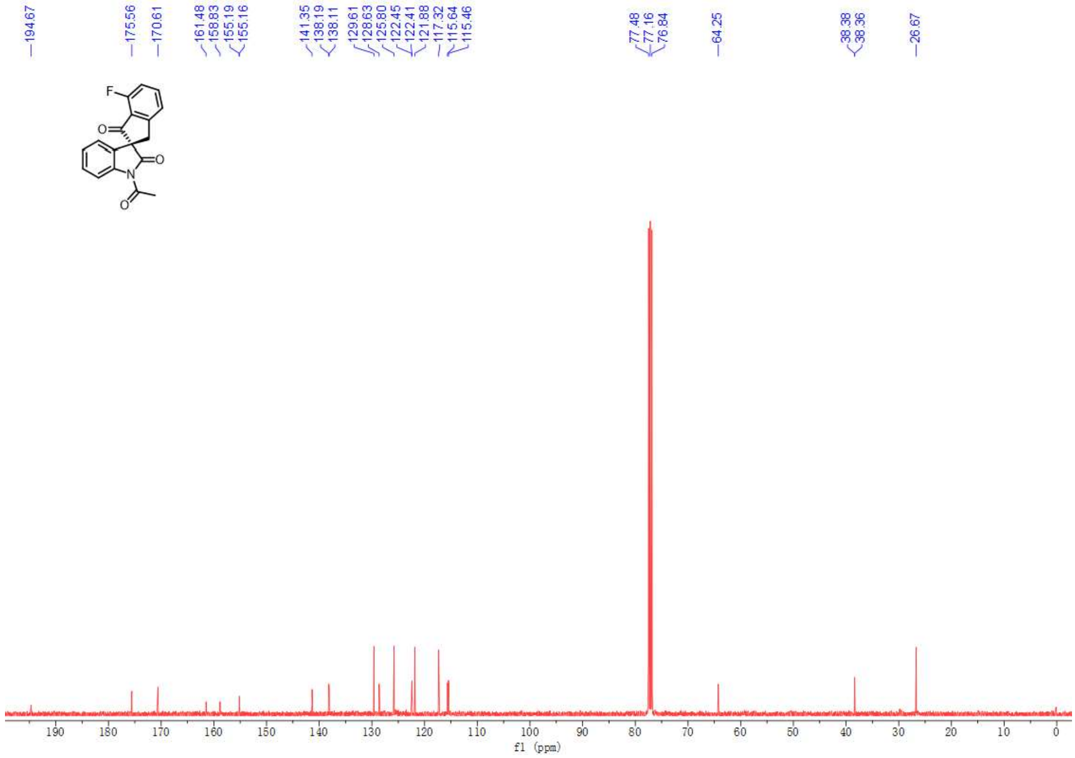

^{19}F NMR of **2b**, 376 MHz, $CDCl_3$

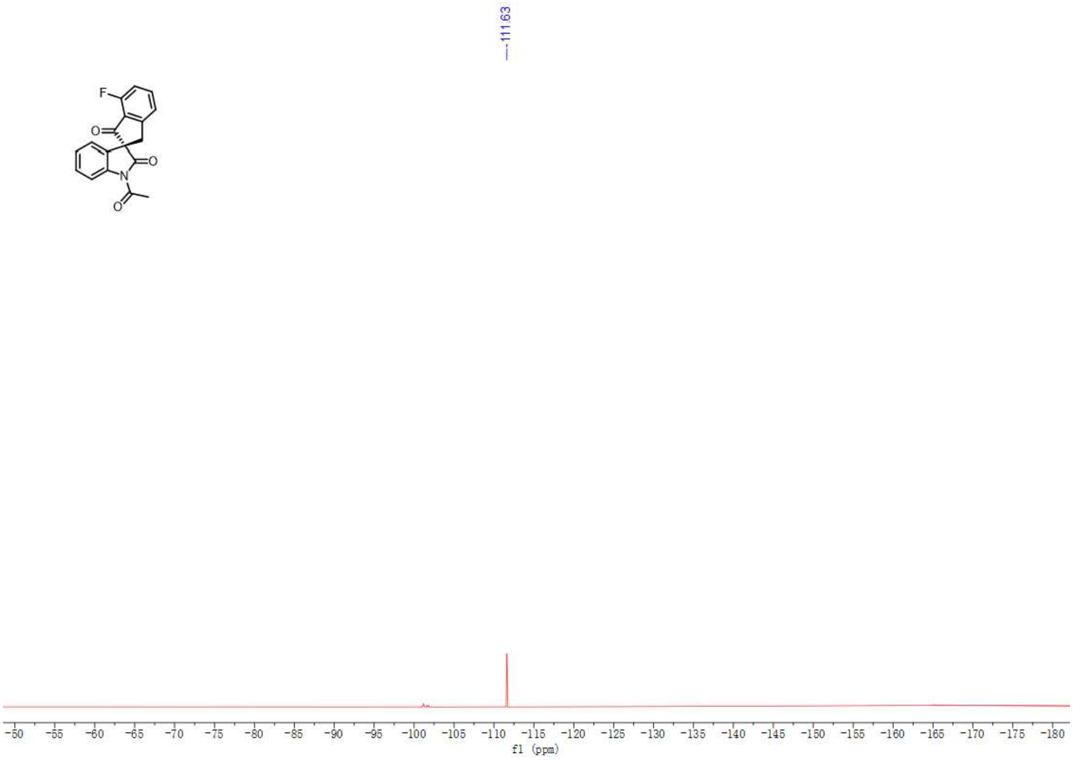

^{1}H NMR of **2c**, 400 MHz, $CDCl_3$

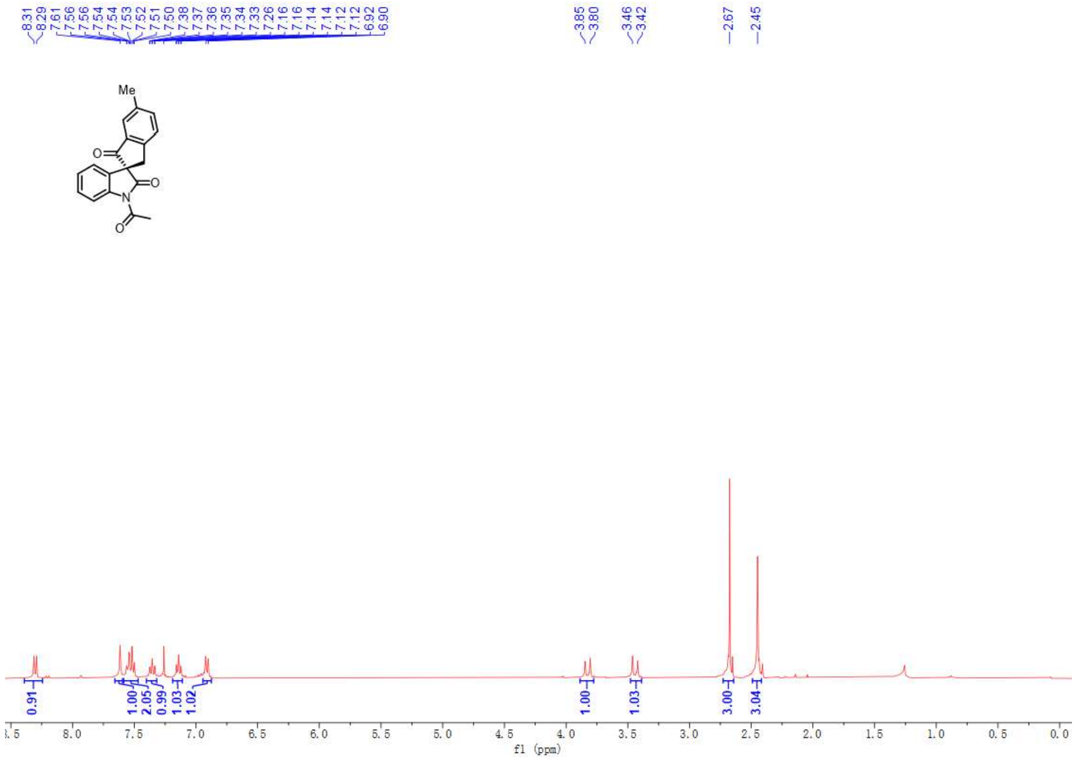

^{13}C {^{1}H} NMR of **2c**, 100 MHz, $CDCl_3$

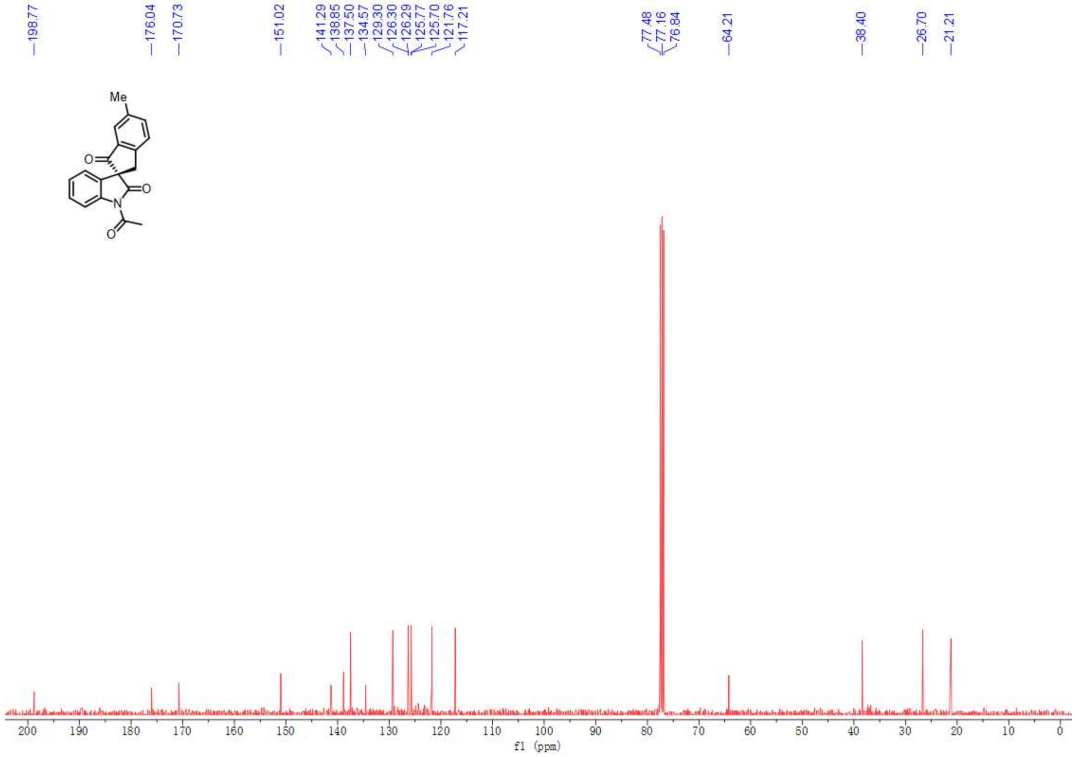

^{1}H NMR of **2d**, 400 MHz, $CDCl_3$

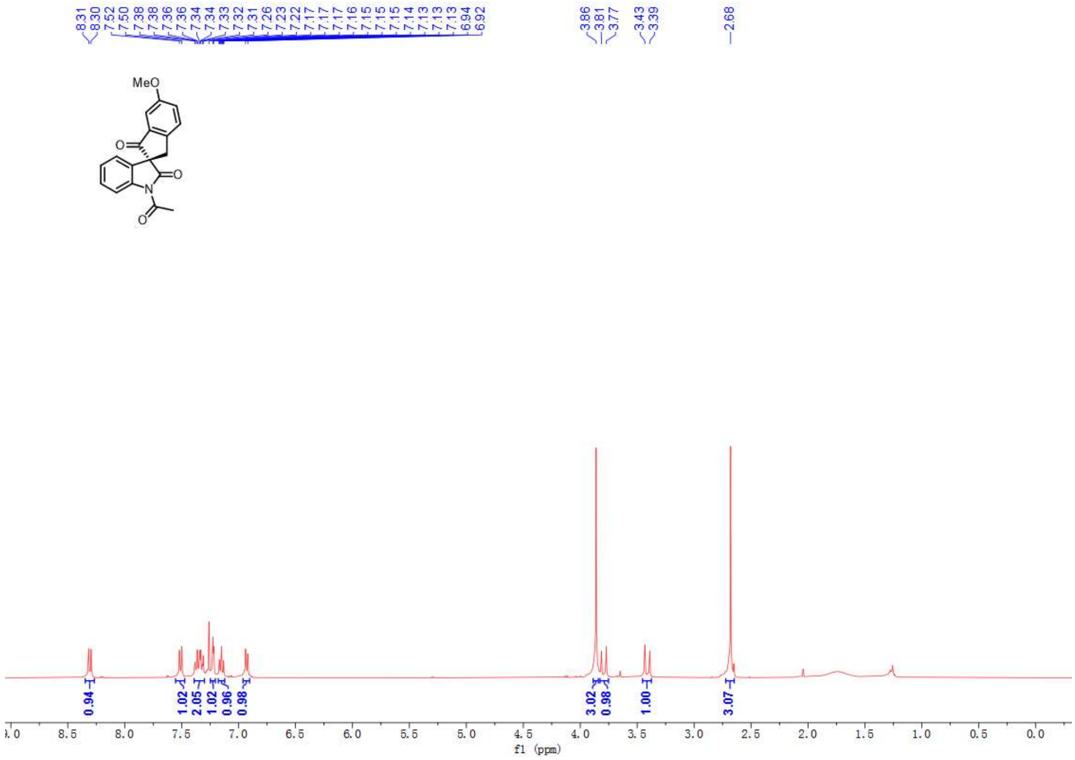

^{13}C {^{1}H} NMR of **2d**, 100 MHz, $CDCl_3$

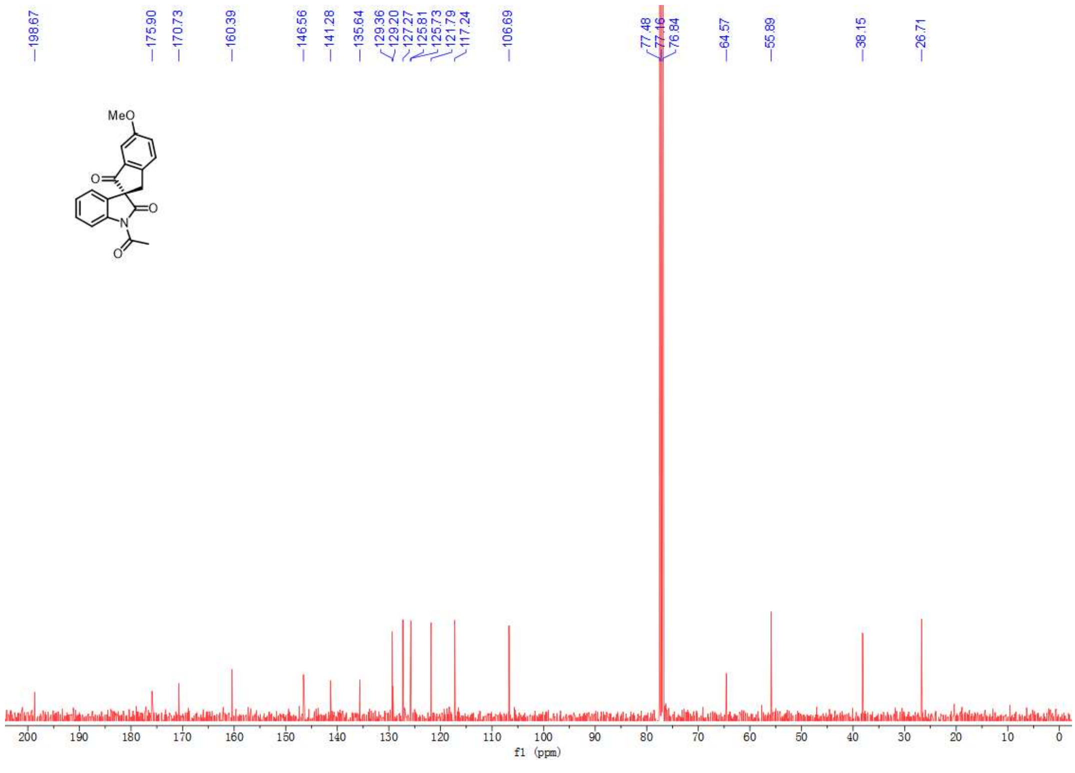

^{1}H NMR of **2e**, 400 MHz, $CDCl_3$

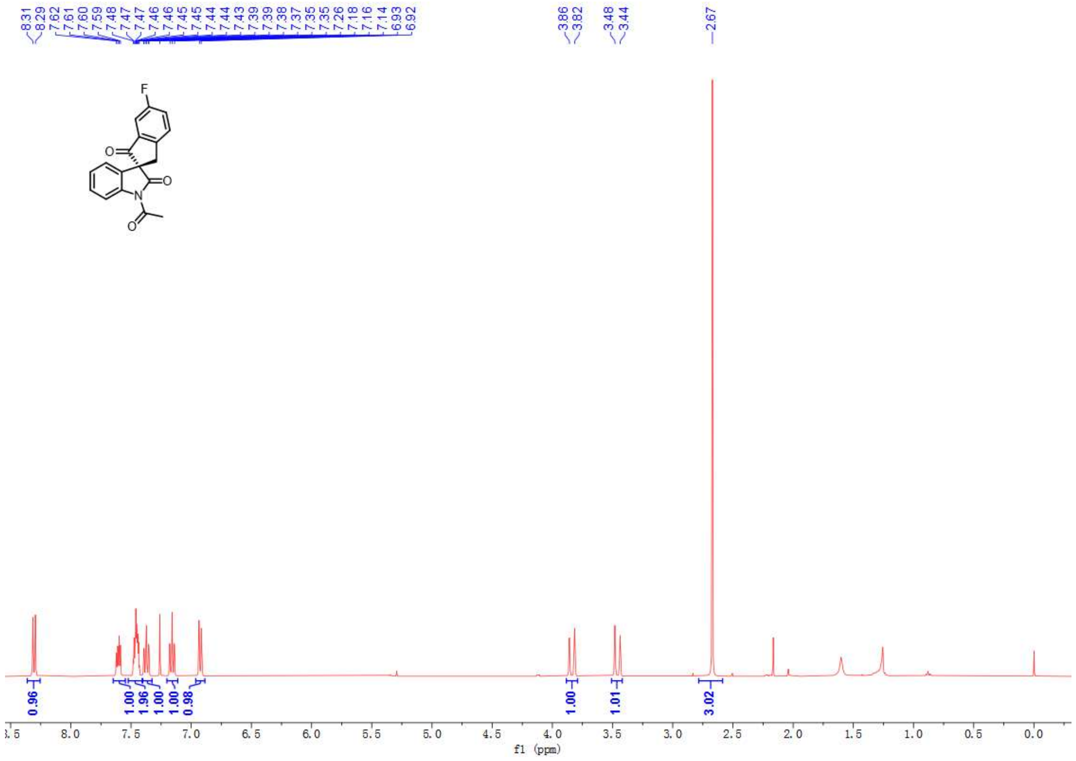

^{13}C {^{1}H} NMR of **2e**, 100 MHz, $CDCl_3$

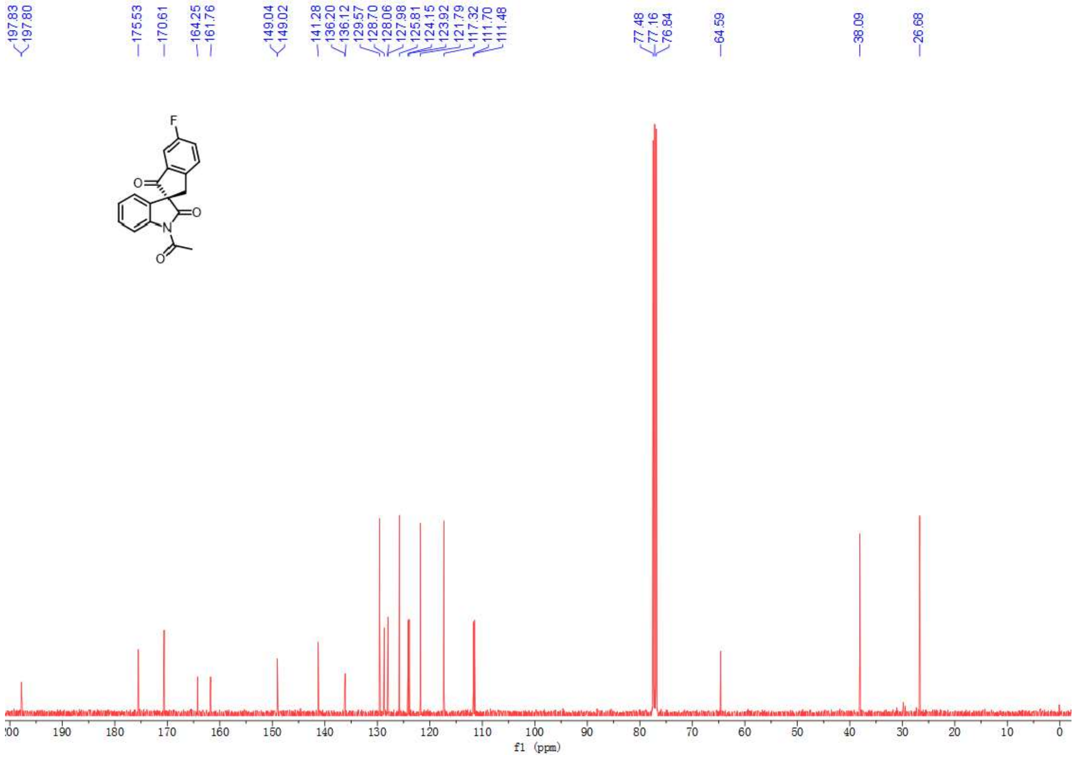

^{19}F NMR of **2e**, 376 MHz, $CDCl_3$

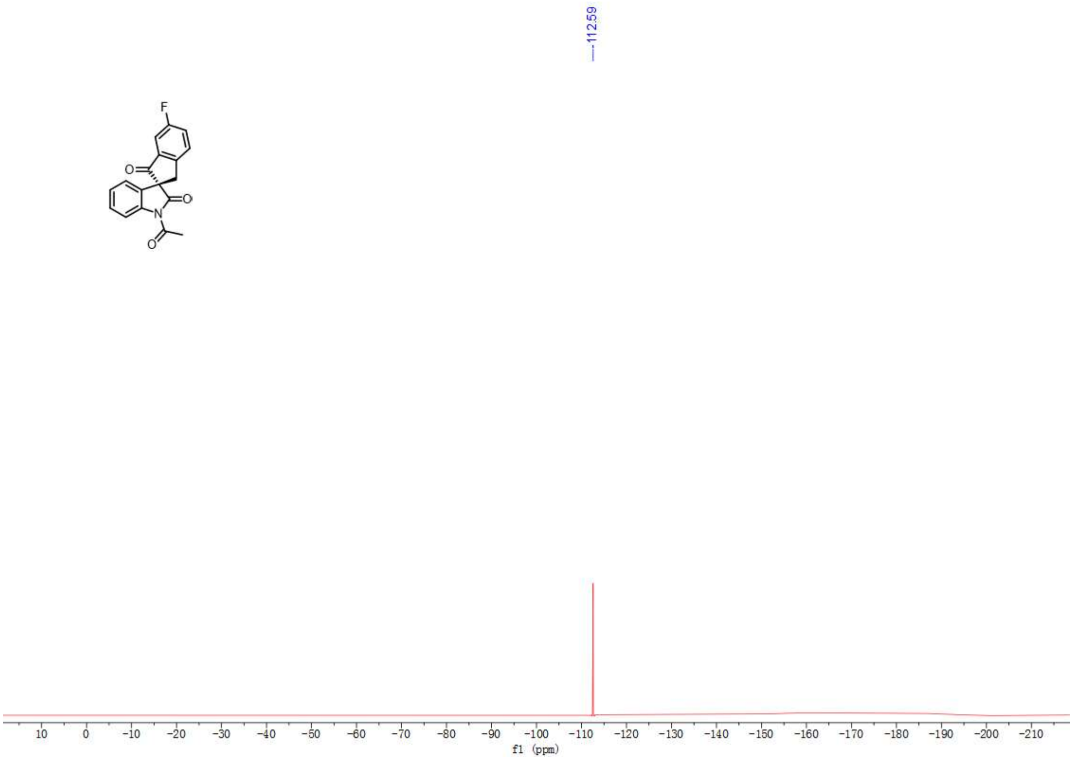

^{1}H NMR of **2f**, 400 MHz, $CDCl_3$

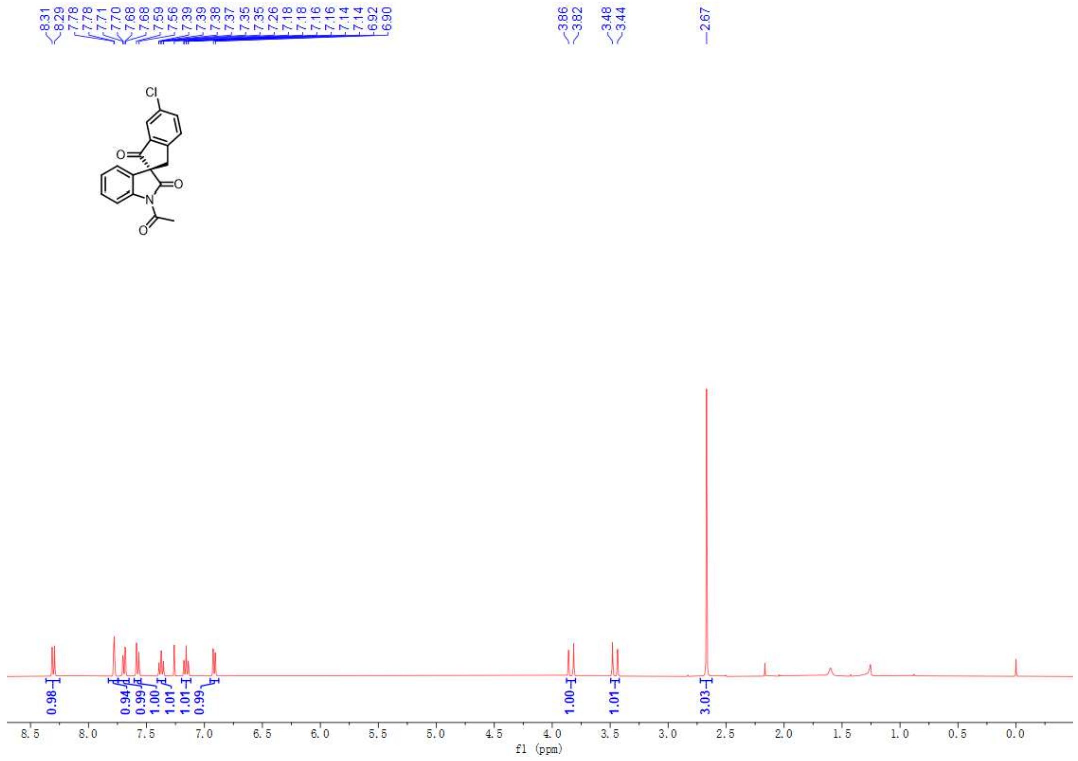

^{13}C {^{1}H} NMR of **2f**, 100 MHz, $CDCl_3$

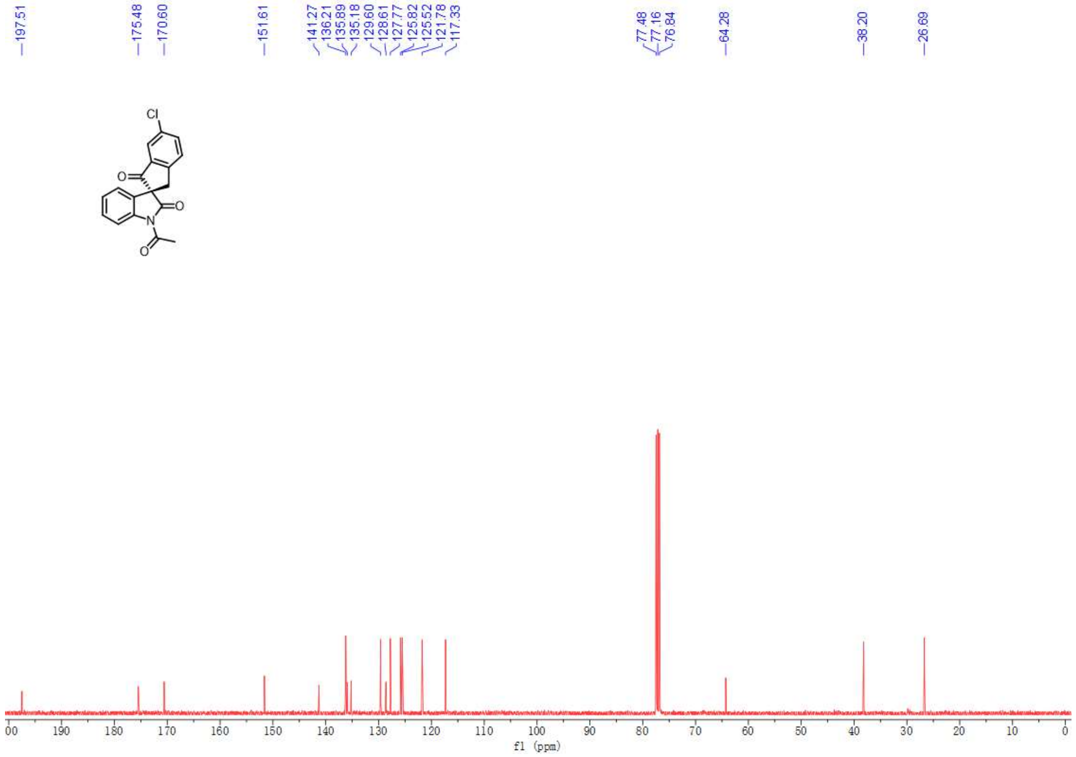

^{1}H NMR of **2g**, 400 MHz, $CDCl_3$

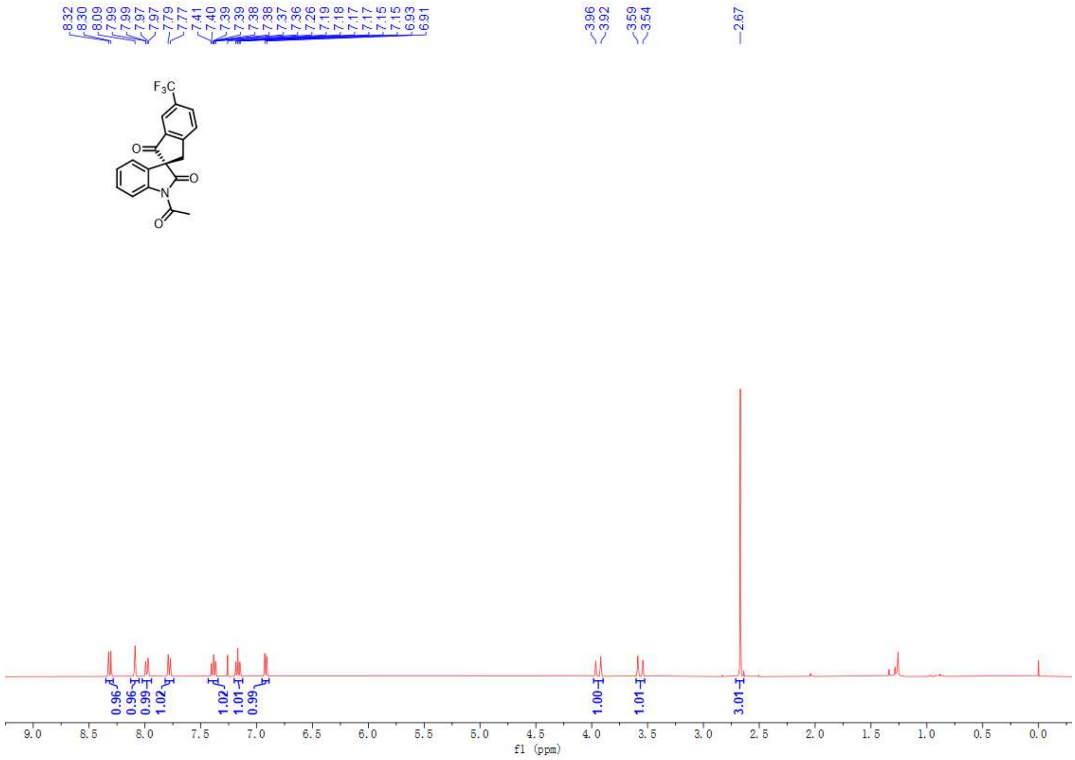

^{13}C {^{1}H} NMR of **2g**, 100 MHz, $CDCl_3$

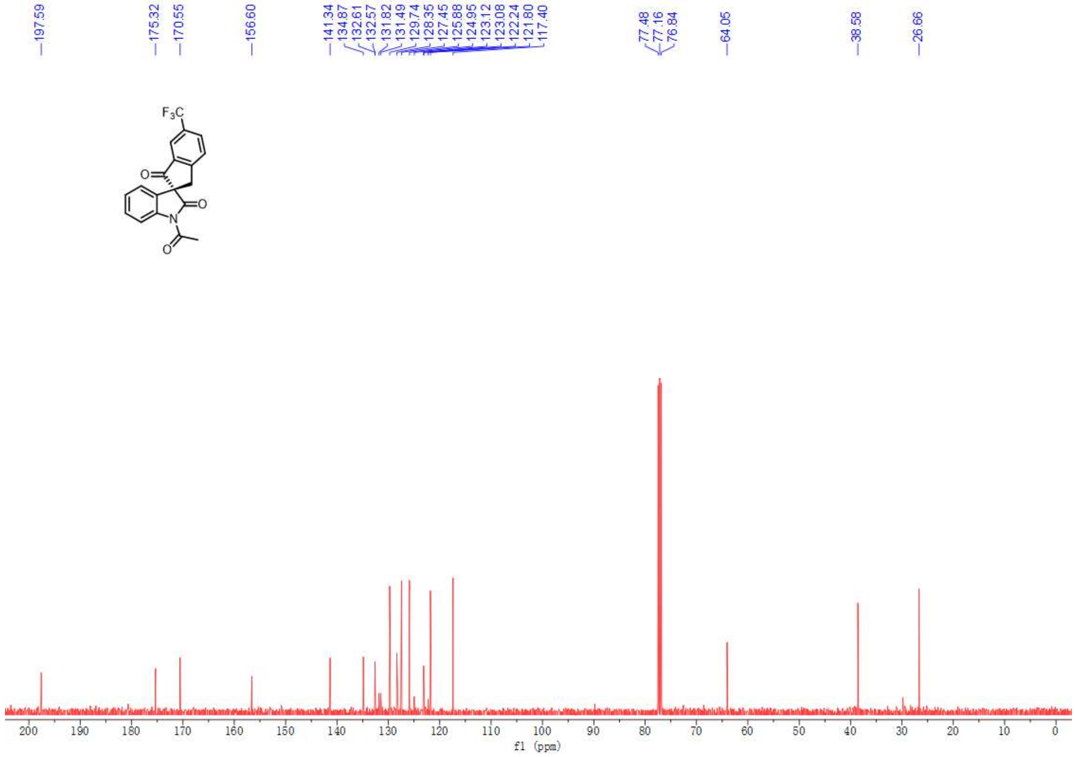

^{19}F NMR of **2g**, 376 MHz, $CDCl_3$

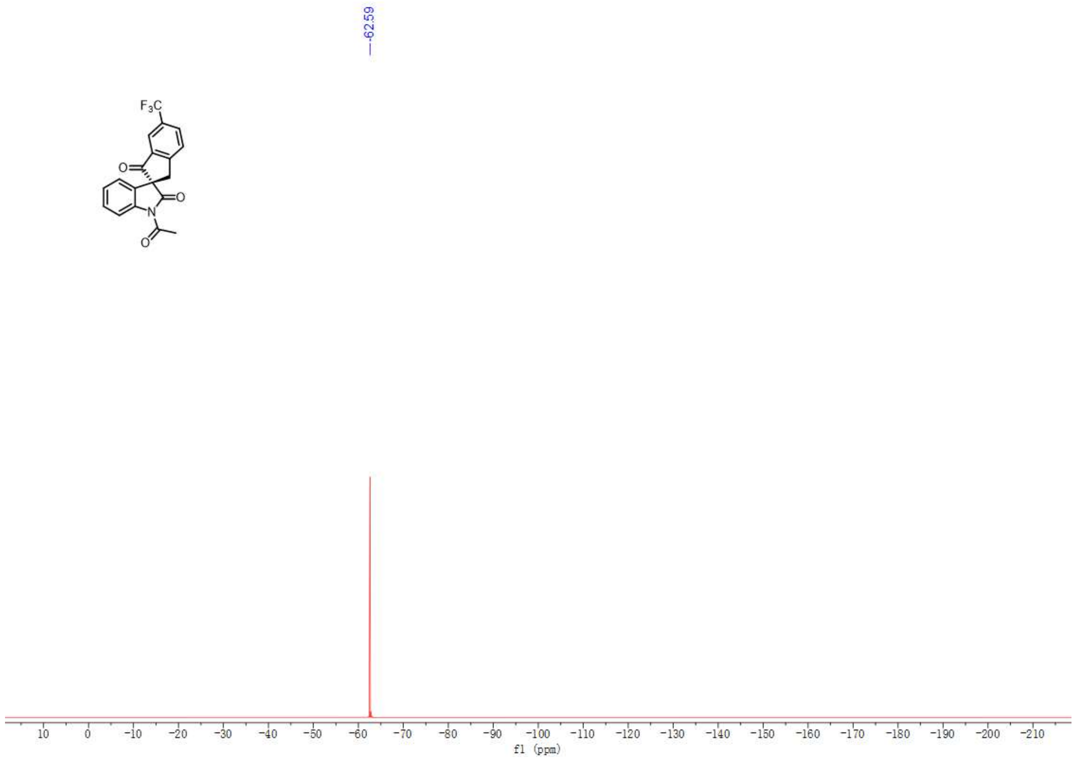

^{1}H NMR of **2h**, 400 MHz, $CDCl_3$

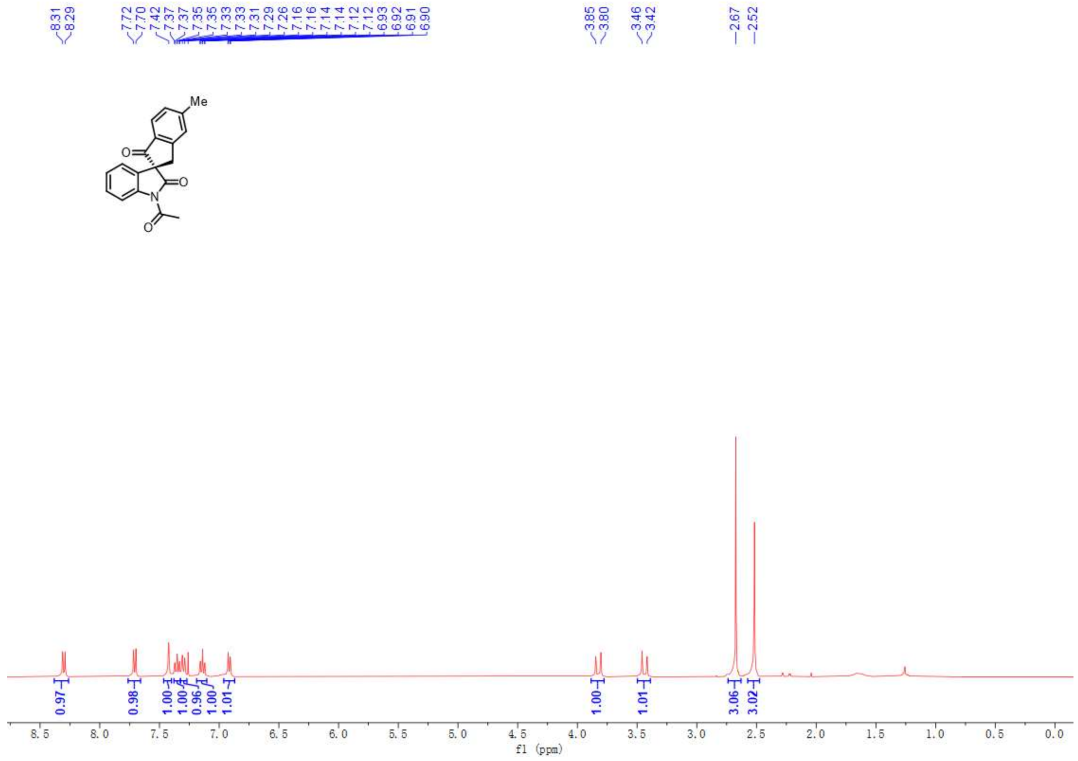

^{13}C {^{1}H} NMR of **2h**, 100 MHz, $CDCl_3$

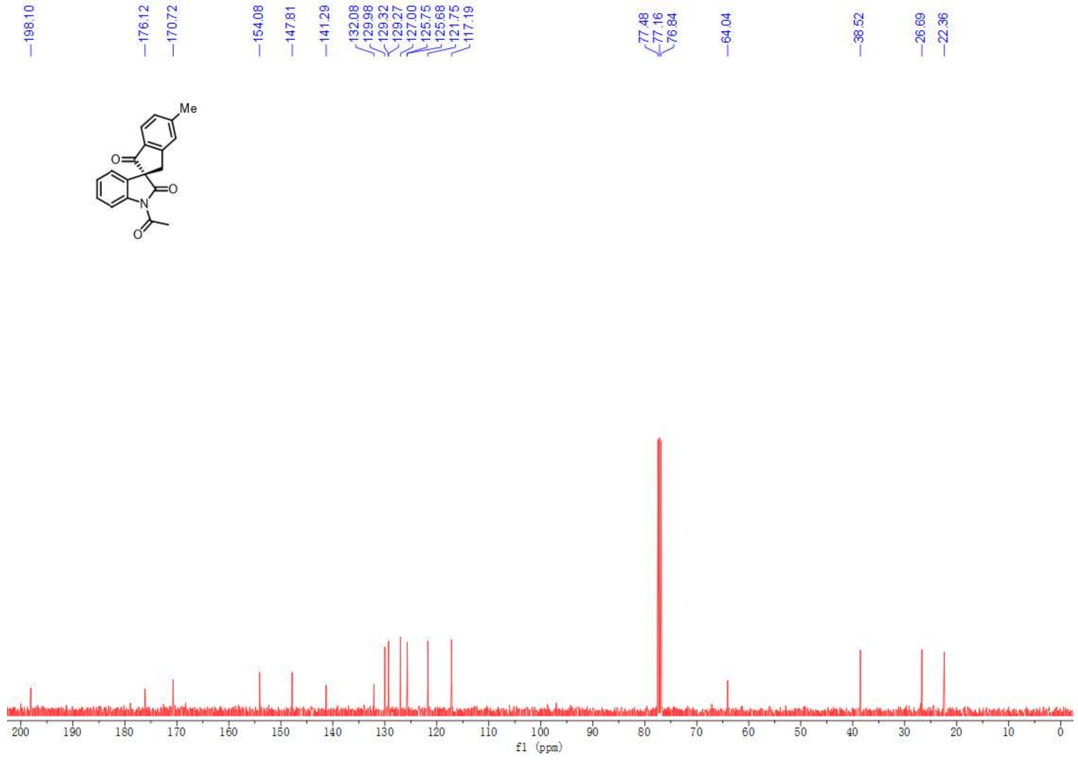

^{1}**H NMR** of **2i**, 400 MHz, $CDCl_3$

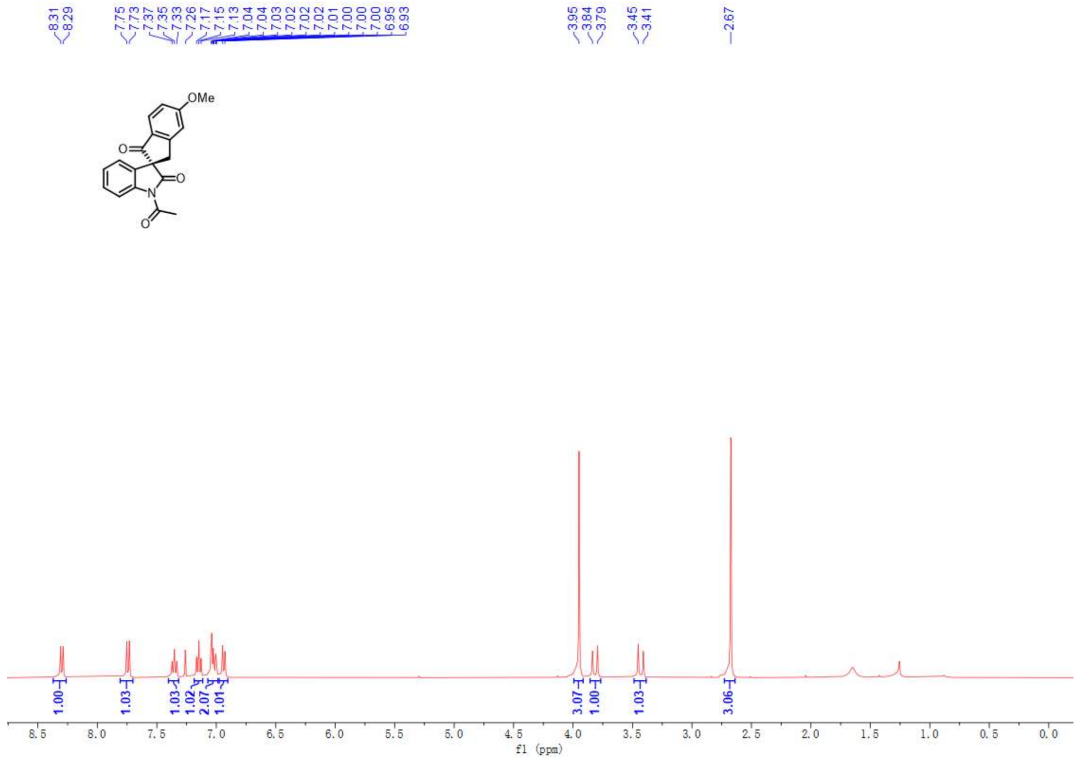

^{13}**C** {^{1}**H**} **NMR** of **2i**, 100 MHz, $CDCl_3$

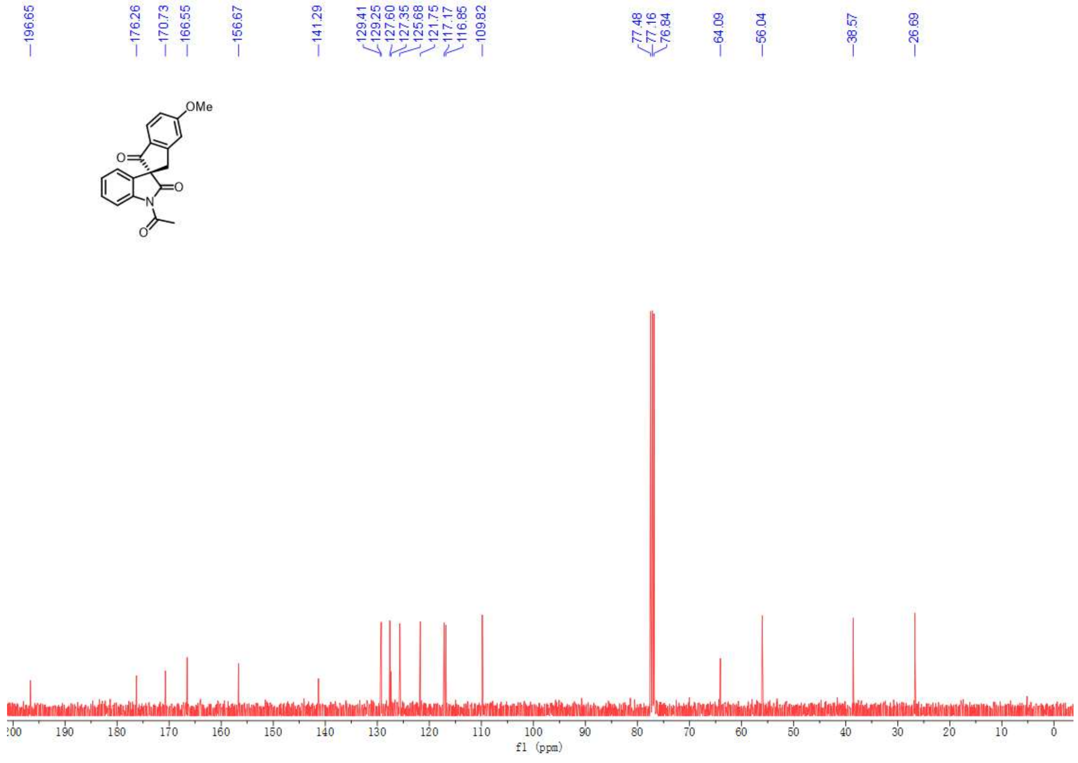

^{1}H NMR of **2j**, 400 MHz, CDCl$_3$

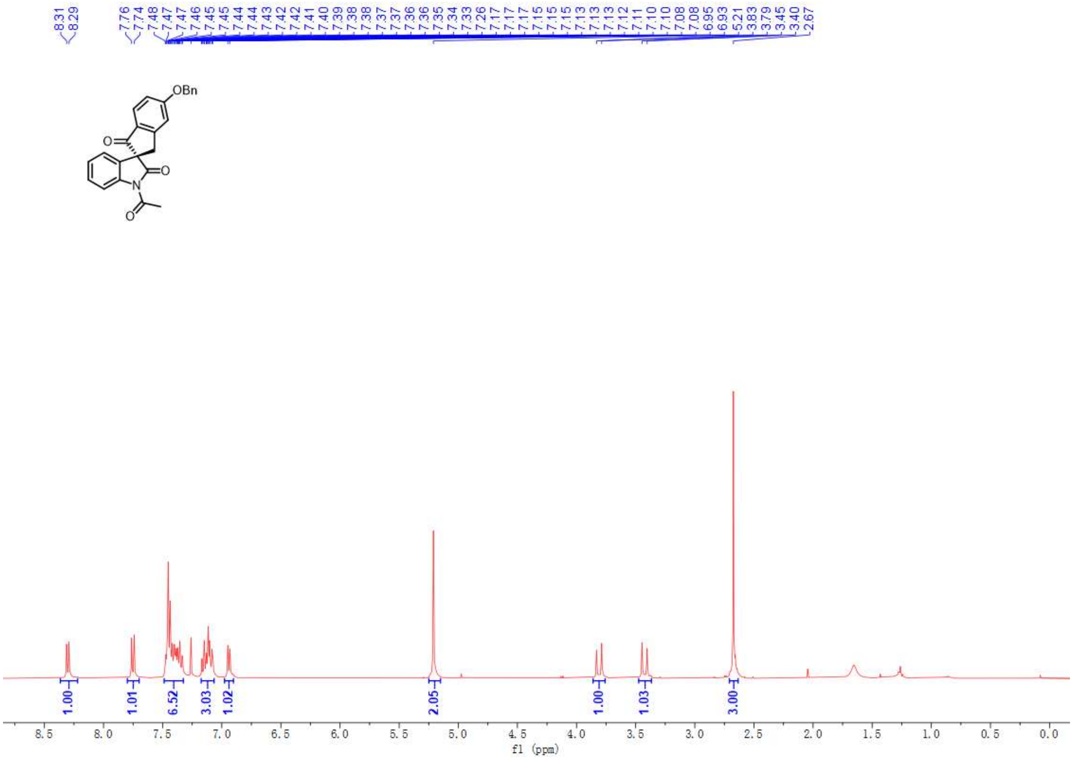

^{13}C {^{1}H} NMR of **2j**, 100 MHz, CDCl$_3$

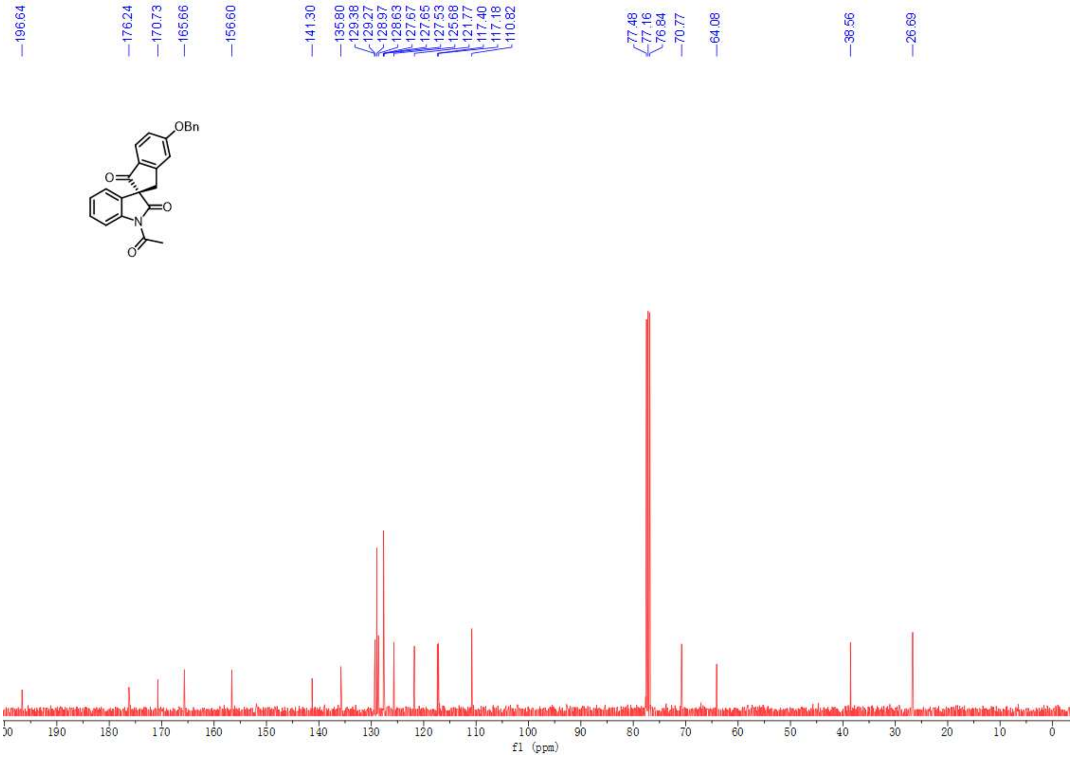

^{1}H NMR of **2k**, 400 MHz, $CDCl_3$

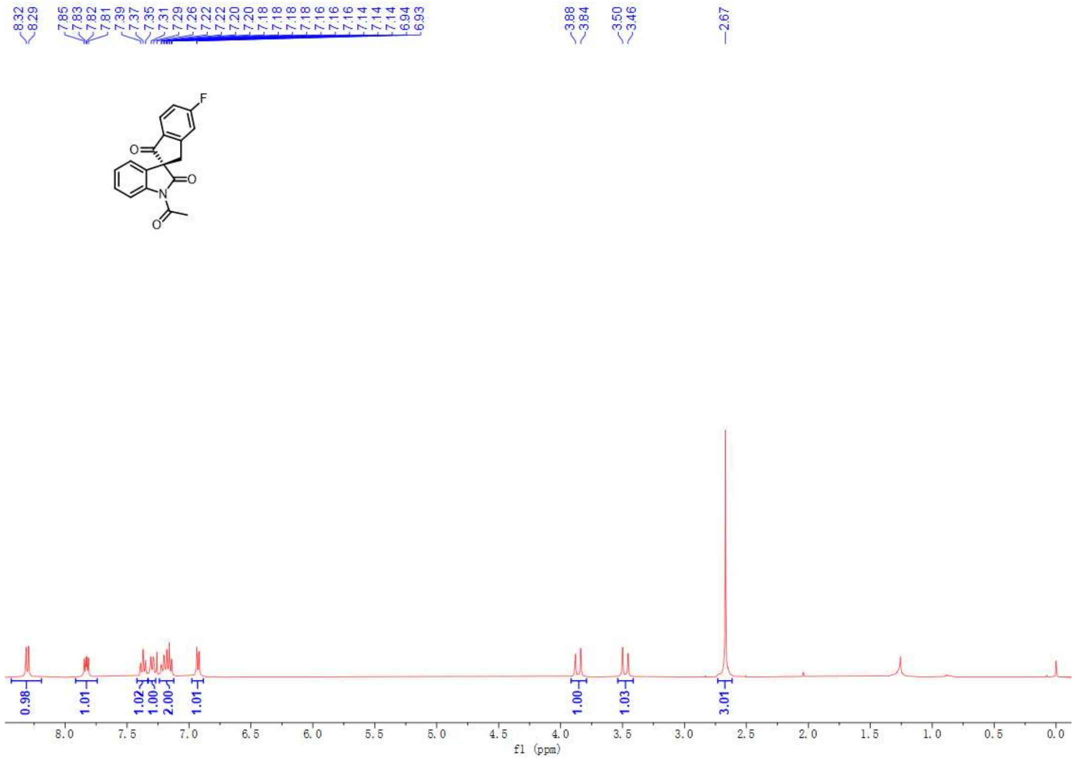

^{13}C {^{1}H} NMR of **2k**, 100 MHz, $CDCl_3$

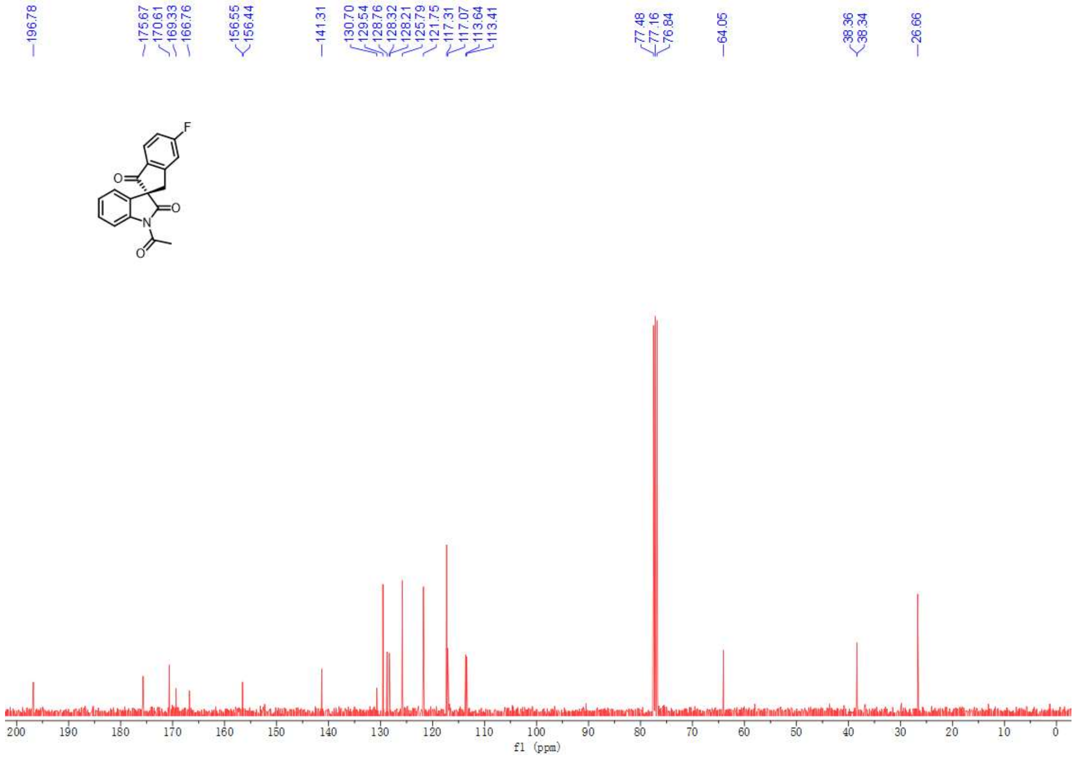

^{19}F NMR of **2k**, 376 MHz, $CDCl_3$

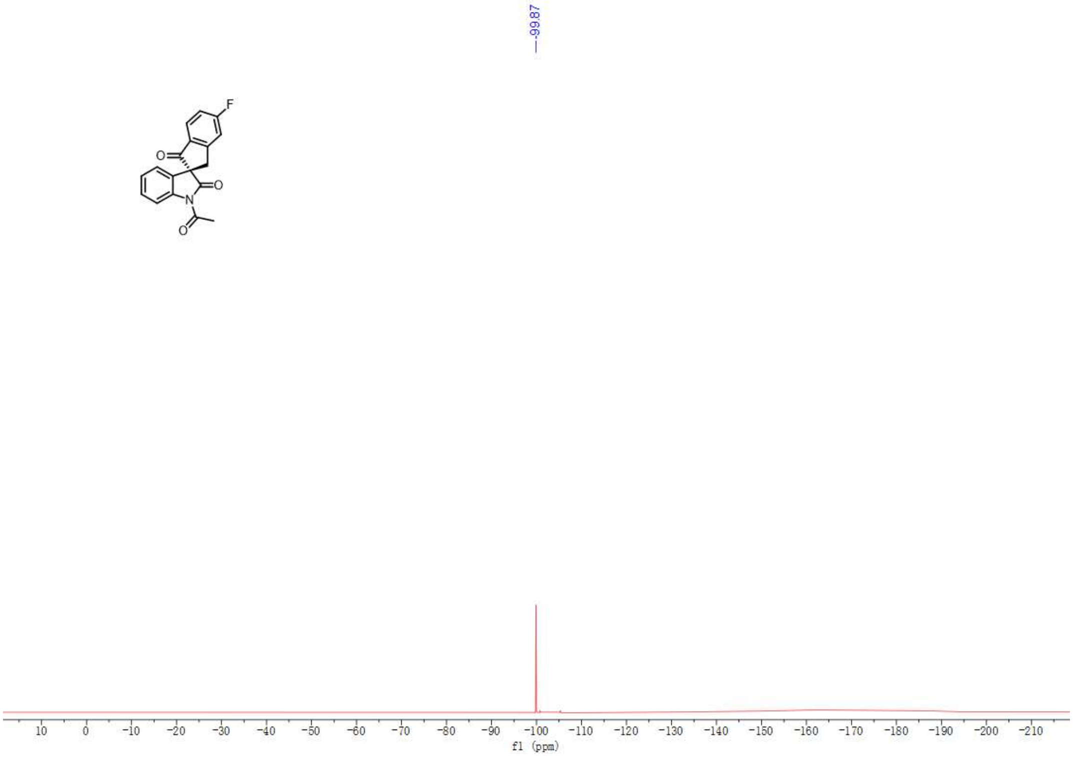

^{1}H NMR of **2l**, 400 MHz, $CDCl_3$

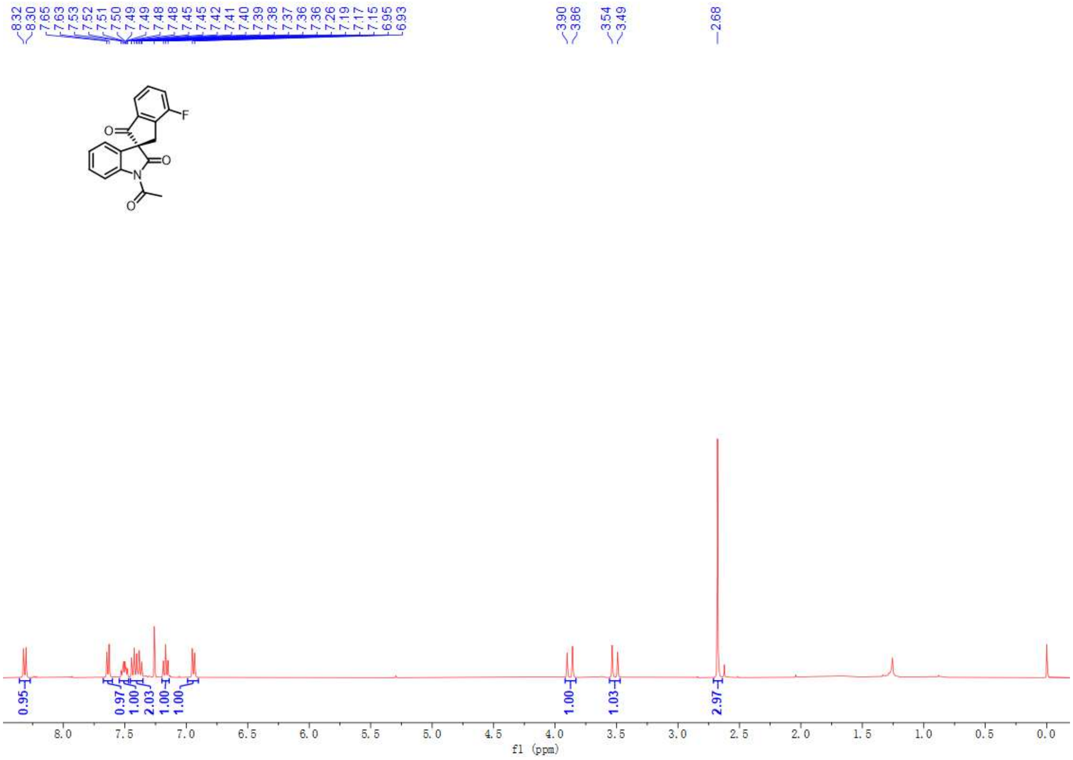

^{13}C {^{1}H} NMR of **2l**, 100 MHz, $CDCl_3$

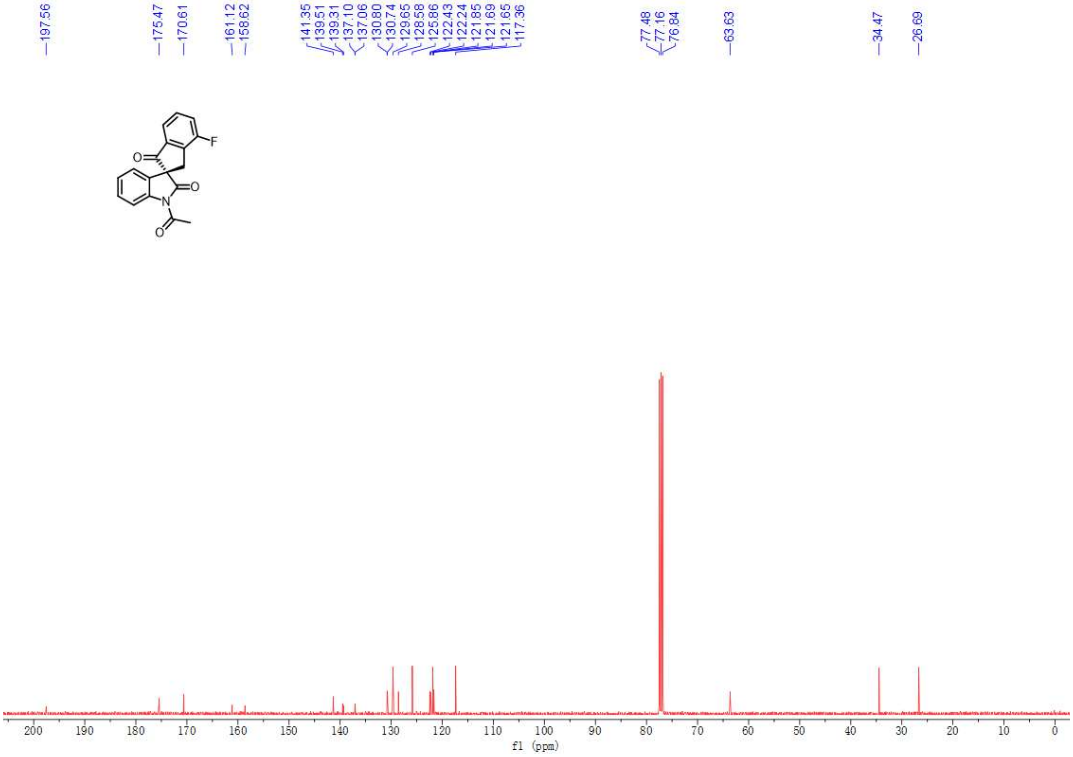

^{19}F NMR of **2l**, 376 MHz, $CDCl_3$

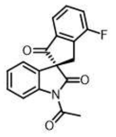

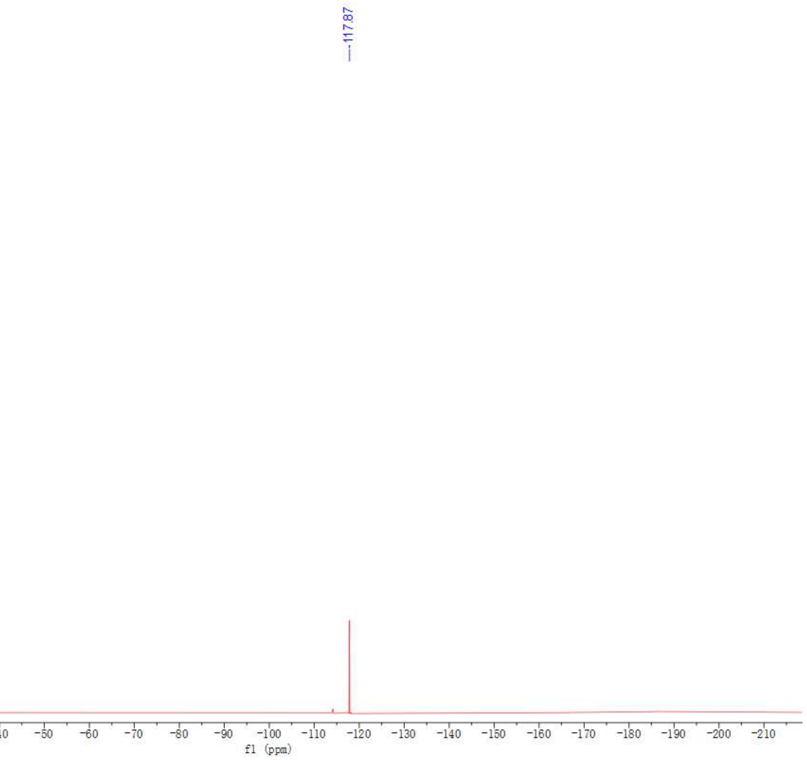

^{1}H NMR of **2m**, 400 MHz, $CDCl_3$

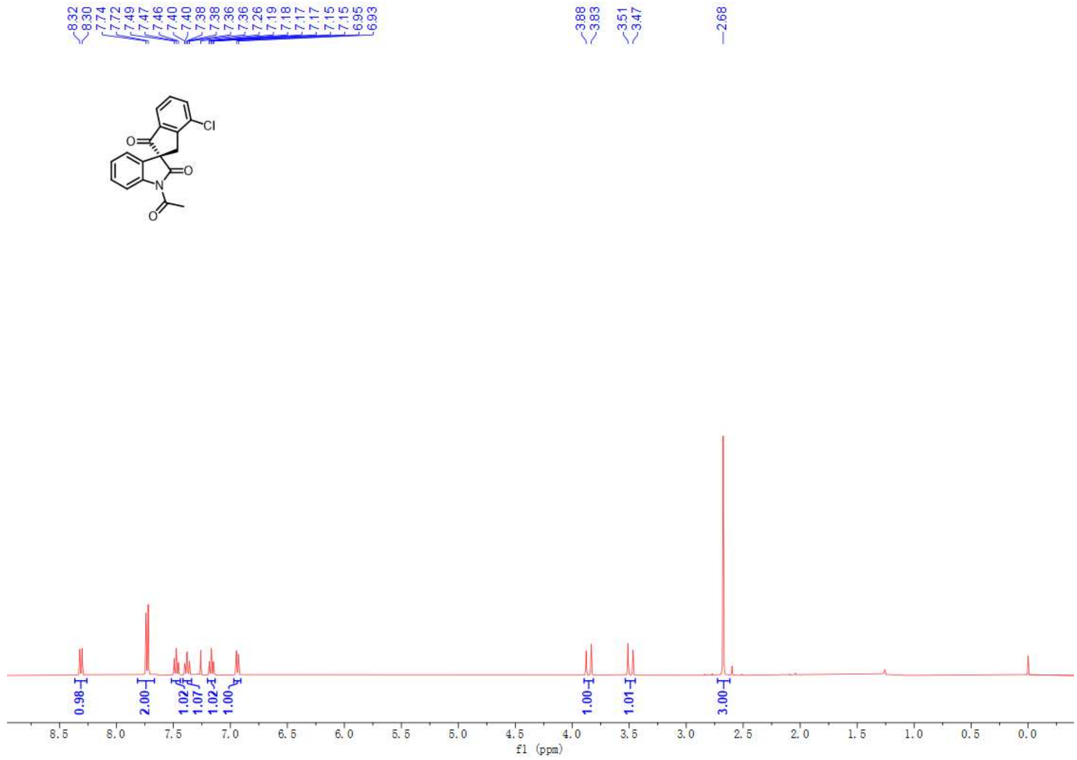

^{13}C {^{1}H} NMR of **2m**, 100 MHz, $CDCl_3$

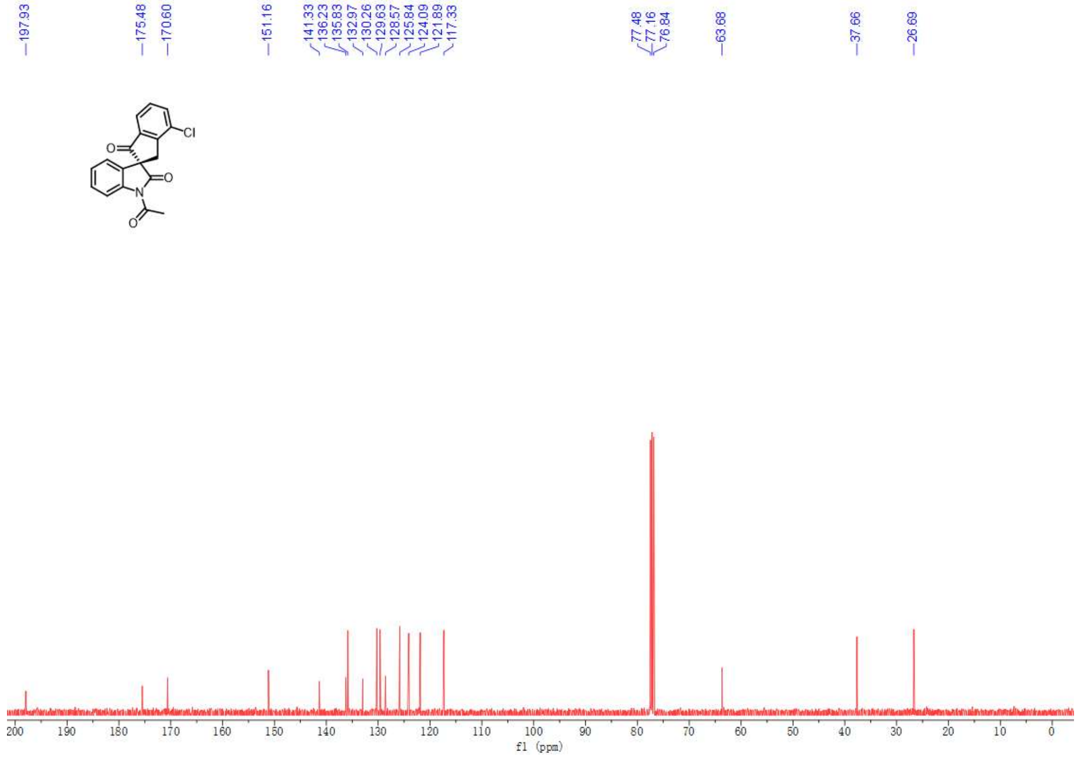

^{1}H NMR of **2n**, 400 MHz, DMSO-d_6

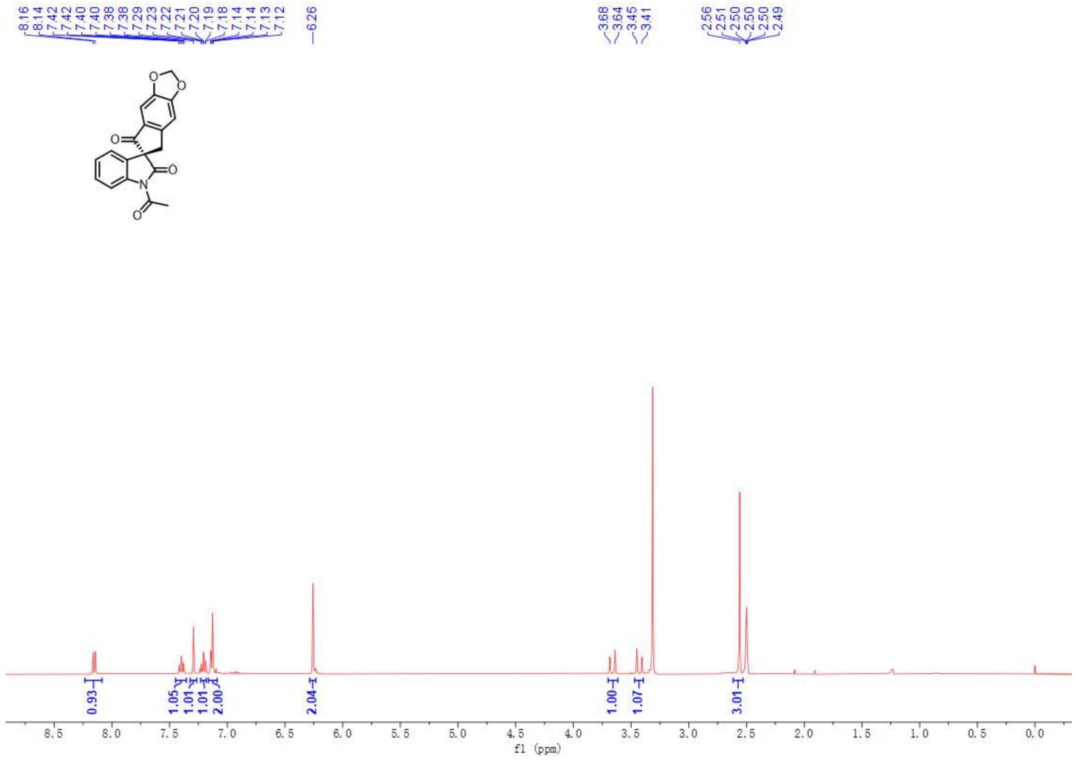

^{13}C {^{1}H} NMR of **2n**, 100 MHz, DMSO-d_6

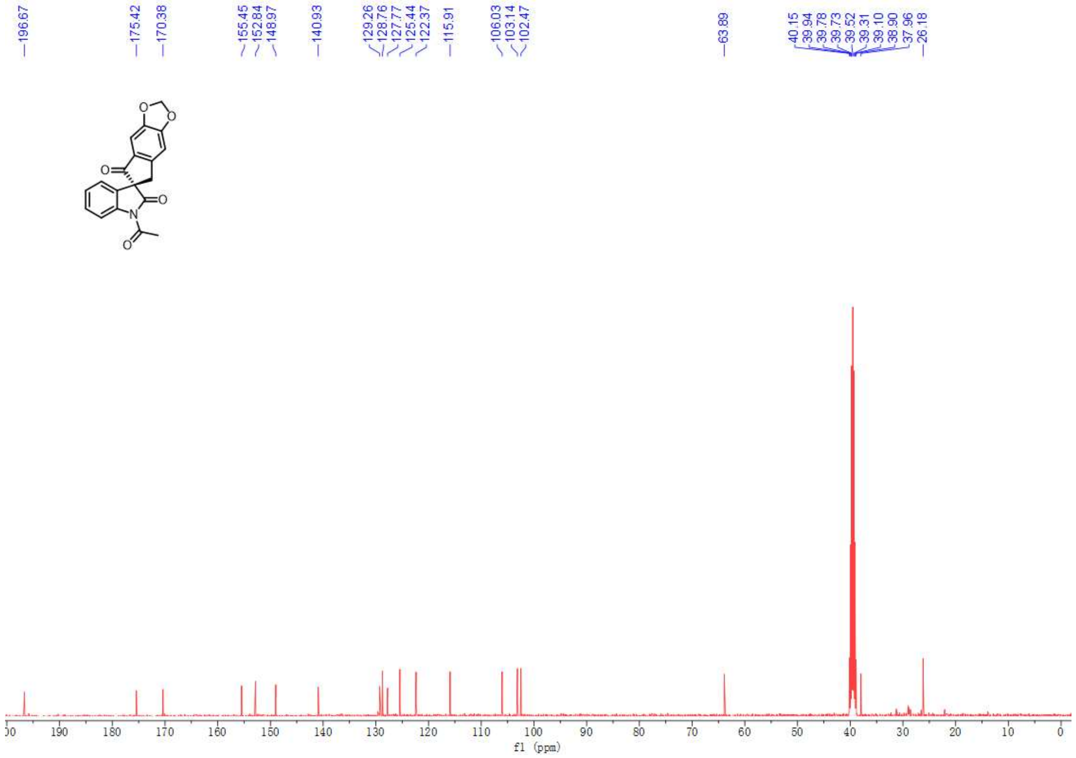

^{1}H NMR of **2o**, 400 MHz, $CDCl_3$

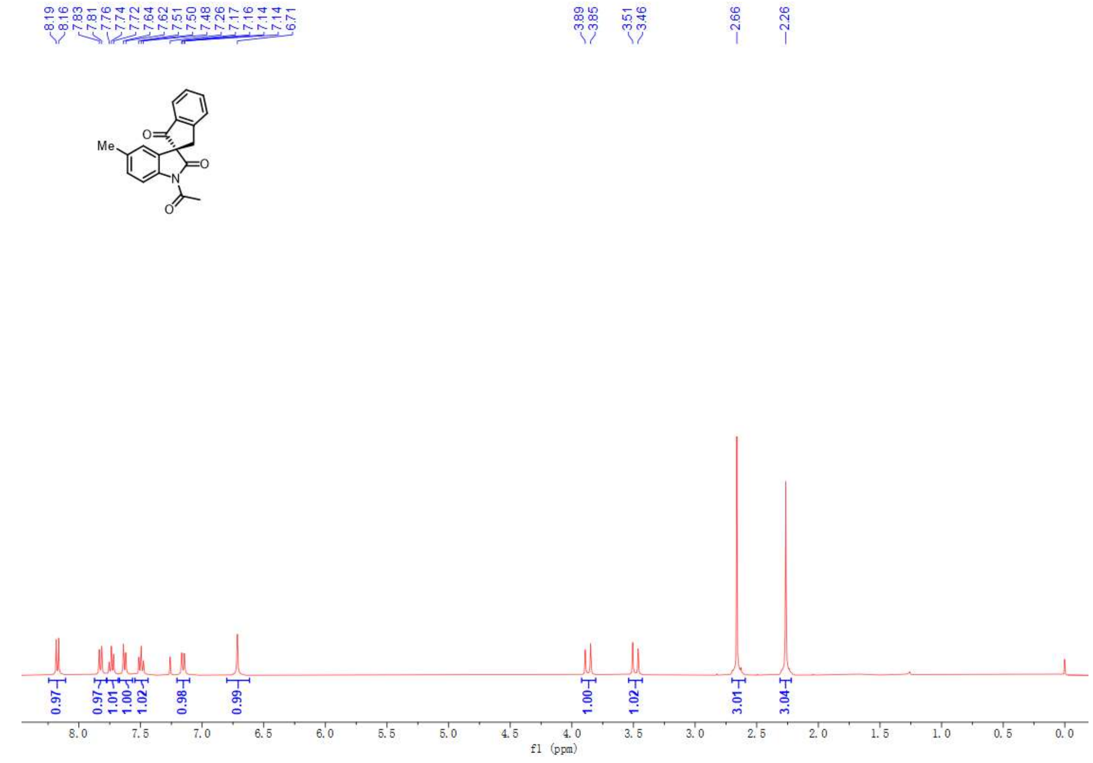

^{13}C {^{1}H} NMR of **2o**, 100 MHz, $CDCl_3$

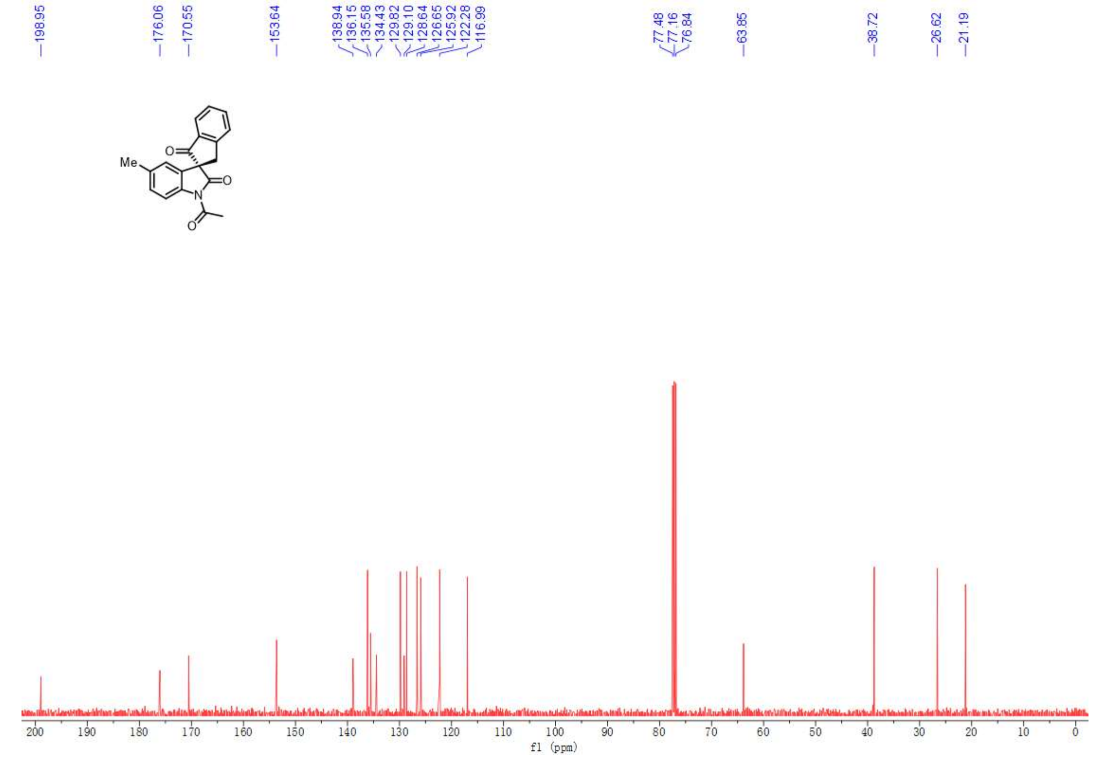

^{1}H NMR of **2p**, 400 MHz, $CDCl_3$

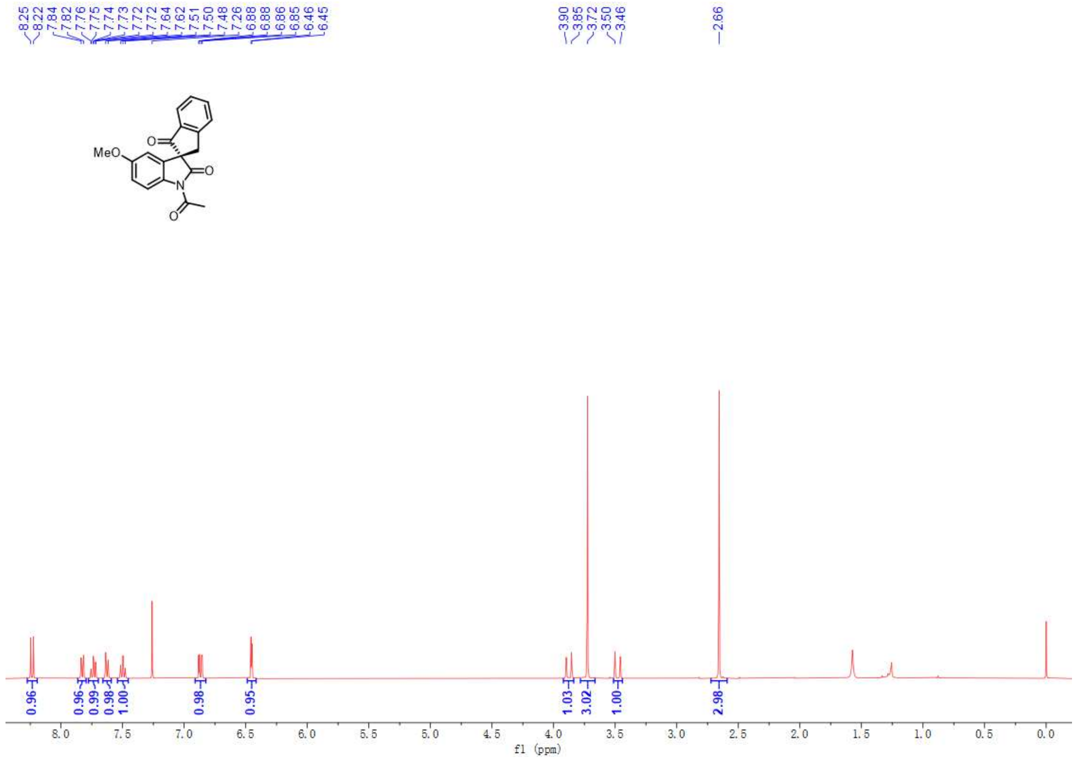

^{13}C {^{1}H} NMR of **2p**, 100 MHz, $CDCl_3$

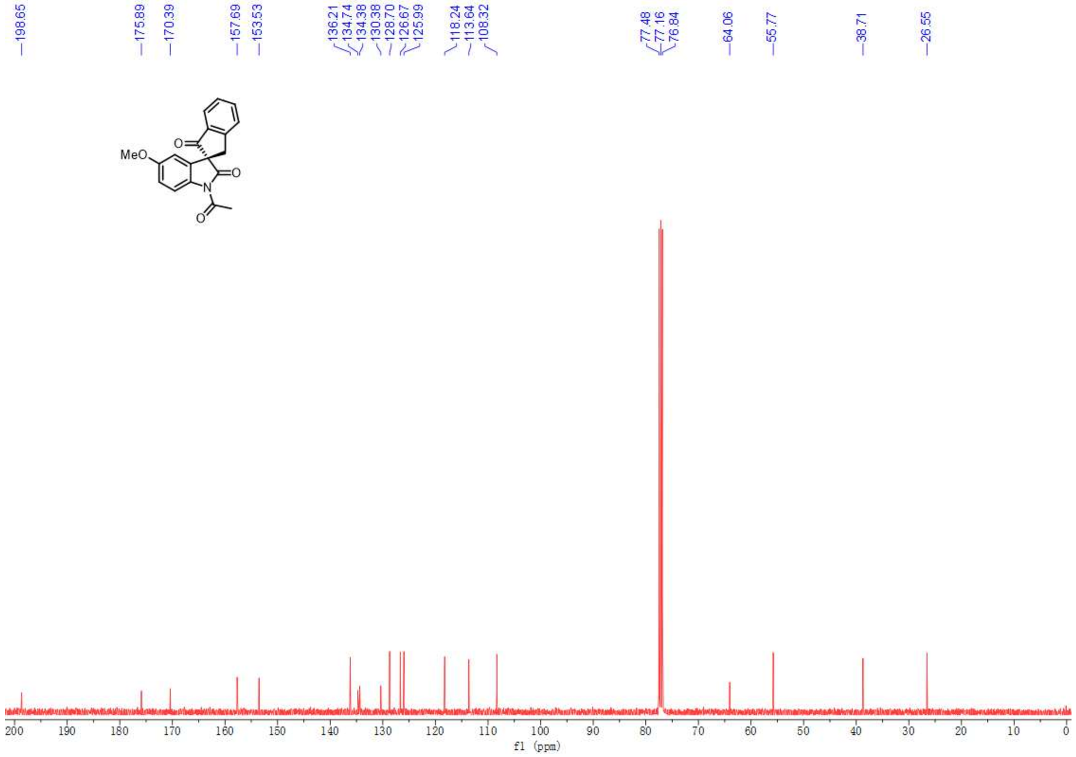

^{1}H NMR of **2q**, 400 MHz, $CDCl_3$

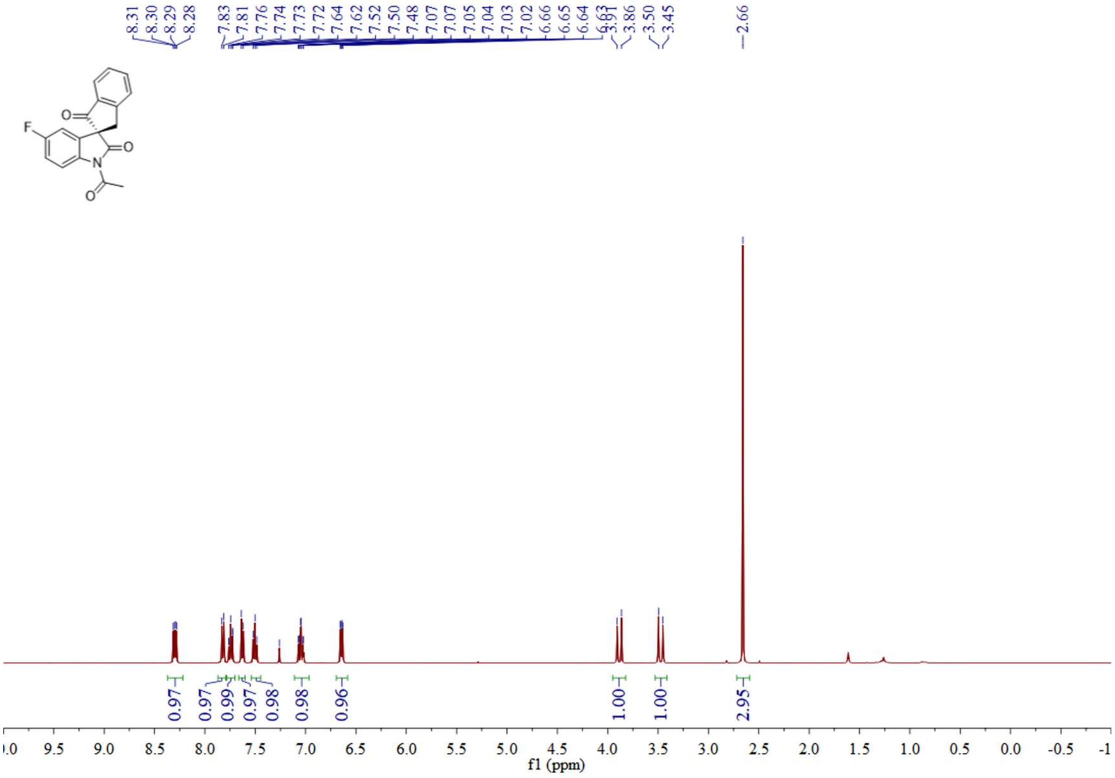

^{13}C {^{1}H} NMR of **2q**, 100 MHz, $CDCl_3$

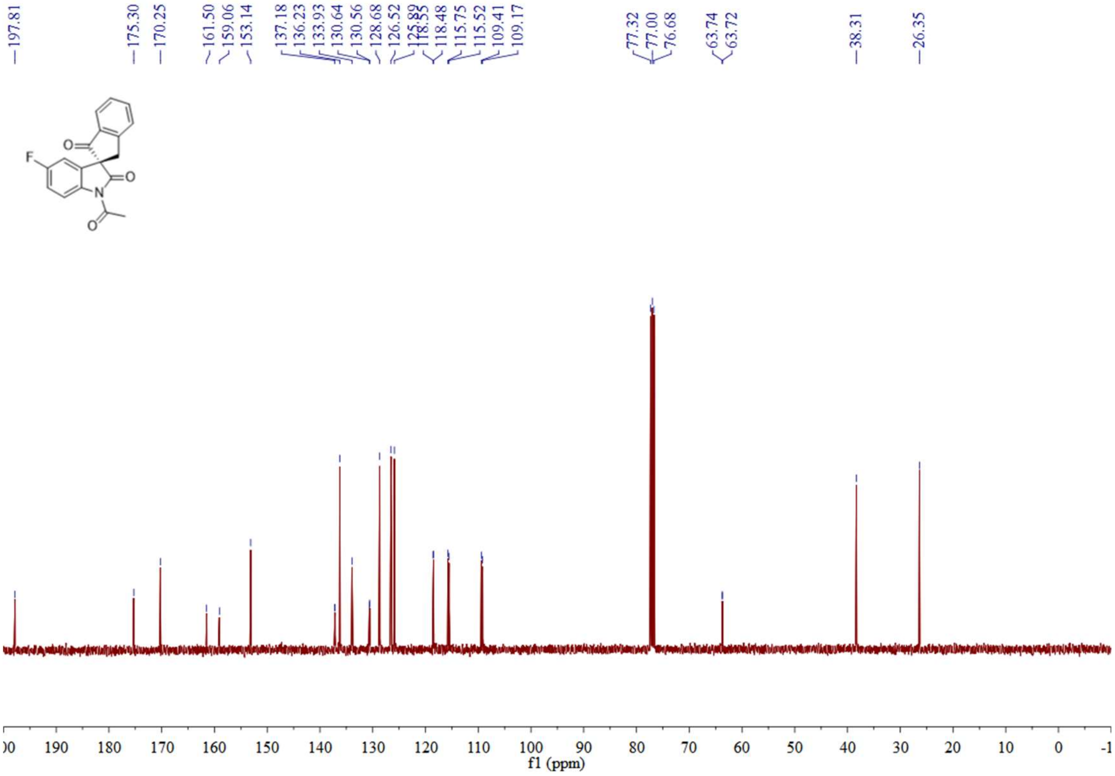

^{19}F NMR of **2q**, 376 MHz, $CDCl_3$

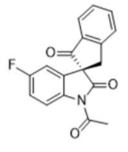

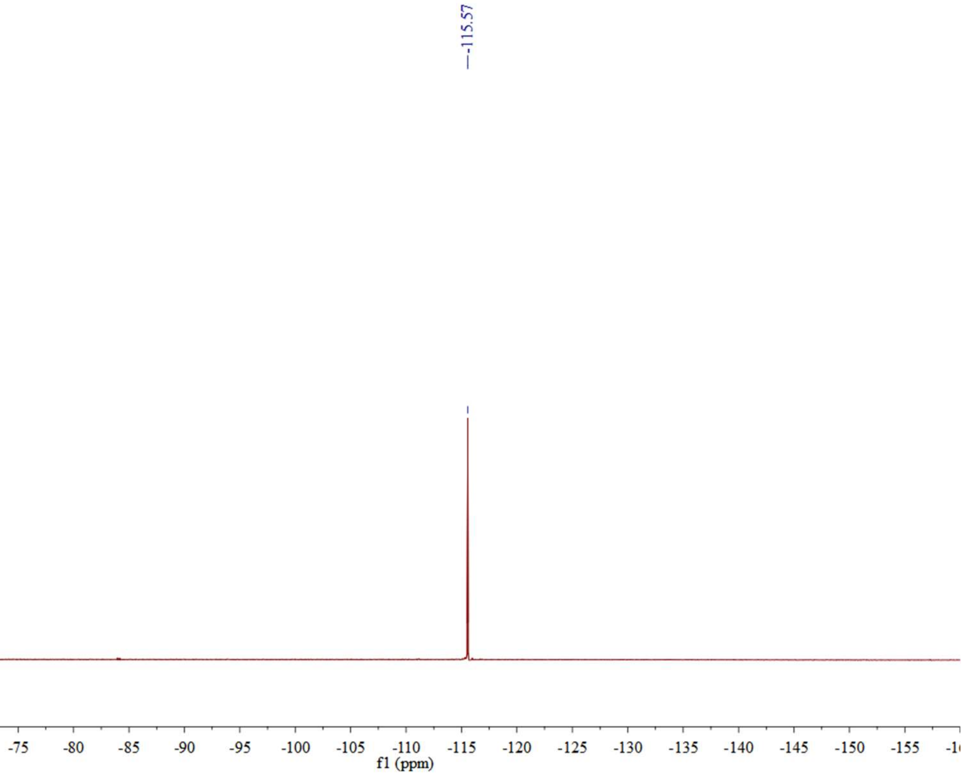

^{1}H NMR of **2r**, 400 MHz, $CDCl_3$

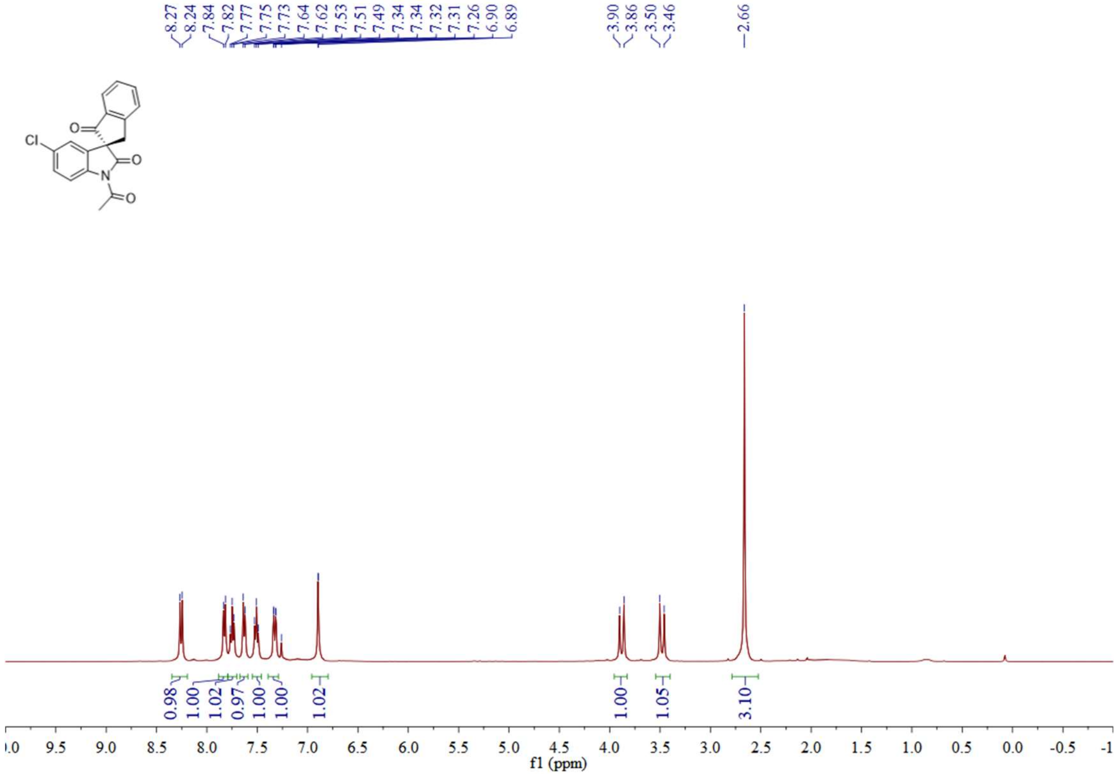

^{13}C {^{1}H} NMR of **2r**, 100 MHz, $CDCl_3$

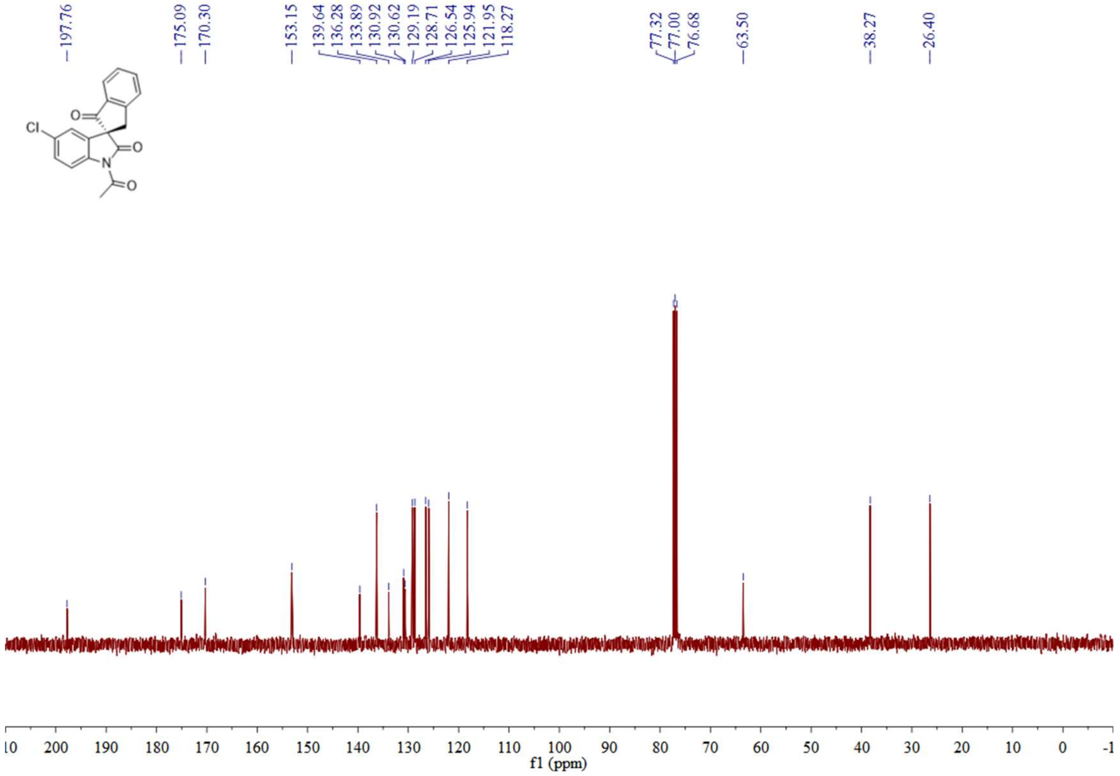

^{1}H NMR of **2s**, 400 MHz, $CDCl_3$

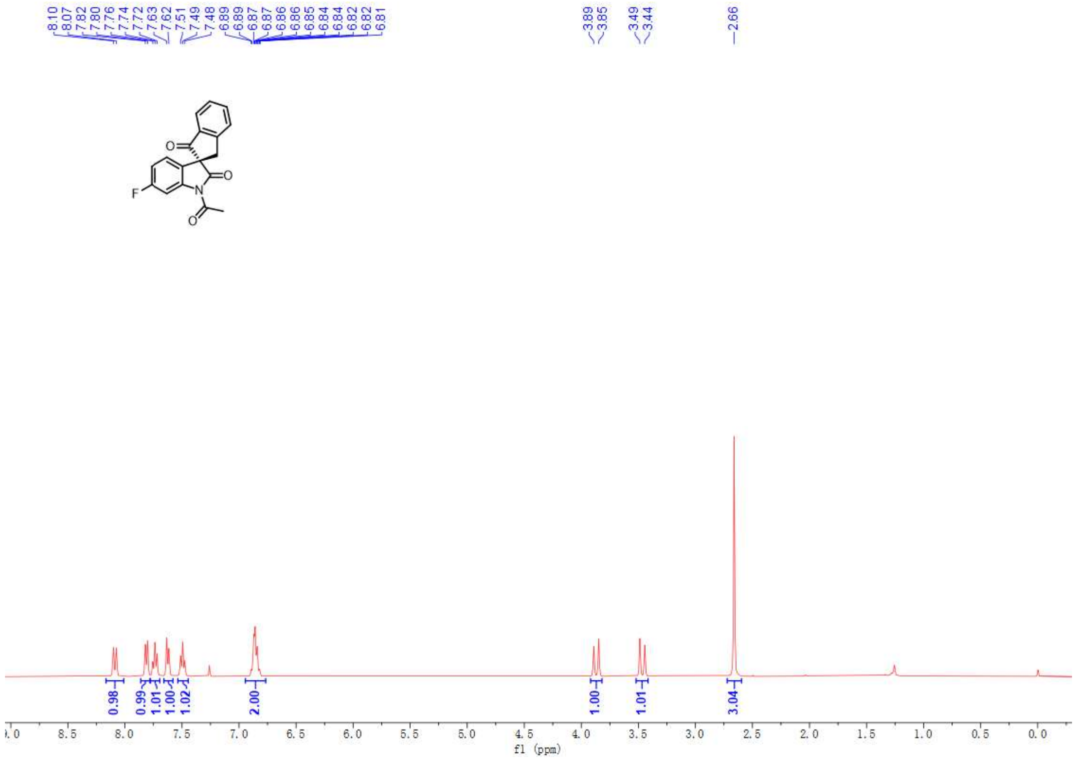

^{13}C {^{1}H} NMR of **2s**, 100 MHz, $CDCl_3$

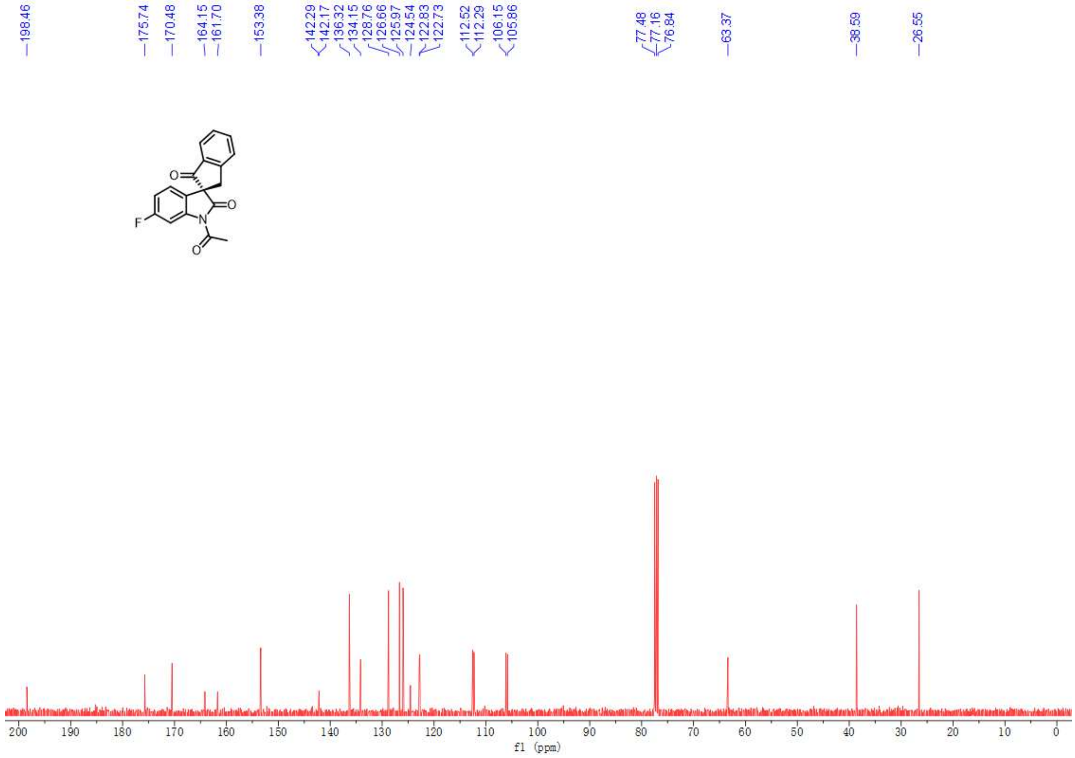

^{19}F NMR of **2s**, 376 MHz, $CDCl_3$

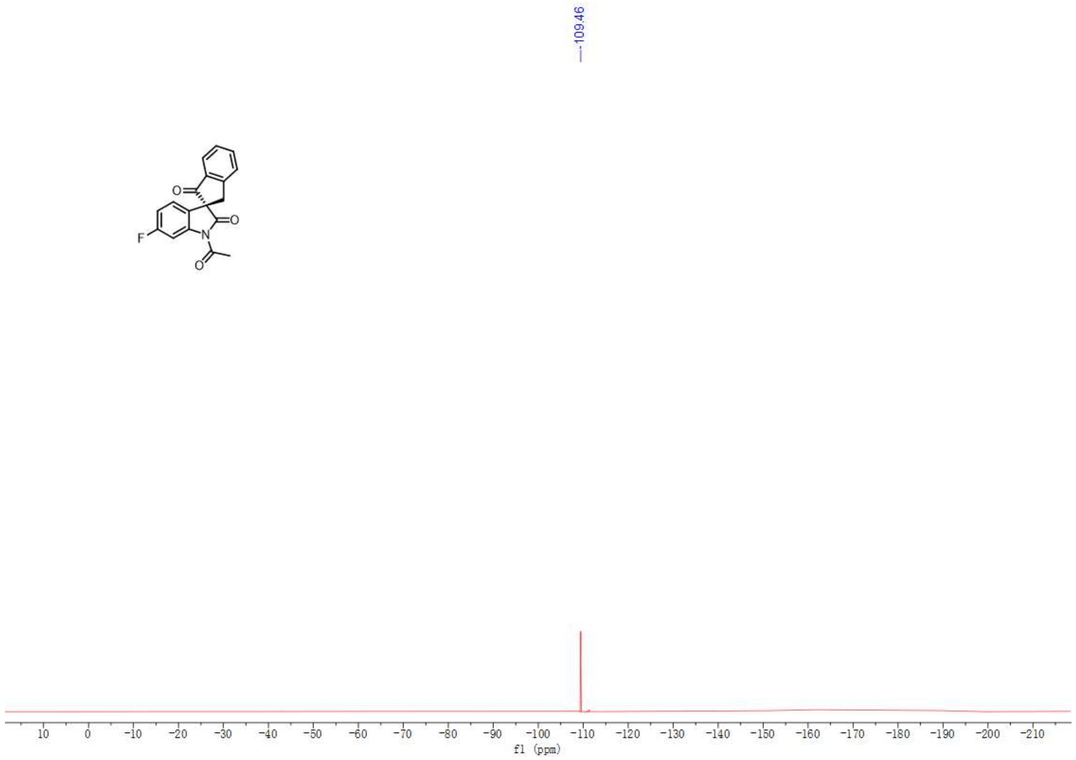

^{1}H NMR of **2t**, 400 MHz, $CDCl_3$

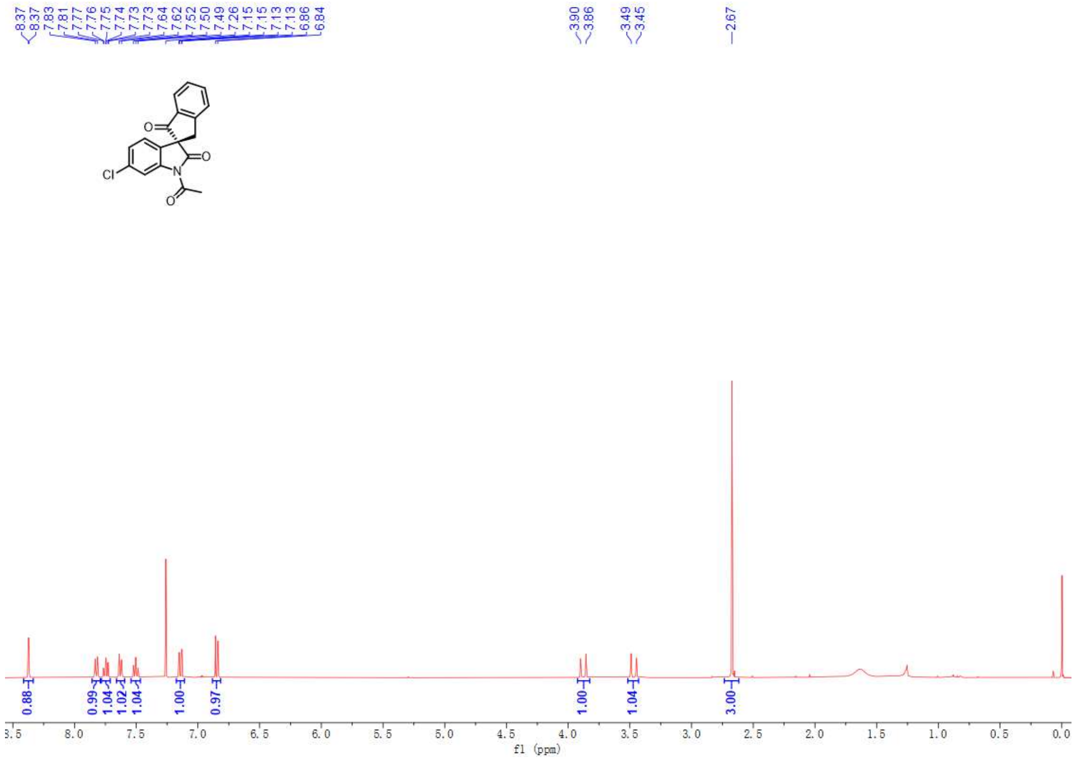

^{13}C {^{1}H} NMR of **2t**, 100 MHz, $CDCl_3$

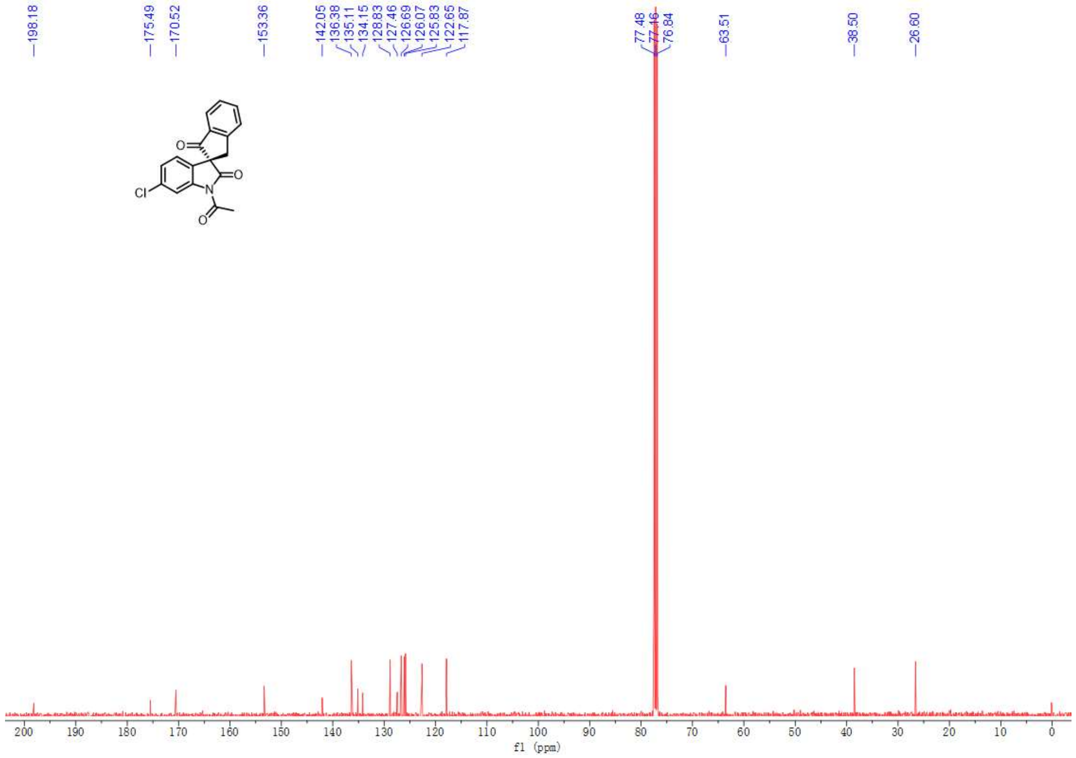

^{1}H NMR of **2u**, 400 MHz, $CDCl_3$

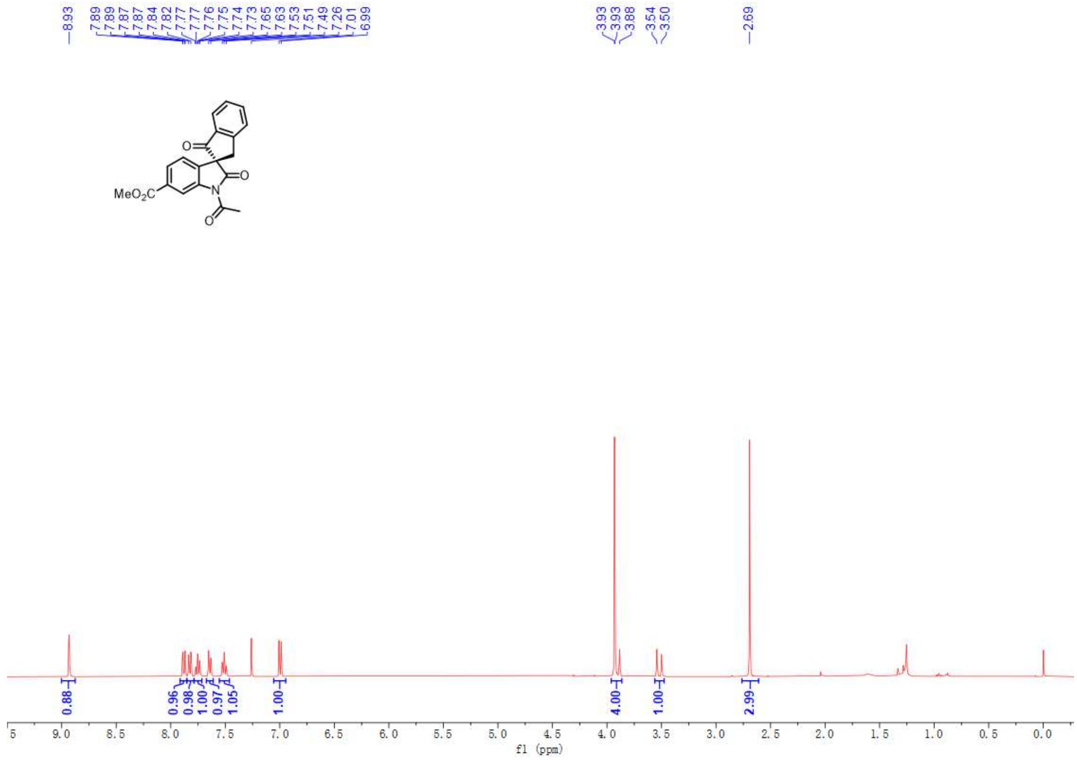

^{13}C {^{1}H} NMR of **2u**, 100 MHz, $CDCl_3$

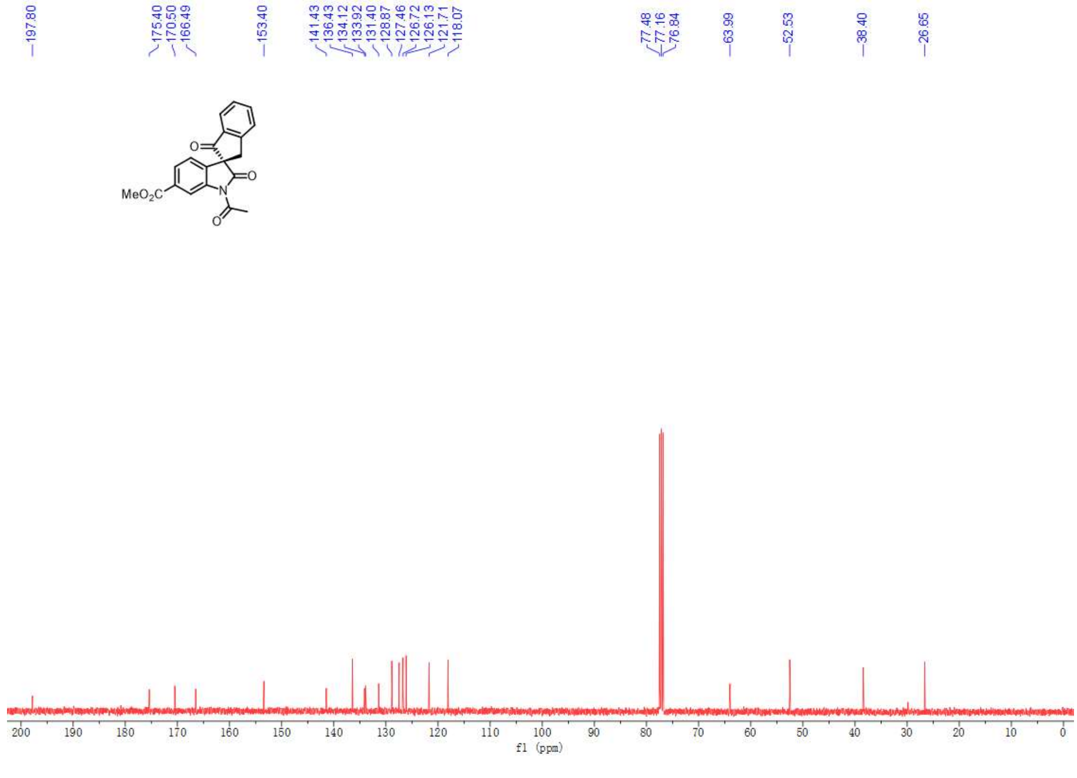

^{1}**H NMR** of **2v**, 400 MHz, $CDCl_3$

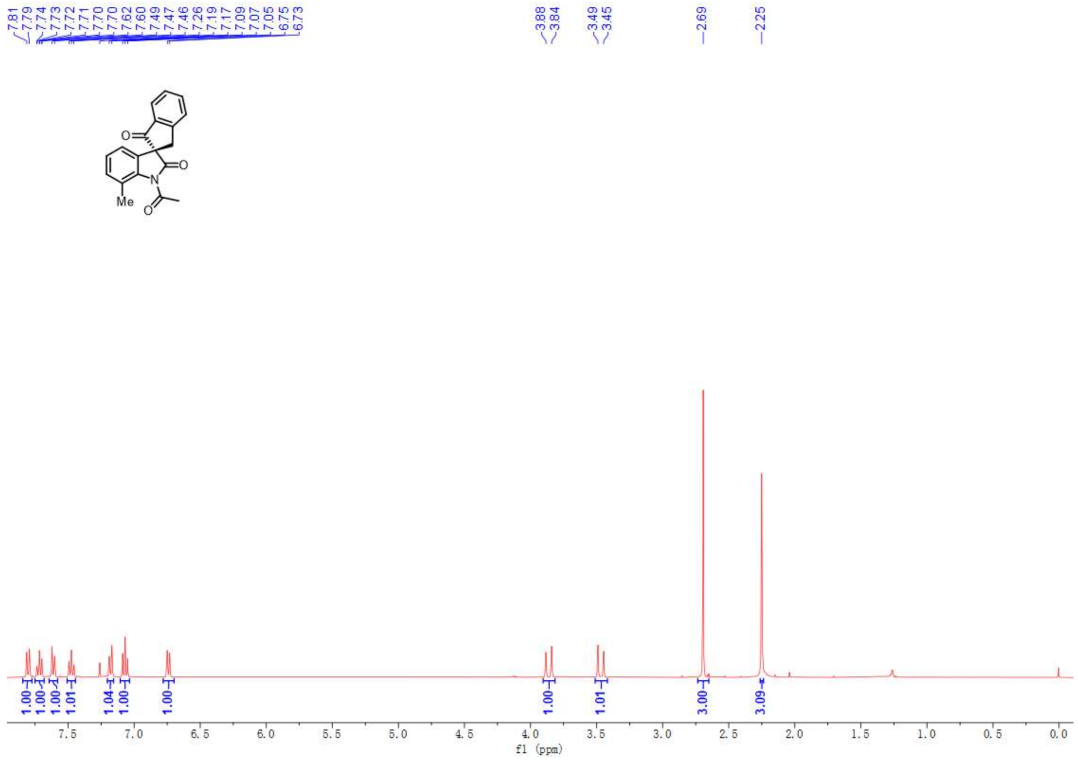

^{13}**C** {^{1}**H**} **NMR** of **2v**, 100 MHz, $CDCl_3$

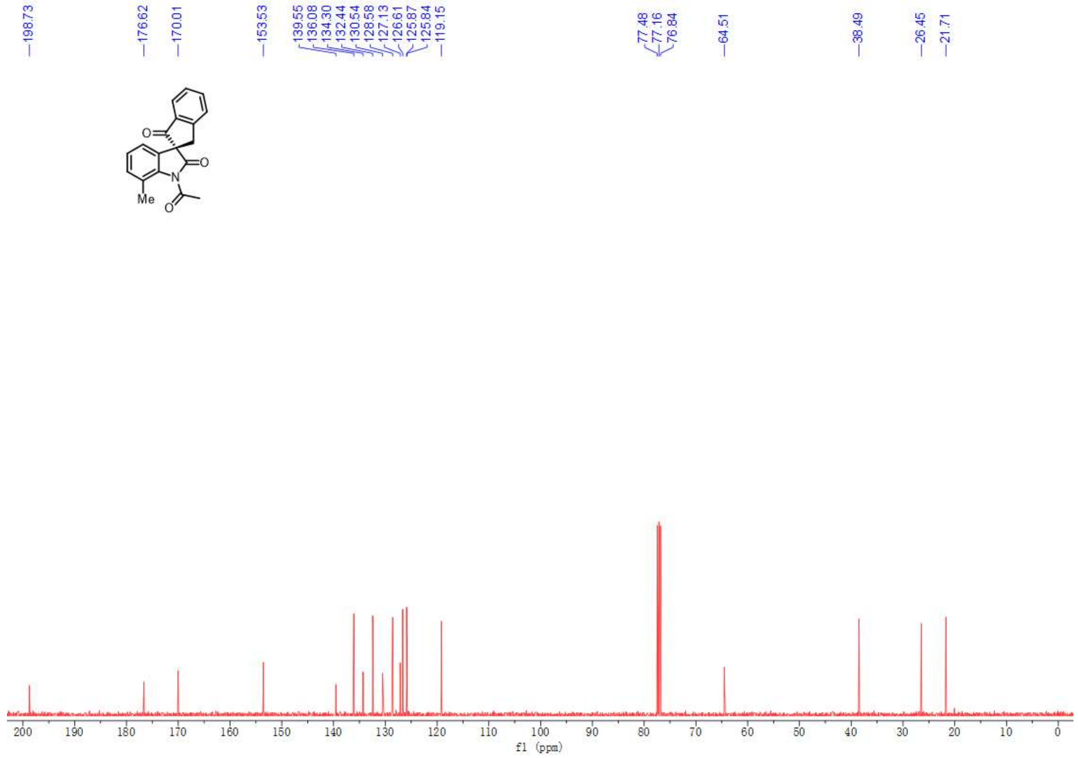

^{1}H NMR of **2w**, 400 MHz, DMSO-d_6

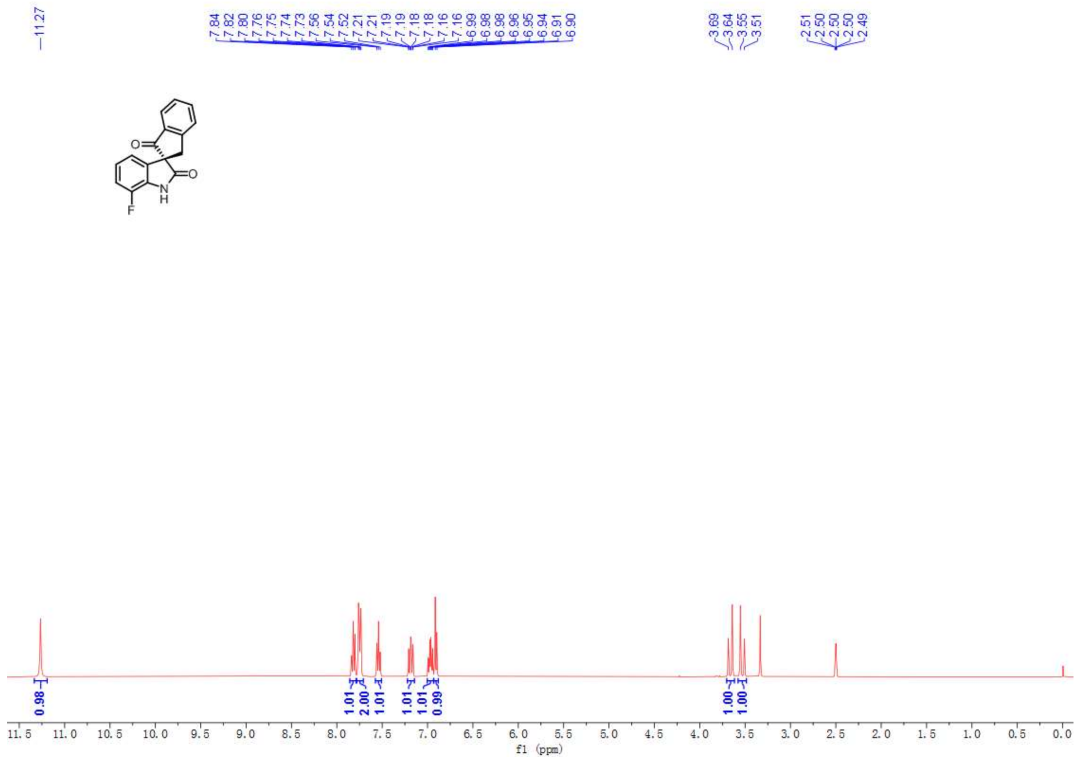

^{13}C {^{1}H} NMR of **2w**, 100 MHz, DMSO-d_6

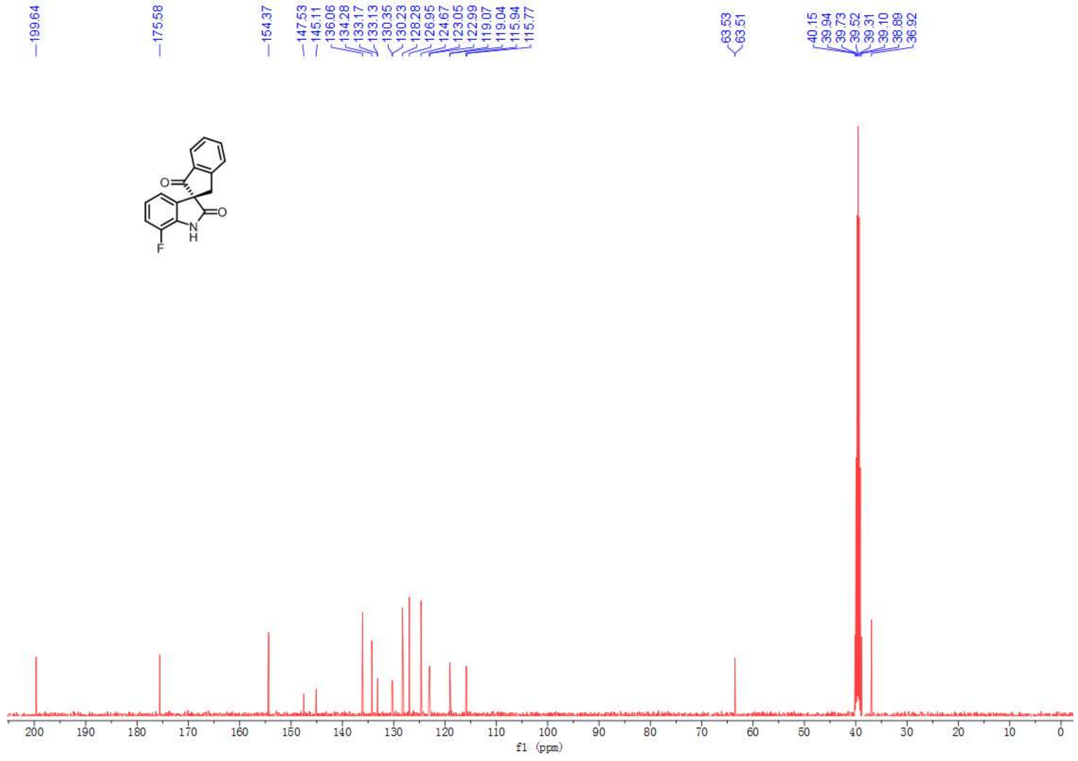

^{19}F NMR of **2w**, 376 MHz, DMSO-d_6

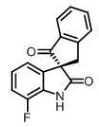

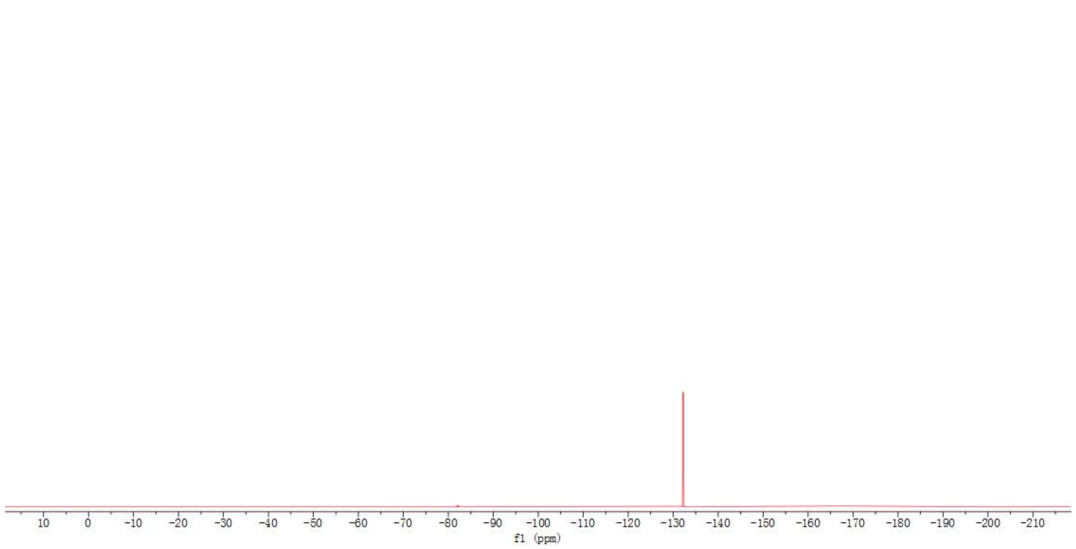

^{1}H NMR of **2x**, 400 MHz, $CDCl_3$

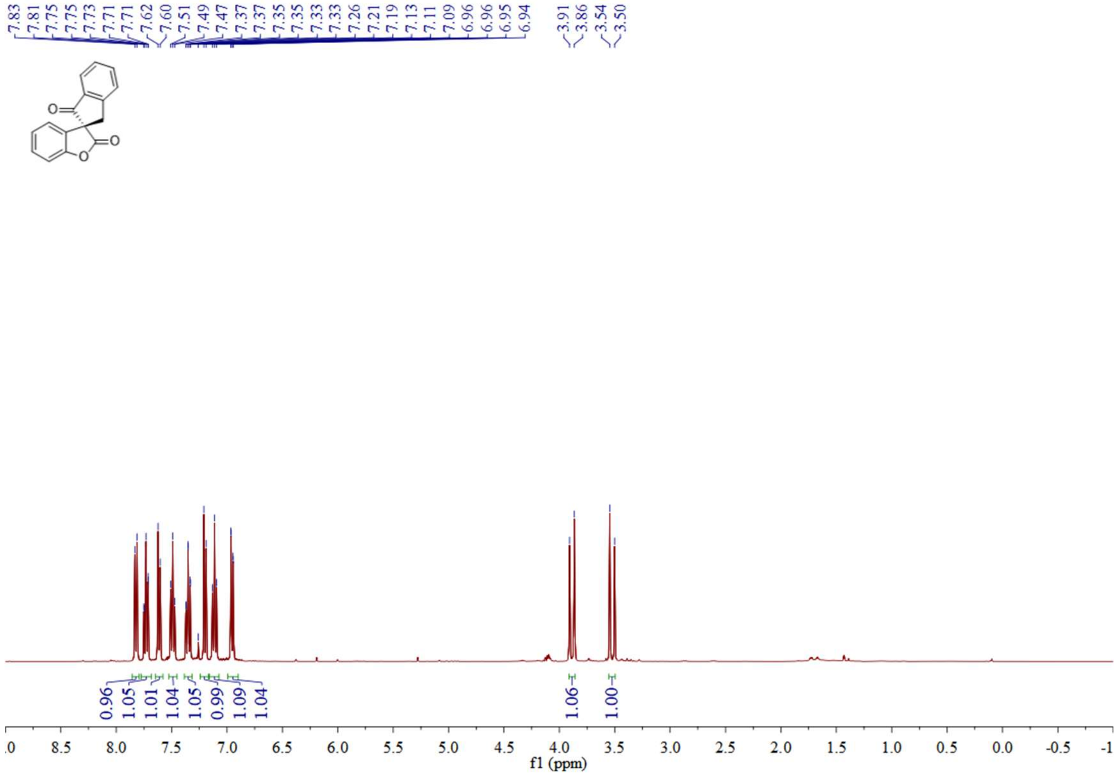

^{13}C {^{1}H} NMR of **2x**, 100 MHz, $CDCl_3$

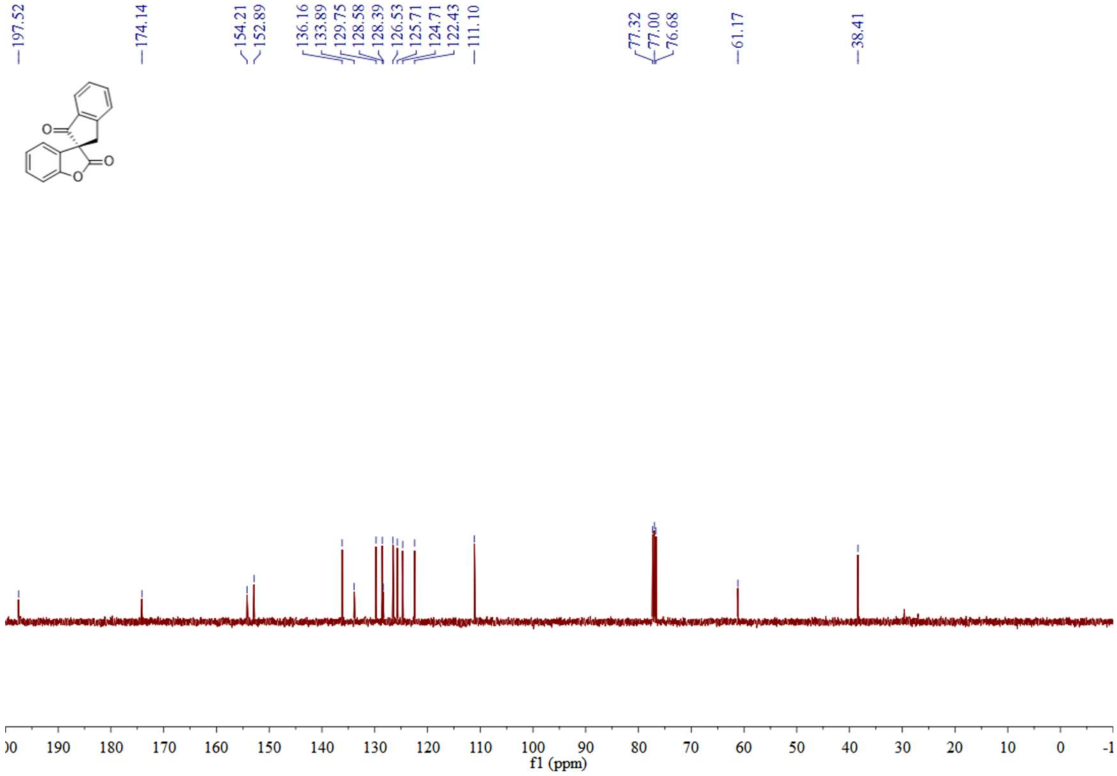

^{1}H NMR of **2y**, 400 MHz, $CDCl_3$

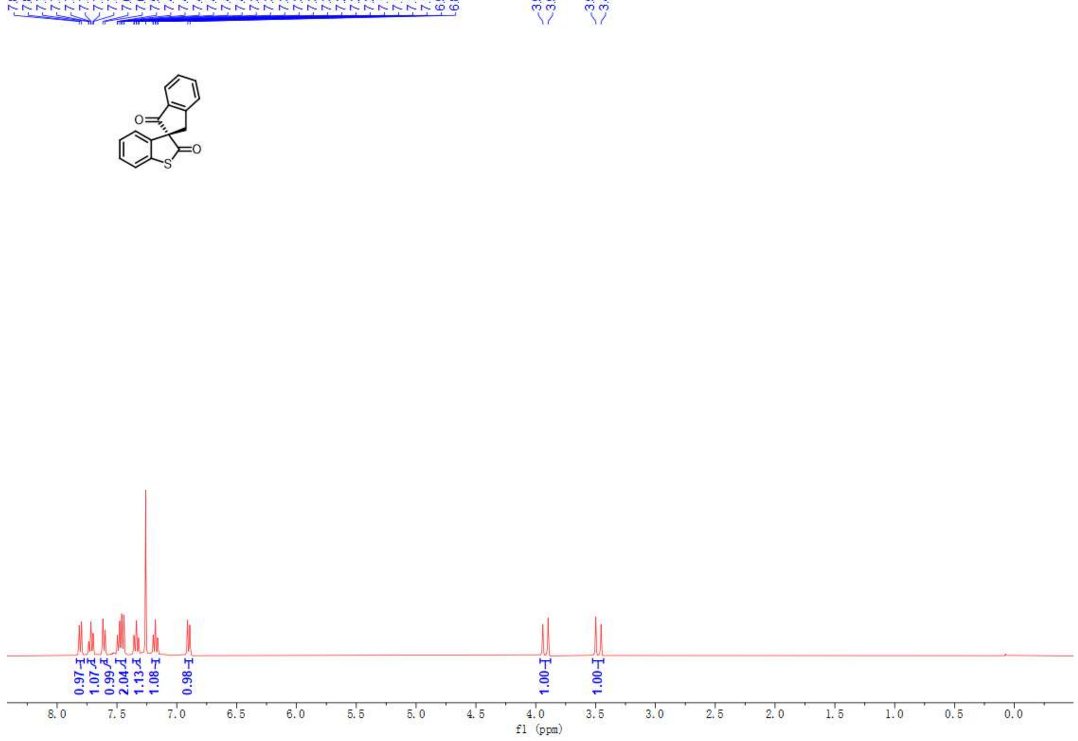

^{13}C {^{1}H} NMR of **2y**, 100 MHz, $CDCl_3$

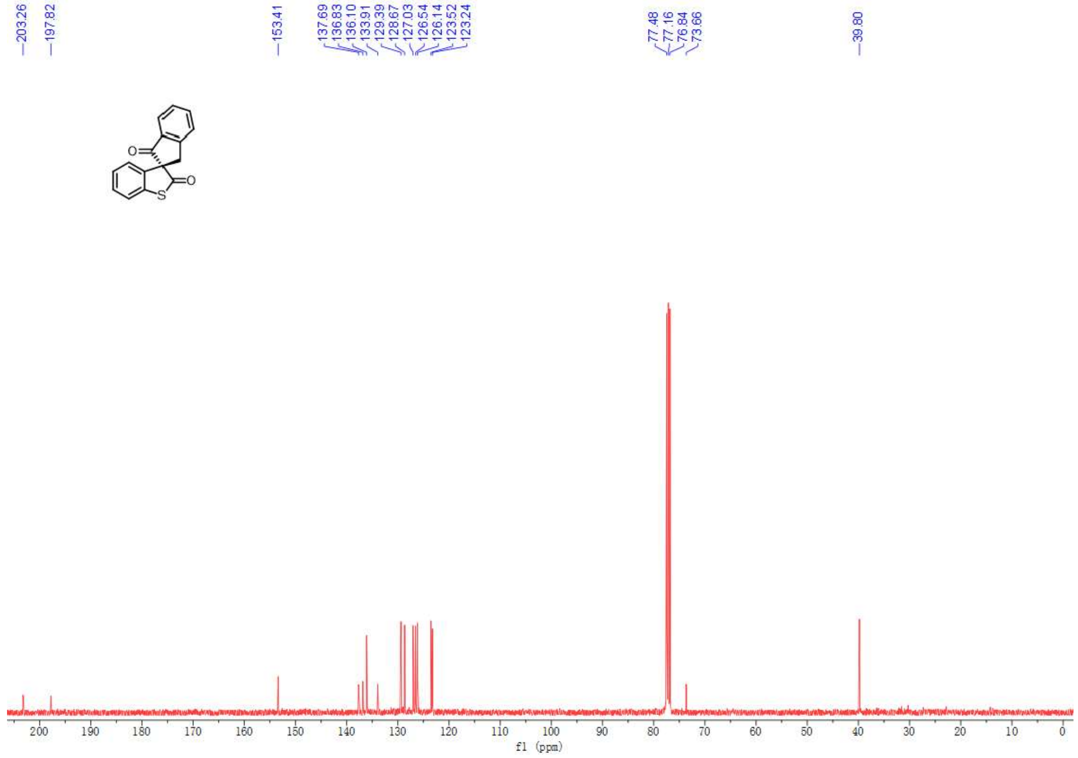

^{1}H NMR of 2z, 400 MHz, $CDCl_3$

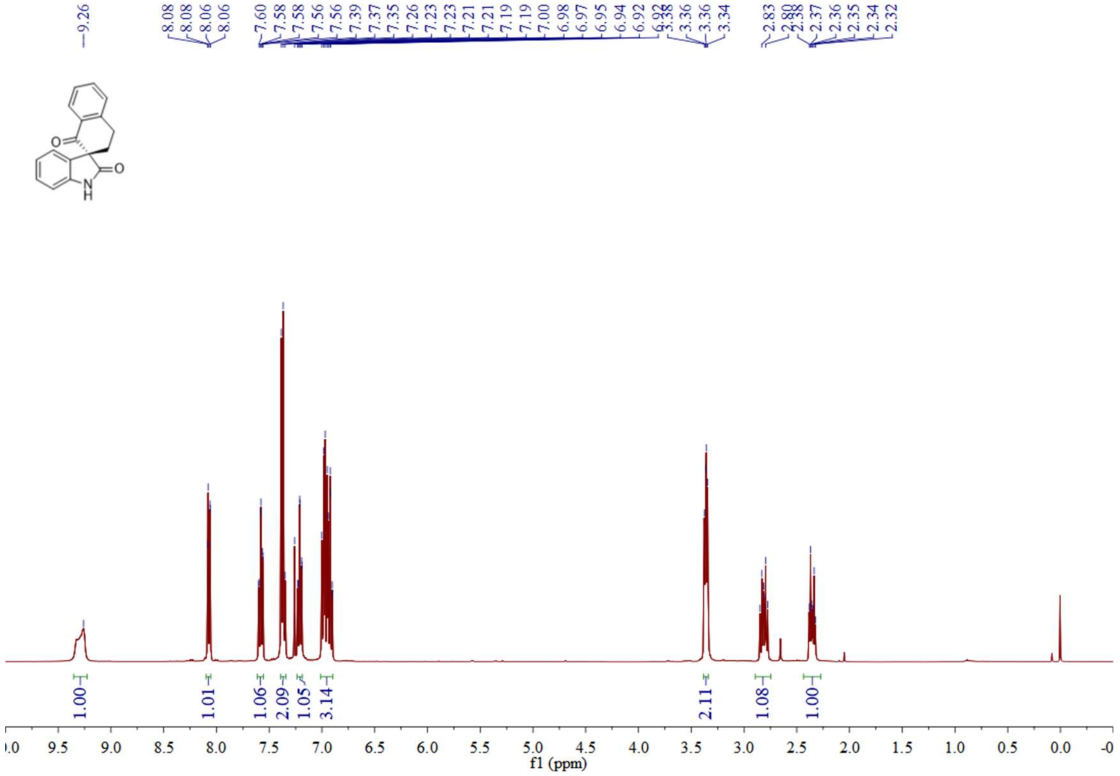

^{13}C {^{1}H} NMR of 2z, 100 MHz, $CDCl_3$

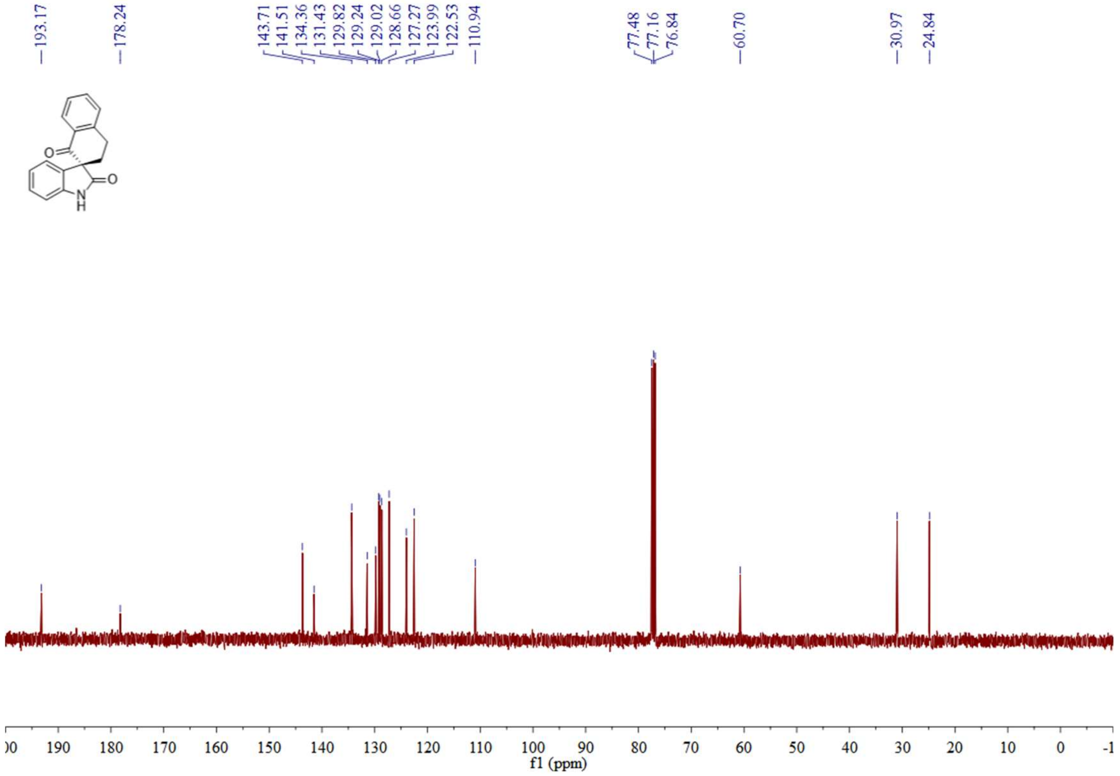

^{1}H **NMR** of **2aa**, 400 MHz, $CDCl_3$

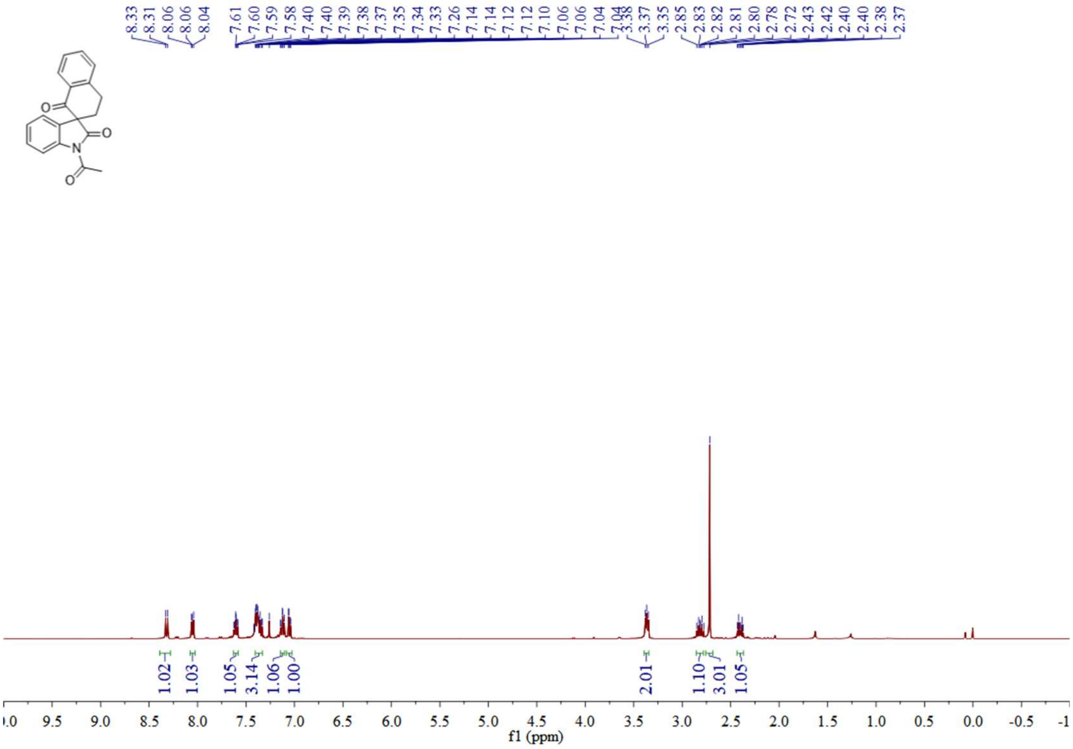

^{13}C {^{1}H} **NMR** of **2aa**, 100 MHz, $CDCl_3$

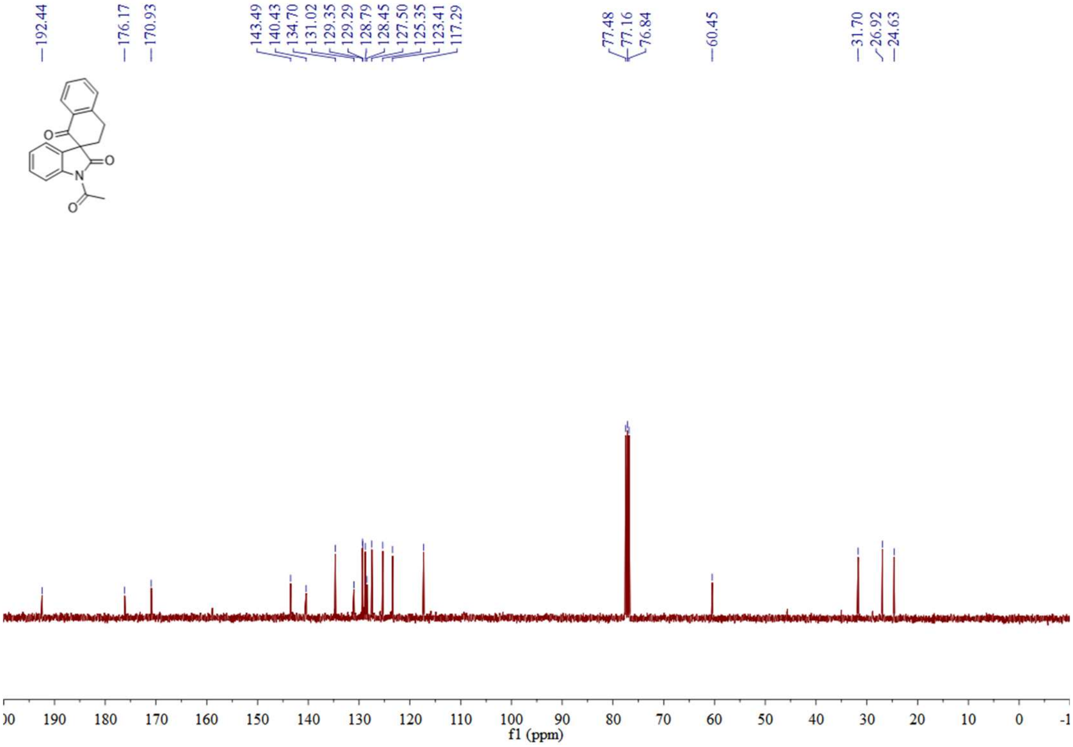

^{1}H NMR of **2ab**, 400 MHz, $CDCl_3$

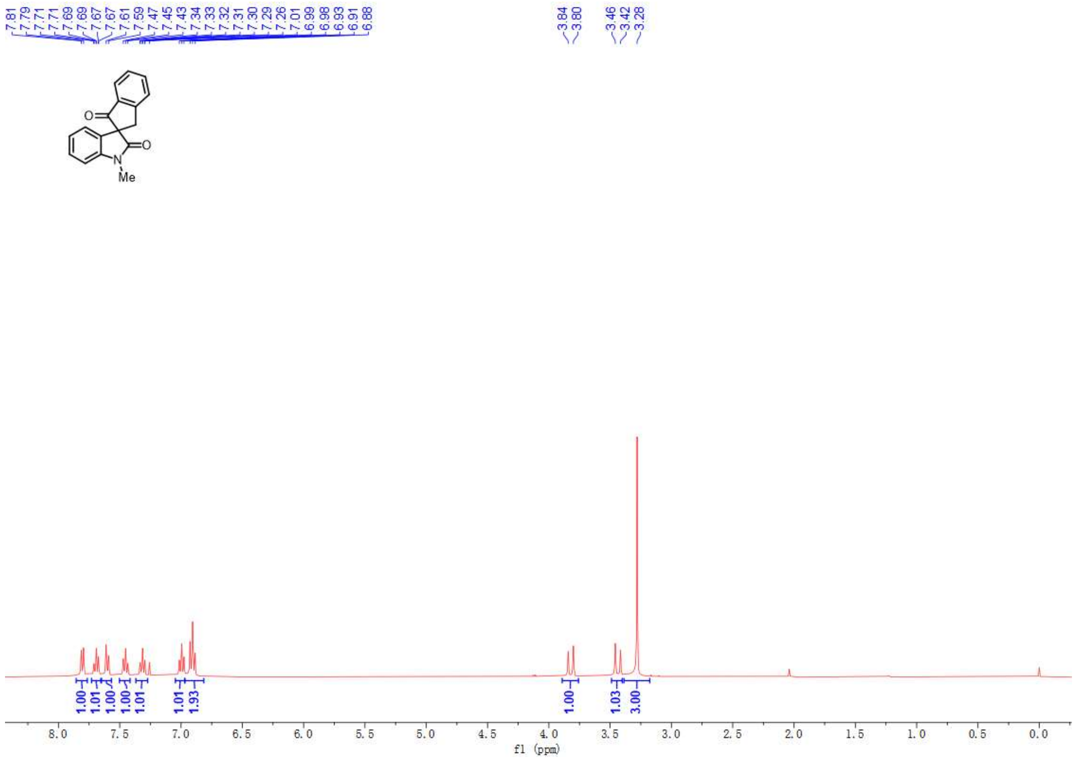

^{13}C {^{1}H} NMR of **2ab**, 100 MHz, $CDCl_3$

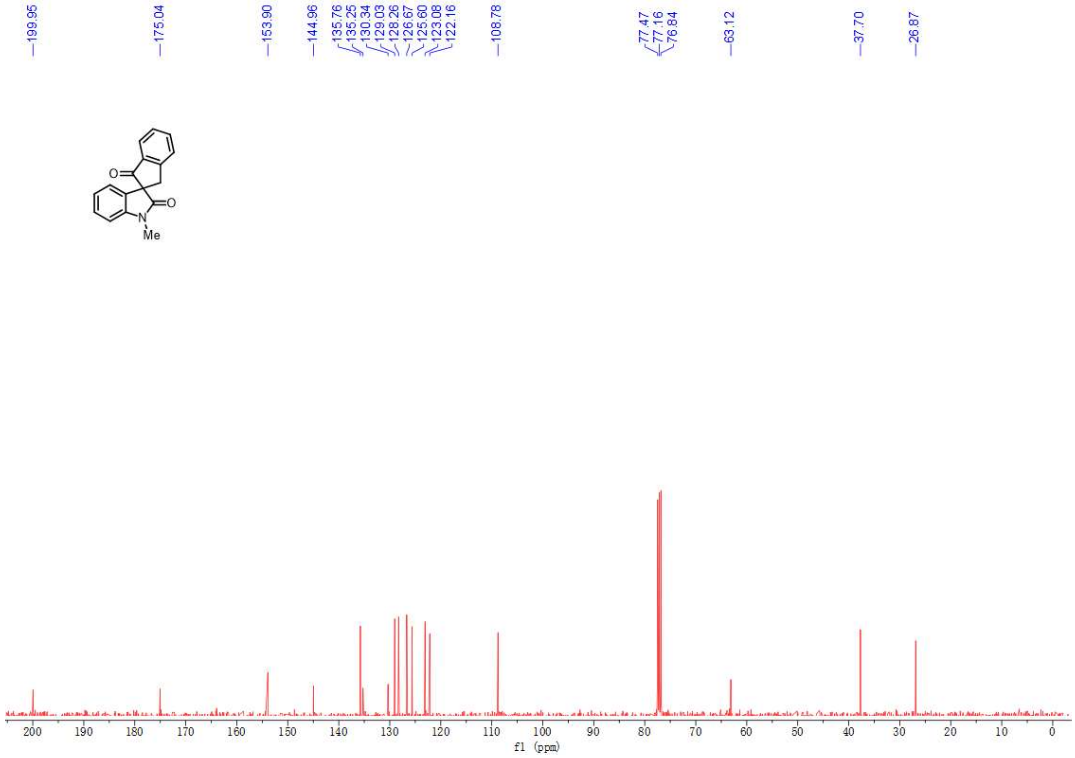

^{1}H NMR of **3a**, 400 MHz, $CDCl_3$

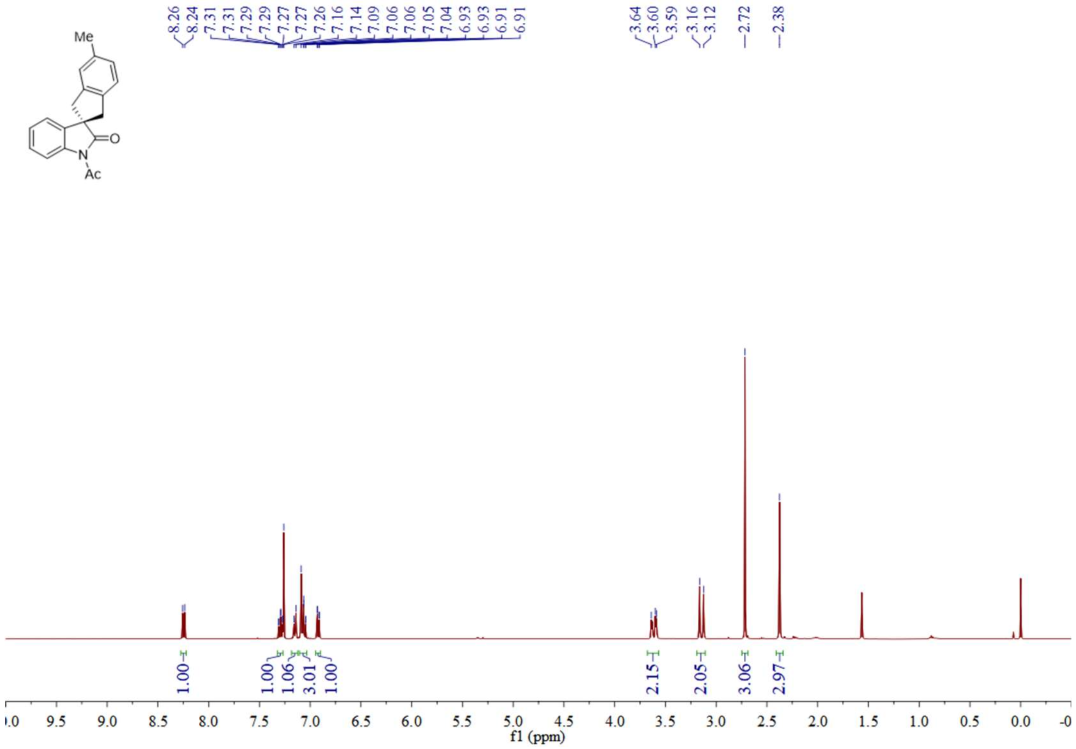

^{13}C {^{1}H} NMR of **3a**, 100 MHz, $CDCl_3$

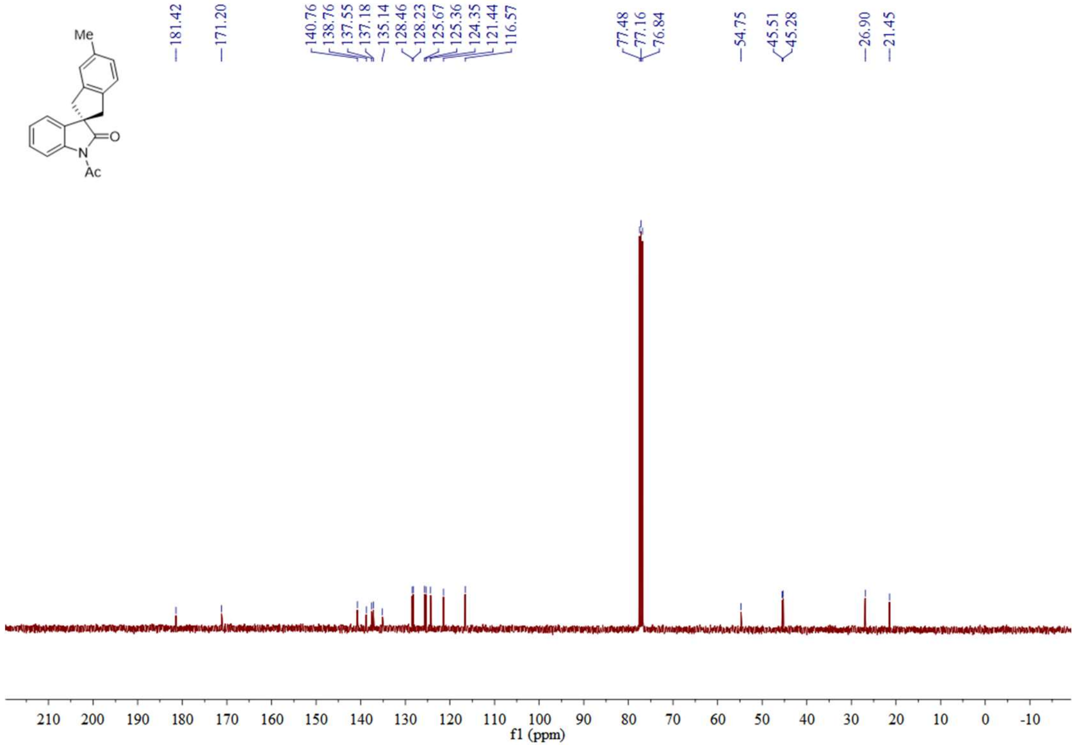

^{1}H NMR of **3b**, 400 MHz, $CDCl_3$

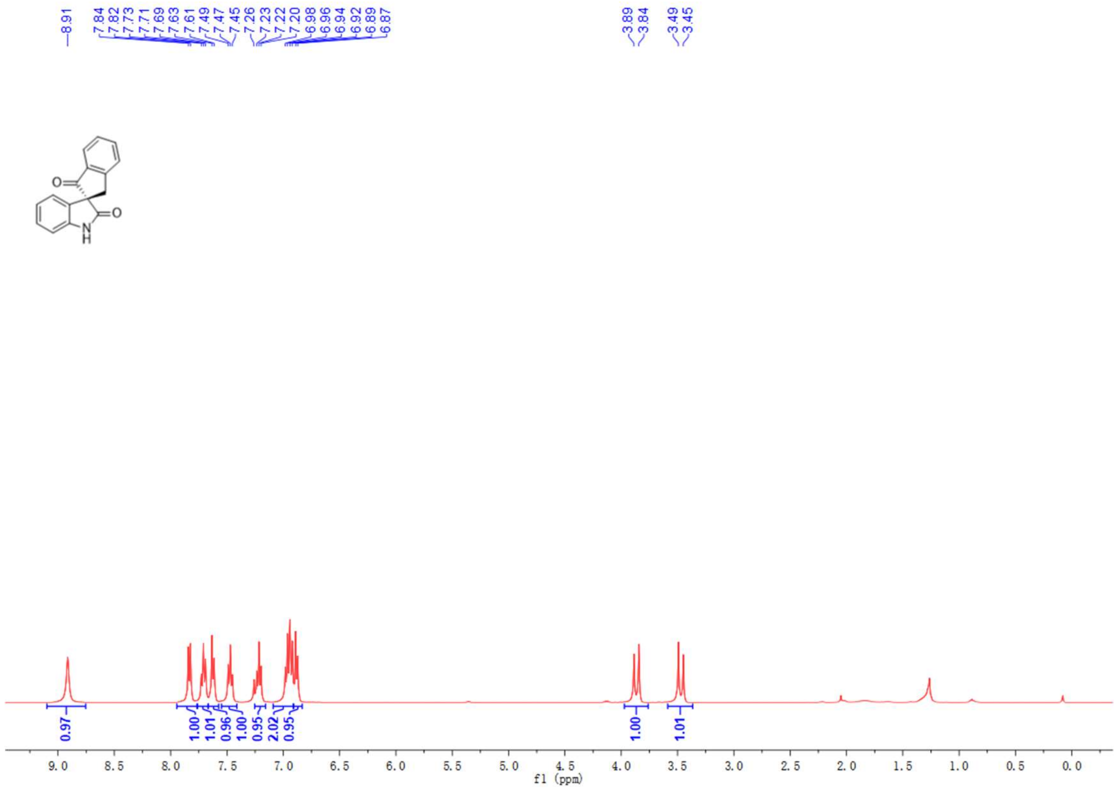

^{13}C {^{1}H} NMR of **3b**, 100 MHz, $CDCl_3$

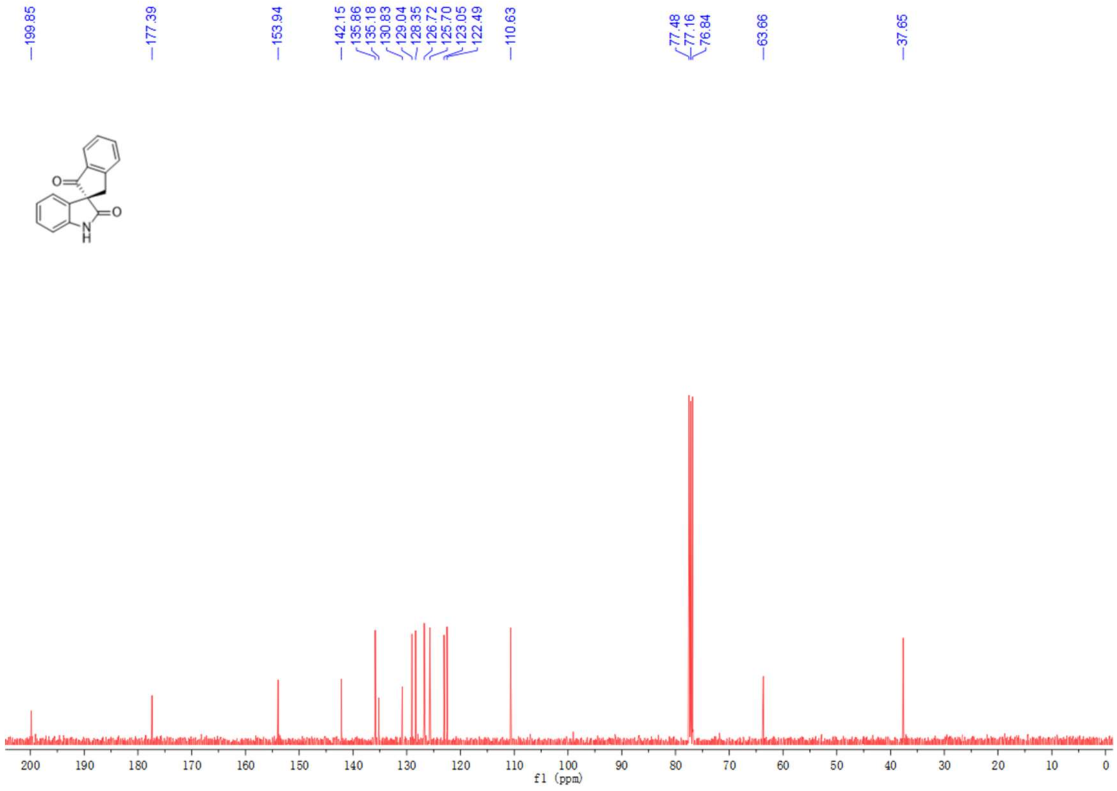

^{1}H NMR of **3c**, 400 MHz, $CDCl_3$

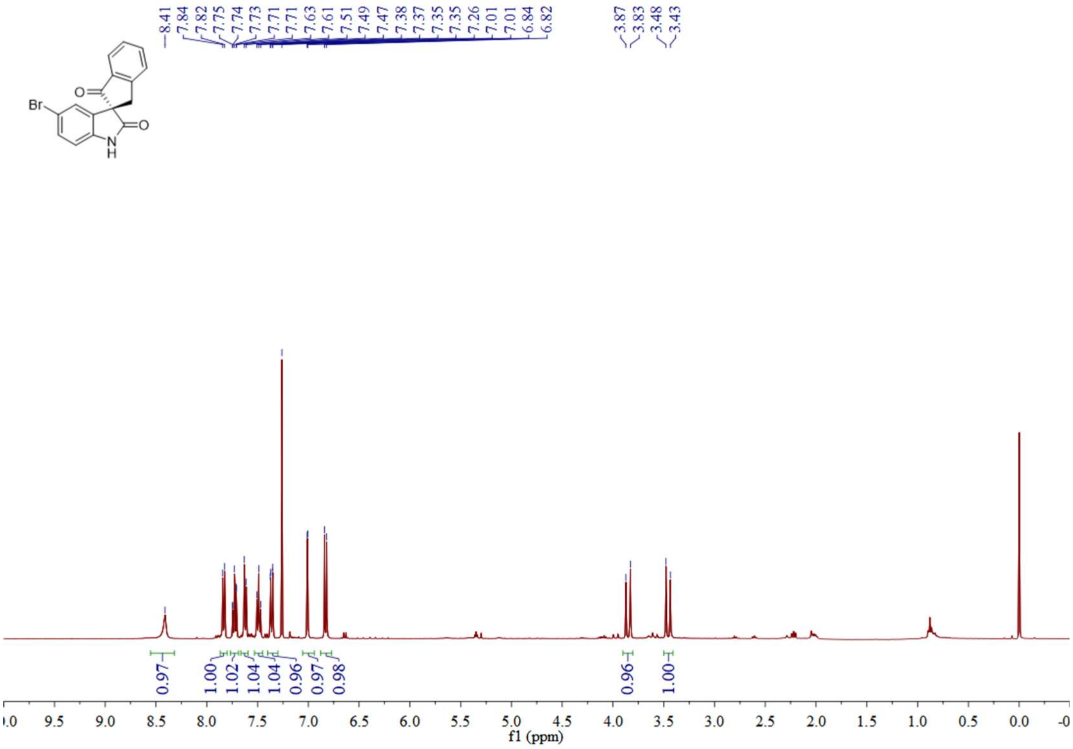

^{13}C {^{1}H} NMR of **3c**, 100 MHz, $CDCl_3$

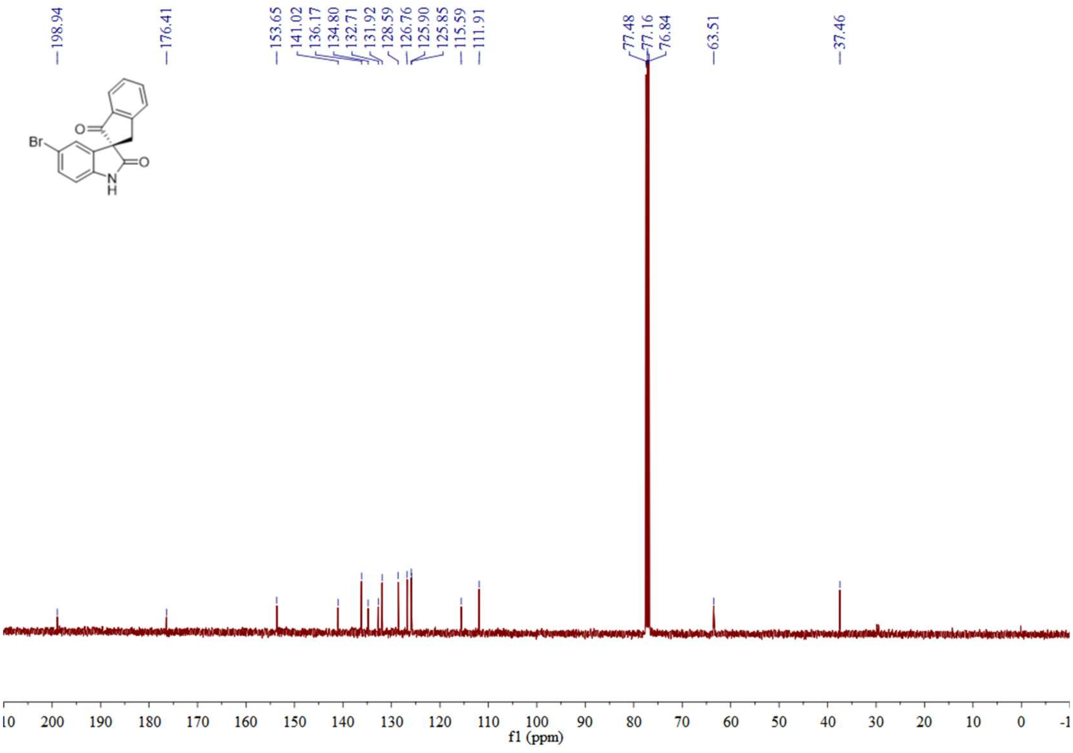

^{1}H NMR of **3d**, 400 MHz, $CDCl_3$

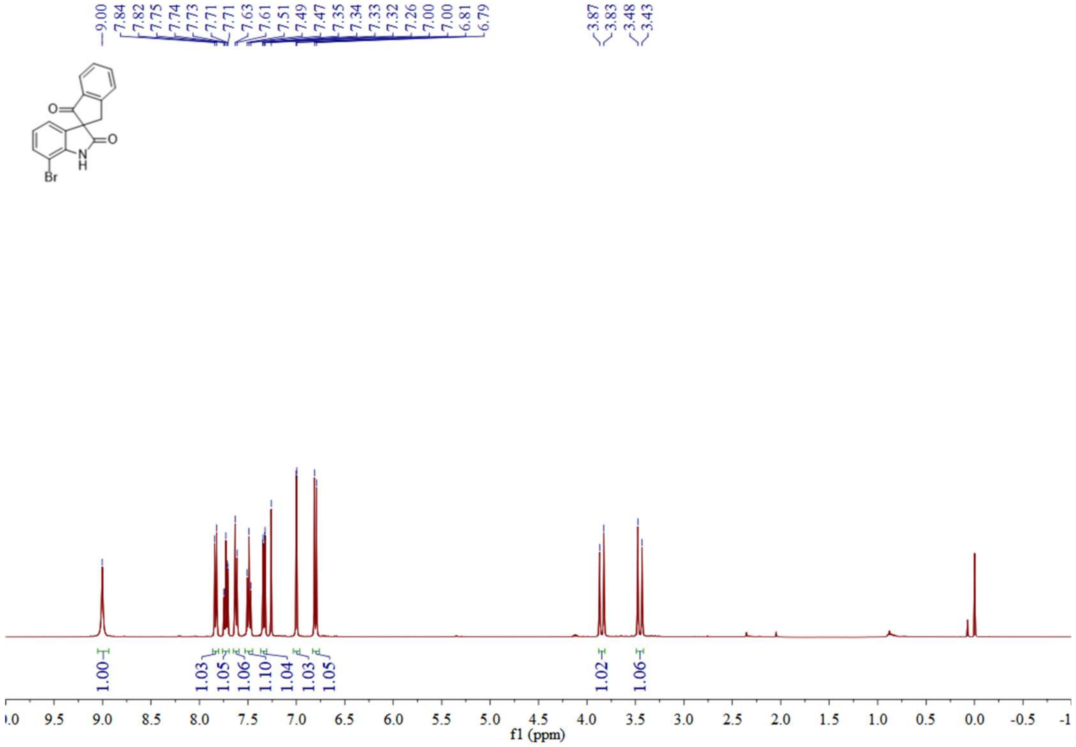

^{13}C {^{1}H} NMR of **3d**, 100 MHz, $CDCl_3$

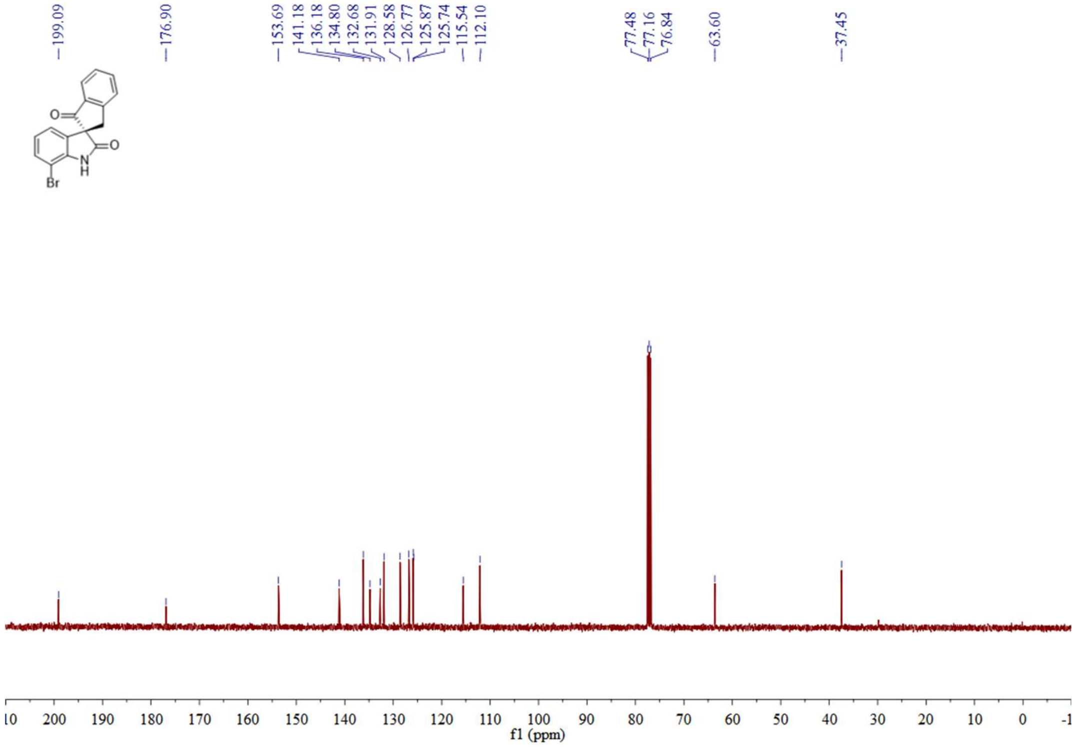

^{1}H NMR of **3e**, 400 MHz, DMSO-d_6

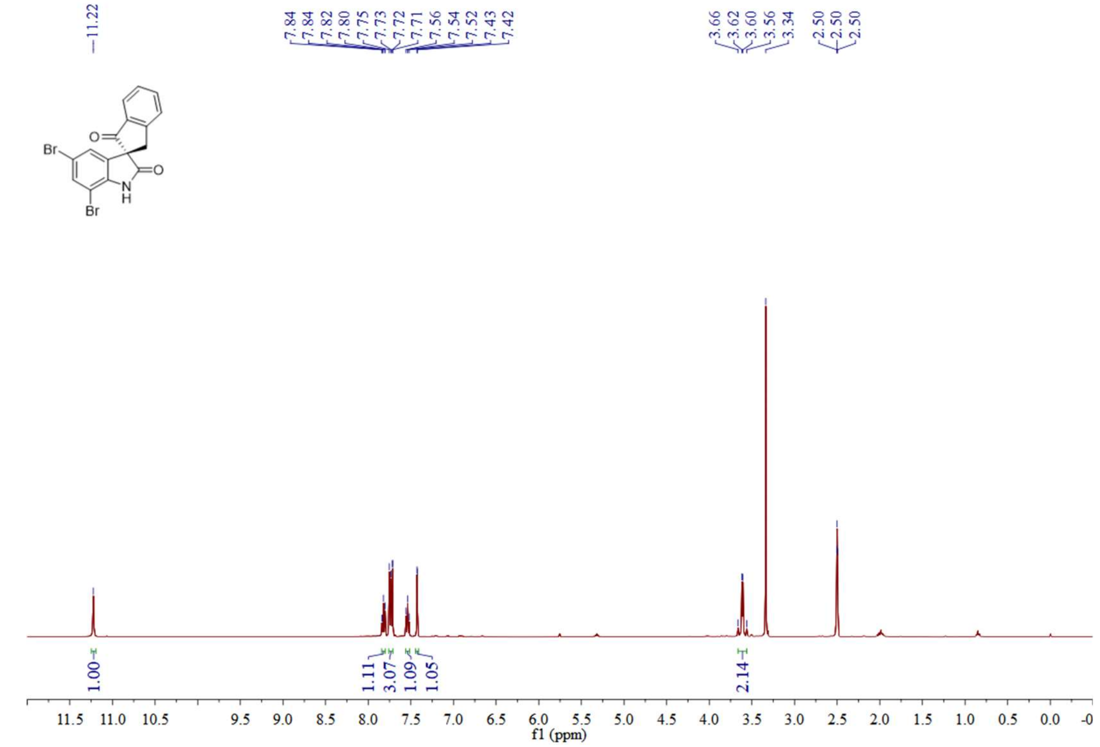

^{13}C {^{1}H} NMR of **3e**, 100 MHz, DMSO-d_6

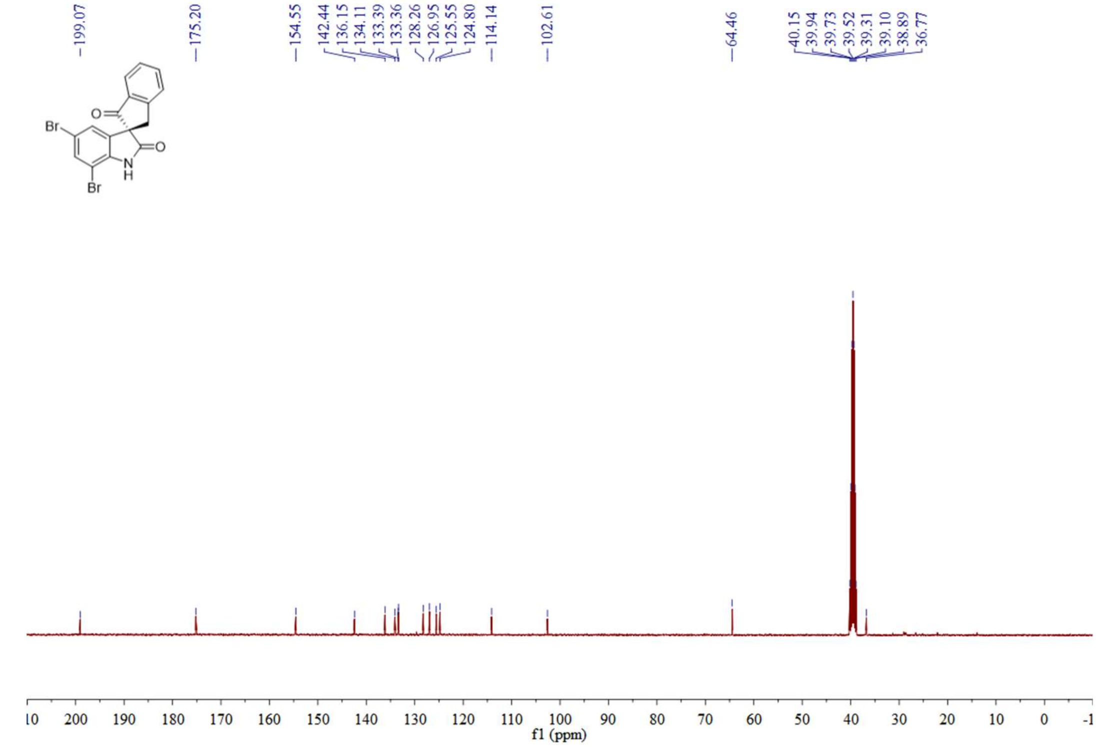

8. HPLC spectra

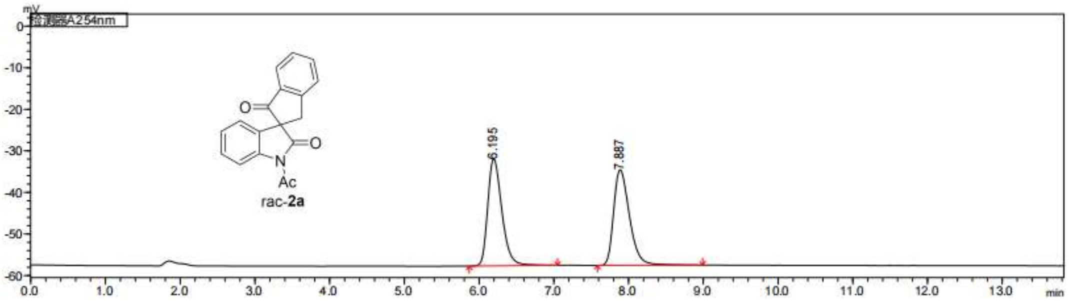

Peak	Time (min)	Area	Height	Concentration	Area (%)
峰号	保留时间	面积	高度	浓度	面积%
1	6.195	339132	25740	49.969	49.969
2	7.887	339548	23005	50.031	50.031

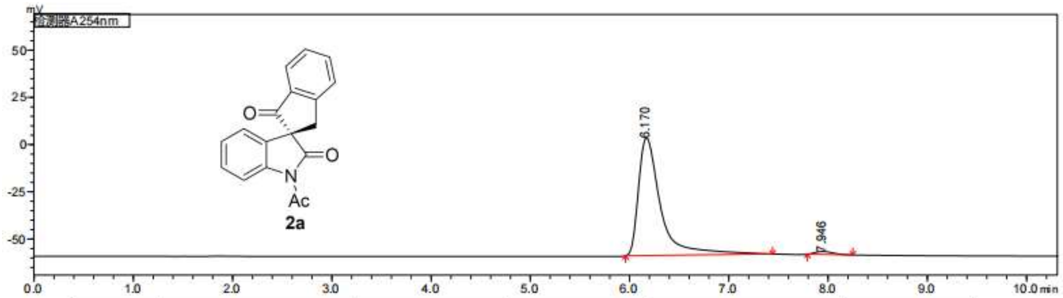

Peak	Time (min)	Area	Height	Concentration	Area (%)
峰号	保留时间	面积	高度	浓度	面积%
1	6.170	970206	62390	98.004	98.004
2	7.946	19762	1577	1.996	1.996

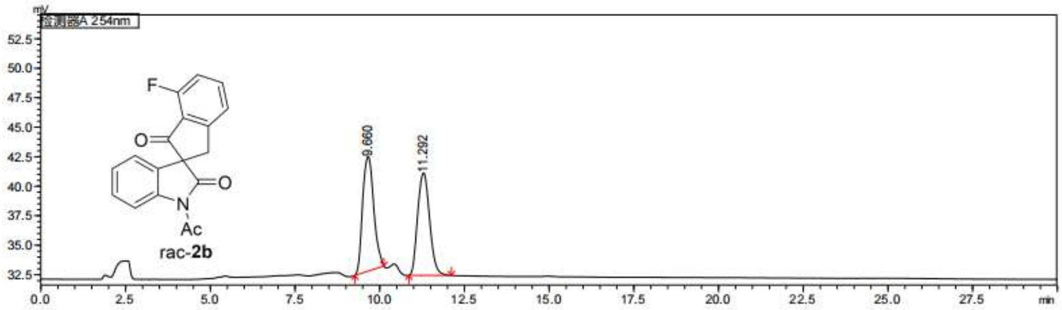

Peak	Time (min)	Area	Height	Concentration	Area (%)
峰号	保留时间	面积	高度	浓度	面积%
1	9.660	222321	9713	51.092	51.092
2	11.292	212815	8688	48.908	48.908

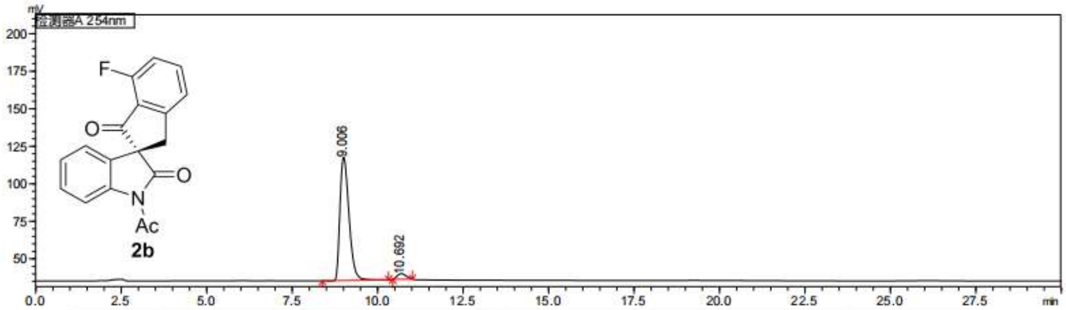

Peak	Time (min)	Area	Height	Concentration	Area (%)
峰号	保留时间	面积	高度	浓度	面积%
1	9.006	1515002	82096	95.737	95.737
2	10.692	67465	3729	4.263	4.263

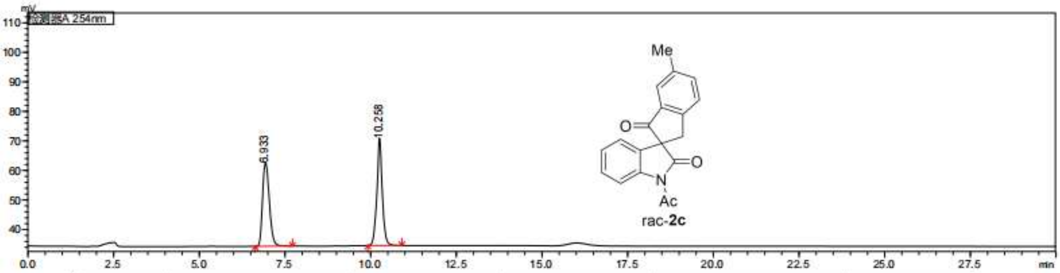

Peak	Time (min)	Area	Height	Concentration	Area (%)
峰号	保留时间	面积	高度	浓度	面积%
1	6.933	391301	28265	49.606	49.606
2	10.258	397517	36366	50.394	50.394

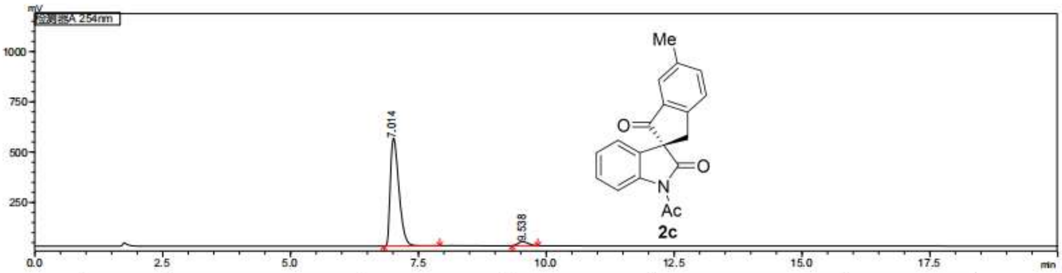

Peak	Time (min)	Area	Height	Concentration	Area (%)
峰号	保留时间	面积	高度	浓度	面积%
1	7.014	6307427	536896	95.817	95.817
2	9.538	275340	19666	4.183	4.183

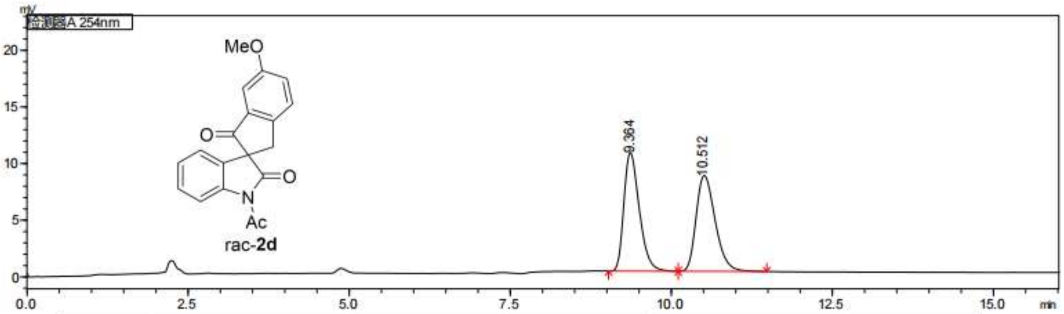

Peak	Time (min)	Area	Height	Concentration	Area (%)
峰号	保留时间	面积	高度	浓度	面积%
1	9.364	175017	10468	50.106	50.106
2	10.512	174279	8455	49.894	49.894

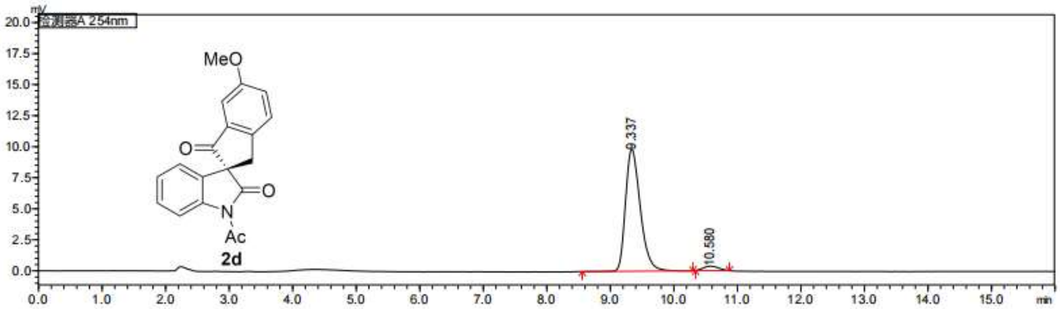

Peak	Time (min)	Area	Height	Concentration	Area (%)
峰号	保留时间	面积	高度	浓度	面积%
1	9.337	159184	9851	96.461	96.461
2	10.580	5840	347	3.539	3.539

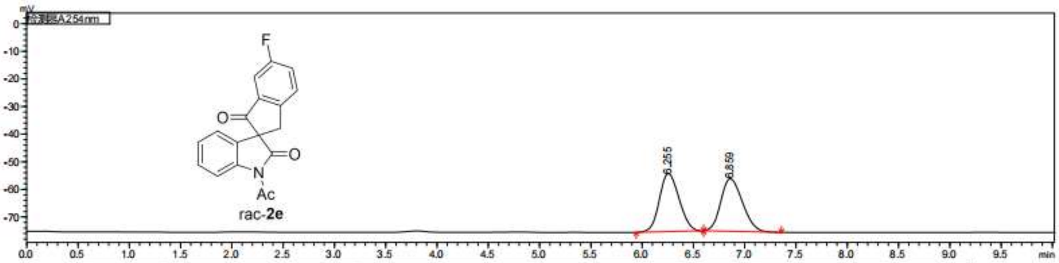

Peak	Time (min)	Area	Height	Concentration	Area (%)

峰号	保留时间	面积	高度	浓度	面积%
1	6.255	281722	21002	50.164	50.164
2	6.859	279878	19023	49.836	49.836

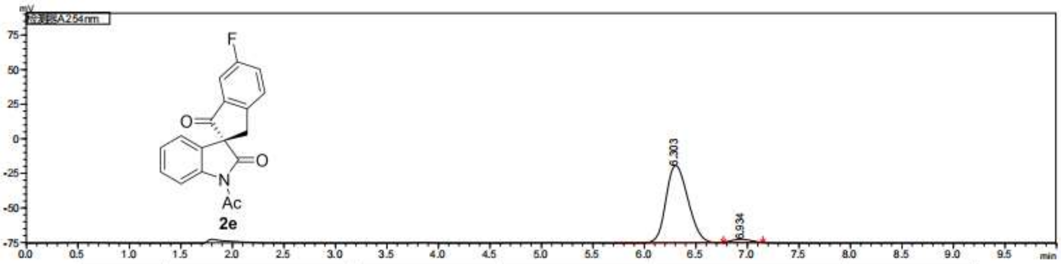

Peak	Time (min)	Area	Height	Concentration	Area (%)

峰号	保留时间	面积	高度	浓度	面积%
1	6.303	823627	55869	96.719	96.719
2	6.934	27937	2221	3.281	3.281

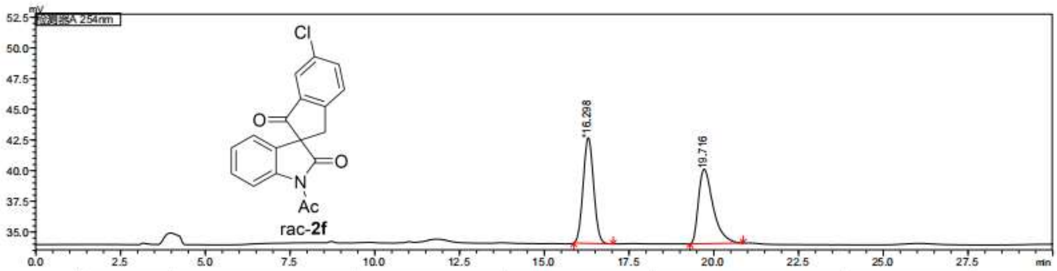

Peak	Time (min)	Area	Height	Concentration	Area (%)
峰号	保留时间	面积	高度	浓度	面积%
1	16.298	185071	8609	0.000	50.277
2	19.716	183033	6075	0.000	49.723

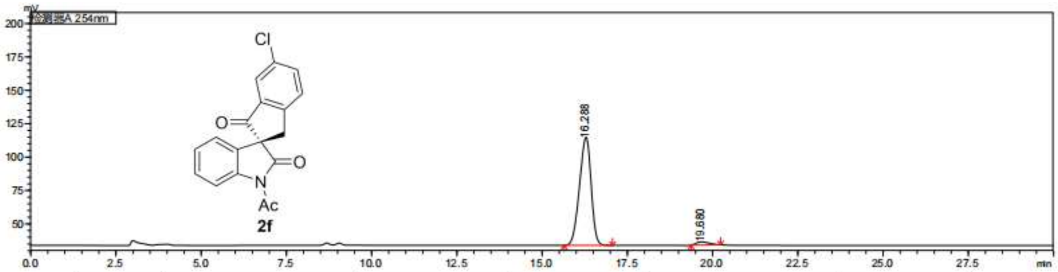

Peak	Time (min)	Area	Height	Concentration	Area (%)
峰号	保留时间	面积	高度	浓度	面积%
1	16.288	1920192	80955	96.821	96.821
2	19.680	63052	2372	3.179	3.179

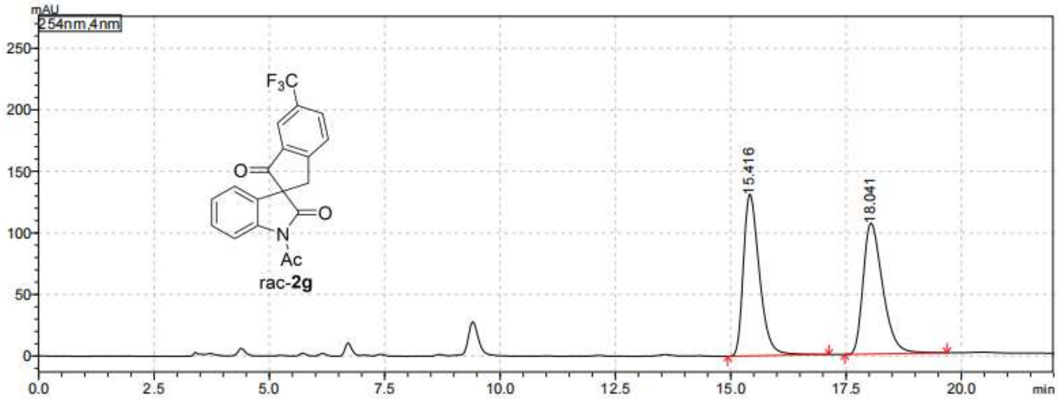

Peak	Time (min)	Area (%)	Area	Height
峰号	保留时间	面积%	面积	高度
1	15.416	50.331	3179507	131225
2	18.041	49.669	3137744	106287

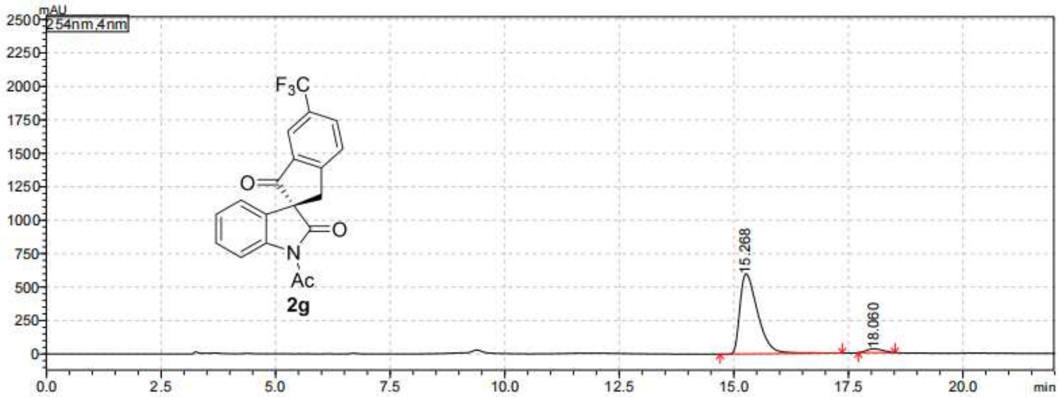

Peak	Time (min)	Area (%)	Area	Height
峰号	保留时间	面积%	面积	高度
1	15.268	95.835	16190927	599471
2	18.060	4.165	703679	29041

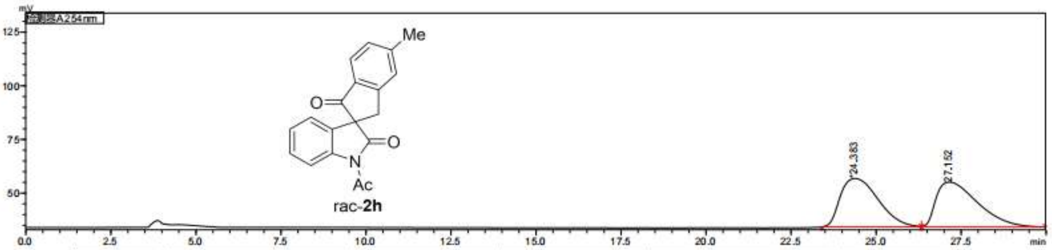

Peak	Time (min)	Area	Height	Concentration	Area (%)
峰号	保留时间	面积	高度	浓度	面积%
1	24.383	1754725	22546	49.582	49.582
2	27.152	1784342	20898	50.418	50.418

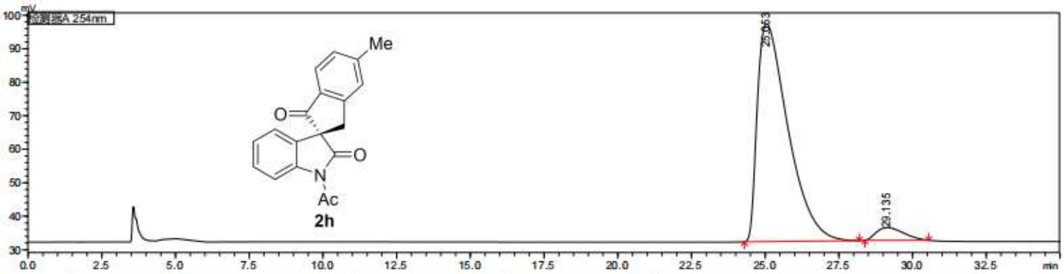

Peak	Time (min)	Area	Height	Concentration	Area (%)
峰号	保留时间	面积	高度	浓度	面积%
1	25.053	4708259	64988	95.889	95.889
2	29.135	201873	3430	4.111	4.111

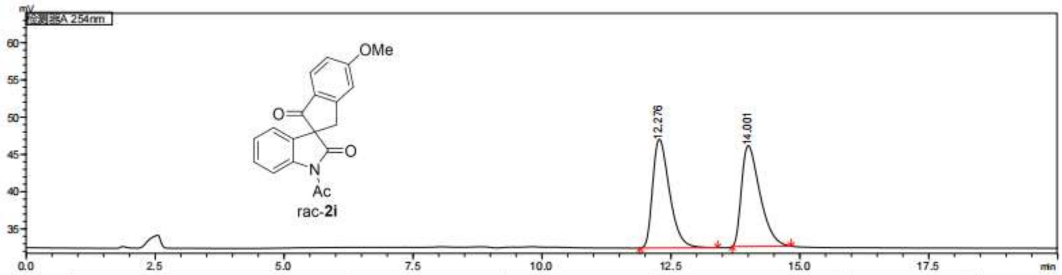

Peak	Time (min)	Area	Height	Concentration	Area (%)
峰号	保留时间	面积	高度	浓度	面积%
1	12.276	331802	14599	49.618	49.618
2	14.001	336912	13537	50.382	50.382

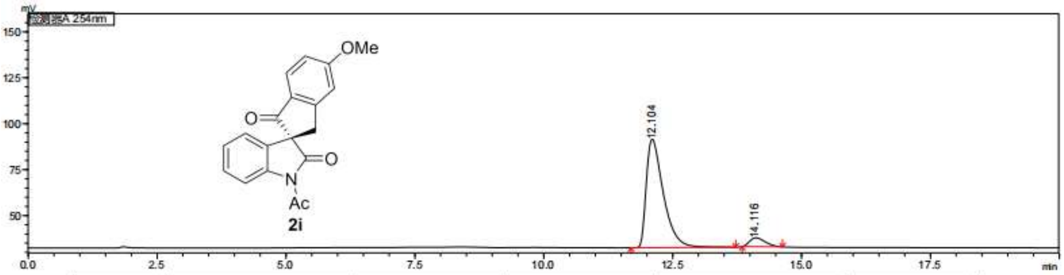

Peak	Time (min)	Area	Height	Concentration	Area (%)
峰号	保留时间	面积	高度	浓度	面积%
1	12.104	1359443	59060	93.034	93.034
2	14.116	101785	4822	6.966	6.966

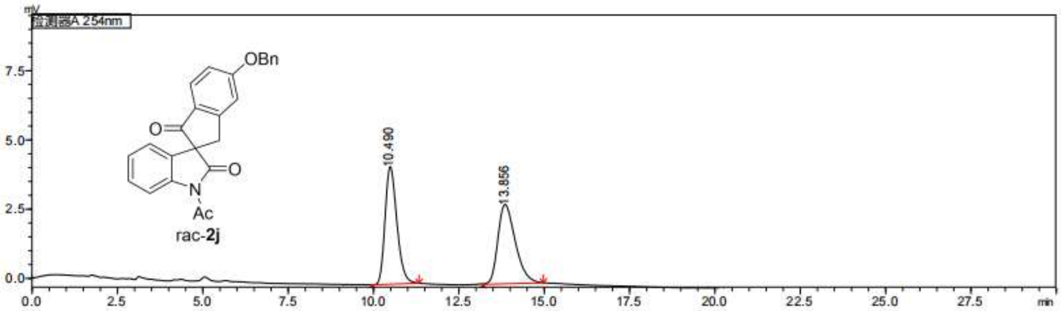

Peak	Time (min)	Area	Height	Concentration	Area (%)
峰号	保留时间	面积	高度	浓度	面积%
1	10.490	102583	4252	50.092	50.092
2	13.856	102204	2869	49.908	49.908

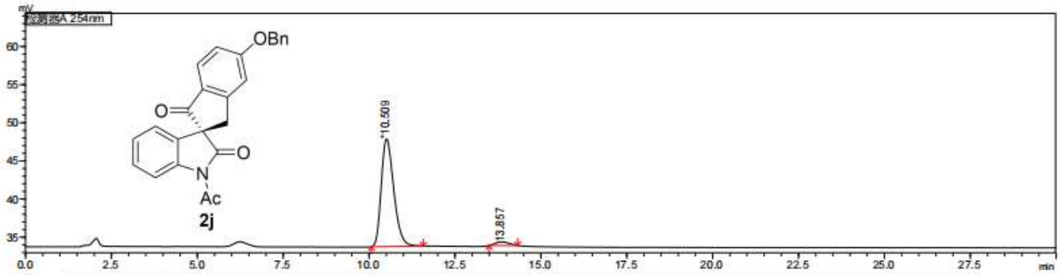

Peak	Time (min)	Area	Height	Concentration	Area (%)
峰号	保留时间	面积	高度	浓度	面积%
1	10.509	356832	14121	96.436	96.436
2	13.857	13187	471	3.564	3.564

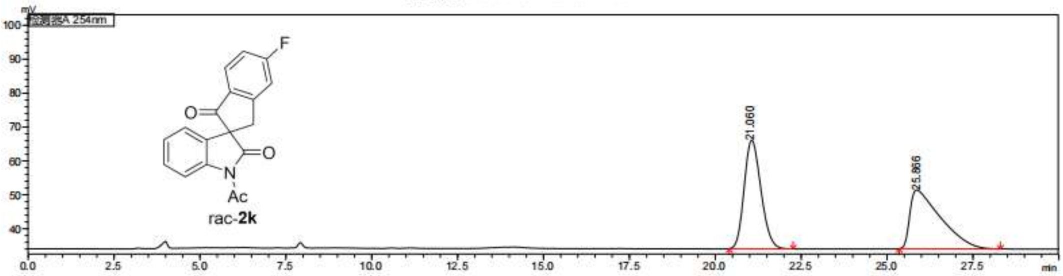

Peak	Time (min)	Area	Height	Concentration	Area (%)
峰号	保留时间	面积	高度	浓度	面积%
1	21.060	1088740	32019	50.301	50.301
2	25.866	1075691	17340	49.699	49.699

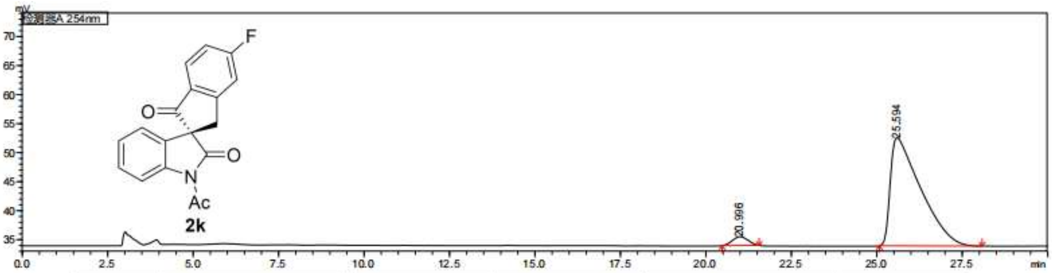

Peak	Time (min)	Area	Height	Concentration	Area (%)
峰号	保留时间	面积	高度	浓度	面积%
1	20.996	47715	1510	3.985	3.985
2	25.594	1149806	18607	96.015	96.015

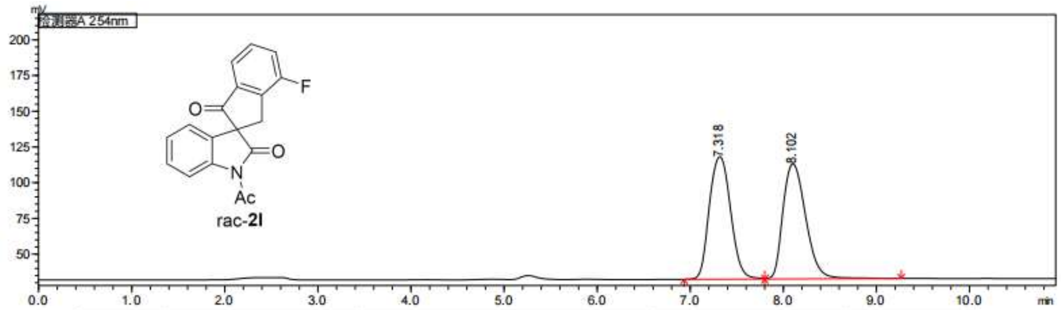

Peak	Time (min)	Area	Height	Concentration	Area (%)
峰号	保留时间	面积	高度	浓度	面积%
1	7.318	1365660	85817	49.846	49.846
2	8.102	1374101	80671	50.154	50.154

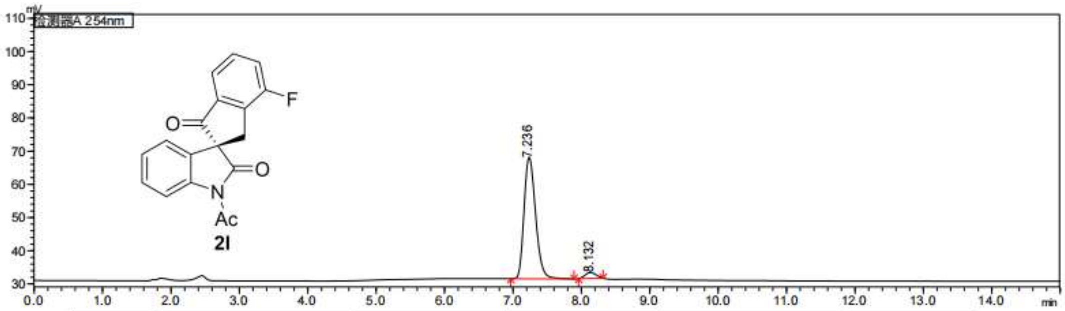

Peak	Time (min)	Area	Height	Concentration	Area (%)
峰号	保留时间	面积	高度	浓度	面积%
1	7.236	429282	36598	95.600	95.600
2	8.132	19758	1786	4.400	4.400

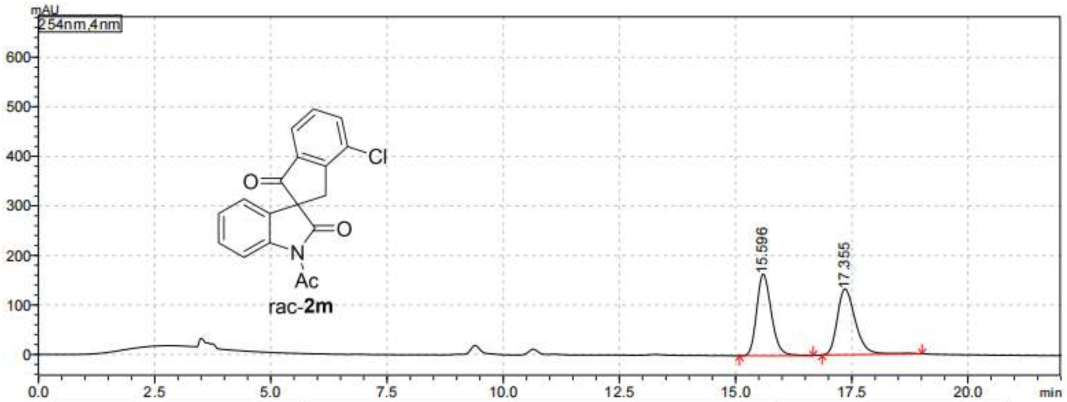

Peak	Time (min)	Area (%)	Area	Height
峰号	保留时间	面积%	面积	高度
1	15.596	50.122	3645454	164774
2	17.355	49.878	3627653	132896

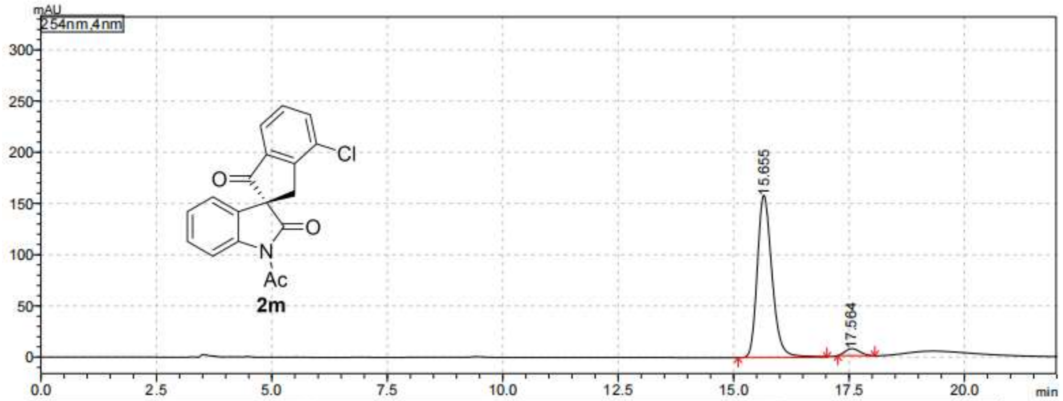

Peak	Time (min)	Area (%)	Area	Height
峰号	保留时间	面积%	面积	高度
1	15.655	95.924	3529906	158530
2	17.564	4.076	149981	6823

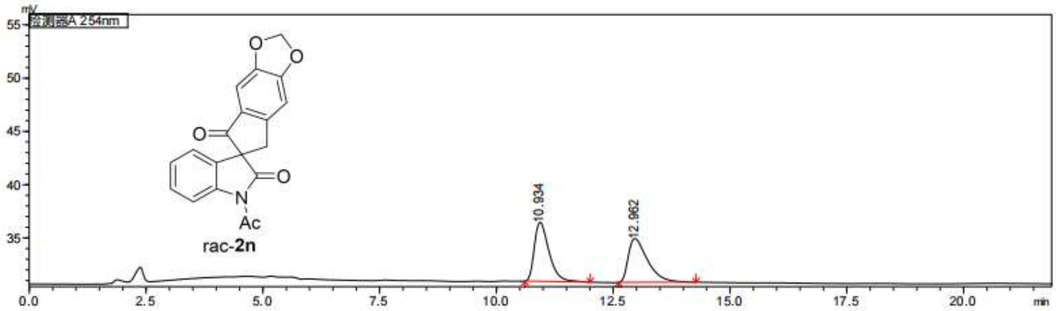

Peak	Time (min)	Area	Height	Concentration	Area (%)
峰号	保留时间	面积	高度	浓度	面积%
1	10.934	121257	5541	50.626	50.626
2	12.962	118256	4108	49.374	49.374

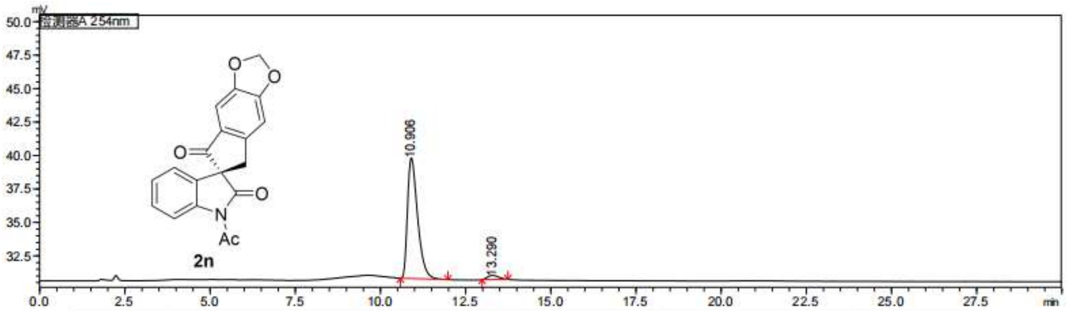

Peak	Time (min)	Area	Height	Concentration	Area (%)
峰号	保留时间	面积	高度	浓度	面积%
1	10.906	186921	9026	96.340	96.340
2	13.290	7102	310	3.660	3.660

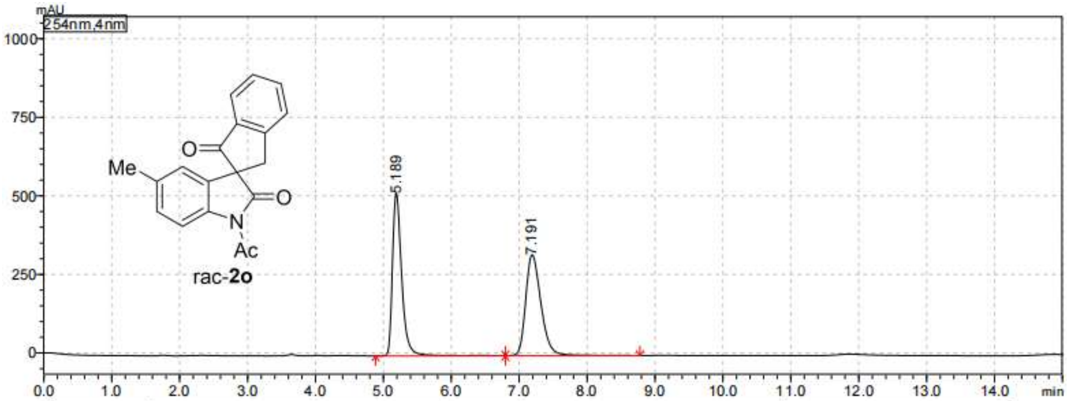

Peak	Time (min)	Area (%)	Area	Height
峰号	保留时间	面积%	面积	高度
1	5.189	49.561	4918793	517926
2	7.191	50.439	5005891	321361

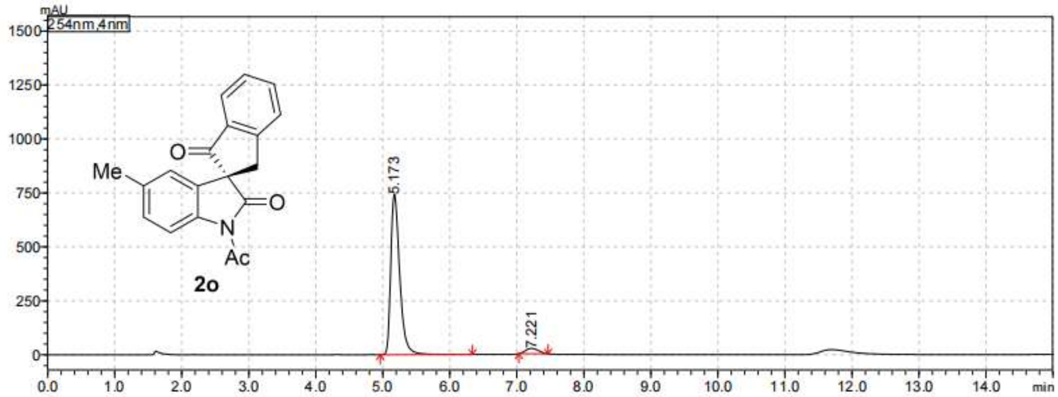

Peak	Time (min)	Area (%)	Area	Height
峰号	保留时间	面积%	面积	高度
1	5.173	95.414	6911078	745010
2	7.221	4.586	332170	25050

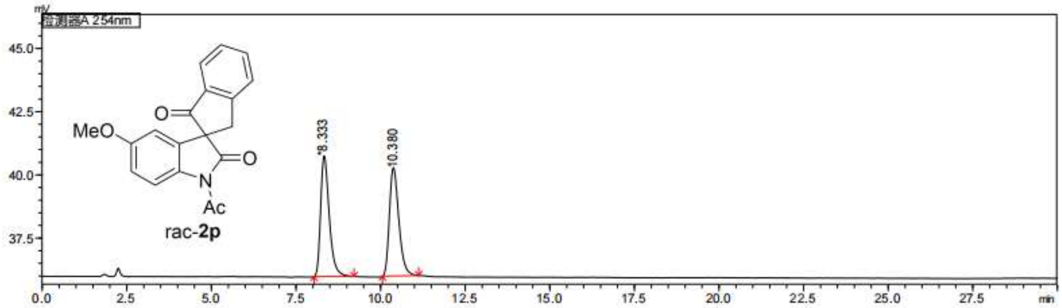

Peak	Time (min)	Area	Height	Concentration	Area (%)
峰号	保留时间	面积	高度	浓度	面积%
1	8.333	84753	4770	0.000	50.486
2	10.380	83121	4286	0.000	49.514

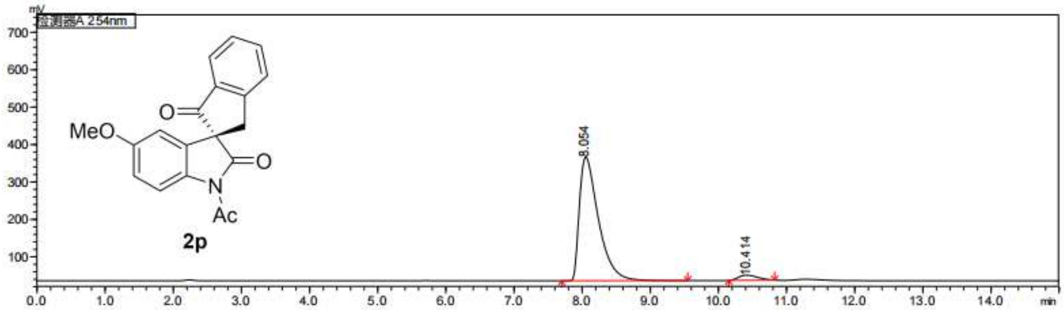

Peak	Time (min)	Area	Height	Concentration	Area (%)
峰号	保留时间	面积	高度	浓度	面积%
1	8.054	6435172	330387	95.970	95.970
2	10.414	270213	13691	4.030	4.030

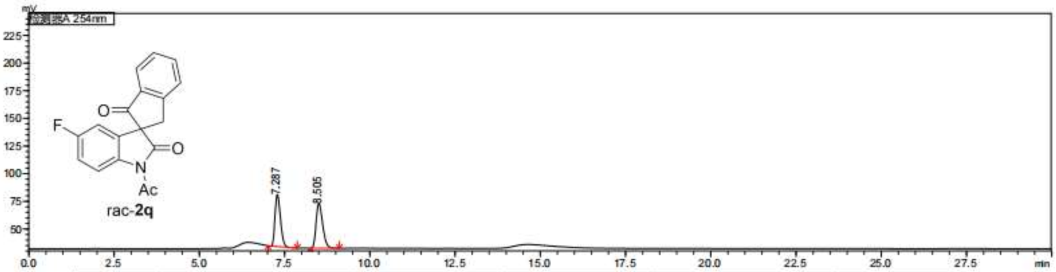

Peak<br>峰号	Time (min)<br>保留时间	Area<br>面积	Height<br>高度	Concentration<br>浓度	Area (%)<br>面积%
1	7.287	520282	46873	49.045	49.045
2	8.505	540542	40266	50.955	50.955

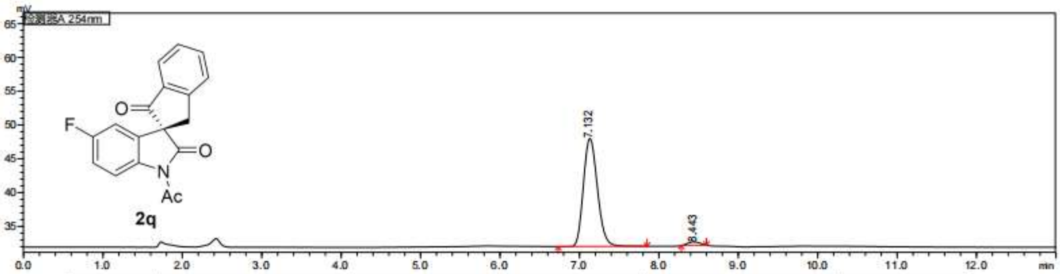

Peak<br>峰号	Time (min)<br>保留时间	Area<br>面积	Height<br>高度	Concentration<br>浓度	Area (%)<br>面积%
1	7.132	195936	15958	97.335	97.335
2	8.443	5365	493	2.665	2.665

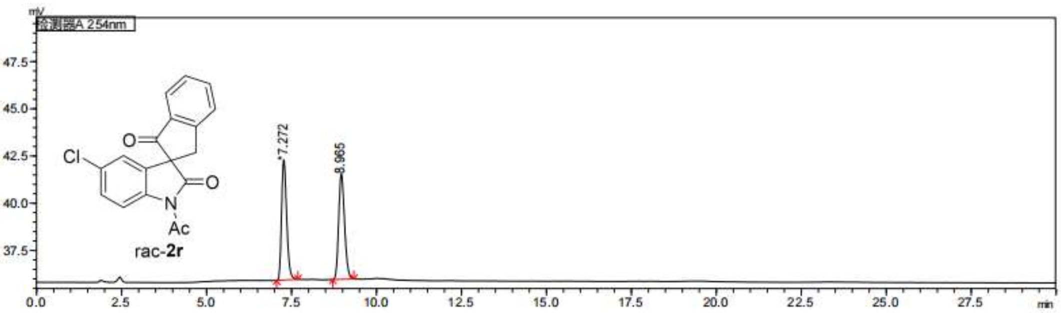

Peak	Time (min)	Area	Height	Concentration	Area (%)
峰号	保留时间	面积	高度	浓度	面积%
1	7.272	66707	6367	0.000	49.737
2	8.965	67413	5556	0.000	50.263

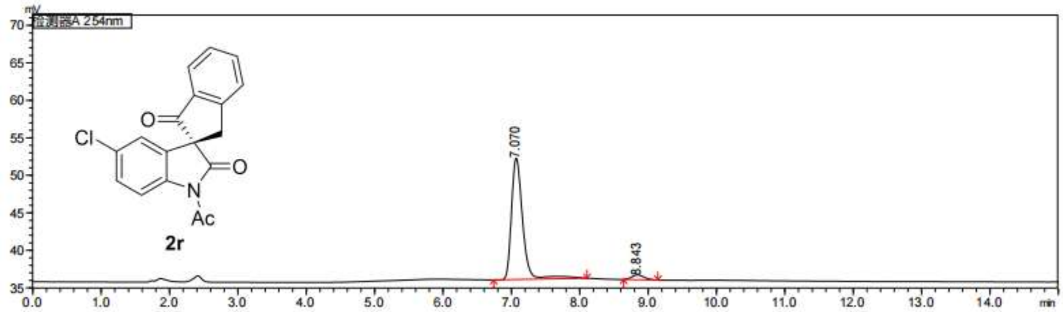

Peak	Time (min)	Area	Height	Concentration	Area (%)
峰号	保留时间	面积	高度	浓度	面积%
1	7.070	185653	16181	95.820	95.820
2	8.843	8099	636	4.180	4.180

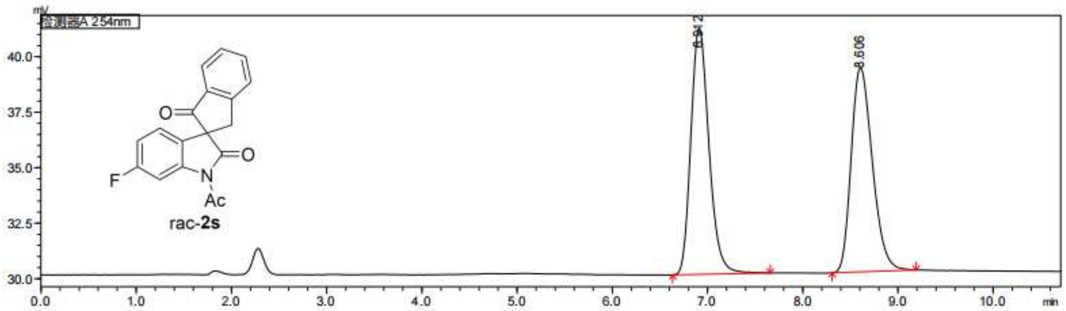

Peak	Time (min)	Area	Height	Concentration	Area (%)
峰号	保留时间	面积	高度	浓度	面积%
1	6.912	146607	11107	50.764	50.764
2	8.606	142196	9212	49.236	49.236

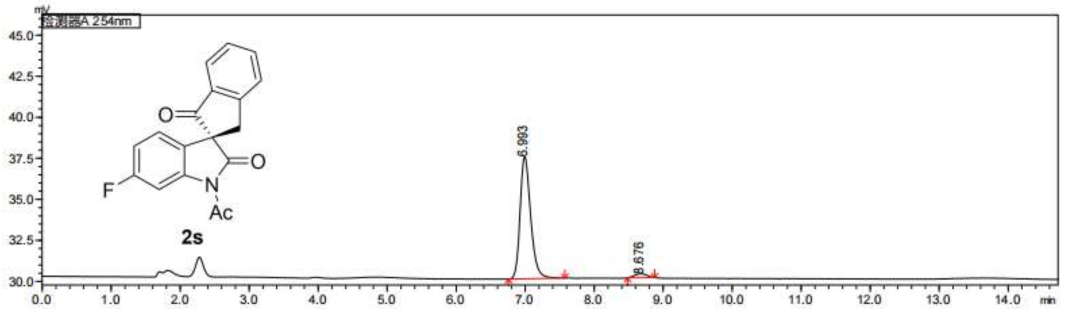

Peak	Time (min)	Area	Height	Concentration	Area (%)
峰号	保留时间	面积	高度	浓度	面积%
1	6.993	79371	7444	96.459	96.459
2	8.676	2914	238	3.541	3.541

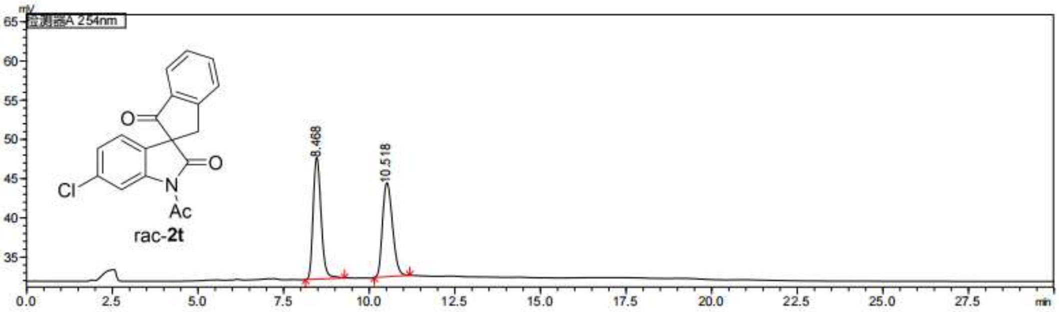

Peak	Time (min)	Area	Height	Concentration	Area (%)
1	8.468	251440	15502	50.926	50.926
2	10.518	242299	11971	49.074	49.074

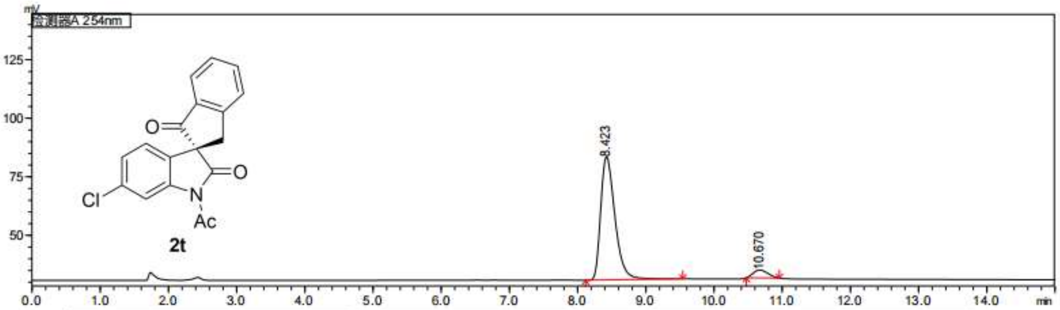

Peak	Time (min)	Area	Height	Concentration	Area (%)
1	8.423	765507	52555	93.888	93.888
2	10.670	49833	3377	6.112	6.112

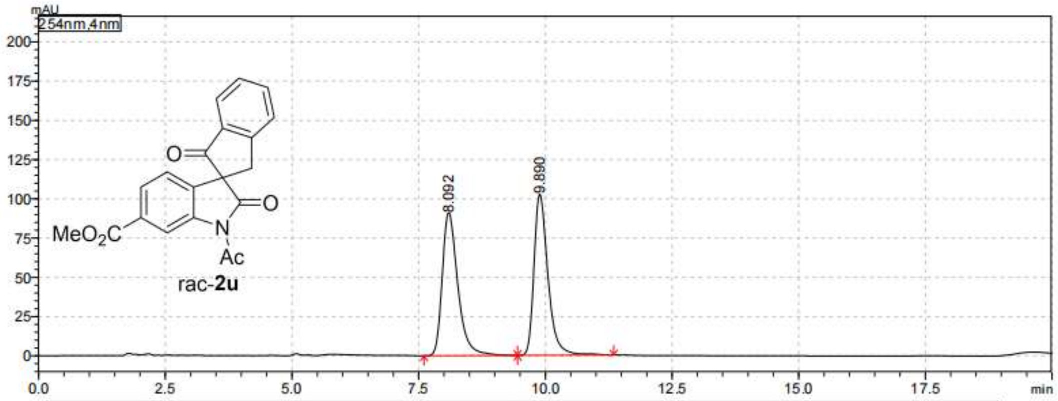

Peak	Time (min)	Area (%)	Area	Height
峰号	保留时间	面积%	面积	高度
1	8.092	49.741	1952408	91101
2	9.890	50.259	1972715	102655

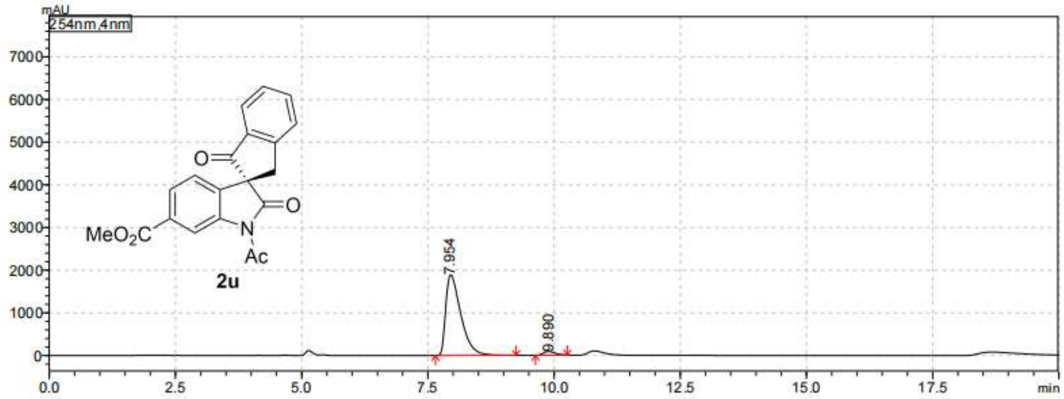

Peak	Time (min)	Area (%)	Area	Height
峰号	保留时间	面积%	面积	高度
1	7.954	96.530	39381288	1888771
2	9.890	3.470	1415466	87221

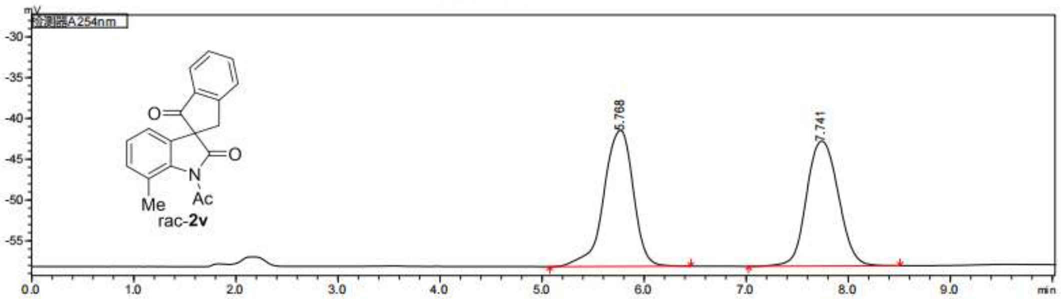

Peak	Time (min)	Area	Height	Concentration	Area (%)
峰号	保留时间	面积	高度	浓度	面积%
1	5.768	336445	16687	50.287	50.287
2	7.741	332606	15307	49.713	49.713

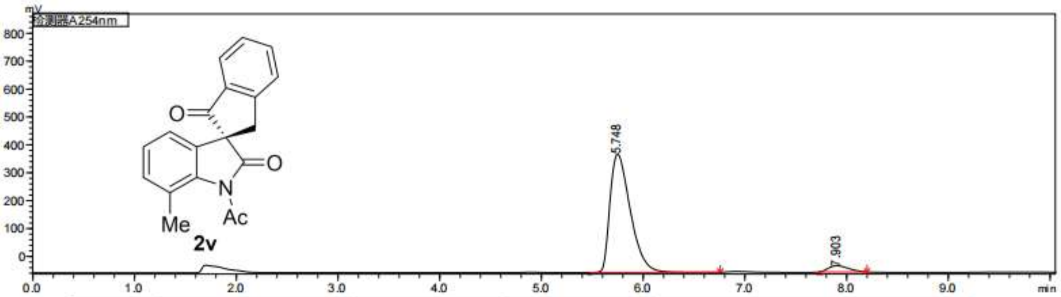

Peak	Time (min)	Area	Height	Concentration	Area (%)
峰号	保留时间	面积	高度	浓度	面积%
1	5.748	5892680	424124	95.155	95.155
2	7.903	300041	21898	4.845	4.845

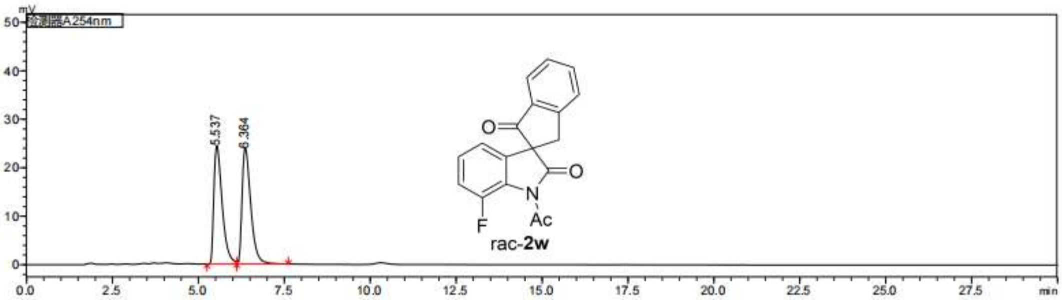

Peak	Time (min)	Area	Height	Concentration	Area (%)

峰号	保留时间	面积	高度	浓度	面积%
1	5.537	413979	24430	49.335	49.335
2	6.364	425144	24001	50.665	50.665

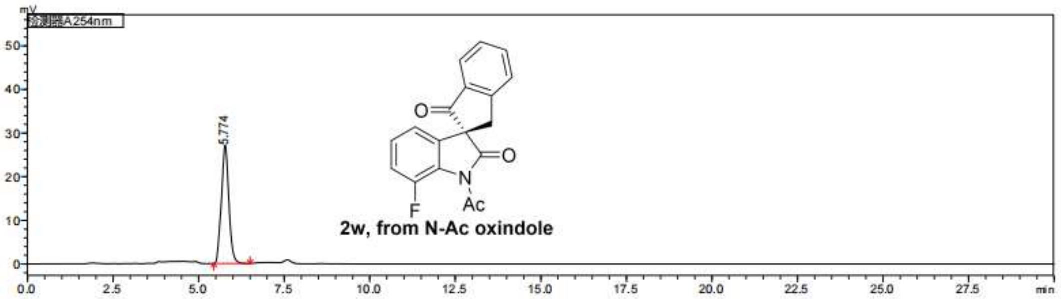

Peak	Time (min)	Area	Height	Concentration	Area (%)

峰号	保留时间	面积	高度	浓度	面积%
1	5.774	414627	27098	100.000	100.000

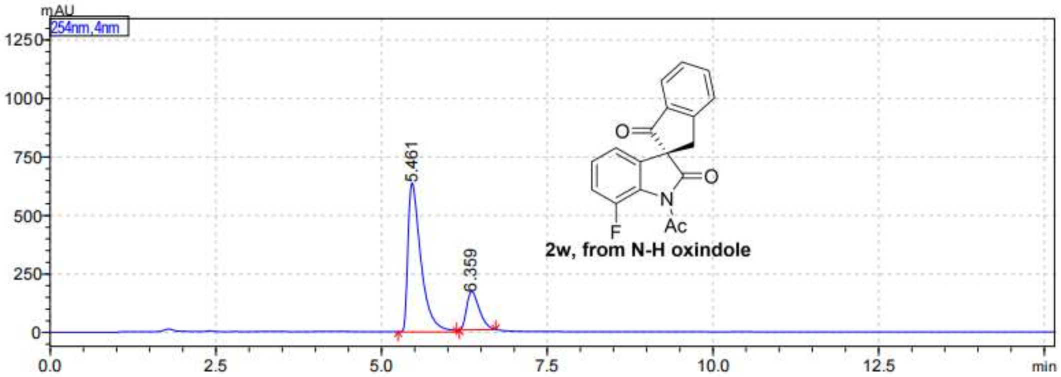

Peak	Time (min)	Area	Height	Concentration	Area (%)

峰号	保留时间	面积%	面积	高度
1	5. 461	79. 977	8715243	636540
2	6. 359	20. 023	2181905	164091

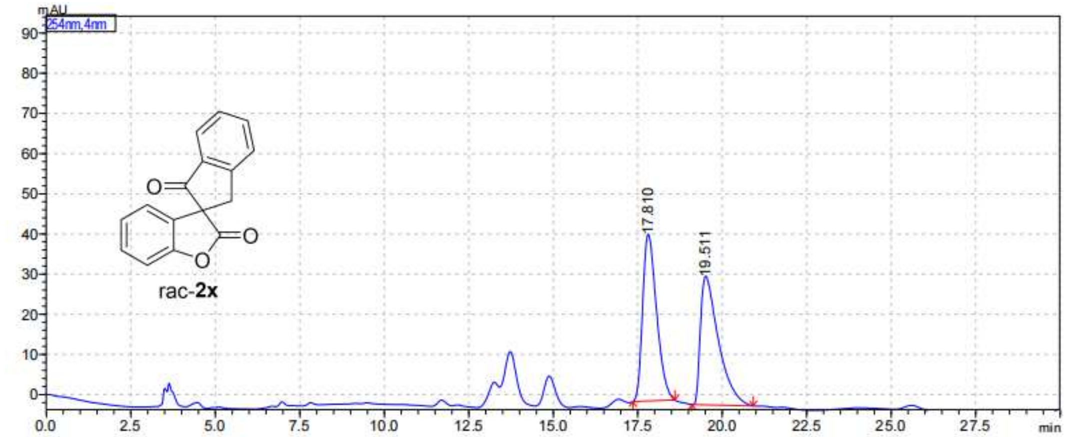

Peak	Time (min)	Area (%)	Area	Height
峰号	保留时间	面积%	面积	高度
1	17.810	50.017	1189383	41564
2	19.511	49.983	1188582	32050

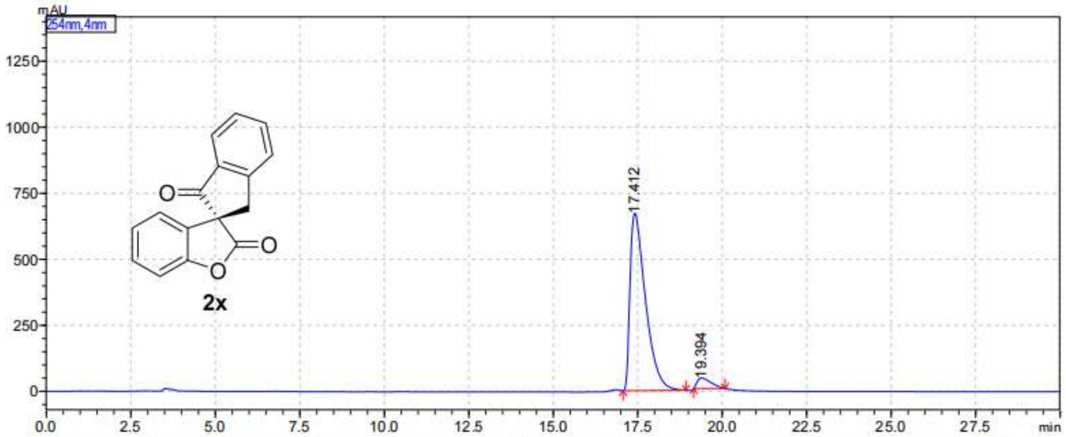

Peak	Time (min)	Area (%)	Area	Height
峰号	保留时间	面积%	面积	高度
1	17.412	94.997	21348360	671794
2	19.394	5.003	1124397	39653

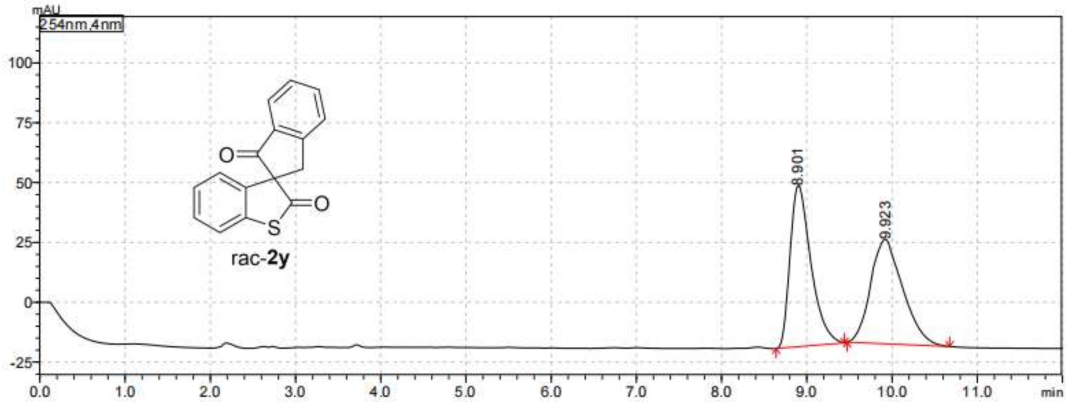

Peak	Time (min)	Area (%)	Area	Height
峰号	保留时间	面积%	面积	高度
1	8.901	50.072	1145543	67274
2	9.923	49.928	1142262	43625

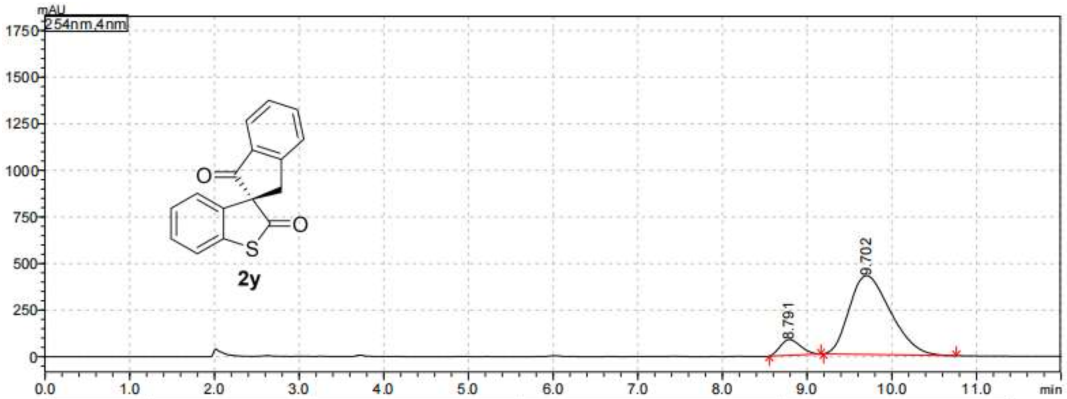

Peak	Time (min)	Area (%)	Area	Height
峰号	保留时间	面积%	面积	高度
1	8.791	8.978	1415815	84992
2	9.702	91.022	14354826	422917

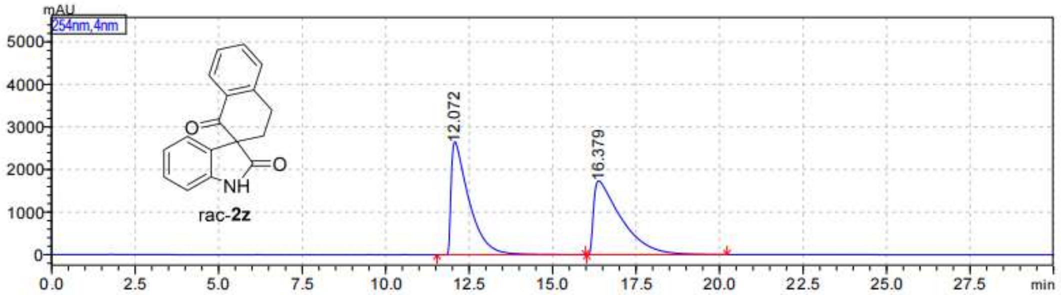

Peak	Time (min)	Area (%)	Area	Height
峰号	保留时间	面积%	面积	高度
1	12.072	49.850	99100871	2654824
2	16.379	50.150	99696402	1731883

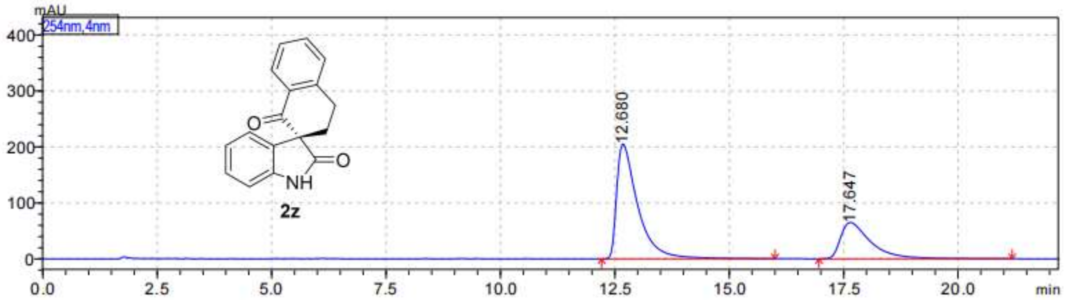

Peak	Time (min)	Area (%)	Area	Height
峰号	保留时间	面积%	面积	高度
1	12.680	68.774	6579442	205022
2	17.647	31.226	2987314	64740

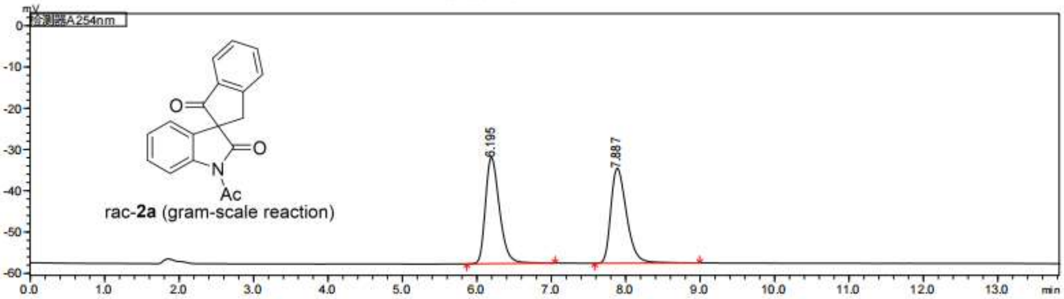

Peak	Time (min)	Area	Height	Concentration	Area (%)
峰号	保留时间	面积	高度	浓度	面积%
1	6.195	339132	25740	49.969	49.969
2	7.887	339548	23005	50.031	50.031

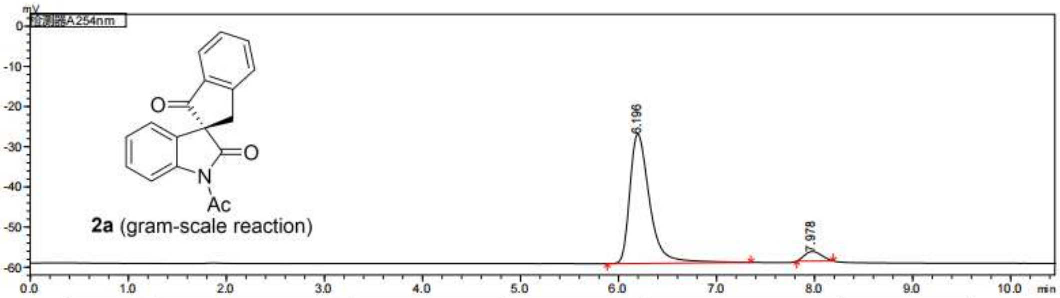

Peak	Time (min)	Area	Height	Concentration	Area (%)
峰号	保留时间	面积	高度	浓度	面积%
1	6.196	454455	32182	94.090	94.090
2	7.978	28545	2375	5.910	5.910

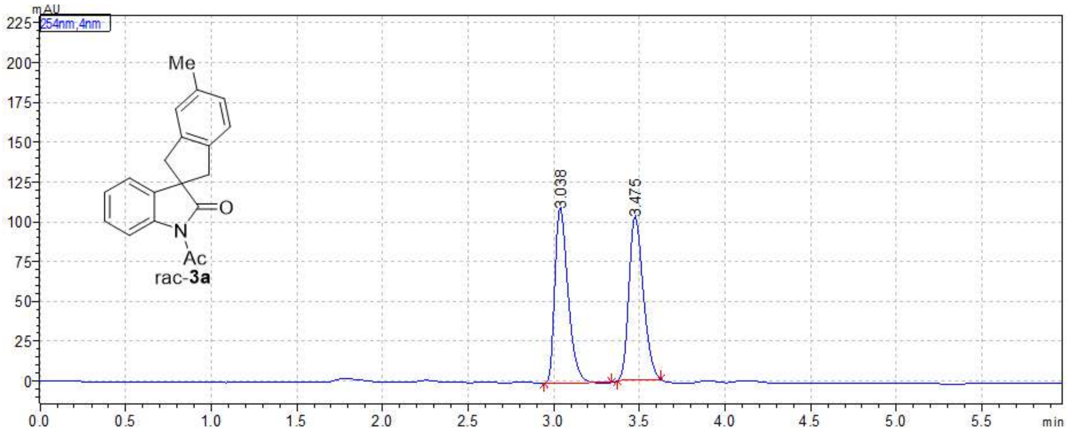

Peak	Time (min)	Area (%)	Area	Height
峰号	保留时间	面积%	面积	高度
1	3. 038	49. 352	585165	110442
2	3. 475	50. 648	600540	103152

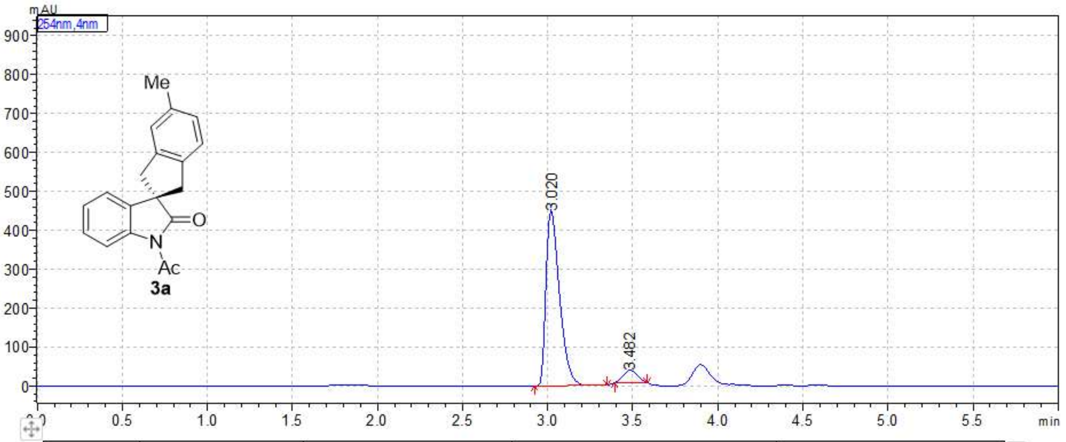

Peak	Time (min)	Area (%)	Area	Height
峰号	保留时间	面积%	面积	高度
1	3. 020	92. 942	2479983	451973
2	3. 482	7. 058	188332	32304

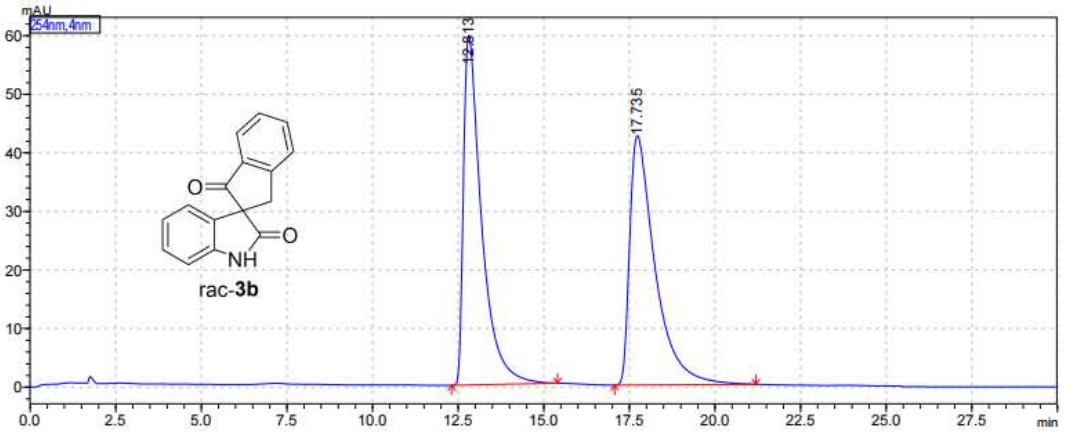

Peak	Time (min)	Area (%)	Area	Height
峰号	保留时间	面积%	面积	高度
1	12.813	50.009	2192796	59724
2	17.735	49.991	2192043	42572

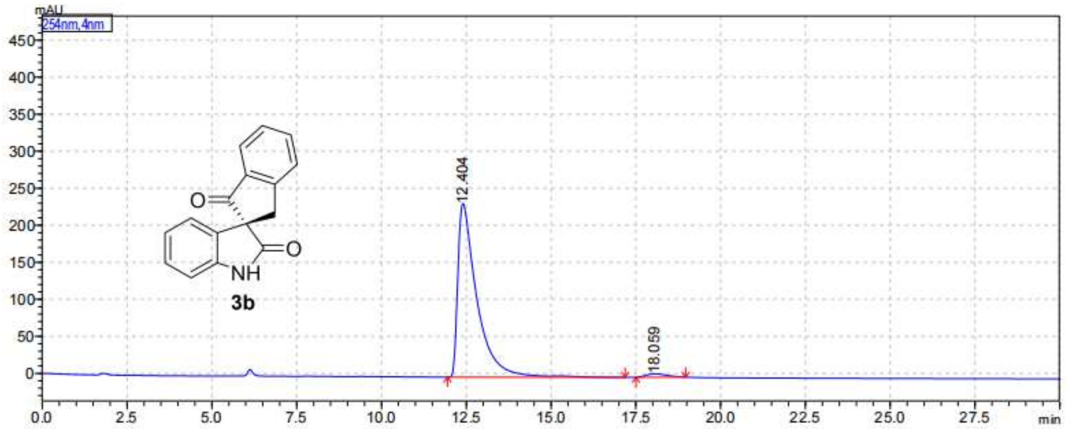

Peak	Time (min)	Area (%)	Area	Height
峰号	保留时间	面积%	面积	高度
1	12.404	97.960	9186002	234459
2	18.059	2.040	191251	4543

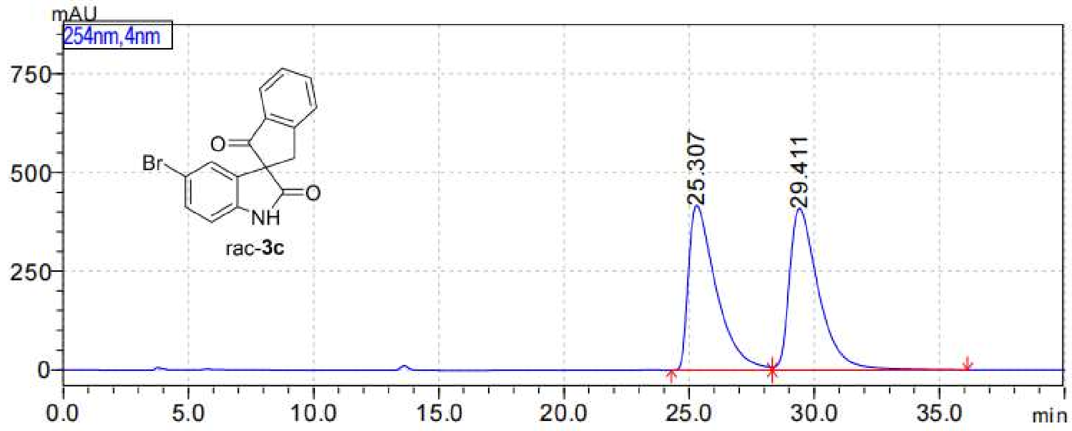

Peak	Time (min)	Area (%)	Area	Height
峰号	保留时间	面积%	面积	高度
1	25. 307	49. 331	32118080	416392
2	29. 411	50. 669	32989464	408706

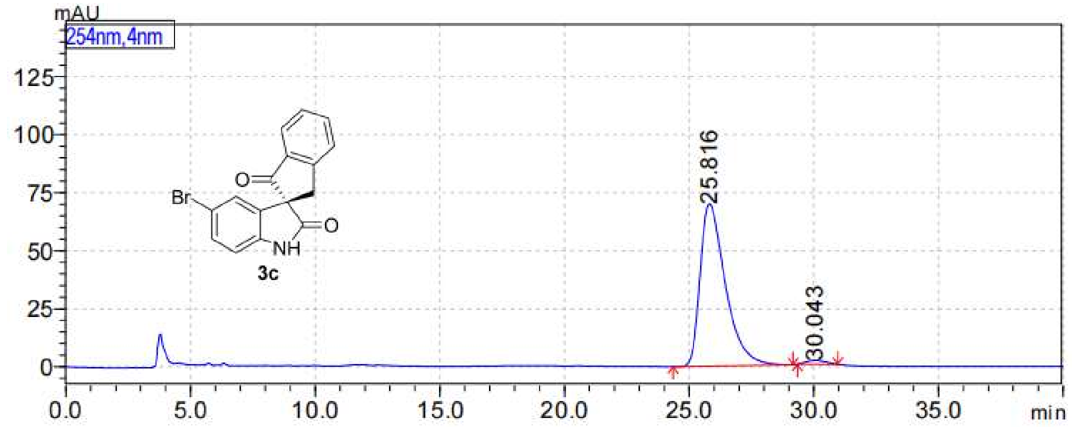

Peak	Time (min)	Area (%)	Area	Height
峰号	保留时间	面积%	面积	高度
1	25. 816	98. 271	4899251	69833
2	30. 043	1. 729	86190	1601

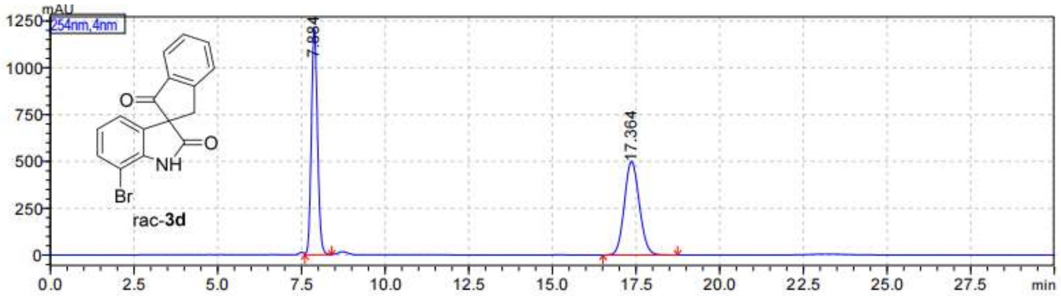

Peak	Time (min)	Area (%)	Area	Height
峰号	保留时间	面积%	面积	高度
1	7. 884	49. 803	15387191	1209655
2	17. 364	50. 197	15508978	500141

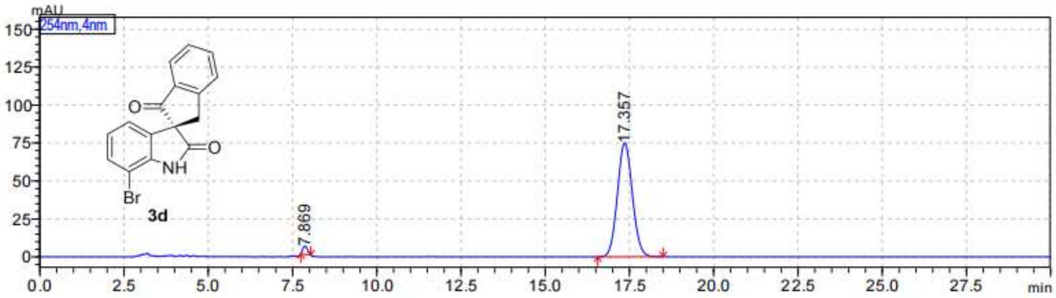

Peak	Time (min)	Area (%)	Area	Height
峰号	保留时间	面积%	面积	高度
1	7. 869	2. 193	52007	5345
2	17. 357	97. 807	2319099	75025

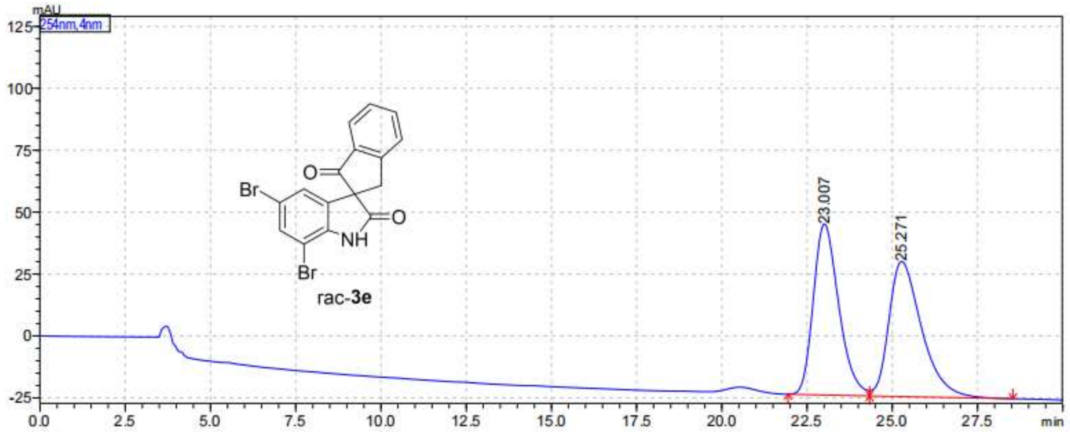

Peak	Time (min)	Area (%)	Area	Height
峰号	保留时间	面积%	面积	高度
1	23.007	49.582	3585394	69102
2	25.271	50.418	3645816	54544

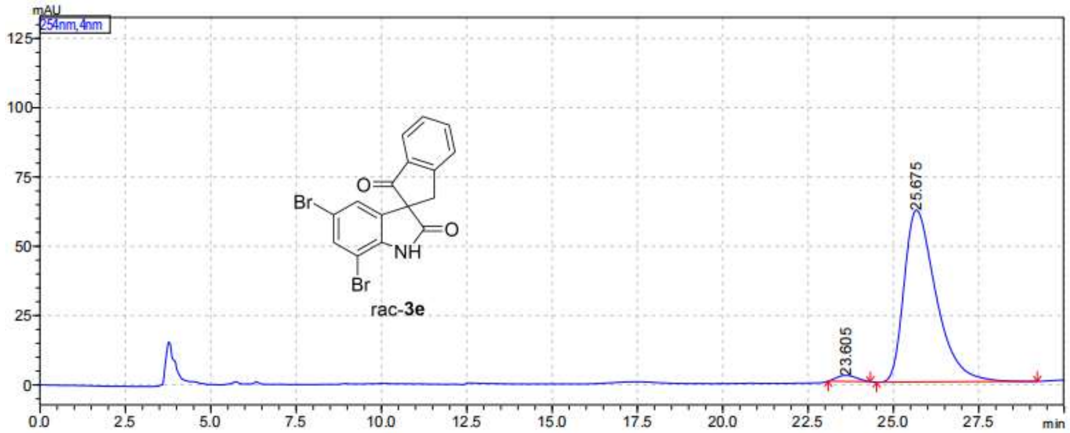

Peak	Time (min)	Area (%)	Area	Height
峰号	保留时间	面积%	面积	高度
1	23.605	2.130	87895	2161
2	25.675	97.870	4038406	62050

9. Crystallographic information

9.1 X-ray crystallography data of 2a

Single crystal was obtained by slow evaporation of a dichloromethane solution of **2a** (>99% ee).

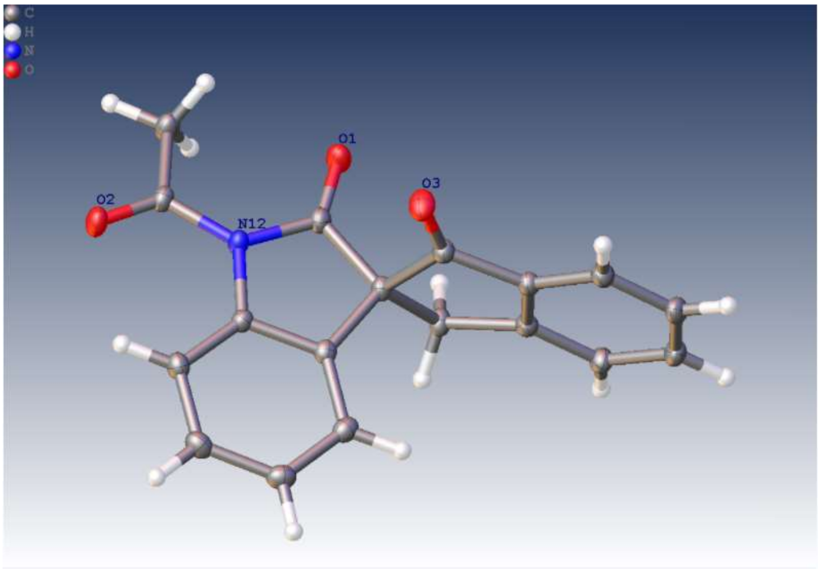

Supplementary Table 13. X-ray crystallography data of **2a** (CCDC No. 2303748) (displacement ellipsoids are drawn at the 50% probability level).

Bond precision: C-C = 0.0023 A Wavelength = 1.34138

Cell: a = 11.7372 (9) b = 8.9494 (9) c = 19.4630 (15)
alpha = 90 beta = 103.491 (3) gamma = 90

Temperature: 100 K

	Calculated	Reported
Volume	2738.5 (4)	2738.5 (4)
Space group	P 21	P 1 21 1
Hall group	P 2yb	P 2yb
Moiety formula	C18 H13 N O3	C18 H13 N O3
Sum formula	C18 H13 N O3	C18 H13 N O3
Mr	291.29	291.29
Dx,g cm-3	1.413	1.413
Z	8	8
Mu (mm-1)	0.504	0.504
F000	1216.0	1216.0
F000’	1218.92	
h, k, lmax	16, 27, 17	16, 27, 17
Nref	15945 [8191]	15502

Tmin, Tmax	0.900, 0.913	0.699, 0.753
Tmin'	0.900	

Correction method = # Reported T Limits: T min=0.699 T max=0.753

AbsCorr = MULTI-SCAN

Data completeness = 1.89/0.97 Theta(max) = 70.539

R(reflections) = 0.0350 (14855) wR2(reflections) = 0.0918 (15502)

S = 1.066 Npar = 798

9.2 X-ray crystallography data of B

Single crystal of compound **B** was obtained by slow evaporation of its THF solution. The single-crystal of compound **B** was measured on a Bruker APEX-II CCD single-crystal diffractometer. The recrystallization solvent of **B** was THF.

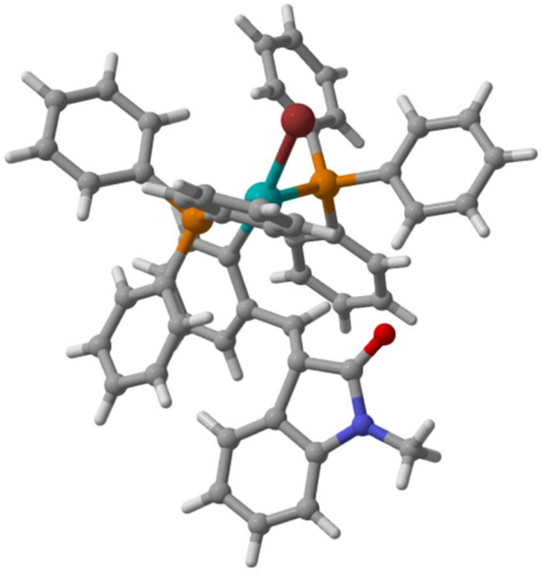

Supplementary Table 14. X-ray crystallography data of **B** (CCDC No. 2308451) (displacement ellipsoids are drawn at the 50% probability level).

Bond precision: C-C = 0.0206 A Wavelength = 1.54178

Cell: a = 12.057 (4) b = 12.844 (4) c = 20.479 (6)
alpha = 101.157 (16) beta = 92.479 (15) gamma = 116.293 (13)

Temperature: 150 K

	Calculated	Reported
Volume	2759.4 (16)	2759.3 (15)
Space group	P -1	P -1
Hall group	-P 1	-P 1
Moiety formula	C52 H42 Br N O P2 Pd	C52 H42 Br N O P2 Pd

	[+solvent]	
Sum formula	C52 H42 Br N O P2 Pd [+solvent]	C52 H42 Br N O P2 Pd
Mr	945.11	945.11
Dx,g cm-3	1.138	1.138
Z	2	2
Mu (mm-1)	4.344	4.345
F000	960.0	960.0
F000’	962.09	
h, k, lmax	14, 15, 24	14, 15, 24
Nref	10152	10068
Tmin, Tmax	0.680, 0.648	0.447, 0.753
Tmin’	0.617	

Correction method = # Reported T Limits: T min=0.447 T max=0.753

AbsCorr = MULTI-SCAN

Data completeness = 0.992 Theta(max) = 68.381

R(reflections) = 0.1099 (6251) wR2(reflections) = 0.3390 (10068)

S = 1.199 Npar = 524

Supplementary References

10. References:

1) Cheng, L.; Liu, L.; Wang, D.; Chen, Y.-J. Highly Enantioselective and Organocatalytic α-Amination of 2-Oxindoles. *Org. Lett.* **2009**, *11*, 3874-3877.

2) Dočekal, V.; Formánek, B.; Císařová, I.; Veselý, J. A formal [4 + 2] cycloaddition of sulfur-containing alkylidene heterocycles with allenic compounds. *Org. Chem. Front.* **2019**, *6*, 3259-3263.

3) Breuers, C. B. J.; Daniliuc, C. G.; Studer, A. Dearomatizing Cyclization of 2-Iodoindoles by Oxidative NHC Catalysis to Access Spirocyclic Indolenines and Oxindoles Bearing an All Carbon Quaternary Stereocenter. *Org. Lett.* **2022**, *24*, 4960-4964.

4) Bedford, R. B.; Haddow, M. F.; Mitchell, C. J.; Webster, R. L. Mild C-H Halogenation of Anilides and the Isolation of an Unusual Palladium(I)-Palladium(II) Species. *Angew. Chem. Int. Ed.* **2011**, *50*, 5524-5527.

5) Mehta, V. P.; Greaney, M. F. S-, N-, and Se-Difluoromethylation Using Sodium Chlorodifluoroacetate. *Org. Lett.* **2013**, *15*, 5036-5039.

6) Sun, G.; Liu, H.; Wang, X.; Zhang, W.; Miao, W.; Luo, Q.; Gao, B.; Hu, J. Palladium-Catalyzed Defluorinative Coupling of Difluoroalkenes and Aryl Boronic Acids for Ketone Synthesis. *Angew. Chem. Int. Ed.* **2023**, *135,* e202213646.

7) Ferguson, D. M.; Bour, J. R.; Canty, A. J.; Kampf, J. W.; Sanford, M. S. Stoichiometric and Catalytic Aryl-Perfluoroalkyl Coupling at Tri-tert-butylphosphine Palladium(II) Complexes. *J. Am. Chem. Soc.* **2017**, *139*, 11662-11665.

8) Wolfe, M. M. W.; Shanahan, J. P.; Kampf, J. W.; Szymczak, N. K. Defluorinative Functionalization of Pd(II) Fluoroalkyl Complexes. *J. Am. Chem. Soc.* **2020**, *142*, 18698-18705.
